# Tunnelling nanotube formation is driven by Eps8/IRSp53‐dependent linear actin polymerization

**DOI:** 10.15252/embj.2023113761

**Published:** 2023-11-27

**Authors:** J Michael Henderson, Nina Ljubojevic, Sevan Belian, Thibault Chaze, Daryl Castaneda, Aude Battistella, Quentin Giai Gianetto, Mariette Matondo, Stéphanie Descroix, Patricia Bassereau, Chiara Zurzolo

**Affiliations:** ^1^ Membrane Traffic and Pathogenesis Unit, Department of Cell Biology and Infection CNRS UMR 3691, Université de Paris, Institut Pasteur Paris France; ^2^ Institut Curie, Université PSL, Sorbonne Université, CNRS UMR 168, Laboratoire Physico‐Chimie Curie Paris France; ^3^ Sorbonne Université Paris France; ^4^ Université Paris‐Saclay Gif‐sur‐Yvette France; ^5^ Proteomics Platform, Mass Spectrometry for Biology Unit, CNRS USR 2000, Institut Pasteur Paris France; ^6^ Keele University Keele UK; ^7^ Bioinformatics and Biostatistics Hub, Computational Biology Department CNRS USR 3756, Institut Pasteur Paris France; ^8^ Institut Pierre‐Gilles de Gennes Paris France; ^9^ Department of Molecular Medicine and Medical Biotechnology University of Naples Federico II Naples Italy; ^10^ Present address: Department of Chemistry Bowdoin College Brunswick ME USA

**Keywords:** actin cytoskeleton, cell biophysics, proteomics, tunnelling nanotubes, Cell Adhesion, Polarity & Cytoskeleton

## Abstract

Tunnelling nanotubes (TNTs) connect distant cells and mediate cargo transfer for intercellular communication in physiological and pathological contexts. How cells generate these actin‐mediated protrusions to span lengths beyond those attainable by canonical filopodia remains unknown. Through a combination of micropatterning, microscopy, and optical tweezer‐based approaches, we demonstrate that TNTs formed through the outward extension of actin achieve distances greater than the mean length of filopodia and that branched Arp2/3‐dependent pathways attenuate the extent to which actin polymerizes in nanotubes, thus limiting their occurrence. Proteomic analysis using epidermal growth factor receptor kinase substrate 8 (Eps8) as a positive effector of TNTs showed that, upon Arp2/3 inhibition, proteins enhancing filament turnover and depolymerization were reduced and Eps8 instead exhibited heightened interactions with the inverted Bin/Amphiphysin/Rvs (I‐BAR) domain protein IRSp53 that provides a direct connection with linear actin polymerases. Our data reveals how common protrusion players (Eps8 and IRSp53) form tunnelling nanotubes, and that when competing pathways overutilizing such proteins and monomeric actin in Arp2/3 networks are inhibited, processes promoting linear actin growth dominate to favour tunnelling nanotube formation.

## Graphical abstract

Inhibition of branched actin networks reveals the role of linear actin filament polymerization in the formation of functional tunnelling nanotubes.

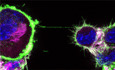

## Introduction

Intercellular communication is vital for organisms, mediating across different length scales events like differentiation, tissue development/homeostasis, and wound healing. One mechanism relies on the generation of filamentous actin (F‐actin)‐based protrusions in which the plasma membrane (PM) envelopes a core of 10–30 bundled, parallel‐oriented filaments that emanate outwards from the cell periphery (Mattila & Lappalainen, 2008; Blake & Gallop, 2023). Typically projecting only a few microns (< 5 μm) from the cell, conventional filopodia are thin (diameters of the order of several hundred nanometres), finger‐like structures that dynamically probe the environment through repeated cycles of growth and retraction, establishing focal adhesions through activated integrins at their tip (Blake & Gallop, 2023) or cell–cell signalling important for collective migration (Bischoff *et al*, 2021), tissue closure (Jacinto *et al*, 2000), and neurogenesis (Mancinelli *et al*, 2021). To establish cell‐to‐cell communication at longer distances spanning several cell diameters, cells remarkably construct actin‐based protrusions such as cytonemes (often termed as specialized signalling filopodia) (Yamashita *et al*, 2018) and tunnelling nanotubes (TNTs) (Ljubojevic *et al*, 2021) that, while exhibit comparable widths to conventional filopodia, are able to reach significantly longer distances (tens of microns). For clarity, we will refer to the short, dynamic protrusions cells make simply as filopodia or qualify them as conventional (canonical) filopodia.

While both cytonemes and TNTs are similar in terms of their capacity to reach extreme intercellular distances, the two differ in their cellular functionality. Cytonemes, originally identified in the developing wing tissue of *Drosophila* (Felipe‐Andrés & Kornberg, 1999), direct morphogen signalling such as the binding of Wnt ligands with Frizzled receptors on another cell (Stanganello & Scholpp, 2016) through closed‐ended structures. Contrastingly, TNTs instead were functionally found to cytoplasmically connect distant cells (Rustom *et al*, 2004), allowing the direct transfer of cargo ranging from Ca^2+^ ions (Wang *et al*, 2010), RNAs (Haimovich *et al*, 2017), and plasma membrane‐resident proteins (e.g., H‐Ras, Fas/CD95) (Arkwright *et al*, 2010; Rainy *et al*, 2013), to lysosomes (Abounit *et al*, 2016) and mitochondria (Wang & Gerdes, 2015; Sartori‐Rupp *et al*, 2019; Saha *et al*, 2022). This material transport ability has transformed the way we think about cell identity and the establishment of cellular networks (Rustom, 2016; Zurzolo, 2021). For example, *in vivo* evidence has supported a physiological role for TNT‐like structures in vascular coupling within the retina (Alarcon‐Martinez *et al*, 2020) and in facilitating cytoplasmic and membrane‐bound material transfer between photoreceptors (Kalargyrou *et al*, 2021; Ortin‐Martinez *et al*, 2021), implicating nanotube‐mediated maintenance in proper tissue function within the nervous system. Beyond their physiological involvement in signal transduction, apoptosis, and immune responses (Arkwright *et al*, 2010; Sisakhtnezhad & Khosravi, 2015), TNTs can contribute to cancer progression (Saha *et al*, 2022), HIV‐1 and SARS‐CoV‐2 transmission (Sowinski *et al*, 2008; Pepe *et al*, 2022) and the propagation of misfolded proteins (e.g., α‐synuclein, Tau, huntingtin) involved in neurodegenerative diseases (Abounit *et al*, 2016; Sharma & Subramaniam, 2019; Chastagner *et al*, 2020; Scheiblich *et al*, 2021). Thus, a key distinguishing characteristic of TNTs is their unique functional ability to transfer cargo, a property absent for filopodia as previous ultrastructural studies have found no vesicles nor other organelles within them (Medalia *et al*, 2007; Almagro *et al*, 2010; Aramaki *et al*, 2016; Mageswaran *et al*, 2021).

For protrusions to form, F‐actin remodelling processes comprising initiation, polymerization, and bundle stabilization need to be spatiotemporally controlled at the PM in order to work against membrane tension resistance to form a tube (Mogilner & Rubinstein, 2005). Proteins like IRSp53 (insulin receptor tyrosine kinase substrate protein of 53 kDa) of the Bin/Amphiphysin/Rvs (BAR) family orchestrate membrane shape changes and actin assembly in filopodia, microvilli, and dendritic spine formation (Choi *et al*, 2005; Disanza *et al*, 2013; Garbett *et al*, 2013). Through its crescent‐shaped inverse BAR domain IRSp53 is able to induce, sense, and stabilize negative curvatures found in cellular protrusions (Saarikangas *et al*, 2009; Prévost *et al*, 2015). Its CRIB (Cdc42/Rac interactive binding) domain permits regulatory control by Rho GTPases (Disanza *et al*, 2006, 2013), while its SH3 (Src homology 3) domain mediates interactions with actin polymerases like VASP (vasodilator‐stimulated phosphoprotein) (Vaggi *et al*, 2011; Disanza *et al*, 2013) and the formin mDia1 (Goh *et al*, 2012), and nucleating promoting factors of the actin‐related protein 2/3 (Arp2/3) complex like N‐WASP (Lim *et al*, 2008) and WAVE2 (Miki *et al*, 2000; Goh *et al*, 2012). Thus, IRSp53 self‐assembly at the PM is sufficient to generate forces for protrusion growth by recruiting polymerases and locally polymerizing F‐actin at IRSp53 clusters (Disanza *et al*, 2013; Tsai *et al*, 2022).

A key partner of I‐BAR proteins in filopodia and microvilli formation is Eps8 (epidermal growth factor receptor kinase substrate 8) (Disanza *et al*, 2006; Sudhaharan *et al*, 2016; Postema *et al*, 2018). Eps8 was identified in a complex with Abi‐1 (Abelson interactor 1) and Sos‐1 (Son of sevenless homolog 1) that activates Rac (Scita *et al*, 1999, 2001) for branched actin formation by Arp2/3 (Yang & Svitkina, 2011). Eps8 displays actin regulatory behaviour that depends on its binding partners: interaction with IRSp53 enhances Eps8's F‐actin bundling activity, while binding with Abi‐1 prompts its capping activity (Disanza *et al*, 2004, 2006; Hertzog *et al*, 2010; Vaggi *et al*, 2011). We recently demonstrated that Eps8's actin bundling activity promotes the formation of functional TNTs in neuronal cells (Delage *et al*, 2016), and our cryogenic electron microscopy (cryo‐EM) work further showed that while TNTs and conventional filopodia showed similarities in both actin filament count and spacing, the filaments within TNTs were instead more continuous and linearly organized (Sartori‐Rupp *et al*, 2019), suggesting a switch in common actin‐regulating components in generating shorter canonical filopodia versus longer TNTs. The mechanism(s) by which the cell uses a common pool of actin‐interfacing proteins to establish length scales characteristic of intercellular protrusions like TNTs, or even cytonemes, is unclear.

Using TNTs as a model system, we addressed this issue using micropatterning to control the distances over which actin‐dependent processes, and not mechanisms based on cells migrating apart forming TNTs through cell dislodgement (Matejka & Reindl, 2019; Ljubojevic *et al*, 2021), lead to TNT formation at lengths beyond those of canonical filopodia. We found that Arp2/3‐dependent pathways deter TNTs and that upon Arp2/3 inhibition, F‐actin polymerizes over longer distances in nanotubes. Arp2/3 inhibition further promoted a specific interaction between Eps8 and IRSp53, and not with other I‐BAR proteins, and their co‐expression facilitated functional TNT formation. Proteomic analysis revealed that Eps8's interactions with branched actin networks and proteins influencing filament turnover and depolymerization are reduced upon Arp2/3 inhibition, while IRSp53's interactome remained unchanged and provided connections to needed polymerases. Our data show that protrusion length results from a competing balance between the utilization of monomeric actin and the availability of actin regulatory proteins in order to build distinct F‐actin networks in a common cytoplasm.

## Results

### TNTs connect micropatterned cells at greater distances compared to filopodia

**Figure 1:**
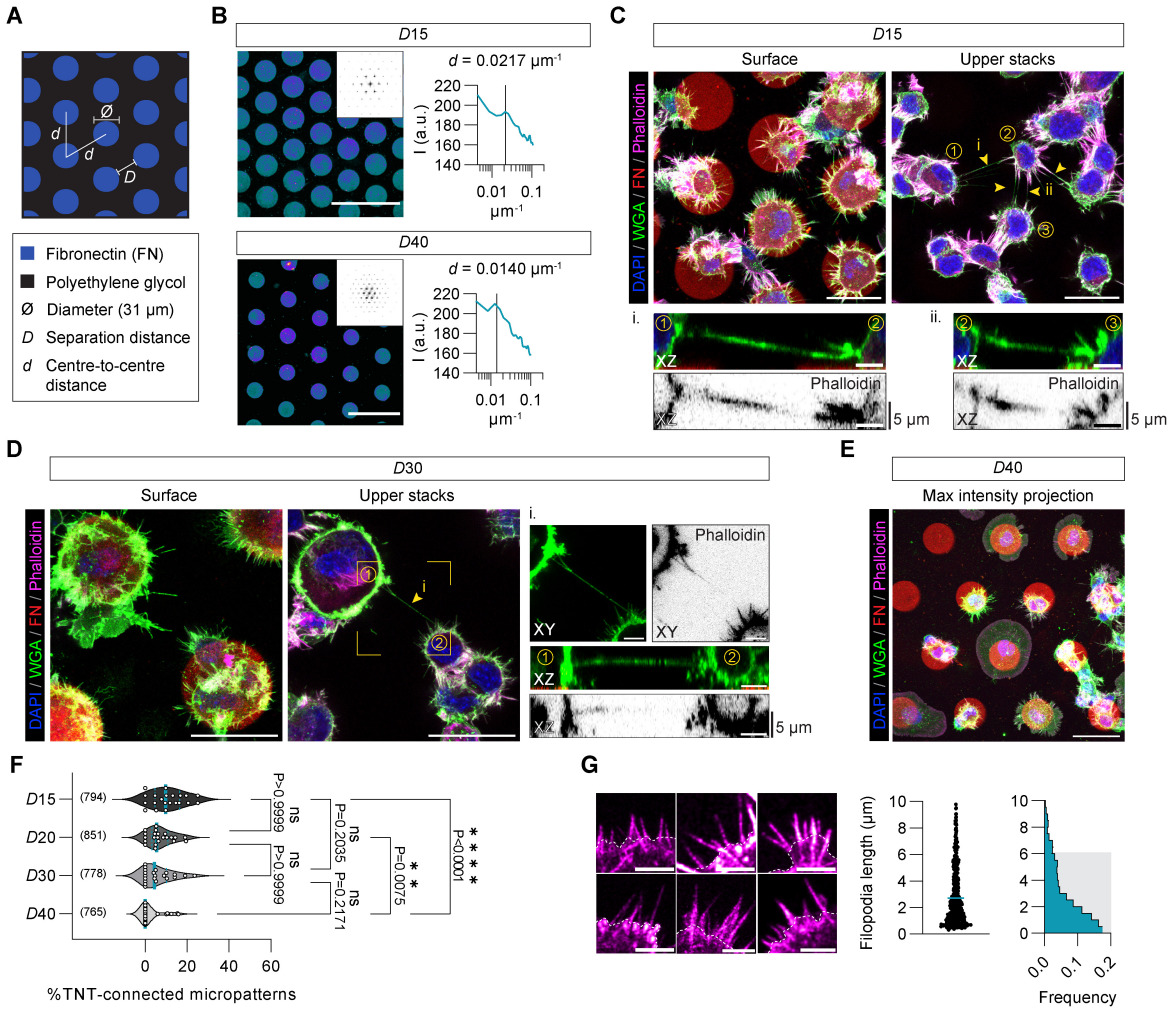
TNTs connect neighbouring CAD cells across varying distances Schematic depicting fibronectin (FN) patterns produced as a hexagonal array of circles (diameter of 31 μm) with edge‐to‐edge separation distances (*D*) of 15, 20, 30, and 40 μm tuned along the centre‐to‐centre distance (*d*) between circles.Left: Representative fluorescent images of FN micropatterns spaced by 15 and 40 μm with their respective calculated fast Fourier transform (FFT) images (upper right insets); Scale bars, 100 μm. Right: Corresponding radial profiles determined from the FFT images plotted as a function of the spatial frequency (μm^−1^). Solid lines in the radial profile plots indicate the expected centre‐to‐centre *d* spacing between circular patterns and line up nicely with peaks in the measured profile, indicating high fidelity in the manufacturing process.Representative surface and upper stack maximum intensity projections of CAD cells plated on 15 μm FN micropatterns; Scale bars, 30 μm. TNTs connecting cells on two different micropatterns are annotated with yellow arrowheads. Subpanels (i, ii) show XZ projections made through the long axis of the indicated TNTs; Scale bars, 5 μm. Cells were fixed and stained with DAPI (blue), AX‐488 WGA (green) and AX‐647 Phalloidin (magenta); micropatterns were visualized using Rhodamine FN (red).Representative surface and upper stack maximum intensity projections of CAD cells plated on 30 μm FN micropatterns; Scale bars, 30 μm. The yellow arrowhead indicates a TNT connecting cells on neighbouring micropatterns. Subpanels in i show an enlargement of the TNT‐connected cells (indicated by the yellow box) and XZ projections made through the long axis of the indicated TNT; Scale bars, 5 μm. Cells were fixed and stained with DAPI (blue), AX‐488 WGA (green) and AX‐647 Phalloidin (magenta); micropatterns were visualized using Rhodamine FN (red).Representative maximum intensity projection of CAD cells plated on 40 μm FN micropatterns showing no TNTs connecting cells; Scale bar, 30 μm. Cells were fixed and stained with DAPI (blue), AX‐488 WGA (green) and AX‐647 Phalloidin (magenta); micropatterns were visualized using Rhodamine FN (red).Violin plot of the percentage of TNT‐connected micropatterns for *D*15, *D*20, *D*30 and *D*40 micropatterns. The median percentage of TNT‐connected micropatterns (solid teal lines in the plot) on *D*15, *D*20, *D*30 and *D*40 micropatterns is 9.8%, 5.5%, 4.4%, and 0%, respectively; quartiles are annotated with dotted teal lines. Each data point corresponds to a quantified image in which on average approximately 30 cell‐occupied micropatterns were within the acquired field of view; data was pooled from three experiments. The total number of individual micropatterns quantified per condition is indicated in parentheses. Statistical analysis was performed using a Kruskal Wallis test with Dunn's multiple comparison test. Adjusted *P* values for each comparison are provided on the plot.Left: Representative images of CAD cell filopodia (magenta, AX‐647 Phalloidin); Scale bars, 5 μm. Cell body edges are marked with a dotted white line. Right: Dot plot of individual filopodia lengths (18 cells, *n* = 548 filopodia) and the corresponding histogram showing that 90% of the population (shaded grey region) had lengths that were less than 6 μm. Source data are available online for this figure.

**Figure EV1:**
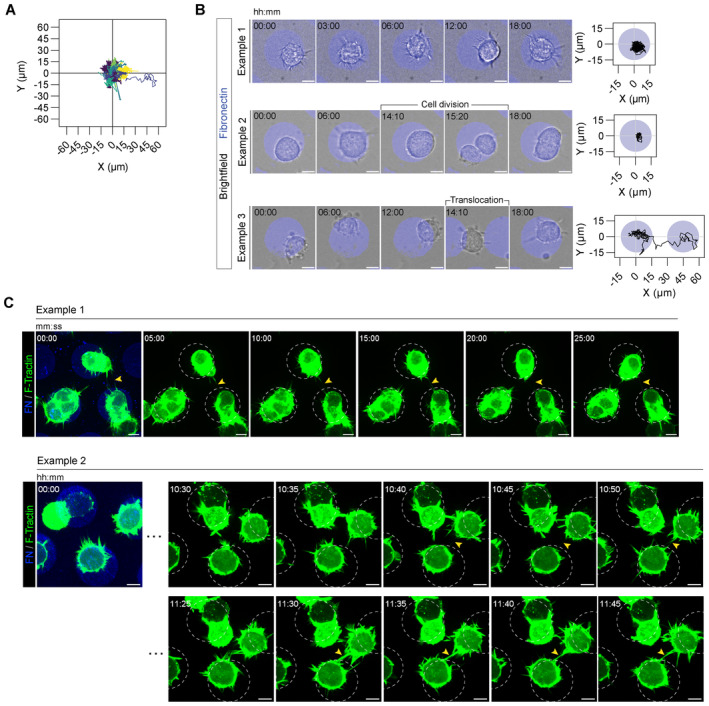
TNT formation between micropatterned cells does not occur through cell dislodgment A, BAssessment of CAD cell confinement over an extended period of time (18 h) on *D*15 fibronectin micropatterns. (A) Plot of individual cell trajectories normalized with respect to the centre of their micropattern (*n* = 56 trajectories). (B) Representative time‐lapse images of CAD cells and their corresponding trajectories. Fibronectin micropatterns are false‐coloured in blue (Rhodamine fibronectin). Scale bars, 10 μm.CSelected time frames from a 30 min (Example 1, Movie EV5) and an overnight (Example 2, Movie EV6) acquisition showing TNT‐like protrusion formation between *D*15 micropatterned cells (expressing F‐Tractin, green) occurs through actin‐based protrusions. Images of the F‐Tractin channel are max intensity projections of the upper stacks in the acquired Z range and were overlayed with the fibronectin channel to highlight cell residency to the micropatterns. White dotted circles annotate the AX‐405‐labelled fibronectin patterns (blue) shown on the left, and yellow arrowheads point to representative TNTs. Scale bars, 10 μm.

How TNTs form and connect cells has remained elusive largely due to standard cell culture methods, as they lack the ability to control cell plating and intercellular distances. Moreover, as TNTs are morphologically similar to filopodia, standard 2D culture methods hamper the proper identification of TNTs if cells are grown in an unconstrained manner where the distance between cultured cells approaches the typical length scales that canonical filopodia are able to achieve. Thus, to assess TNT formation as a function of intercellular distances, we fabricated hexagonal arrays of circular fibronectin (FN) micropatterns of constant diameter (31 μm) in which the separation distance *D* (Fig 1A and B, Appendix Fig S1A) was tuned to be beyond the reach of canonical filopodia which typically ranges 1–2 μm and rarely exceeds 10 μm (Mogilner & Rubinstein, 2005; Mattila & Lappalainen, 2008). CAD cell adherence was first optimized on appropriately sized patterns to prevent unwanted cell infiltration into the surrounding PEG region (Appendix Fig S1B–E and Movie EV1), ensuring that the connections we observe come from actin‐driven mechanisms, and not from cell dislodgement. Overnight cell tracking (18 h) confirmed that CAD cells remained resident to their initial micropatterns (Fig EV1A and B, Example 1 and Movie EV2), even during mitosis (Fig EV1B, Example 2 and Movie EV3). Cells rarely exhibited events of inter‐micropattern translocation where only 1 event out of 56 was observed (Fig EV1A and B, Example 3 and Movie EV4). Furthermore, live‐cell imaging of patterned cells expressing the F‐actin reporter F‐Tractin showed that TNTs originate from dorsal generated actin‐based protrusions that reach a neighbouring cell to form a stable cell‐to‐cell connection (Fig EV1C and Movies EV5 and EV6).

Thus, after establishing a micropatterning approach that faithfully ensures actin‐driven TNT formation, we evaluated the propensity for TNTs to form as a function of increasing intercellular distances; cells were fixed following a 16–18 h overnight culture on the patterns to give sufficient time for a basal level of TNTs to form (see Materials and Methods). We identified thin, substrate‐detached TNT‐like protrusions positive for F‐actin connecting cells on neighbouring patterns at 15–30 μm (Fig 1C and D), and containing DiD‐labelled vesicles (Appendix Fig S2A). Using a donor‐acceptor co‐culture where we can discriminate the two cell populations (Appendix Fig S2B), we found DiD vesicles both in the histone 2B (H2B)‐positive acceptor cells (Appendix Fig S2C, Example 1) and in the connecting TNTs (Appendix Fig S2C, Example 2) supporting TNT‐mediated cargo transport. After increasing *D* to 40 μm, we observed fewer TNTs (Fig 1E). *D*15 and *D*20 patterns showed the highest TNT frequency, while approaching *D*40, there was a significant decrease (Fig 1F), indicating a distance dependency. On the other hand, filopodia lengths showed that a majority of filopodia (90% of the population) were far shorter and did not exceed 6 μm with an average length of 2.7 μm (Fig 1G). These data show that TNTs are able to reach considerably longer lengths than average filopodia, yet there seems to be an upper limit to F‐actin based elongation.

### Inhibition of branched actin networks favours TNTs

**Figure 2:**
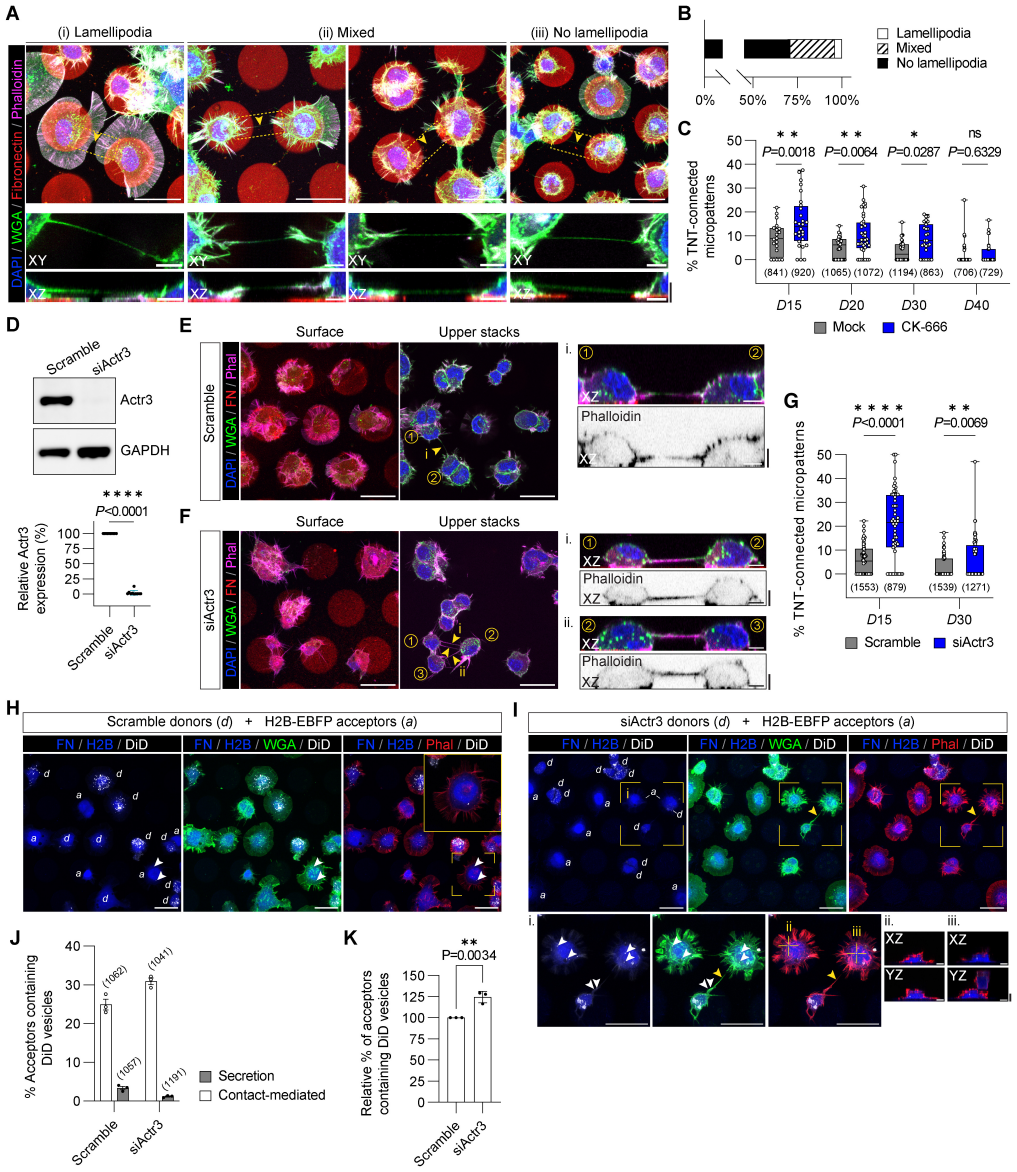
TNT prevalence negatively correlates with Arp2/3 activity TNT‐connected cells characteristically lack lamellipodial features. Representative maximum intensity projection images of cells with different morphologies that are connected by TNTs; Scale bars, 30 μm. TNTs can be formed between: (i) two cells forming only lamellipodia, (ii) two cells showing a mix of phenotypes (e.g., one of the connected cells displays only (or partial) lamellipodia while the other connected cell shows no lamellipodia), or (iii) two non‐lamellipodia cells displaying a higher density of peripheral protrusions. Dashed yellow boxes correspond to subpanels below each image that show expanded views and XZ projections of TNTs that were made through the long axis of the connection; Scale bars, 5 μm. Yellow arrowheads directly point to the TNT.Bar graph showing the characterization of TNTs between cells of different morphologies plated on *D*15 micropatterns (*n* = 97 TNTs). Lamellipodia, 4.1%; Mixed, 24.7%; and No Lamellipodia, 71.1%.Whisker box plot showing the percentage of TNT‐connected micropatterns for *D*15, *D*20, *D*30 and *D*40 micropatterns in cells treated with 50 μM CK‐666 as compared to the mock condition (DMSO vehicle control). Mock vs. CK‐666 average values were 8.8 ± 1.5% vs. 15.5 ± 2.0% for *D*15 micropatterns, 5.0 ± 0.8% vs. 10.4 ± 1.3% for *D2*0 micropatterns, 3.6 ± 0.7% vs. 8.5 ± 1.3% for *D*30 micropatterns, and 2.0 ± 0.9% vs. 2.3 ± 0.8% for *D*40 micropatterns (mean ± SEM). Each data point corresponds to a quantified image in which on average approximately 30 cell‐occupied micropatterns were within the acquired field of view. Representative images of the different conditions can be found in Appendix Fig S4. The total number of individual micropatterns quantified in each condition is indicated below each box. Data was pooled from 3 experiments and was analysed using an unpaired Mann–Whitney test.siRNA knockdown of Actr3 (Arp3) in CAD cells. Top: Representative Western blot of Scramble control cells and siActr3 cells revealed with α‐Actr3 and α‐GAPDH (loading control) antibodies. Bottom: Graph showing the relative expression of Actr3 in Scramble control cells (set to 100%) and siActr3 cells (1.9 ± 1.2%, mean ± SEM). Statistical analysis was performed using an unpaired Student's *t*‐test (*n* = 10 biological repeats of the knockdown).Representative surface and upper stack maximum intensity projections of Scramble control CAD cells plated on *D*15 micropattern; Scale bars, 30 μm. Subpanels in i show XZ projections made through the long axis of the indicated TNT (yellow arrowhead); Scale bars, 5 μm. Cells were fixed and stained with DAPI (blue), AX‐488 WGA (green), and AX‐647 Phalloidin (magenta); micropatterns were visualized using Rhodamine FN (red).Representative surface and upper stack maximum intensity projections of siActr3 treated CAD cells plated on *D*15 micropatterns; Scale bars, 30 μm. Subpanels (i, ii) show XZ projections made through the long axis of the indicated TNTs (yellow arrowheads); Scale bars, 5 μm. Cells were fixed and stained with DAPI (blue), AX‐488 WGA (green) and AX‐647 Phalloidin (magenta); micropatterns were visualized using Rhodamine‐FN (red).Whisker box plot showing the percentage of TNT‐connected micropatterns for Scramble control and siActr3 CAD cells plated on *D*15 and *D*30 micropatterns. Scramble vs. siActr3 average values were 5.9 ± 0.7% vs. 20.5 ± 1.9% for *D*15 micropatterns and 2.6 ± 0.6% vs. 6.3 ± 1.1% for *D*30 micropatterns (mean ± SEM). Each data point corresponds to a quantified image in which on average approximately 20 cell‐occupied micropatterns were within the acquired field of view. Representative images of the different conditions can be found in Appendix Fig S5. The total number of individual micropatterns quantified in each condition is indicated below each box. Data for *D*15 and *D*30 was pooled from 4 and 3 experiments, respectively, and was analysed using an unpaired Mann–Whitney test.Representative images of Scramble‐treated donor cells (*d*) co‐cultured with H2B‐EBFP expressing acceptor cells (*a*) on *D*15 micropatterns; Scale bars, 30 μm. White arrowheads annotate donor‐originating DiD‐labelled vesicles (white) in an acceptor cell. The yellow box indicates the enlarged area shown in the upper right inset. Cells were fixed and stained with AX‐488 WGA (green) and Rhodamine Phalloidin (red); micropatterns were visualized using AX‐405 FN (blue).Representative images of siActr3‐treated donor cells (*d*) co‐cultured with H2B‐EBFP expressing acceptor cells (*a*) on *D*15 micropatterns; Scale bars, 30 μm. Subpanels in i show an enlargement of the enclosed area noted by the yellow box in the main micrographs; Scale bars, 30 μm. White arrowheads annotate donor‐originating DiD‐labelled vesicles (white) in an acceptor cell; yellow arrowheads annotate TNTs connecting cells on neighbouring micropatterns. Indicated crosshairs (ii, iii) correspond to XZ and YZ orthogonal projections of internalized vesicles in the subpanels; Scale bars, 5 μm. Cells were fixed and stained with AX‐488 WGA (green) and Rhodamine Phalloidin (red); micropatterns were visualized using AX‐405 FN (blue).Bar graph showing the percentage of H2B‐EBFP acceptors containing DiD‐labelled vesicles received through cell–cell contact (i.e., corrected for secretion‐based transfer) from Scramble‐ (24.9 ± 1.4%) and siActr3‐treated (30.9 ± 0.9%) donors. Corresponding secretion‐based transfer levels used for determining contact‐mediated transfer levels are shown for comparison and were 3.3 ± 0.4% and 1.1 ± 0.1% for Scramble and siActr3, respectively. The total number of quantified acceptor cells is indicated for each condition. Data are from three individual experiments and are represented as a mean ± SEM.Bar graph showing the relative percentage of H2B‐EBFP acceptors containing DiD‐labelled vesicles from siActr3‐treated donors (124.5 ± 3.9%, mean ± SEM) as compared to Scramble control donors (set to 100%). Data are from three individual experiments and were analysed using an unpaired Student's *t*‐test. Data information: Whisker box plots in (C) and (G) display the median values as internal central lines while the box edges mark the lower and upper quartiles; whiskers mark the min and max of a data set. Source data are available online for this figure.

Actin assembly can be organized into distinct architectures such as Arp2/3‐dependent branched filaments in lamellipodia, or bundles of un‐branched linear filaments. Phenotypic characterization of TNT‐connected cells on *D*15 micropatterns revealed that cells displaying lamellipodia accounted for 4.1% of the examined cells with TNTs (Fig 2A and B). In contrast, in 71.1% of cases both cells involved in the connection had no lamellipodia (Fig 2A and B) while 24.7% of these TNT‐connected cells had a mix of cellular phenotypes (Fig 2A and B). Further analysis revealed that TNTs rarely emerge directly from lamellipodia (< 2% of cases) and instead originate either further up on the cell body or from protrusion‐rich zones (Appendix Fig S3). Together, this suggests an anticorrelation between regions with high Arp2/3 activity and a cell's ability to form TNTs.

To directly test this hypothesis, we next assessed if the inhibition of actin incorporation in Arp2/3‐dependent networks favours the formation of TNTs. First using a pharmacological approach to inhibit Arp2/3 activity with the specific inhibitor CK‐666 (Hetrick *et al*, 2013; Sartori‐Rupp *et al*, 2019), we observed a significant increase in the percent of TNT‐connected cells on *D*15, *D*20 and *D*30 micropatterns (Fig 2C and Appendix Fig S4). In contrast, CK‐666 had no impact on the number of TNT‐connected cells on *D*40 patterns (Fig 2C and Appendix Fig S4) supporting a distance dependency for TNT formation in which an upper limit to the length a TNT can obtain through the polymerization of actin exists (as earlier assessed in Fig 1F).

To further substantiate the negative role that Arp2/3 activity has on TNT formation, we then implemented a knockdown (KD) approach using siRNA to interfere with the expression of the Arp2/3 complex protein Actr3, i.e., Arp3, (an average decrease of 98% compared to Scramble control) in CAD cells (Fig 2D). Quantification of the percent of TNT‐connected cells on both *D*15 and *D*30 micropatterns showed a significant increase in TNT numbers for Actr3 KD cells as compared to control cells treated with nontargeting “Scramble” siRNA duplexes (Fig 2E−G, and Appendix Fig S5) and agreed with our previous CK‐666 results. To examine whether the increase in TNTs upon Actr3 KD is correlated with an increase in functional TNTs capable of cargo transfer, we then performed a vesicle transfer assay (Appendix Fig S2B) in which Scramble‐ and siActr3‐treated donor cells containing DiD‐labelled vesicles were co‐cultured with H2B‐EBFP expressing acceptor cells (Fig 2H and I). In order to rule out the contribution of vesicle transfer by secretion and determine the amount of vesicles transferred via cell–cell contact, samples were prepared in parallel in which conditioned media from Scramble and siActr3 donor cells was applied to acceptor cells. While in both control and Actr3 KD conditions the transfer mediated through the secretion was negligible, after 16–18 h of co‐culture, Actr3 KD co‐cultures showed an increased contact‐mediated transfer of vesicles (Fig 2J) that was approximately 25% higher when compared to Scramble controls (Fig 2K). This suggests that a reduction in active Arp2/3 complexes enhances the transfer of vesicles via an increased number of TNT connections between patterned cells.

We hypothesized that a competition between different actin networks has a direct effect on the likelihood for a TNT to form over a given distance. Thus we directly shifted the actin balance in favour of linear F‐actin by using the formin‐specific agonist drug IMM‐01 (Lash *et al*, 2013) and assessed the effect on TNTs. With IMM‐01 treatment, we observed an increased number of TNT‐connected cells (Appendix Fig S6A and B) and of vesicles transferred from donor to acceptor cells (Appendix Fig S6C), indicating that the promotion of linear actin networks directly favours functional TNT formation. Together this data suggests that TNT formation is based on the polymerization of long, linear filaments and becomes enhanced when branched actin formation is inhibited.

### F‐actin polymerization occurs over longer distances when Arp2/3 is inhibited

**Figure 3:**
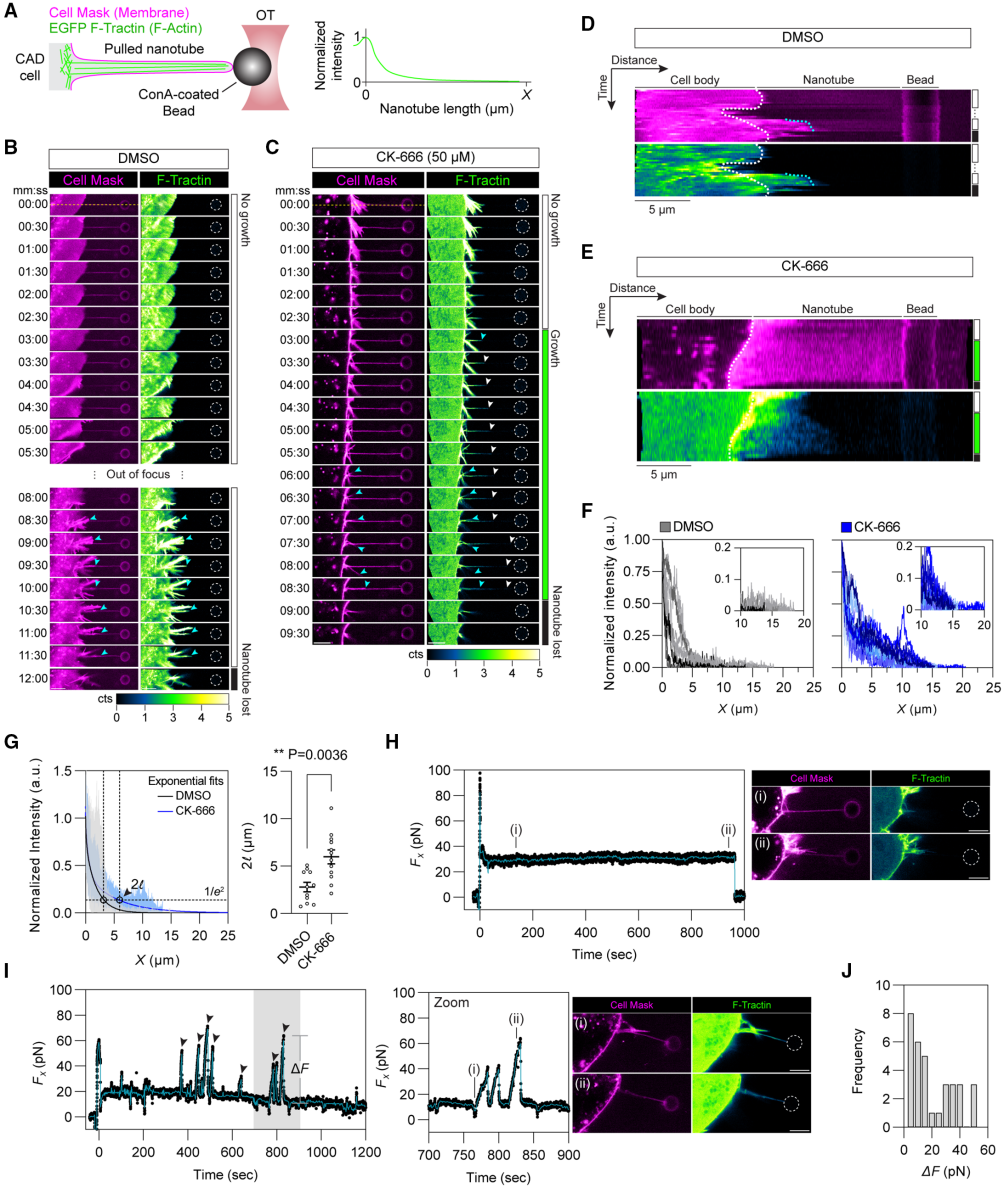
Inhibition of branched actin formation results in greater F‐actin development in pulled nanotubes AExperimental setup for pulling membrane nanotubes using a concanavalin A (Con‐A)‐coated bead (~3 μm in diameter) trapped in an optical tweezer (OT). CAD cells stably expressing the small F‐actin‐binding peptide F‐Tractin fused to EGFP (green) were exogenously labelled with the lipophilic Cell Mask™ Deep Red plasma membrane stain (magenta). Intensity profiles of the F‐actin fluorescence were measured along the nanotube axis and plotted against the nanotube length. Pulled nanotube lengths averaged 14.2 ± 0.6 μm (mean ± SEM, *n* = 23 tubes).B, CTime‐lapse montages of nanotubes pulled from a DMSO‐treated cell (Movie EV7) and a CK‐666‐treated cell (Movie EV8). Displayed intensities for the F‐Tractin channel were set between 0 and 5 photon counts (cts) for better visualization. White arrowheads annotate the progression of actin development within the nanotube, while cyan arrowheads annotate obstructing protrusions that grew along the pulled nanotube. The trapped bead is annotated with a dotted white circle when not clearly visible. Scale bars, 5 μm.D, ECorresponding kymographs for the time‐lapse montages in (B) and (C), generated along the nanotube axis (yellow dotted lines in B and C). Dotted white and cyan lines in the kymographs mark the cell body edge and obstructing protrusions, respectively. Scale bars, 5 μm.FPlot of actin profiles within pulled nanotubes for mock‐ and CK‐666‐treated cells. Insets show a magnified view at the tube extremity to better highlight the greater presence of F‐actin in the CK‐666 condition as compared to the mock condition. Mock condition, 11 tubes; CK‐666 condition, 12 tubes.GLeft: Exponential fits to the actin intensity profiles were performed to determine a characteristic decay length (2ℓ) at which the initial intensity at *X* = 0 decays to a value of 1/*e* ^2^. For visualization purposes of the analysis, exponential fits are shown for the mean actin profiles computed from the individual plots presented in (F). Upper and lower limits of the intensity range are shaded. Right: Dot plot of the characteristic decay lengths (2ℓ) for mock‐ and CK‐666‐treated cells. Data is represented as the mean ± SEM. Mock (11 tubes), 2.78 ± 0.50; CK‐666 (12 tubes), 5.80 ± 0.73. Statistical analysis was performed using an unpaired Mann–Whitney test.HLeft: Force plot of a pulled nanotube from a mock‐treated cell showing no F‐actin development. Solid teal line, 10‐point moving average curve. Right: Associated images of the indicated time points (i, ii). The trapped bead is annotated with a dotted white circle when not clearly visible. Scale bars, 5 μm.ILeft: Force plot of a pulled nanotube from a CK‐666‐treated cell showing F‐actin development spanning the entire nanotube length. Peaks in the force plot (black arrowheads), with magnitudes of Δ*F*, arise when retrograde flows outcompete actin polymerization (at the nanotube tip) causing bead displacement towards the cell body (recorded as a positive rise in the force in the lab frame). Solid teal line, 10‐point moving average curve. Shaded grey region corresponds to a magnified view in the centre. Right: Associated images of the indicated time points (i, ii). The trapped bead is annotated with a dotted white circle when not clearly visible. Scale bars, 5 μm.JHistogram of the force peak magnitudes (Δ*F*). Sample size, 33 peaks. Source data are available online for this figure.

**Figure EV2:**
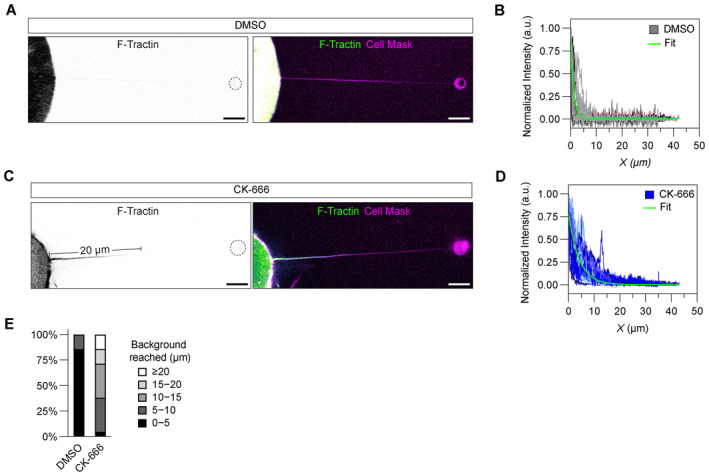
Inhibition of Arp2/3 activity increases the frequency of actin polymerization occurring at extreme distances Representative nanotube pulled to an extreme length from a DMSO‐treated CAD cell showing little to no actin polymerization within. Displayed F‐Tractin intensities were set between 0 and 10 photon counts for better visualization. The trapped bead is annotated with a dotted white circle when not clearly visible. Scale bars, 5 μm.Plot of actin profiles within pulled nanotubes of extreme lengths for DMSO‐treated CAD cells (*n* = 21 tubes). The solid green line is an exponential fit to the combined data.Representative nanotube pulled to an extreme length from a CK‐666‐treated (50 μM) CAD cell showing actin growth 20 μm within the nanotube. Displayed F‐Tractin intensities were set between 0 and 10 photon counts for better visualization. The trapped bead is annotated with a dotted white circle when not clearly visible. Scale bars, 5 μm.Plot of actin profiles within pulled nanotubes of extreme lengths for CK‐666‐treated CAD cells (*n* = 21 tubes). The solid green line is an exponential fit to the combined data.Categorization of the percentage of pulled nanotubes with F‐actin profiles becoming indistinguishable from background intensity levels within a given micron range for DMSO‐ and CK‐666‐treated CAD cells.

To test our hypothesis and gain a greater insight into how TNTs can reach longer distances through actin polymerization, we utilized an optical tweezer (OT) setup to pull nanotubes of comparable lengths to the TNTs observed on the micropatterns. Given these pulled nanotubes are initially devoid of any actin, filament growth within the nanotube can be monitored by confocal microscopy (Fig 3A) (Pontes *et al*, 2011; Bornschlögl *et al*, 2013; Datar *et al*, 2015; Tsai *et al*, 2022). While the measured length of any cellular protrusion is a direct readout of the overall actin filament length within, our assay instead advantageously permits us to ascertain the most probable length scales actin reaches in a given condition as filament growth can occur in a situation unencumbered by restraining factors, such as the tension of the membrane, that “real” protrusions must work against to outwardly grow. Thus, our assay permits a controlled and high‐throughput means for assessing the true extent to which Arp2/3 inhibition may favour longer filament formation necessary for achieving the lengths exhibited by TNTs. In mock, DMSO‐treated conditions, cells displayed little to no F‐actin growth within the nanotubes (Fig 3B and Movie EV7) while CK‐666‐treated cells showed instead a robust development of F‐actin (Fig 3C and Movie EV8) that emanated from the base of the nanotube and grew towards the nanotube end (see comparative kymograph analysis in Fig 3D and E). Measurement of the F‐actin fluorescence profiles showed a rapid decay of the signal in mock‐treated cells (Fig 3F) where intensity values were indistinguishable from background levels beyond 5 μm, a length scale consistent with the average length measured previously for CAD cell filopodia (Fig 1G). However, CK‐666‐treated cells exhibited a shallower decay in the fluorescence profile and intensities remained higher throughout the entire nanotube (Fig 3F). Exponential fitting of the actin profiles (Fig 3G) and force measurements (Fig 3H and I) confirmed CK‐666‐treated cells had greater F‐actin levels reaching significantly longer distances within the nanotube compared to mock conditions. For example, force profiles remained static over time in control conditions for actin‐devoid nanotubes (Fig 3H), whereas large bead displacements (i.e., a rise in the force, Δ*F*) were observed in the CK‐666 condition (Fig 3I), indicating that F‐actin development had reached the end of the tube, imparting large traction forces (Fig 3J). This characteristic actin‐dependent behaviour has been described previously by us and others (Bornschlögl *et al*, 2013; Datar *et al*, 2015; Leijnse *et al*, 2022; Tsai *et al*, 2022). These data indicate that an inhibition of Arp2/3 activity directly correlates with a greater occurrence of actin filaments able to polymerize and reach longer distances. To further substantiate these results, we pulled nanotubes at more extreme lengths averaging 33.6 ± 1.2 μm (mean ± SEM, 42 tubes) (Fig EV2) to assess the most probable lengths that F‐actin is able to achieve upon Arp2/3 inhibition. As before, we found that cells in the mock, control condition exhibited little infiltration of F‐actin growth within the nanotube (Fig EV2A and B); F‐actin profiles typically decayed to intensity values indistinguishable from background levels within 5 μm inside the nanotube and did not extend beyond 10 μm (Fig EV2E). Contrastingly, Arp2/3 inhibition led to far more F‐actin growth (Fig EV2C and D) with a noticeable increase in the percentage of pulled tubes exhibiting F‐actin infiltration beyond 10 μm with even events of F‐actin growth reaching distances upwards of 20 μm (Fig EV2E). These data indicate that an inhibition of Arp2/3 activity directly correlates with the polymerization of longer actin filaments, permitting TNTs to more frequently reach extreme distances (i.e., their distance dependency is shifted to longer length scales).

### An Eps8‐IRSp53 interaction is enhanced upon Arp2/3 inhibition

**Figure 4:**
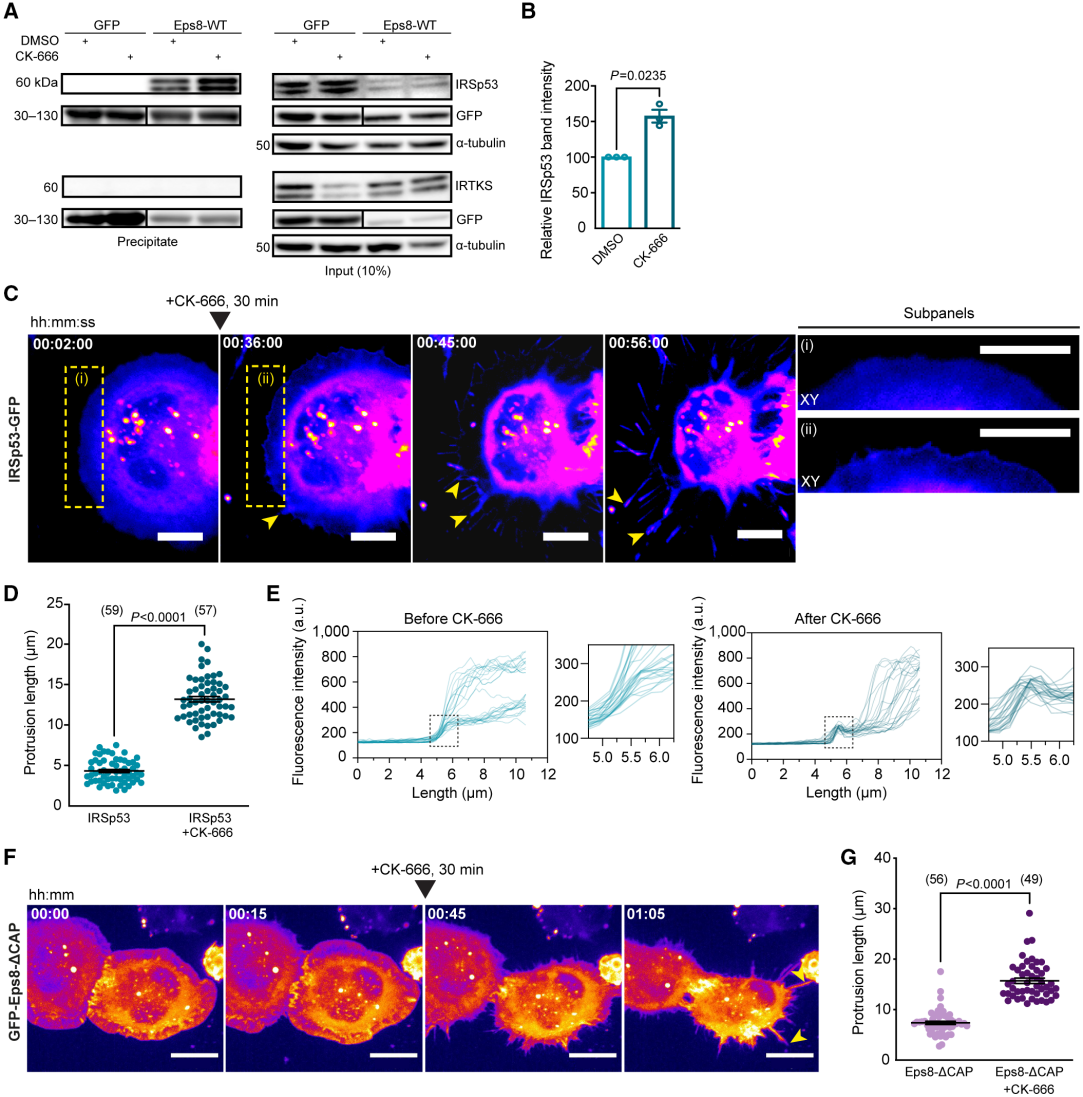
Eps8 and IRSp53 are recruited to form longer protrusions upon Arp2/3 inhibition Representative Western Blots showing GFP‐trap immunoprecipitation bands of the IRSp53 and IRTKS prey proteins in the GFP‐Eps8‐WT bait precipitate (left) and input lysate (right), with or without 50 μM CK‐666 treatment.Relative IRSp53 band intensity quantification. Both the CK‐666‐treated and DMSO‐treated bands for IRSp53 were normalized with respect to their Eps8‐WT bait bands (detected by an anti‐GFP antibody). Relative band intensity was 157.5 ± 8.9% for the CK‐666 condition as compared to the DMSO control (was set to 100%). Data are from three individual experiments and are represented as a mean ± SEM. Statistical analysis was performed using a *t*‐test with Welch's correction, *P* = 0.0235.Representative time‐lapse images (2, 36, 45, and 56 min from Movie EV9) of protrusion formation in IRSp53‐transfected cells before and after CK‐666 addition (at 30 min). Yellow arrowheads show protrusions that are formed after CK‐666 addition. Subpanels (i, ii) show the enlarged sections of the cell edge before (i) and after (ii) the addition of CK‐666, demonstrating the recruitment of IRSp53 at the membrane (ii). Scale bars, 10 μm.Scatter plot comparing the maximum protrusion length before (4.3 ± 0.2 μm) and after CK‐666 addition (13.2 ± 0.3 μm) in IRSp53‐transfected cells containing the F‐actin label tdTomato‐F‐Tractin. Twenty‐five cells were analysed, and the number of protrusions analysed in each condition is indicated in parentheses. Data are from 10 individual experiments and are represented as a mean ± SEM. Statistical analysis was performed using an unpaired *t*‐test with Welch's correction, *P* < 0.0001.Profile plots of IRSp53 intensity determined across the membrane before (left) and after the addition of CK‐666 (right). Magnified views of the cell edge (dashed black boxes) are provided to better highlight the recruitment of IRSp53 at the cell membrane in the CK‐666 condition as compared to the control. Two cells were analysed: before CK‐666, 28 measurements; after CK‐666, 25 measurements.Representative time‐lapse images (0, 15, 45 and 65 min from Movie EV10) of protrusion formation in Eps8‐ΔCAP‐transfected cells before and after CK‐666 addition (at 30 min). Yellow arrowheads show protrusions that are formed after CK‐666 addition. Scale bars, 20 μm.Scatter plot comparing the maximum protrusion length before (7.4 ± 0.3 μm) and after CK‐666 addition (15.7 ± 0.5 μm) in Eps8‐ΔCAP‐transfected cells containing the F‐actin label tdTomato‐F‐Tractin. Twenty‐one cells were analysed, and the number of protrusions analysed in each condition is indicated in parentheses. Data are from nine individual experiments and are represented as a mean ± SEM. Statistical analysis was performed using unpaired *t*‐test with Welch's correction and *P* value <0.0001. Source data are available online for this figure.

**Figure EV3:**
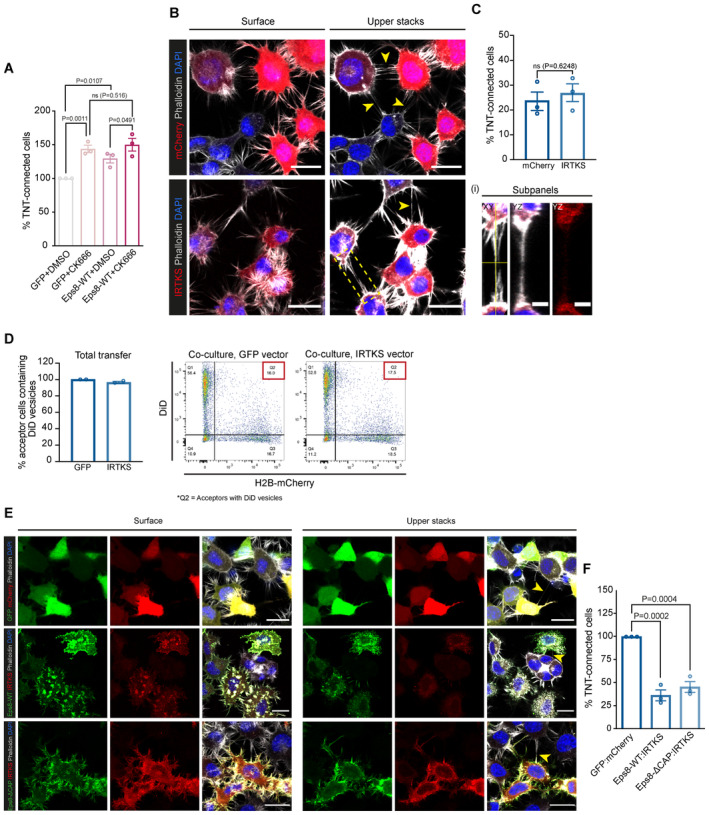
IRTKS does not promote TNT formation, but rather decreases it when co‐expressed with Eps8 Bar graph showing the quantification of TNT‐connected cells prior to immunoprecipitation for GFP + DMSO (297 cells analysed; 100%), GFP + CK‐666 (229 cells analysed; 144.0 ± 5.4%), GFP‐Eps8‐WT + DMSO (224 cells analysed; 129.4 ± 6.2%) and GFP‐Eps8‐WT + CK‐666 cells (246 cells analysed; 150.0 ± 9.5%). Data are from three individual experiments and are represented as a mean ± SEM. Statistical analysis was performed using an ordinary ANOVA with Tukey's multiple comparison test. *P* values for each comparison are stated on the bar graph.Representative images of surface and upper stacks of mCherry‐transfected control cells and IRTKS‐mCherry transfected cells plated on nonpatterned surfaces. Yellow arrowheads annotate TNT‐like protrusions. Subpanels (i) show the XY and YZ projections through the axis of the TNT indicated in the dashed yellow box.Bar graph showing quantification of TNT‐connected cells in mCherry control (309 cells analysed; 23.6 ± 4%) and in IRTKS‐mCherry cells (268 cells analysed; 26.5 ± 3.9%). Data are from three individual experiments and are represented as a mean ± SEM. Statistical analysis was performed using a *t*‐test with Welch's correction, *P* = 0.6248.Left: Bar graph showing total transfer analysis in GFP control co‐culture (100%) and IRTKS‐GFP co‐culture (96.5 ± 1.3%). Data are from two individual experiments and are represented as a mean ± SEM. Right: Gating strategy for flow cytometry measurements of total transfer. Q2 represents H2B‐mCherry‐labelled acceptor cells containing donor‐derived DiD vesicles.Left: Representative surface images of GFP:mCherry (control), GFP‐Eps8‐WT:IRTKS‐mCherry and GFP‐Eps8‐ΔCAP:IRTKS‐mCherry co‐transfected cells. Right: Representative images of upper stacks of GFP:mCherry, GFP‐Eps8‐WT:IRTKS‐mCherry and GFP‐Eps8‐ΔCAP:IRTKS‐mCherry co‐transfected cells. Yellow arrowheads annotate TNT‐like protrusions. Cells were plated on nonpatterned surfaces.Bar graph showing the quantification of TNT‐connected cells in GFP:mCherry (453 cells analysed; 100%), Eps8‐WT:IRTKS (203 cells analysed; 36.0 ± 5.9%) and in Eps8‐ΔCAP:IRTKS (252 cells analysed; 45.1 ± 5.9%) co‐transfected control cells. Data are from three individual experiments and are represented as a mean ± SEM. Statistical analysis was performed using an ordinary ANOVA with Dunnett's multiple comparison test. *P* values for each comparison are stated on the bar graph. Data information: In (B) and (E), large panel images have scale bars representing 20 μm, while the subpanel images have scale bars representing 5 μm.

A shift in actin utilization upon Arp2/3 inhibition likely promotes direct protein–protein interactions that outwardly deform the PM and polymerize F‐actin locally for TNT outgrowth. Given Eps8's previously identified TNT‐promoting capability (Delage *et al*, 2016), we investigated whether a specific Eps8‐I‐BAR interaction favours long TNT formation. Such an interaction would couple Eps8's ability to bundle F‐actin necessary to overcome the tension imposed by the membrane, and an I‐BAR's ability to induce membrane deformation through the protein's intrinsic curvature. We first screened Eps8's interaction with IRTKS (insulin receptor tyrosine kinase substrate) and IRSp53 based on their I‐BAR domain sequence similarity (Saarikangas *et al*, 2009) and their involvement in the formation of filopodia and microvilli (Disanza *et al*, 2013; Garbett *et al*, 2013; Sudhaharan *et al*, 2016; Postema *et al*, 2018). GFP‐Eps8‐wild type (WT) was used to immunoprecipitate endogenous IRTKS and/or IRSp53 in control and TNT‐promoting conditions using CK‐666 (Fig EV3A). IRSp53 precipitated with GFP‐Eps8‐WT in control conditions, indicating their direct interaction in CAD cells, while IRTKS did not (Fig 4A). Furthermore, immunofluorescence showed both endogenous Eps8 and IRSp53 were detected in TNT‐like structures (Appendix Fig S7). To see if the interaction is affected by Arp2/3 inhibition, we treated GFP‐Eps8‐WT cells with CK‐666. A significant increase in eluted IRSp53 was observed, whereas no effect was observed for IRTKS (Fig 4A and B). These data indicated that interaction between Eps8 and IRSp53, but not IRTKS, would favour long TNT growth.

Using spinning disk microscopy, we then characterized the process of IRSp53‐ and Eps8‐driven protrusions and analysed the maximum length of cell‐generated protrusions before and after CK‐666 addition (Fig 4C–G). In these experiments we utilized Eps8 in its bundling active form (capping defunct mutant; Eps8‐ΔCAP) to directly observe the impact of this function on protrusion formation without secondary effects caused by its capping function. Prior to CK‐666 treatment (first 30 min), IRSp53‐ and Eps8‐ΔCAP‐expressing cells were found to form lamellipodia devoid of protrusions (Fig 4C and F, and Movies EV9 and EV10) or shorter filopodia‐like protrusions (Appendix Fig S8A and B, and Movies [Link], [Link]). After CK‐666 addition, new IRSp53‐ and Eps8‐positive protrusions grew two to three times longer than those found in the nontreated condition (Fig 4D and G). Furthermore, following CK‐666 addition, we observed an increase of IRSp53 fluorescence at the plasma membrane (Fig 4E and Movie EV9) which then coincided with the generation and growth of IRSp53‐positive dorsal protrusions, indicating a greater availability of IRSp53 to generate protrusions when branched actin pathways are inactivated.

### Eps8 and IRSp53 co‐expression increases the formation of functional TNTs

**Figure 5:**
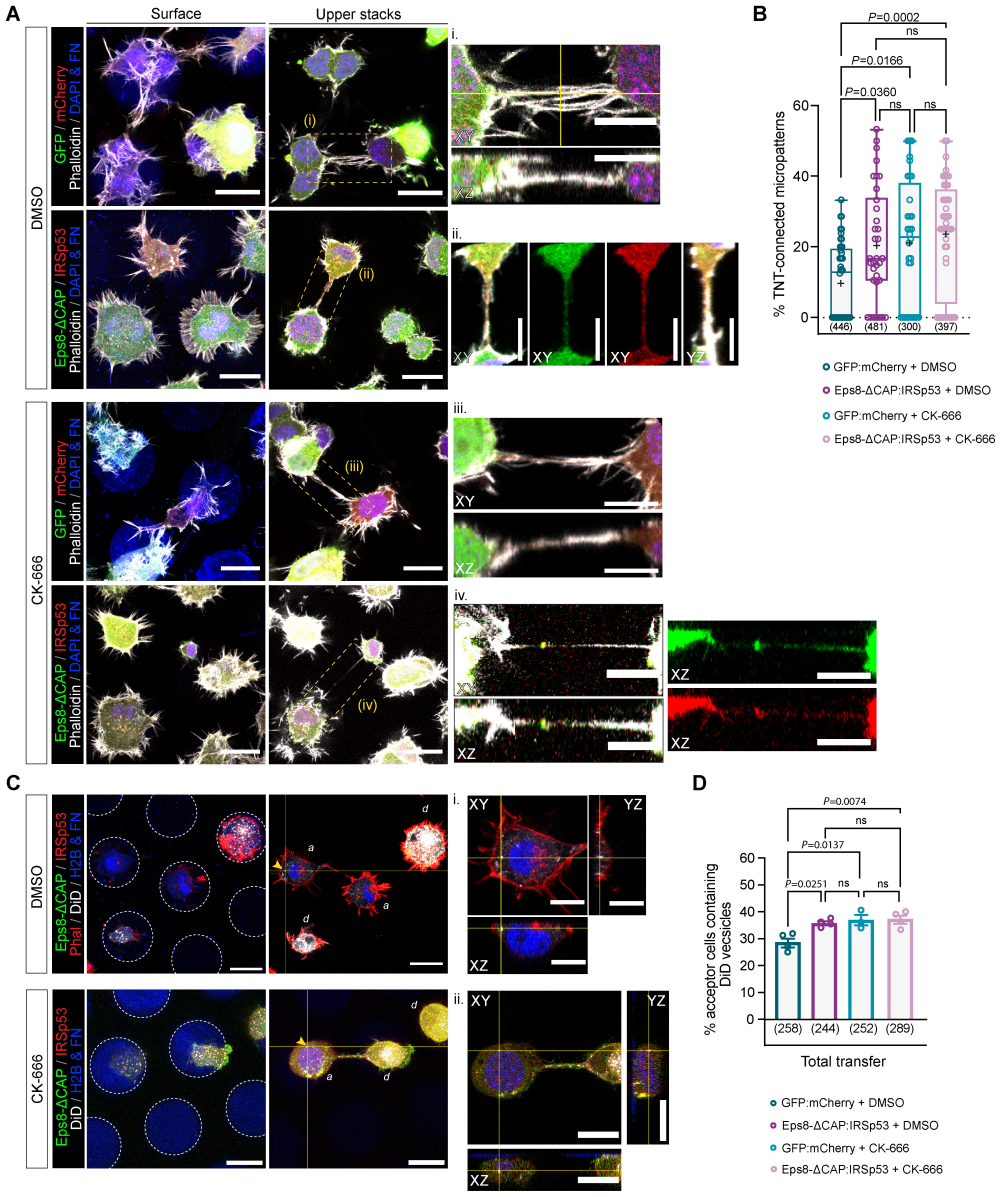
Eps8–IRSp53 co‐expression leads to the formation of functional TNT‐like protrusions on micropatterns Representative surface and upper stack images of control GFP:mCherry and GFP‐Eps8‐ΔCAP:IRSp53‐mCherry expressing CAD cells plated on *D*15 micropatterns (Alexa 405‐labelled FN) treated with either DMSO or CK‐666. Subpanels (i–iv) show magnified projections of the TNTs indicated in the dashed yellow boxes; XZ and YZ projections were made through the axis of the TNT.Whisker box plot of the percentage of TNT‐connected cells on *D*15 micropatterns for GFP:mCherry + DMSO (10.3 ± 1.6%), Eps8‐ΔCAP:IRSp53 + DMSO (20.8 ± 2.8%), GFP:mCherry + CK‐666 (21.4 ± 3.1%), and Eps8‐ΔCAP:IRSp53 + CK‐666 cells (23.5 ± 2.4%) (mean ± SEM). Total number of quantified cells on patterns is indicated below the Whisker plots for each condition. Data were pooled from three individual experiments and each data point corresponds to a quantified image in which on average approximately 10 cell‐occupied micropatterns were within the acquired field of view. Mean values are indicated as a + symbol on the graph for each condition. Statistical analysis was performed using a Kruskal Wallis test with Dunn's multiple comparison test. Significant *P* values for each comparison are stated on the plot, ns = nonsignificant for *P* > 0.9999.Representative images of GFP‐Eps8‐ΔCAP:IRSp53‐mCherry donor cells (*d*) co‐cultured with H2B‐EBFP acceptors (*a*) treated with either DMSO or CK‐666. Dashed yellow circles annotate Alexa 405‐labelled FN patterns for clarity and yellow arrowheads show the donor‐originating vesicle in the acceptor cell. Subpanels (i, ii) show magnified projections of the acceptor cell containing DiD‐labelled vesicles (yellow arrowheads); XZ and YZ projections were made through the vesicle.Bar graph showing the percentage of H2B‐EBFP acceptors containing DiD‐stained vesicles in GFP:mCherry + DMSO (28.8 ± 1.7%), Eps8‐ΔCAP:IRSp53 + DMSO (35.7 ± 0.7%), GFP:mCherry + CK‐666 (37.0 ± 1.9%), and Eps8‐ΔCAP:IRSp53 + CK‐666 (37.2 ± 1.5%) experiments. Total number of quantified acceptor cells is indicated for each condition. Data are from at least three individual experiments and are represented as a mean ± SEM. Statistical analysis was performed using an ordinary ANOVA with Tukey's multiple comparison test. Significant *P* values for each comparison are stated on the bar graph. ns = nonsignificant for GFP:mCherry + CK‐666 vs. Eps8‐ΔCAP:IRSp53 + DMSO, *P* = 0.9246; for GFP:mCherry + CK‐666 vs. Eps8‐ΔCAP:IRSp53 + CK‐666, *P* = 0.9999; and for Eps8‐ΔCAP:IRSp53 + DMSO vs. Eps8‐ΔCAP:IRSp53 + CK‐666, *P* = 0.8807. Data information: In (A) and (C), large panel images have scale bars representing 20 μm, while the subpanel images have scale bars representing 10 μm. Source data are available online for this figure.

**Figure 6:**
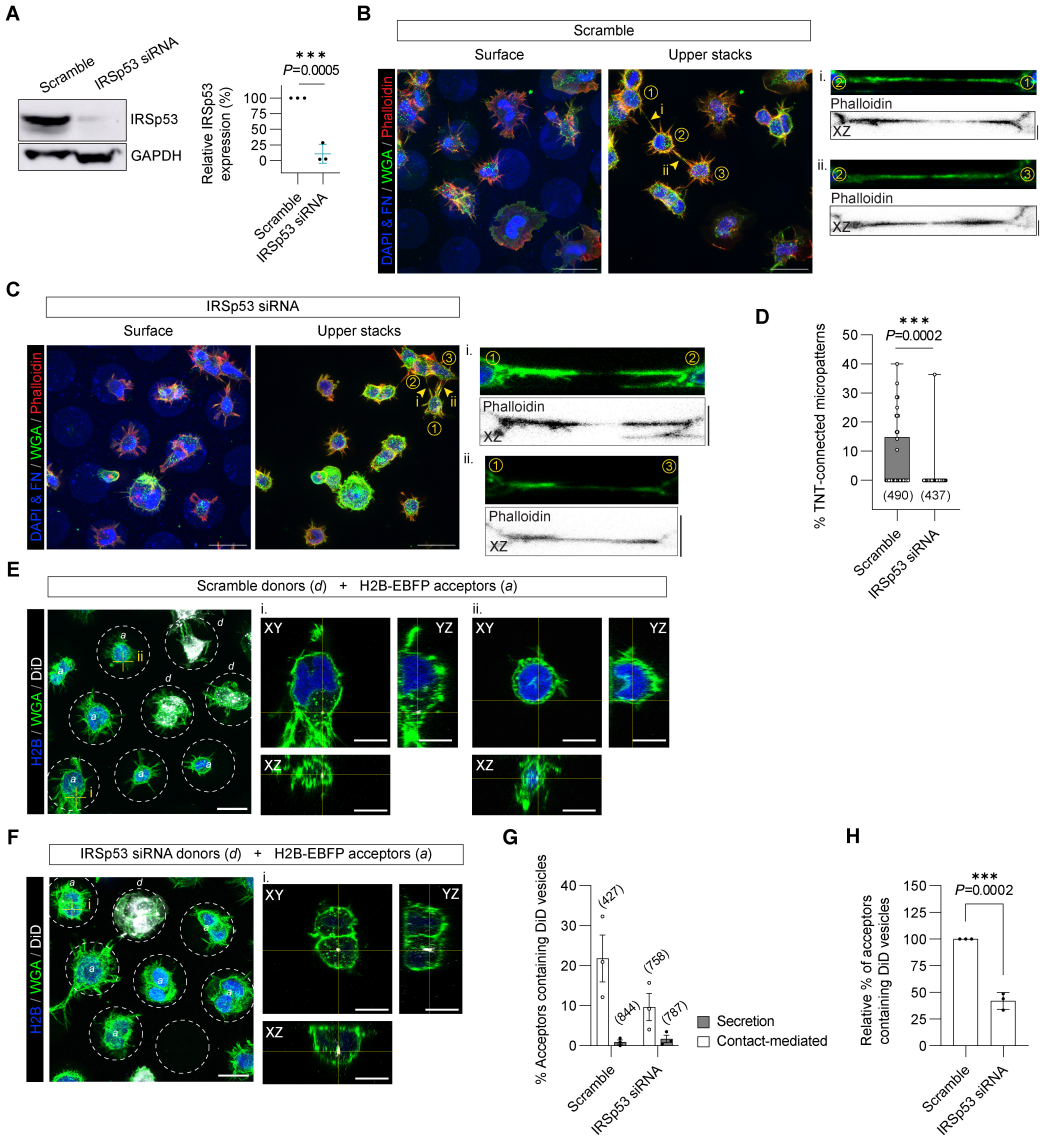
IRSp53 knockdown leads to a decrease in functional TNTs siRNA knockdown of IRsp53 in CAD cells. Left: Representative Western blot of Scramble control cells and IRSp53 siRNA cells revealed with α‐IRSp53 and α‐GAPDH (loading control) antibodies. Right: Graph showing the relative expression of IRSp53 in Scramble control cells (set to 100%) and IRSp53 siRNA cells (10.8 ± 8.7%, mean ± SEM). Statistical analysis was performed using an unpaired Student's *t*‐test (*n* = 3 biological repeats of the knockdown).Representative surface and upper stack images of control CAD cells plated on *D*15 micropatterns treated with Scramble siRNA; Scale bars, 30 μm. Subpanels (i, ii) show XZ projections made through the long axis of the indicated TNTs (yellow arrowheads); Scale bars, 5 μm. Cells were fixed and stained with DAPI (blue), AX‐488 WGA (green) and Rhodamine Phalloidin (red); micropatterns were visualized using AX‐405 FN (blue).Representative surface and upper stack images of IRSp53 knock‐down CAD cells plated on *D*15 micropatterns; Scale bars, 30 μm. Subpanels (i, ii) show XZ projections made through the long axis of the indicated TNTs (yellow arrowheads); Scale bars, 10 μm. Cells were fixed and stained with DAPI (blue), AX‐488 WGA (green) and Rhodamine Phalloidin (red); micropatterns were visualized using AX‐405 FN (blue).Whisker box plot showing the percentage of TNT‐connected micropatterns for Scramble control and IRSp53 siRNA CAD cells plated on *D*15 micropatterns. Scramble vs. IRSp53 siRNA average values were 6.8 ± 0.8% vs. 0.7 ± 0.7% (mean ± SEM). Each data point corresponds to a quantified image in which on average approximately 10 cell‐occupied micropatterns were within the acquired field of view. The total number of individual micropatterns quantified in each condition is indicated below each box. Data was pooled from three experiments and was analysed using an unpaired Mann–Whitney test.Representative images of Scramble‐treated donor cells (*d*) co‐cultured with H2B‐EBFP (blue) expressing acceptor cells (*a*) on *D*15 micropatterns; Scale bars, 20 μm. Indicated crosshairs (i, ii) correspond to XZ and YZ orthogonal projections of DiD‐labelled vesicles (white) internalized within acceptor cells (see subpanels); Scale bars, 10 μm. Cells were fixed and stained with AX‐488 WGA (green); micropatterns were unstained but visible upon over‐saturation in the green channel and outlined with dotted white circles for clarity.Representative images of IRSp53 siRNA‐treated donor cells (*d*) co‐cultured with H2B‐EBFP (blue) expressing acceptor cells (*a*) on *D*15 micropatterns; Scale bars, 20 μm. The indicated crosshair (i) corresponds to XZ and YZ orthogonal projections of an example DiD‐labelled vesicle (white) internalized within an acceptor cell (see subpanels); Scale bars, 10 μm. Cells were fixed and stained with AX‐488 WGA (green); micropatterns were unstained but visible upon over‐saturation in the green channel and outlined with dotted white circles for clarity.Bar graph showing the percentage of H2B‐EBFP acceptors containing DiD‐labelled vesicles received through cell–cell contact (i.e., corrected for secretion‐based transfer) from Scramble‐ (21.8 ± 5.8%) and IRSp53 siRNA‐treated (9.7 ± 3.4%) donors. Corresponding secretion‐based transfer levels used for determining contact‐mediated transfer levels are shown for comparison and were 0.9 ± 0.5% and 1.7 ± 0.9% for Scramble and siActr3, respectively. The total number of quantified acceptor cells is indicated for each condition. Data are from three individual experiments and are represented as a mean ± SEM.Bar graph showing the relative percentage of H2B‐EBFP acceptors containing DiD‐labelled vesicles from IRSp53 siRNA‐treated donors (42.0 ± 4.5%, mean ± SEM) as compared to Scramble control donors (set to 100%). Data are from three individual experiments and were analysed using an unpaired Student's *t*‐test. Data information: The whisker box plot in (D) displays median values as internal central lines while the box edges mark the lower and upper quartiles; whiskers mark the min and max of a data set. Source data are available online for this figure.

Together the above data suggest that Eps8 and IRSp53 specifically drive long protrusion formation and that their interaction is enhanced upon Arp2/3 inhibition. To further explore this hypothesis, we tested the effect of Eps8 and IRSp53 co‐expression on TNT formation and their ability to transfer vesicles (donor–acceptor assay). Stable co‐expression of GFP‐Eps8‐ΔCAP and IRSp53‐mCherry led to a doubling in the percentage of TNT‐connected cells on *D*15 micropatterns (20.8%) compared to the GFP:mCherry control cells (10.3%) (Fig 5A and B). Notably, TNTs found in Eps8‐ΔCAP:IRSp53 cells were positive for both proteins (Fig 5A, inset ii), and both Eps8 and IRSp53 were co‐recruited into optically pulled nanotubes positive for F‐actin (Appendix Fig S9). These results support a role for these two proteins in TNT initiation and formation through IRSp53's capacity to initiate localized membrane deformations (Prévost *et al*, 2015; Tsai *et al*, 2022) that then lead to localized actin growth and the recruitment of partners like Eps8 to bundle the actin filaments in a growing TNT. Furthermore, vesicle transfer from Eps8‐ΔCAP:IRSp53 donors to H2B‐EBFP acceptors also increased (Fig 5C and D), while secretion‐based transfer remained unchanged (Appendix Fig S10). To perturb the interaction, we performed siRNA silencing against IRSp53 achieving an 89% decrease in protein expression levels (Fig 6A). As compared to Scramble control cells, IRSp53 silenced cells plated on *D*15 micropatterns showed a near abrogation in the percent of TNT‐connected cells (Fig 6B–D). We then examined whether a decrease in TNTs upon IRSp53 KD is also correlated with a reduction in TNT‐mediated cargo transfer, thus supporting a functional role for IRSp53 in TNTs. Scramble‐ and IRSp53 siRNA‐treated donor cells containing DiD‐labelled vesicles were co‐cultured with H2B‐EBFP expressing acceptor cells (Fig 6E and F) and assayed in parallel against secretion‐based controls to determine the portion of transfer mediated by cell–cell contact. Following overnight co‐culture, IRSp53 KD co‐cultures indeed showed a marked decrease in contact‐mediated transfer of vesicles (Fig 6G), reflecting a relative decrease of 58% (Fig 6H).

**Figure EV4:**
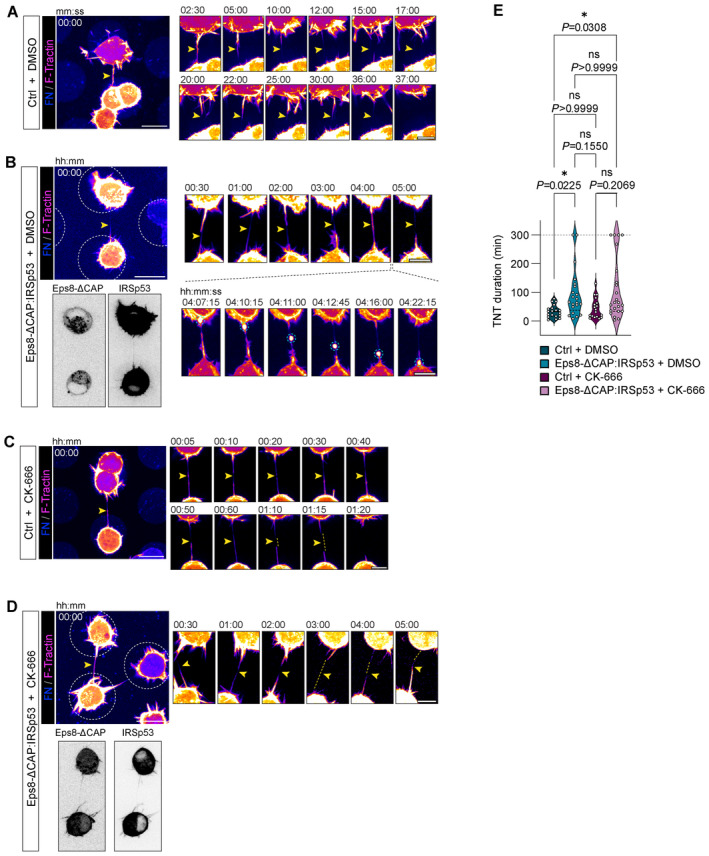
The co‐expression of Eps8 and IRSp53 alone increases the stability of TNTs Selected time frames from Movie EV15 showing the duration of a TNT connecting EGFP F‐Tractin expressing control cells treated with DMSO. Displayed images of the F‐Tractin channel (false coloured in the “Fire” lookup table) are max intensity projections of the upper stacks in the acquired Z range. At the initial time point (left large panel), the *D*15 micropatterns (AX‐405 FN, blue) visualized at the sample surface are overlayed for reference. Yellow arrowheads point to the TNT throughout its lifetime. Scale bars, 20 μm (large panel) and 10 μm (subpanels).Selected time frames from Movies EV16 and EV17 showing the duration of a TNT connecting iRFP670‐Eps8‐ΔCAP, IRSp53‐mCherry, and EGFP F‐Tractin expressing CAD cells treated with DMSO. Displayed images of the F‐Tractin channel (false coloured in the “Fire” lookup table) are max intensity projections of the upper stacks in the acquired Z range. At the initial time point, the *D*15 micropatterns (AX‐405 FN, blue) visualized at the sample surface are overlayed with the F‐Tractin channel and outlined with white dotted circles for reference (top large panel); additionally, images confirming Eps8 and IRSp53 expression are presented (bottom panels with inverted greyscales). Yellow arrowheads point to the TNT throughout its lifetime. Cyan dotted circles annotate an observed transfer event (see Movie EV17). Scale bars, 20 μm (large panel) and 10 μm (subpanels).Selected time frames from Movie EV18 showing the duration of a TNT connecting EGFP F‐Tractin expressing control cells treated with 50 μM CK‐666. Displayed images of the F‐Tractin channel (false coloured in the “Fire” lookup table) are max intensity projections of the upper stacks in the acquired Z range. At the initial time point (left large panel), the *D*15 micropatterns (AX‐405 FN, blue) visualized at the sample surface are overlayed for reference. Yellow arrowheads point to the TNT throughout its lifetime. Yellow dotted lines annotate sections of the TNT with weak F‐Tractin fluorescence. Scale bars, 20 μm (large panel) and 10 μm (subpanels).Selected time frames from Movie EV19 showing the duration of a TNT connecting i670‐Eps8‐ΔCAP, IRSp53‐mCherry, and EGFP F‐Tractin expressing CAD cells treated with 50 μM CK‐666. Displayed images of the F‐Tractin channel (false coloured in the “Fire” lookup table) are max intensity projections of the upper stacks in the acquired Z range. At the initial time point, the *D*15 micropatterns (AX‐405 FN, blue) visualized at the sample surface are overlayed with the F‐Tractin channel and outlined with white dotted circles for reference (top large panel); additionally, images confirming Eps8 and IRSp53 expression are presented (bottom panels with inverted greyscales). Yellow arrowheads point to the TNT throughout its lifetime. Yellow dotted lines annotate sections of the TNT with weak F‐Tractin fluorescence. Scale bars, 20 μm (large panel) and 10 μm (subpanels).Violin plot of TNT durations for control cells (those only expressing EGFP F‐Tractin) and cells additionally co‐expressing i670‐Eps8‐ΔCAP and IRSp53‐mCherry mock treated with DMSO or treated with 50 μM CK‐666. Median TNT durations were: Ctrl + DMSO, 33.50 min (*n* = 19); Eps8‐ΔCAP:IRSp53 + DMSO, 66.50 min (*n* = 21); Ctrl + CK‐666, 36.13 min (*n* = 18); and Eps8‐ΔCAP:IRSp53 + CK‐666, 64.75 min (*n* = 23). The black dotted line marks TNTs remaining up until the maximum allotted observational time. Statistical analysis was performed using a Kruskal Wallis test with Dunn's multiple comparison test. Adjusted *P* values for each comparison are provided on the plot.

These data support a cooperative interaction between Eps8 and IRSp53 in forming functional TNTs; if one partner in the interaction is absent, e.g., IRSp53, TNT formation is severely hampered. To further substantiate the specificity of this interaction in neuronal TNT formation, we returned to IRTKS and its overexpression (Fig EV3B) and showed it had no positive impact on the percentage of TNT‐connected cells (Fig EV3C) nor on the transfer of vesicles (Fig EV3D). Furthermore, the co‐expression of IRTKS with Eps8‐WT or Eps8‐ΔCAP showed a significant decrease in the percentage of TNT‐connected cells (Fig EV3E and F) supporting our earlier immunoprecipitation results showing that Eps8 does not coordinate with IRTKS and thus IRTKS is not involved in forming TNTs. Interestingly, we found that the addition of CK‐666 to Eps8‐ΔCAP:IRSp53 cells had no further enhancement on the percentage of TNT‐connected cells nor on vesicle transfer as compared to control GFP:mCherry cells (Fig 5B and D), suggesting that the overexpression of Eps8 with IRSp53 together may already saturate the cellular ability for stable TNTs to form. To test this, we performed live imaging of preexisting TNTs linking micropatterned Eps8‐ΔCAP:IRSp53 cells expressing F‐Tractin and measured the duration of the TNT to the point of its breakage (i.e., a proxy for its lifetime) in conditions of either DMSO or CK‐666 treatment as compared to TNT‐connected cells solely expressing F‐Tractin (control cells) (Fig EV4A–D). CK‐666 treatment had no significant effect on increasing TNT duration as compared to DMSO‐treated control cells (Fig EV4E, Ctrl + DMSO vs. Ctrl + CK‐666), signifying the dominant effect of CK‐666 treatment is its promotion of TNT numbers, not their stability. However, upon expressing Eps8‐ΔCAP:IRSp53, the median TNT duration was significantly increased from 33.50 min (Fig EV4E, Ctrl + DMSO) to 66.50 min (Fig EV4E, Eps8‐ΔCAP:IRSp53 + DMSO) and was not further increased with CK‐666 treatment (Fig EV4E, Eps8‐ΔCAP:IRSp53 + CK‐666). These data support that the saturation effect observed in Fig 5B and D arises from an increased TNT stability afforded by Eps8 and IRSp53.

### Eps8 is removed from pathways associated with filament turnover upon Arp2/3 inhibition

**Figure 7:**
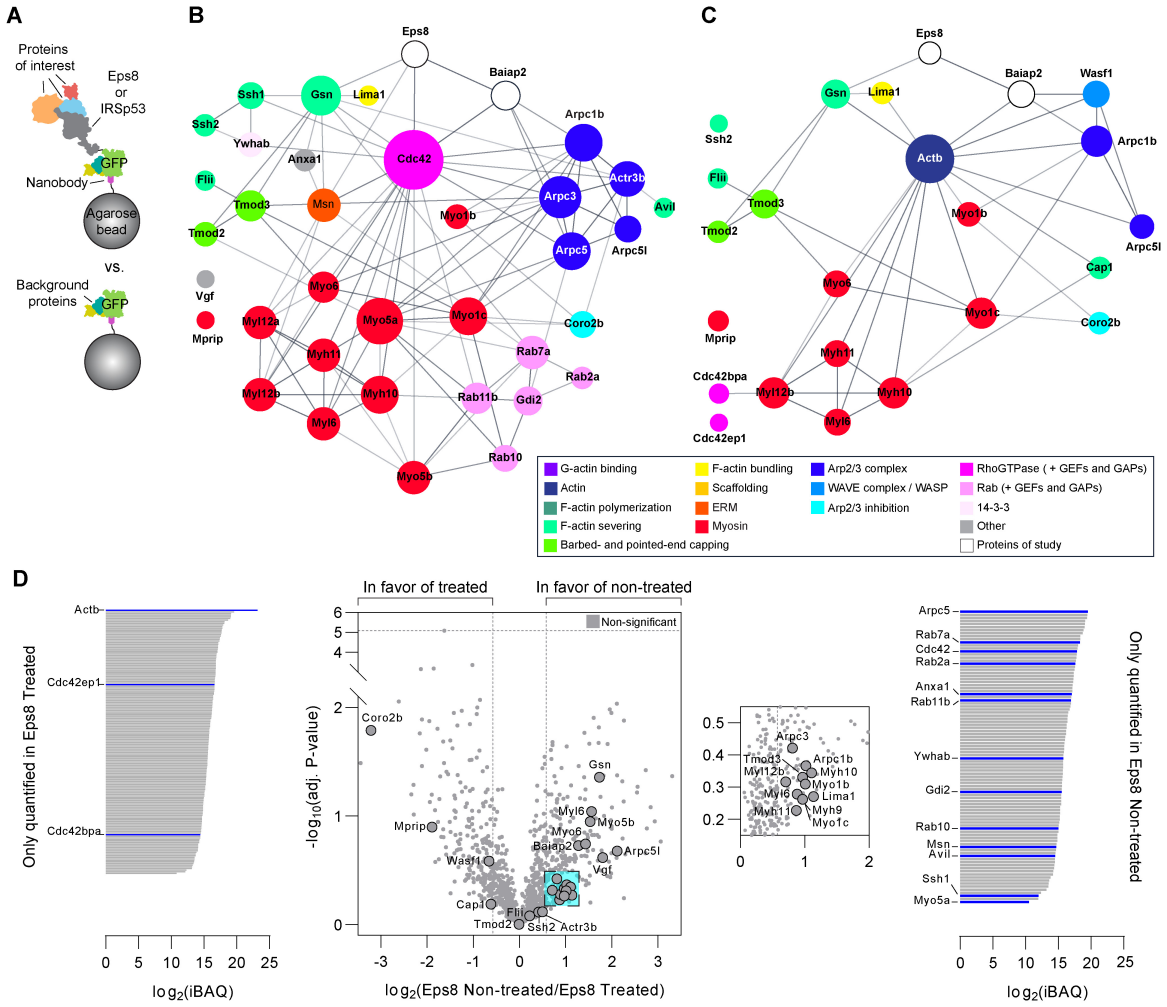
Eps8's interactome reveals a weakened interaction with Arp2/3 complex components and proteins affecting filament turnover and depolymerization upon CK‐666 treatment ASchematic depicting the GFP‐Trap immunoprecipitation strategy for identifying actin‐related protein hits associated with Eps8 and IRSp53 “bait” proteins.B, CSTRING‐generated networks of differentially abundant proteins present in Eps8‐WT as compared to the negative control for nontreated (DMSO) (B) and CK‐666‐treated (C) CAD cells. The sizes of the nodes (i.e., protein gene names) were scaled based on the degree of connectivity in the networks, and the transparency of the edges relate to the calculated score of the protein–protein interaction in the STRING database.DDirect comparison of identified protein hits between nontreated and treated Eps8 samples. Left: iBAQ plot of proteins only present in the Eps8 treated sample. Centre: Volcano plot of common proteins to both nontreated and treated Eps8 samples. Dashed vertical lines mark the binary logarithm position of a fold change of 1.5, and the dashed horizontal lines marks the adjusted *P*‐value threshold. Cyan‐shaded square corresponds to a magnified view of the indicated region on the right of the volcano plot. Right: iBAQ plot of proteins only present in the Eps8 nontreated sample.

To analyse the actin‐centric pathways through which Eps8 and IRSp53 function, GFP‐Eps8‐WT or IRSp53‐GFP were utilized as bait proteins in a whole‐cell GFP‐trap pull‐down and the interactomes were assessed against appropriately‐treated cytosolic GFP‐expressing cells (negative controls) to identify protein hits (Fig 7A). In the Eps8‐WT pull‐down, we identified 36 proteins (out of 145) (Appendix Fig S11A), and the online Search Tool for Recurring Instances of Neighbouring Genes (STRING) database (Szklarczyk *et al*, 2021) was used to generate association networks where individual proteins were grouped into actin‐based modules (Fig 7B). As expected, in control conditions Eps8 preferentially interacts with IRSp53 (Baiap2), while no other I‐BAR proteins were identified. Consistent with Eps8's role in membrane trafficking, several Rab‐related proteins were observed, e.g., Rab5 (Lanzetti *et al*, 2000). In addition, our analysis revealed novel hits in Eps8's interactome. We observed enrichment of Arp2/3 complex proteins (Arpc1b, Arpc3, Arpc5, Arpc5l, and Actr3b). We also noted proteins directly affecting F‐actin dynamics and organization, including: (1) severing proteins (gelsolin (Gsn), advillin (Avil), and flightless 1 (Flii) (Nag *et al*, 2013)) and phosphatases (Slingshot homology 1 and 2 (Ssh1 and Ssh2)) that activate cofilin (Niwa *et al*, 2002); (2) pointed‐end capping proteins (tropomodulins 2 and 3 (Tmod2 and Tmod3)) (Kumari *et al*, 2020); (3) the bundling protein eplin (Lima 1) (Maul *et al*, 2003); and (4) coronin 2b (Coro2b) that disassembles actin branches (Cai *et al*, 2007). Finally, we observed significant pull‐down of myosin‐II complex proteins, consisting of both light (Myl6, Myl12a/b) and heavy (Myh10, Myh11) chain proteins, unconventional myosin motors (Myo5a/b, Myo6), and membrane‐actin linkers (Myo1b/c).

**Figure EV5:**
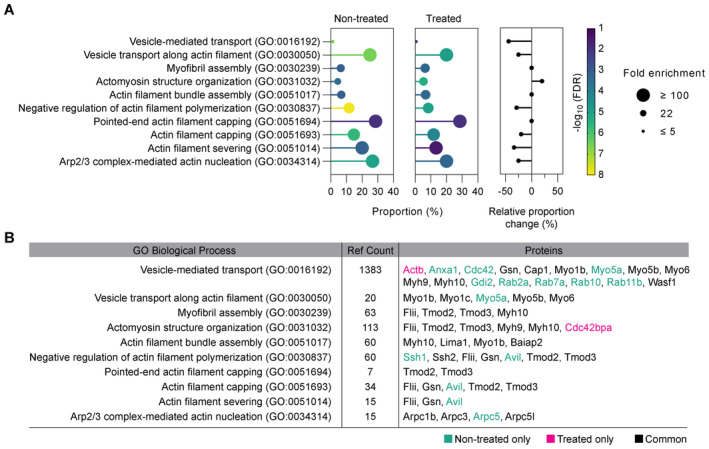
Gene ontology (GO) biological process term analysis for nontreated and CK‐666‐treated Eps8 samples Left: Proportion of protein hits for a given GO term. The number of protein hits in the network for a given GO term was normalized by the total number of proteins assigned to a given GO term using the whole mouse genome as a reference (Ref count in B). The graph is coloured by the false discovery rate (FDR). Sizes reflect the fold enrichment of the number proteins in the network divided by the number of proteins expected to be annotated with a given GO term in a randomly generated network of the same size. Right: Relative change in the proportion of proteins in a GO term when comparing CK‐666‐treated to nontreated Eps8‐WT expressing CAD cells.Table summarizing mapped proteins to their corresponding GO term for (A). The number of reference proteins in the mouse genome for a given GO term is provided (Ref count). Colour code: Teal, proteins only present in the nontreated (DMSO) Eps8 pull down; Magenta, proteins only present in the CK‐666‐treated Eps8 pull down; Black, proteins common to both pull‐downs.

After CK‐666 treatment, 23 actin‐related proteins were identified (out of 166), with several overlapping hits compared to the nontreated case (Fig 7C and Appendix Fig S11B). Direct comparison of all the proteins in the two conditions showed changes in Eps8's interactome. Arpc5 disappeared upon treatment, while other Arp2/3 complex proteins had a fold change decrease tendency (Fig 7D). Furthermore, we saw a clear change in proteins that are involved in actin assembly/disassembly. Avil and Ssh1 were lost (only found in the nontreated sample), while a greater enrichment of actin (Actb) and the Cdc42‐effector proteins Cdc42ep1 (MSE55) and Cdc42bpa (MRCKα), which promote long extensions in fibroblasts (Burbelo *et al*, 1999) and deactivate cofilin through LIM kinase activation (Sumi *et al*, 2001), respectively, were present in the CK‐666‐treated sample (Fig 7D). Gene ontological (GO) analysis (Fig EV5A and B) showed relative decreases in GO biological processes associated with the Arp2/3 complex, actin filament severing and capping, the negative regulation of actin filament polymerization, and vesicle‐mediated transport; no relative changes were observed for pointed‐end capping and F‐actin bundle assembly. Together these data reveal a novel ability for Eps8 to switch its association with different proteins when branched actin networks are inhibited.

### IRSp53 Acts as a converging platform for actin‐based processes

**Figure 8:**
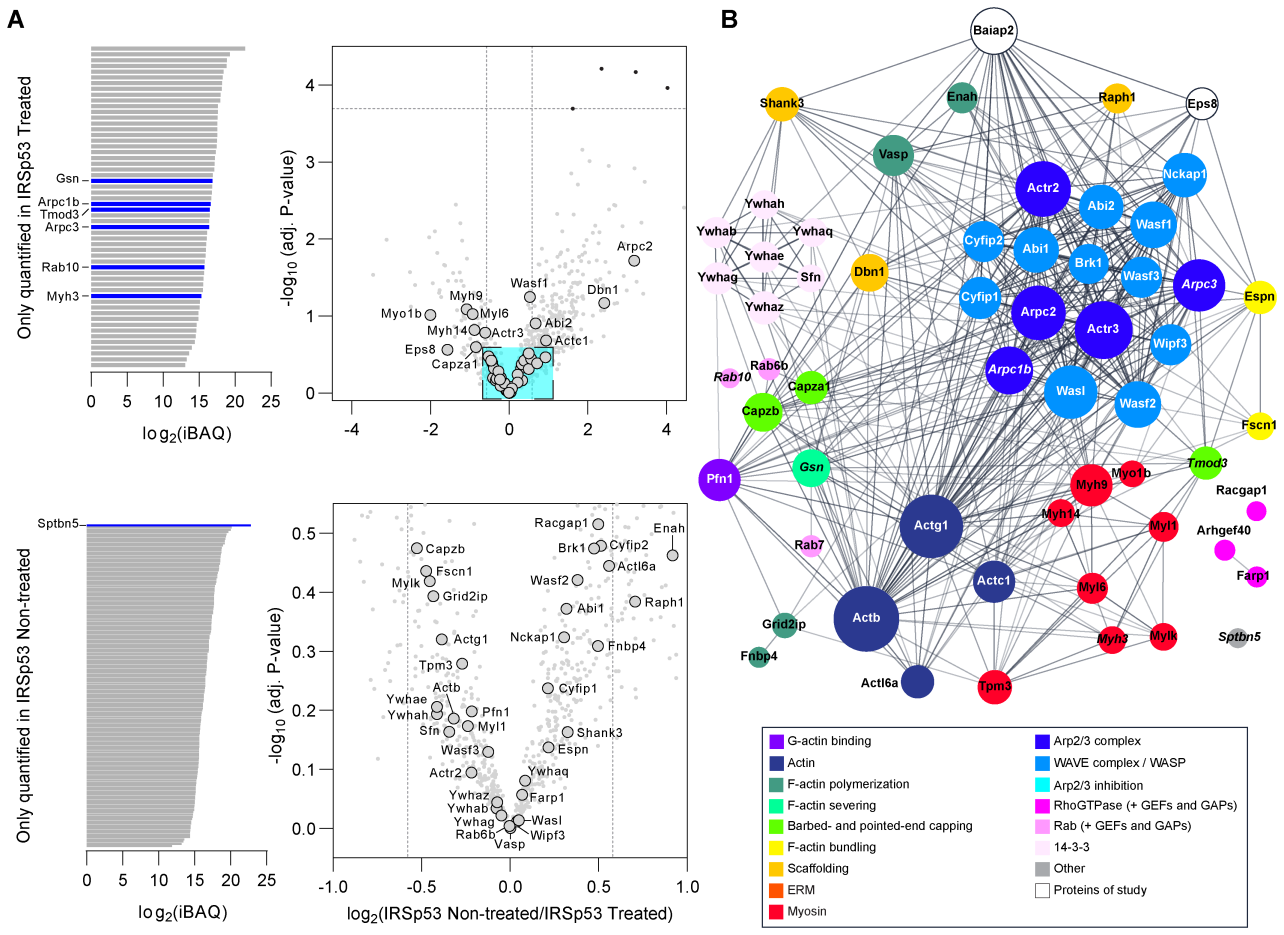
IRSp53's interactome remains invariant to Arp2/3 inhibition Direct comparison between nontreated and treated IRSp53 samples. Left: iBAQ plot of proteins only present in the treated (top) and nontreated (bottom) IRSp53 samples. Right: Volcano plots of common proteins to both nontreated and treated IRSp53 samples. Dashed vertical lines mark the binary logarithm position of a fold change of 1.5, and the dashed horizontal line marks the adjusted *P*‐value threshold. Cyan‐shaded square corresponds to a magnified view of the indicated region.Combined STRING‐generated network of all identified protein hits in the IRSp53 pull down (both nontreated and treated samples). The sizes of the nodes (i.e., protein gene names) were scaled based on the degree of connectivity in the networks, and the transparency of the edges relate to the calculated score of the protein–protein interaction in the STRING database. Italicized hits are those proteins present only in the treated (e.g., Gsn, Arpc1b, Arpc3, Tmod3, Rab10, Myh3) or nontreated (e.g., Sptbn5) IRSp53 sample shown in (A).

Our data show that IRSp53 cooperates with Eps8 to facilitate processes that lead to protrusions obtaining lengths characteristic of TNTs. How these proteins coordinate with other F‐actin regulators (e.g., polymerases) in this process is unclear. Therefore, we analysed IRSp53's interactome and identified 34 and 50 actin‐related protein hits in nontreated and CK‐666‐treated conditions, respectively (Appendix Fig S12A and B). From these hits, direct comparison of nontreated versus treated samples showed no significant difference (Fig 8A) and a STRING‐generated network (Fig 8B) shows IRSp53 has an enlarged interactome as compared to Eps8. Like with Eps8, we found IRSp53 interfaces with the Arp2/3 complex and associates with proteins such as Shank3, Drebrin (Dbn1) and Lamellipodin (Raph1) that sculpt branched networks in dendritic spines and lamellipodia (Hotulainen & Hoogenraad, 2010; Hansen & Mullins, 2015). However, far more WAVE and WASP proteins were observed for IRSp53 (11 proteins) as compared to Eps8 (Wasf1 only). Unlike Eps8's interactome, F‐actin polymerases like the formin delphilin (Grid2ip), and those known to bind IRSp53 via SH3‐mediated interactions (Vaggi *et al*, 2011; Disanza *et al*, 2013), like Vasp and Enah, were identified. IRSp53's greater link to polymerization processes appears consistent with the observation that more actin isoforms (Actb, Actc1, Actg1, Actl6a) as well as profilin (Pfn1) were present as compared to Eps8 (Fig 6). Furthermore, bundling proteins such as espin (Espn) (Sekerková *et al*, 2003) and fascin (Fscn1) were noted. Overall, these data show IRSp53 establishes a stable, multivalent interaction profile necessary to converge and promote processes affecting F‐actin polymerization; however, we did not observe major changes following CK‐666 treatment suggesting that Eps8's interaction with IRSp53 (which is increased upon Arp2/3 inhibition) switches the system towards longer actin filament formation necessary for TNTs.

## Discussion

TNTs were identified as long, actin‐supported protrusions mediating direct cargo transfer between distant cells (Önfelt *et al*, 2004; Rustom *et al*, 2004). This transfer function in different physiological and pathological contexts (Sowinski *et al*, 2008; Arkwright *et al*, 2010; Wang *et al*, 2010; Sisakhtnezhad & Khosravi, 2015; Wang & Gerdes, 2015; Abounit *et al*, 2016; Sharma & Subramaniam, 2019; Alarcon‐Martinez *et al*, 2020; Kalargyrou *et al*, 2021; Ljubojevic *et al*, 2021; Ortin‐Martinez *et al*, 2021; Scheiblich *et al*, 2021; Saha *et al*, 2022) is a key difference between TNTs and other protrusions like filopodia and cytonemes (Yamashita *et al*, 2018; Ljubojevic *et al*, 2021). While actin is ubiquitously found in TNTs, how cells develop different protrusions spanning greater distances than conventional filopodia has remained unclear, and is a remarkable feat considering any structure must rely on the polymerization of cytoskeletal elements far from the cell soma where globular actin (G‐actin) is at a higher effective concentration. In this work, we sought to address how actin‐based structures, using TNTs as a model system, can achieve the lengths necessary for connecting remote cells for communication and whether this process relies on unique cellular machinery.

We showed that TNT formation occurs at distances significantly higher than the average length of filopodia (Fig 1), and that a reduction in Arp2/3 activity (achieved here through inhibition or knockdown) favours both TNT formation (Fig 2) and actin polymerization over longer distances in nanotubes mechanically pulled from cells (Figs 3 and EV2). This indicates that TNTs result from a balance between linear and branched actin networks (Fig 2 and Appendix Fig S6). These results bear similarity to reports in yeast, and in fibroblasts and macrophages, where the inhibition of Arp2/3, or the knockout of Arpc2, promoted formin‐driven cable assembly (Burke *et al*, 2014), stress fibres and filopodia (Rotty *et al*, 2015, 2017), suggesting different actin networks are in homeostasis and compete for the same pool of polymerizable G‐actin (Suarez & Kovar, 2016). As a filopodium's steady‐state length is limited by the establishment of a G‐actin concentration gradient between the base and the barbed ends at the protrusion tip (Mogilner & Rubinstein, 2005; Lan & Papoian, 2008), the formation of longer filaments here for TNTs could in part result from an increase in G‐actin levels upon Arp2/3 inhibition.

Using Eps8 as an F‐actin bundling protein shown to enhance TNTs (Delage *et al*, 2016), we identified a preferential interaction with the I‐BAR protein IRSp53 in CAD cells (Fig 4A), and not with IRTKS (Figs 4A and EV3), that promoted functional TNTs capable of vesicle transfer (Fig 5), likely through a combined effect that promotes F‐actin bundling and stabilizes the nanotube's curved topology leading to an enhancement in TNT persistence. In support of this TNT‐promoting Eps8‐IRSp53 pathway, we found that TNT lifetimes were increased in the presence of the two proteins (Fig EV4) and a significant reduction in functional TNTs was observed when the interaction was adversely perturbed using RNA silencing against IRSp53 (Fig 6). This result bears similarity with recent work on cytonemes showing that the overexpression of a dominant‐negative IRSp53 mutant perturbed in its membrane binding capacity led to shortened cytonemes (Brunt *et al*, 2021). Furthermore, we found that Arp2/3 activity serves as an appreciable sink for the availability of Eps8 and IRSp53 (Figs 7 and 8). Notably, Eps8 associates with Arpc1b and Arpc5L isoform‐specific Arp2/3 complexes recently described as having higher branching activity (Abella *et al*, 2016). Eps8's interaction with Arp2/3 is likely direct given the scarcity of WAVE/WASP proteins observed in our pull‐down, and that diminished pull‐down of complex proteins and loss of Arpc5—which tethers Arp2 and Arp3 to the mother filament for daughter filament branching (Rouiller *et al*, 2008)—was observed upon CK‐666 treatment. This contrasts IRSp53's SH3 domain‐mediated (Miki *et al*, 2000) pull‐down of far more WAVE/WASP proteins that were unaffected by CK‐666 treatment. Such a scenario in which different pathways compete for reactants is equivalent to a mass action dependency and is a general concept controlling receptor tyrosine kinase signalling (Kiel *et al*, 2013) to cytoskeletal network sizes (Rank *et al*, 2018; Banerjee & Banerjee, 2022). Here, the reduced availability of Eps8 and IRSp53 when involved in branched networks diminishes their ability to drive outward extension of long, linear F‐actin. Indeed, upon Arp2/3 inhibition, an Eps8‐IRSp53 interaction was enhanced (Fig 4A and B), existing Eps8‐ and IRSp53‐positive protrusions further extended (Fig 4F and G, and Appendix Fig S8) and IRSp53 even relocalized to the plasma membrane driving new protrusion formation and extension (Fig 4C and E). This also explains, for example, why IRSp53 overexpression alone partially inhibited the formation of functional TNTs in CAD cells (Delage *et al*, 2016), as a new steady‐state favouring actin branching was likely promoted, an effect which is amplified if needed TNT‐factors like Eps8 are limited within the cell.

Our results show a segregation between respective areas from where lamellipodia and TNTs emerge (Fig 2 and Appendix Fig S3) suggesting spatial segregation of different actin networks is needed to form TNTs. While our study substantiates the role that competing networks have on the propensity for TNTs to form, regulation of protein availability and distribution to different pathways is needed. One possibility could involve 14‐3‐3 phospho‐binding proteins (identified in IRSp53's interactome, Fig 8) which “clamp” IRSp53 in an inactive state (Robens *et al*, 2010; Kast & Dominguez, 2019), thus preventing its membrane binding, partner associations (e.g., Eps8) and hence its downstream control of F‐actin polymerization (Tsai *et al*, 2022); however, CK‐666 treatment showed no change in 14‐3‐3 protein pull‐down in IRSp53's interactome. More likely, it is through Eps8's novel ability to switch its protein and pathway associations when Arp2/3 activity is attenuated that results in linear actin growth at longer distances (Figs 3 and EV2) and promotes TNTs (Fig 2C). As observed in Eps8's interactome changes (Fig 7), regulation of F‐actin severing could be achieved through: (i) diminished filament binding of barbed‐end capping and actin‐severing proteins (loss of Gsn, Avil and Flii) and (ii) reinforcement of processes inactivating cofilin, for example, through the loss of cofilin‐activating Slingshot phosphatases, and more LIM kinase activity and effectors (Cdc42ep1, Cdc42bpa) to deactivate cofilin. Proteins such as coronins (e.g., Coro2b identified in Eps8's interactome, Fig 7) directly dissociate Arp2/3 at branching points leading to network disassembly (Cai *et al*, 2007; Jansen *et al*, 2015) and would thus modify the frictional coupling of the actin bundle within the TNT to the cortex (Bornschlögl *et al*, 2013), diminishing retrograde flows and thus permit more growth. Similarly, the inhibition of myosin II‐mediated contractility elongated microvilli through retrograde flow reduction (Chinowsky *et al*, 2020). Thus, by attenuating processes that inhibit growth, the balance is shifted to processes that promote growth via Eps8‐ and IRSp53‐mediated interactions to directly influence F‐actin polymerization and bundling needed for TNTs. Our experimental work therefore reinforces *in vitro* reconstitution and theoretical work describing the control of F‐actin network sizes through a polymerase to capping protein balance (Antkowiak *et al*, 2019; Banerjee & Banerjee, 2022). Finally, such a biphasic behaviour for Eps8 is analogous with reports showing dendritic versus linear network antagonism is dependent upon profilin (Skruber *et al*, 2020), since WASP (Bieling *et al*, 2018), formins (Suarez *et al*, 2015) and Mena/VASP (Rotty *et al*, 2015; Skruber *et al*, 2020) all contain profilin‐binding sequences, and via the binding of tropomodulins to specific tropomyosin‐decorated actin filaments (Kumari *et al*, 2020). Our results using a formin agonist support our conclusions that the promotion of linear actin networks over branched directly favours functional TNT formation (Appendix Fig S6). It also suggests that activating diaphanous‐related formins (i.e., those such as mDia and FMNL proteins that harbour auto‐inhibitory interactions between their C‐terminal diaphanous autoregulatory domain (DAD) with an N‐terminal diaphanous inhibitory domain (DID)) could facilitate TNT elongation. However, in a preliminary screen, the effect of formin expression on TNTs in neuronal CAD cells was confounded by a strong effect on neuronal morphology which impaired the recognition of TNTs in the absence of a specific marker (Appendix Fig S13). The IMM‐01‐induced TNT increase we observed could be resultant from mDia1 and FMNL2 activity as, even with the poor expression, the transfection of these two proteins resulted in a trend to increase TNT‐connected cells (Appendix Fig S13A and B). However, the direct involvement of formins in TNT elongation needs to be further explored.

Altogether, our study shows that the formation of TNTs depends upon common protein players and a balance between G‐actin and accessory protein utilization in branched versus linear actin networks. When competing Arp2/3 networks are inhibited, this increases the availability of proteins like Eps8 and IRSp53 to exert their functions that positively lead to the extension and organization of F‐actin while limiting pathways that lead to filament turnover and disassembly. While further studies are needed to elucidate the details of how Arp2/3 networks are spatially separated and disassembled, and how severing proteins are controlled to thus favour TNT growth, our work reinforces a general principle for actin network control for cellular protrusions where simple shifts in the balance between processes that inhibit growth versus those that promote growth dictate protrusion length scales.

## Materials and Methods

### Cell culture

Mouse neuronal Cath.a‐differentiated (CAD) cells were used in this study and were a gift from Hubert Laude (Institut National de la Recherche Agronomique, Jouy‐en‐Josas, France). CAD cells were cultured in Gibco Opti‐MEM + GlutaMAX (Invitrogen) supplemented with 10% foetal bovine serum (FBS; EuroBio) and 1% penicillin/streptomycin (100 u/ml final concentration; Thermo Fisher Scientific). Cells were cultured at 37°C in a humidified 5% CO_2_ atmosphere. Cells were routinely monitored for mycoplasma contamination and found to be negative.

### Drug treatments

Drugs used in this study include the Arp2/3 complex inhibitor CK‐666 (Hetrick *et al*, 2013) and the formin agonist IMM‐01 (Lash *et al*, 2013) and were purchased from Sigma‐Aldrich. For drug treatments prior to immunoprecipitation experiments, we used 50–70% confluent CAD cells that were treated with 50 μM CK‐666 for 1 h. In the case of overnight drug treatments for TNT counting and co‐culture experiments, cells first adhered for 4–6 h and were then treated with 50 μM CK‐666 or 1 μM IMM‐01 for 16–18 h. For optical tweezer experiments, preattached cells were pretreated for 1 h with 50 μM CK‐666. Control samples were treated with an equivalent amount of DMSO which did not exceed 0.001% v/v.

### Plasmids and lentiviral constructs

IRSp53‐EGFP, IRTKS‐EGFP, IRSp53‐mCherry, and IRTKS‐mCherry were a kind gift from Pekka Lappalainen (University of Helsinki, Finland) and have been described elsewhere (Saarikangas *et al*, 2009). GFP‐Eps8‐WT and the capping‐deficient mutant GFP‐Eps8‐ΔCAP (V689D and L693D point mutations within the C‐terminal region) were a kind gift from Giorgio Scita (Istituto FIRC di Oncologia Molecolare, Italy) and have been described elsewhere (Hertzog *et al*, 2010). tdTomato‐F‐tractin and EGFP‐F‐tractin were a gift from Evelyne Coudrier (Institut Curie, France). Cytosolic GFP (pEGFP‐N1) and mCherry (pmCherry‐N1) vectors were purchased from Clontech. H2B‐mCherry was a gift from Robert Benezra (Addgene plasmid #20972), and H2B‐EBFP was a gift from Michael Davidson (Addgene plasmid #55243).

For co‐expression studies, gene inserts from GFP‐Eps8‐WT, GFP‐Eps8‐ΔCAP, and IRSp53‐mCherry were PCR amplified using PrimeSTAR GXL DNA Polymerase (Takara Bio), and the amplicons were extracted from a 1% agarose gel (Monarch Gel Extraction Kit, NEB). Inserts were then cloned into a pCDH1 lentiviral vector, cut at EcoRI and BamHI restriction sites, using In‐Fusion HD Cloning Plus (Takara Bio). Short Gly/Ser linkers were added between GFP and Eps8, and between IRSp53 and mCherry. Primers used in the cloning strategy include: EGFP‐Xho1 forward (5′‐GATTCTAGAGCTAGCGAATTCGCCACCATGGTGAGCA AGG‐3′); EGFP‐linker‐Xho1 reverse (5′‐CTCGAGGCTAGCGCTCCCACTCCCGCTTCC CTTGTACAGCTCGTCCATGCC‐3′); Linker‐mEps8 forward (5′‐GTGGGAGCGCTAGCC TCGAGATGAATGGTCATATGTCTAACCGCTC‐3′); mEps8 reverse (5′‐ATCCTTCGC GGCCGCGGATCCTCAGTGGCTGCTCCCTTC‐3′); IRSp53 forward (5′‐GATTCTAGAGC TAGCGAATTCGCCACCATGTCTCTGTCTCGCTCAGAGG‐3′); IRSp53‐linker reverse (5′‐CCGCTTCCGCTGTTTAAACGTCTTCCAGCCAGGGTGCG‐3′); linker‐mCherry forward (5′‐CGTTTAAACAGCGGAAGCGGGAGTGGGAGCGTGAGCAAGGGCGAGGAG‐3′); mCherry reverse (5′‐TCCTTCGCGGCCGCGGATCCCTACTTGTACAGCTCGT CCATGC‐3′).

An iRFP670 tagged version of Eps8‐ΔCAP was generated using the aforementioned pCDH1‐GFP‐Eps8‐ΔCAP construct as a backbone template and a vector containing the iRFP670 sequence of interest (generous gift from Mathieu Coppey, Institut Curie). Briefly, the pCDH1‐GFP‐Eps8‐ΔCAP construct was cut at EcoRI and XhoI sites to excise the GFP sequence. Following PCR amplification and agarose gel extraction of the iRFP670 amplicon, the amplicon was ligated into the cut backbone using the In‐Fusion HD Cloning Plus kit (Takara Bio) according to the manufacturer's recommendations. Primers used in the cloning strategy were: iRFP forward (5′‐CTAGAGCTAGCGAATTCGCCACCATGGTAGCAGGTCATGCCTCTGGCAG‐3′); iRFP reverse (5′‐GAGCGGTTAGACATATGACCATTCATCTCGAGCCCACTCCCGCTTCCGCTC TCAAGCGCGGTGATCCGCGTC‐3′.

### Transient transfection and knockdown

Plasmid transfections were performed using Lipofectamine 2000 (Thermo Fisher Scientific) according to the manufacturer's protocol. DNA:Lipofectamine 2000 complexes at a ratio of 1:3 (μg:μl) were added in serum‐free Opti‐MEM media on cells plated in 6‐well plates (70% confluency) and were incubated for 6 h before removal. Transfection efficiency was verified 24 h post‐transfection and transfected cells were used for further experiments.

ON‐TARGETplus siRNA pools against mouse Actr3 (L‐046642‐01) and IRSp53 (L‐046696‐01) were purchased from Horizon Discovery, while a nontargeting, universal Scramble siRNA duplex (SR30004) was purchased from Origene. siRNAs were transiently transfected into cells using serum‐free Opti‐MEM media and Lipofectamine RNAiMAX (Thermo Fisher Scientific) following the manufacture's recommendation. Briefly, CAD cells were plated in 6‐well plates to achieve 70% confluency at the time of transfection. A two‐day knockdown procedure was performed in which cells on each day were incubated with 30 pmol of siRNA over 6 h. Cells were used for further experiments at 24 h following a knockdown procedure, and knockdown efficiency was assessed by SDS‐PAGE and Western blot at the time of sample fixation (i.e., 48 h post knockdown completion).

### Stable lentiviral transduction

Lentiviral particles (LVs) were produced in HEK 293T cells cultured in Dulbecco's Modified Eagle's Medium (Thermo Fisher Scientific), supplemented with 10% FBS (EuroBio) and 1% penicillin/streptomycin (100 u/ml final concentration; Thermo Fisher Scientific) at 37°C in 5% CO_2_ humidified incubators. Cells were plated the day before transfection in T75 flasks (approx. 2 million) to achieve a 50–70% confluency the next day. Plasmids coding lentiviral components, pCMVR8.74 (Gag‐Pol‐Hiv1) and pMDG2 (VSV‐G), and the plasmid of interest at a ratio of 4:1:4 (μg), respectively, were transfected using FuGENE HD Transfection reagent (Promega) according to the manufacturer's protocol. After 48 h, LVs were concentrated using LentiX‐Concentrator (Takara Bio), and the pellet was resuspended up to 1 ml in serum‐free Opti‐MEM. CAD cells were plated the day before infection in 6‐well plates (approx. 100,000 cells) to achieve a 50–70% confluency the next day. Cells were sequentially transduced with 100 μl of the desired LVs, each for 24 h. After transduction, co‐expressing cells were treated with 2 μg/ml puromycin for 3 days. Double‐positive cells were sorted using a BD FACSAriaIII cell sorter (BD Biosciences).

### Surface micropatterning

Micropatterning on glass coverslips was performed using an adapted method from Azioune *et al* (2009). Round glass coverslips (25 mm, No. 1.5, Marienfeld) were sequentially cleaned in a bath sonicator with 70% ethanol for 10 min and 2% Hellmanex (Hellma Analytics) for 20–30 min at 30°C, and then in 1 M KOH for 20 min; copious rinsing using ultra‐pure water (resistivity > 18 MΩ·cm) from a Milli‐Q Advantage A‐10 purification system (EMD Millipore) was performed after each step. The coverslips were then dried under a stream of nitrogen and then plasma activated (Harrick Plasma) for 5 min and then functionalized overnight in a 3% v/v solution of (3‐aminopropyl)trimethoxysilane (APTMS) in acidified methanol (5% v/v acetic acid). Following aminosilanization, coverslips were briefly rinsed with 70% ethanol and then annealed in a 120°C oven for 1 h. Individual coverslips were then placed on a 60–70 μl drop of a 100 mg/ml solution of mPEG‐succinimidyl valerate (MW 5000, Laysan Bio) dissolved in 0.1 M NaHCO_3,_ pH 8.5 and left to react overnight in a humified environment to prevent evaporation. The following day, PEG‐coated coverslips were rinsed in ultrapure water and stored dry in a desiccator until they were used for deep‐UV micropatterning.

Custom micropattern designs were drawn in CleWin (WieWeb), and the chrome‐quartz photomasks were manufactured at Delta Mask (Enschede, NL). The PEG‐coated coverslips were held in contact with the photomask using a 2.5 μl drop of water (providing an estimated spacing of 5 μm), and the photomask was placed 5 cm away (~10 mW/cm^2^) from the low‐pressure mercury lamps (Heraeus Noblelight GmbH, NIQ 60/35 XL) housed within a UV box (UVO Cleaner, Jelight); coverslips were then exposed for 5 min to deep UV light (< 200 nm) through the photomask, burning exposed regions of the PEG layer to create the desired pattern on the coverslip. Subsequently, to allow for stronger covalent binding of fibronectin to the patterned areas, *N*‐hydroxysulfosuccinimide (sulfo‐NHS)/1‐ethyl‐3‐(3‐dimethylaminopropyl)carbodiimide (EDC) chemistry for amine coupling of proteins to the surface was employed. Sulfo‐NHS and EDC were dissolved in 0.1 M MES, 0.5 M NaCl, pH 6.0 at a concentration of 6 and 124 μM, respectively, and the patterned sides of the coverslips were incubated with the sulfo‐NHS/EDC solution for 30 min. Coverslips were then rinsed with ultrapure water, and then incubated for 1 h with a fibronectin (FN; Bovine plasma, Sigma‐Aldrich) solution having a total concentration of 35 μg/ml (dissolved in 0.1 M NaHCO_3_, pH 8.5). The FN solution was a mixture of unlabelled and dye‐labelled FN, where for example, fluorescent FN typically comprised 15% of the total FN concentration. The micropatterned surfaces were stored at 4°C in PBS containing 1% penicillin/streptomycin until used for cell seeding and typically used within 1 week.

### Fluorescent fibronectin

Rhodamine‐labelled FN was purchased from Cytoskeleton. Labelling of FN with Alexa Fluor 405 succinimidyl ester (Thermo Fisher Scientific) was performed in house. Briefly, lyophilized FN (Bovine plasma, Sigma‐Aldrich) was dissolved in PBS and dialyzed overnight against PBS to remove free amines. The dialyzed FN solution was then labelled at a dye to protein ratio of 70:1 (mol:mol) for 1 h at room temperature (RT) and in the dark to protect the reaction from light. Extensive dialysis against PBS was then performed to remove free Alexa Fluor dye. The labelled FN was concentrated using Amicon Ultra centrifugal filters (Merck) and aliquoted and stored at −80°C before use. The labelling efficiency for Alexa 405 FN was determined to be 30.

### Characterization and analysis of cell adherence on micropatterns

Reflection interference contrast microscopy (RICM) was performed on an inverted Eclipse Ti‐E inverted microscope (Nikon) equipped with an Antiflex 63× 1.25 NA oil immersion objective (Zeiss Plan‐Neofluar) and a Moment sCMOS camera (Teledyne Photometrics). Light from a mercury‐vapour arc lamp was bandpass filtered (546.1/10 nm, Melles Griot) and then directed towards a filter cube containing a 50/50 beam splitter and two crossed polarizers prior to entering the objective. An aliquot of suspended CAD cells (~20,000 cells) was added on top of a micropatterned substrate mounted on a Chemlide CMB 35 mm magnetic chamber (Quorum Technologies). Acquisition was controlled through MetaMorph; brightfield and RICM images were acquired every 60 s over 5 h to capture as cells begin to adhere and spread on the micropatterns. A reference fluorescence image of the FN micropatterns was taken at the first timepoint only. RICM images were variance filtered in ImageJ/Fiji to highlight the contour of the cell as the surrounding background exhibited low signal variance. Ten variance‐filtered images were then used as a training dataset in Weka for automatic segmentation of the contour of adhered cells. Segmented images were binarized, and the area was determined in ImageJ/Fiji through time.

### Tracking cell confinement on micropatterns

Brightfield and fluorescence imaging was performed on an inverted Eclipse Ti‐E or Ti2 inverted microscope (Nikon) equipped with either a CSU‐X1 or a CSU‐W1 spinning disk confocal scanning unit (Yokogawa), a fibre‐coupled Coherent OBIS laser illumination system (405/488/561/640 nm), a 40× 1.15 NA objective (CFI Apochromat LWD Lambda S, Nikon), and a Prime 95B sCMOS camera (Teledyne Photometrics). CAD cells were first preadhered for 1 h to the micropatterns and then imaged every 5 min over 18 h using MetaMorph or NIS‐Elements (Nikon) for instrumental control. Reference fluorescence images of the FN micropatterns were taken at the first timepoint only. Cells in the brightfield images were segmented from the background using an Ilastik‐trained model resultant from a four‐image training dataset. Resulting binarized images were then inputted into TrackMate to track an individual cell's centre‐of‐mass, with respect to the centre of the cell's micropattern, through the acquisition. Tracks were overlaid on images using “Track Manager” in Icy.

### Bead preparation for nanotube pulling experiments

Streptavidin‐coated polystyrene beads (3 μm in diameter, SVP‐30‐5, 0.5% w/v, Spherotech) were washed three times in a 10× volume of PBS and recovered by centrifugation (9,000 *g*, 5 min). Beads were then resuspended in PBS to a concentration of 0.05% w/v, and an appropriate amount of a 1 mg/ml biotin‐conjugated concanavalin A (ConA) solution (C2272, Sigma‐Aldrich) was added to the bead suspension assuming a binding capacity of 10 μg ConA per mg of beads. The mixture was incubated overnight at 4°C on a tabletop shaker. ConA‐coated beads were rinsed three times according to the steps above and finally resuspended in PBS to a concentration of 0.5% w/v. ConA‐beads were stored at 4°C and generally usable up to 1 month.

### Nanotube pulling experiments

A home‐built optical tweezer (OT) coupled to an inverted Eclipse Ti C1 Plus confocal microscope (Nikon) was used as previously described (Tsai *et al*, 2022). Briefly, a 3‐watt 1,064 nm Ytterbium fibre laser (IPG Photonics) was expanded through a Keplerian telescope and directed towards the back aperture of a 100× 1.45 NA oil immersion objective (Nikon CFI Plan Apochromat Lambda). The viscous drag method, including Faxen's correction for calibration near surfaces, was used to determine the OT trap stiffness which averaged 60 pN/μm. Displacements of the bead from the fixed trap centre were recorded on a CCD camera (Marlin F‐046B, Allied‐Vision) at a frame rate of 20 fps and videos were later analysed using a custom MATLAB (Mathworks) script based on the “imfindcircles” function to determine the bead's centre of mass. Forces were calculated from the bead positions according to the equation F=κ∙Δx where κ is the trap stiffness and Δx is the displacement of the bead from its initial reference position determined before nanotube pulling. A Nano‐LP100 piezo‐driven stage (MadCityLabs) was used for lateral XY movements on the setup. A temperature and CO_2_ controllable stage‐top incubator (STXG‐WELSX, Tokai Hit) maintained cells at 37°C in a humidified, 5% CO_2_ atmosphere during experimentation.

The day before experimentation, CAD cells expressing EGFP‐F‐Tractin were seeded on micropatterned coverslips in which the fibronectin patches were separated at a distance of 50 μm to ensure cells were well‐separated for micromanipulation. One‐hour prior to experimentation the phenol‐containing culture medium was removed, cells were rinsed with PBS, and exchanged for a phenol‐free Opti‐MEM medium containing: 10% FCS, ProLong™ Live Antifade Reagent (Invitrogen) at a 1:75 dilution, 2 mg/ml β‐Casein (> 98% pure, from bovine milk, Sigma‐Aldrich) for surface passivation, and 50 μM CK‐666 (or the equivalent volume of DMSO for control experiments). The cells were taken to the optical tweezer setup and labelled with Cell Mask™ Deep Red plasma membrane stain (Invitrogen) at a 1:2,000 dilution for 10 min, and ConA‐coated beads were added (1:50–1:100 dilution). Using a custom LabVIEW (National Instruments) program to control the piezo stage, membrane nanotubes were pulled by trapping an isolated floating bead, bringing it into contact with the cell for a short period of time (< 10 s), and then moving the cell away from the bead in the X direction. Confocal image acquisition using 488 nm (Coherent) and 642 nm (Melles Griot) lasers were controlled with the EZ‐C1 software (Nikon). Fluorescence was bandpass filtered (ET525/50, ET665, Chroma) and detected using τ‐SPAD single‐photon avalanche diodes (PicoQuant) that were controlled by the SymPhoTime 64 software (PicoQuant). Images encompassing the nanotube and some of the cell body (typically 1,024 × 512 pixels, 5× zoom) were gathered every 30 s after pulling.

### Quantification of actin profiles in pulled nanotubes

Image analysis was performed using custom written macros in ImageJ/Fiji. Images for the membrane and actin channels were first background subtracted, and the actin channel was then normalized by the average cytosolic signal measured within the cell interior to account for differences in F‐Tractin expression. From the membrane channel image, the nanotube's cross‐section was fit to a Gaussian function to determine the ± 2σ width of the Gaussian profile. Using the line selection tool, a line was drawn starting within the cytosol, intersecting through the plasma membrane and then following all along the length of the nanotube; the width of the line was set to the measured ± 2σ width in order to encompass the entire thickness of the nanotube. Profiles were then extracted and the actin intensity plotted as a function of the length of the nanotube *X*, where *X* = 0 was defined as the position of the max intensity value found within the plasma membrane rim near the base of the nanotube. For curve averaging, profiles were interpolated in MATLAB to obtain vectors of all the same length.

### GFP‐trap immunoprecipitation

GFP‐trap was performed using anti‐GFP nanobody‐coated agarose beads (Chromotek) and was carried out following the supplier's recommendations. Briefly, 1.2 million CAD cells expressing the GFP‐tagged “bait” protein of interest were plated in 100 mm dishes. The subsequent day the cells were briefly washed with PBS after drug treatment, and then scraped and centrifuged at 500 *g* for 3 min at 4°C. The supernatant was gently removed, and lysis buffer was added (4–5 volumes of lysis buffer for 1 volume of cell pellet). The lysis buffer consisted of 50 mM Tris, 1% Triton, 300 mM NaCl, 5 mM MgCl_2_, cOmplete™ Mini EDTA‐free Protease Inhibitor Cocktail (Merck) at pH 7.4. The cells were lysed on ice for 30 min, after which the lysate was cleared at 18,000 *g* for 30 min at 4°C. The cell lysate protein concentration was quantified using a Bradford protein assay (Bio‐Rad). Agarose beads were rinsed and equilibrated in PBS, recovered by centrifugation and then resuspended in PBS +/+ (1:1 ratio). After which 20 μl of resuspended beads were added per immunoprecipitation (IP) sample (300 μg of total protein) along with 300 μl of dilution buffer (10 mM Tris, 150 mM NaCl, 0.5% Triton, pH 7.5). The IP mixture was diluted with sterile distilled water, so that the final Triton concentration did not exceed 0.5% v/v and was then incubated for 1 h at 4°C with gentle agitation to bind proteins. Three washes of 3 min each were carried out with wash buffer (10 mM Tris, 150 mM NaCl, 0.5% Triton, pH 7.5). Finally, bound proteins were eluted with 20 μl of 2× Laemmli sample buffer (Bio‐Rad), and the sample was subsequently denatured at 100°C for 5 min.

### SDS‐PAGE and Western blot analysis

SDS‐PAGE was performed in 1× MOPS buffer (Sigma‐Aldrich) on Criterion 4–12% precast gels (Bio‐Rad). Wells were loaded with the total eluent volume from the IP samples, containing 300 μg of protein, whereas 10% (30 μg) was loaded for the lysate control samples (input). The migration was done at 120 mV for approximately 2 h. Proteins were transferred (100 V, 1 h) onto PVDF (polyvinylidene difluoride) membranes (GE Healthcare Life Sciences) using 1× Tris/Glycine buffer (in‐house 50× solution containing 250 mM Tris and 1920 mM Glycine). After transfer, the membrane was blocked using 5% nonfat milk in 0.1% TBS‐T (1× Tris‐Buffered Saline, 0.1% Tween 20, Bio‐Rad) for 1 h at RT. Primary antibodies were added at the appropriate dilution, and membranes were incubated overnight at 4°C under agitation. Primary antibodies used include: rabbit polyclonal anti‐GFP (1:4,000, A‐6455, Thermo Fisher Scientific), rabbit polyclonal anti‐IRSp53 (1:1,000, HPA023310, Sigma‐Aldrich), rabbit polyclonal anti‐IRTKS (1:2,000, HPA019484, Sigma‐Aldrich), and mouse monoclonal anti‐α tubulin (1:5,000, T5168, Sigma‐Aldrich) as a loading control. Membranes were washed three times in TBS‐T for 10 min and mouse and rabbit horseradish peroxidase (HRP)‐tagged secondary antibodies were added to the membrane at a 1:5,000 dilution in 5% milk, for 1 h at RT. Membranes were revealed using Amersham ECL Prime (Cytiva) chemiluminescent detection reagent and imaged using an ImageQuant LAS 500TM imager (GE Healthcare Life Sciences).

To quantify relative changes in band intensities (measured as an average value), regions of interest of the same size were drawn in ImageJ/Fiji to encompass the appropriate GFP band of the bait protein (indicates the amount of eluted bait protein), the band of the prey protein, and background values adjacent to the measured bands. Background‐corrected band intensities were finally normalized by taking the prey band divided by the bait band.

### TNT quantification

#### On micropatterns

CAD cells were mechanically detached in serum‐free Opti‐MEM containing 0.5 mM EGTA (to prevent cadherin‐mediated cell clumping), strained through a 40 μm nylon cell strainer (Corning) and diluted to a concentration of 175,000 cells/ml using Opti‐MEM containing 10% FBS to better ensure singularized cells. Micropatterned coverslips were generally attached to the bottom of open 35 mm dishes (P35G‐1.5‐20‐C, MatTek) using silicon grease (Dow Corning), and 60,000 cells were seeded within the volume defined by the 20 mm dish opening. Otherwise, micropatterned coverslips were placed in 6‐well plates, and 250,000 cells were used for seeding in this case. After 4–6 h of adherence, unattached cells were then removed manually using a pipette by gently rinsing with PBS. Cells were then cultured 16–18 h overnight prior to a two‐step fixation and dye staining.

Cells were fixed with 2% paraformaldehyde (16% PFA aqueous solution, methanol free, EM grade, Electron Microscopy Sciences), 0.05% glutaraldehyde (25% GA aqueous solution, Grade I, Sigma‐Aldrich), 0.2 M HEPES (1 M HEPES, Gibco) in PBS for 20 min at 37°C, followed by a second fixation in 4% PFA, 0.2 M HEPES in PBS for 20 min at 37°C. Following fixation, cells were quenched in 50 mM NH_4_Cl for 10–30 min at RT and carefully washed in PBS prior to dye labelling. Alexa Fluor 647 phalloidin or Rhodamine phalloidin (1:50–1:200 dilution in PBS, 30 min incubation in the dark at RT; Thermo Fisher Scientific) was used for F‐actin staining, WGA Alexa Fluor 488 conjugate (1:300 dilution in PBS, 20 min incubation in the dark at RT; Thermo Fisher Scientific) was used to stain the plasma membrane, and 4′,6‐diamidino‐2‐phenylindole (DAPI) (1:1,000 dilution, 2 min incubation in the dark at RT) was used to stain the nuclei. Between staining, samples were washed with PBS. Finally, samples were mounted/sealed using Aqua‐Poly/Mount (Polysciences) or Mowiol 4‐88 (Sigma‐Aldrich) and left to polymerize overnight in the dark.

Samples were imaged using a 40× 1.3 NA oil immersion objective (Zeiss Plan Apo) on an inverted confocal microscope (Zeiss LSM 700) equipped with 405/488/561/640 nm lasers and controlled by the ZEN software. The whole cellular volume, typically 12 μm in thickness, was imaged by acquiring 0.4 μm‐thick slices. Prior to analysis, image file names were randomized using the ImageJ/Fiji plugin “Filename_Randomizer” to minimize subject bias between conditions. Images were analysed in the Icy software using the “Manual TNT annotation” plug‐in (http://icy.bioimageanalysis.org/plugin/manual‐tnt‐annotation/) for manual identification and numeration of cells connected by TNTs. Connections between cells on neighbouring micropatterns were annotated as TNTs if the following criteria were met: (i) they were thin (diameter < 800 nm), (ii) membranous and F‐actin positive, (iii) did not contact the substrate and thus would be found in the middle and upper stacks, and (iv) were continuous along their length without obvious breaks (e.g., tip‐to‐tip bound filopodia) to directly link the cell bodies of the connected cells. The percent of TNT‐connected cells on micropatterns was obtained by counting the TNT‐connected cells in areas of robust patterning (areas of cell clumping were avoided as TNT formation by cell dislodgment could not be fully excluded) and dividing by the number of nearest‐neighbour cell‐occupied micropatterns (i.e., isolated cell‐occupied micropatterns were excluded since nearest‐neighbour micropatterns are unoccupied and thus incapable of having a TNT spanning between micropatterns).

#### In normal cell culture

In Appendix Fig S6 and Fig EV3, 220,000 cells were plated into 35 mm Ibidi μ‐dishes (Ibidi), cultured 18 h overnight and then fixed, stained and imaged similarly to what was described above for the micropatterned samples. As similarly stated above, the whole cellular volume was imaged and image file names were randomized prior to analysis. The percent of TNT‐connected cells was obtained by counting the TNT‐connected cells in an image and dividing by the total number of cells present in the field of view.

### Manual single‐cell analysis of TNT origin

Wild‐type CAD cells plated on *D*15 micropatterns (see TNT quantification on micropatterns, Fig 2) were classified into either a mixed cell phenotype (exhibiting hairy/filopodial and lamellipodial/ruffled characteristics) or a lamellipodia‐only phenotype. Then, the immediate cellular vicinity from which the TNT was originating was categorized. TNTs were categorized as emanating from a filopodia‐rich region, directly from a lamellipodia/ruffle region, or spatially separated above a lamellipodia/ruffle region.

### Quantification of vesicle transfer by co‐culture assays

#### By confocal microscopy

Vesicle transfer assays such as those in Appendix Fig S2, Figs 2, 5, and 6 were performed on *D*15 micropatterns, while IMM‐01 drug‐treated co‐cultures were performed in 35 mm Ibidi μ‐dishes (Appendix Fig S6). Donor cells were stained with DiD (Thermo Fisher Scientific; 1:3,000 dilution in culture media for 30 min in the dark at 37°C) to visualize vesicles, whereas cells expressing a fluorescently tagged histone 2b (H2B) fusion protein were used as acceptor cells. Predominately, H2B‐EBFP‐transfected CAD cells were used as an acceptor cell population unless otherwise stated. Donors and acceptors were co‐cultured overnight (16–18 h) at a 1:1 ratio; the total amount of cells plated in Ibidi μ‐dishes was 200,000, while the number of cells plated on *D*15 micropatterns (placed in 6‐well plates) was 250,000. In parallel, supernatant controls were performed in Ibidi μ‐dishes to measure secretion‐based vesicle transfer. Here, equal amounts of donor and acceptor cells (100,000 for Ibidi‐based co‐cultures and 125,000 for micropattern‐based co‐cultures) were separately cultured. After 16–18 h, the conditioned media of the donors was collected, briefly centrifuged (200 *g*, 4 min) to remove dead cells, and then exchanged for the media of the acceptor cell sample. The acceptors cells were then cultured for another overnight incubation with the conditioned media. All samples were fixed, stained and imaged according to the protocol described above for TNT quantification. Prior to analysis, image file names were randomized using the ImageJ/Fiji plugin “Filename_Randomizer” to minimize subject bias between conditions. Icy software was used to annotate acceptor cells with and without internalized DiD‐positive vesicles. The percent of total transfer was assessed by quantifying the number of acceptor cells containing donor‐derived DiD‐vesicles and dividing it by the total number of acceptor cells analysed across the acquired images for a given sample.

#### By flow cytometry

In Fig EV3D, CAD cells expressing IRTKS‐EGFP were stained with DiD (donor population) and cultured at a 1:1 ratio (300,000 total cells) with H2B‐mCherry expressing CAD cells (acceptor population) in 6‐well plates. In parallel, co‐cultures using EGFP expressing donor cells were used as controls. After an overnight co‐culture (16–18 h), cells were mechanically detached and washed briefly with a dilute trypsin solution (0.03% w/v trypsin in PBS) to remove noninternalized vesicles attached on the cell exterior. Cells were passed through a 40 μm nylon cell strainer to separate cell aggregates and fixed in 2% PFA. Flow cytometry data was acquired with a FACSymphony A5 flow cytometer (BD Biosciences). GFP, mCherry and DiD fluorescence were analysed at excitation wavelengths of 488, 561, and 647 nm, respectively. Ten thousand events were acquired for each condition, and data were analysed using FlowJo (BD Biosciences). The percent of total transfer was calculated by dividing the number of double positive cells (H2B‐mCherry acceptor cells containing donor‐derived DiD‐vesicles) with the total number of analysed H2B‐mCherry acceptor cells (Q2/Q1). Two biological repeats were conducted with each condition done in triplicate.

### Time‐lapse microscopy of protrusion formation

CAD cells at 50–70% confluency were co‐transfected with IRSp53‐EGFP and tdTomato‐F‐tractin or GFP‐Eps8‐ΔCAP and tdTomato‐F‐tractin according to the transfection protocol above. Six hours prior to the acquisition, 220,000 of the co‐transfected cells were plated in 35 mm dishes (Ibidi), or 250,000 cells were plated on 40 μm fibronectin micropatterns placed in 6‐well plates (Appendix Fig S8, Example 3). Samples were mounted on a Chemlide CMB 35 mm magnetic chamber (Quorum Technologies). Time‐lapse imaging was performed on an Eclipse Ti‐E inverted microscope (Nikon) equipped with a CSU‐X1 spinning disk confocal scanning unit (Yokogawa), a 60× 1.4 NA oil immersion objective (Nikon Plan Apo VC), a fibre‐coupled Coherent OBIS laser illumination system (405/488/561/640 nm), and Prime 95B sCMOS cameras (Teledyne Photometrics) for single or dual camera mode acquisition. Acquisition was controlled by MetaMorph. Two positions with z‐stack slices (0.6 μm thickness) covering the 12 μm cell volume were acquired at a 1‐min interval over 90 min (except when imaging IRSp53 recruitment at the membrane which was imaged with a 30‐s interval). Cells were imaged 30 min prior to flowing in CK‐666 at a final concentration of 50 μM, that was subsequently imaged for 60 min. A humidified 37°C and a 5% CO_2_ environment was maintained in a Life Imaging Services Gmbh enclosure.

### Assessment of TNT duration

CAD cells expressing EGFP F‐Tractin alone, or co‐expressing EGFP F‐Tractin, IRSp53‐mCherry, and iRFP670‐Eps8‐ΔCAP were plated and cultured on *D*15 micropatterns (generally labelled with AX‐405 fibronectin) minimally for 4 h and up to 16–18 h overnight before acquisition. One hour prior to the start of acquisition, samples were mounted on a Chemlide CMB 35 mm magnetic chamber (Quorum Technologies) and treated with 50 μM CK‐666 or an equivalent amount of DMSO solubilized in phenol‐free complete Opti‐MEM medium containing ProLong™ Live Antifade Reagent (Invitrogen) at a 1:75 dilution. Time‐lapse imaging of an existing TNT connecting well‐patterned CAD cells was performed on an Eclipse Ti2 inverted microscope (Nikon) equipped with a CSU‐W1 spinning disk confocal scanning unit (Yokogawa), a 60× 1.4 NA oil immersion objective (Nikon Plan Apo VC), a fibre‐coupled Coherent OBIS laser illumination system (405/488/561/640 nm), and a Prime 95B sCMOS camera (Teledyne Photometrics). Acquisition was controlled by Nikon's NIS‐Elements software, and a humidified 37°C and a 5% CO_2_ environment was maintained in an OkoLab enclosure. Z‐stack slices (0.5 μm thickness) encompassing the TNT and covering the cell volume were acquired at a 15‐s interval until the TNT broke or until the maximum allotted observational time was reached (300 min) if the TNT remained attached and unbroken. After the initial time point in which reference images for the micropatterns, Eps8 and IRSp53 were acquired, the remaining time points continued only with F‐Tractin acquisitions to prevent photo‐induced cellular toxicity.

### Filopodia quantification and length measurements

For fixed conditions (Fig 1G), filopodia were quantified using the automated analysis pipeline developed by Bagonis *et al* (2018). Maximum intensity projection images of filopodia (both surface‐ and nonattached) were created and the cell bodies were manually segmented for filopodia detection using phalloidin staining. When needed, Hyugens (Scientific Volume Imaging) was used for image deconvolution to improve filopodia detection in samples having weak actin fluorescence. Detected filopodia that had lengths less than 0.4 μm were excluded.

For time‐lapse imaging (Fig 4D and G), maximum intensity projection images were created in ImageJ/Fiji. In the first 30 min of imaging (control condition prior to CK‐666 addition), a protrusion containing actin (as F‐tractin) and the protein of interest was followed and when it reached its maximum length (e.g., just before retraction), its length was manually measured from its base to the tip. The same procedure was performed after CK‐666 treatment, with the difference being that the imaging duration was increased to 60 min in order to sample length changes in manually tracked protrusions.

### Fluorescence profile measurements

The intensity of IRSp53 at the membrane (Fig 4E) before and after the addition of CK‐666 was analysed in ImageJ/Fiji (“Plot Profile” plugin) by drawing a straight line (10 μm long) perpendicular to the cell edge. The middle of the line was positioned directly at the boundary of the cell edge and the background such that individual curves could be properly aligned and overlaid.

### Proteomic analysis by mass spectrometry

#### Sample preparation

CAD cells were plated in 100 mm dishes (800,000 cells) the day before transfection. Following a 24‐h transfection, GFP‐Eps8‐WT or IRSp53‐EGFP cells, along with cytosolic GFP‐expressing cells, were treated in parallel either with DMSO or with 50 μM CK‐666 for 1 h. After washing and lysing the cells, samples were processed for GFP‐trap immunoprecipitation (described above). After protein binding, two 3‐min washes were done in lysis buffer followed by three 3‐min washes in the dilution buffer prior to on‐bead digestion and mass spectrometry (MS) analysis. Three independent preparations were performed for Eps8, while five independent preparations were performed for IRSp53.

#### On‐bead digestion followed by LC–MS/MS analysis

Digestion was performed strictly as described by Chromotek. Briefly, beads were resuspended in digestion buffer (50 mM Tris–HCl pH 7.5, 2 M urea, 1 mM DTT and 5 μg/μl of trypsin (Promega)) for 3 min at 30°C. Supernatants were transferred to new vials and beads were washed twice using 50 mM Tris–HCl pH 7.5, 2 M urea and 5 mM iodoacetamide buffer. All washes were pooled and incubated at 32°C for overnight digestion in the dark. Peptides were purified using a standard C18‐based clean‐up protocol using a Bravo AssayMap device (Agilent). LC–MS/MS analysis of digested peptides was performed on an Orbitrap Q Exactive Plus mass spectrometer (Thermo Fisher Scientific) coupled to an EASY‐nLC 1200 (Thermo Fisher Scientific). A home‐made C18 column was used for peptide separation and consisted of a 30 cm nano‐HPLC capillary column (75 μm inner diameter) filled with 1.9 μm Reprosil‐Pur Basic C18‐HD resin (Dr. Maisch HPLC GmbH) that terminated with a silica PicoTip® emitter tip (New Objective). The column was equilibrated, and peptides were loaded in 100% solvent A (H_2_O, 0.1% formic acid (FA)) at 900 bars. Peptides were eluted at 300 nl/min using a linear gradient of solvent B (acetonitrile (ACN), 0.1% FA) from 2 to 35% during 55 min, 35 to 60% during 10 min, and 60 to 90% during 5 min (total chromatographic run was 80 min including a high ACN step and a column regeneration step). Mass spectra were acquired in the data‐dependent acquisition mode with the XCalibur 2.2 software (Thermo Fisher Scientific). Automatic switching between MS and MS/MS scans was performed to select the 10 most abundant precursor ions (top10 method) from the survey scan for higher energy collision dissociation (HCD) peptide fragmentation. Survey MS scans were acquired at a resolution of 70,000 (at m/z 400) with a target value of 3 × 10^6^ ions and was limited in range from 400 to 1700 m/z. HCD peptide fragmentation was performed with a normalized collision energy (NCE) set at 27%. Intensity threshold for ion selection was set at 1 × 10^6^ ions with a charge‐state exclusion of *z* = 1 and *z* > 7. The MS/MS spectra were acquired at a resolution of 17,500 (at m/z 400). Isolation window was set at 1.6 Th. Dynamic exclusion was employed within 30 s.

#### Protein identification and quantification

MS data were searched using MaxQuant (version 1.6.6.0) employing the Andromeda search engine (Tyanova *et al*, 2016) against a reference *Mus musculus* proteome (55,470 entries; downloaded from Uniprot June 1, 2021). The following search parameters were applied: carbamidomethylation of cysteines was set as a fixed modification, while oxidation of methionine and protein N‐terminal acetylation were set as variable modifications. The mass tolerances in MS and MS/MS were set to 5 and 20 ppm, respectively. Maximum peptide charge was set to 7, and 5 amino acids were required as a minimum peptide length. At least 2 peptides (including 1 unique peptide) were asked to report a protein identification. A false discovery rate (FDR) of 1% was set for both protein and peptide levels. Intensity‐based absolute quantification (iBAQ) values were generated from the sum of peak intensities of all peptides corresponding to a specific protein divided by the number of observable peptides. The match between runs features was allowed for biological replicate only.

#### Statistical analysis

Proteins identified in the reverse and contaminant databases and proteins “only identified by site” were first removed from the analysis. Remaining proteins with at least 2 peptides were kept for further analysis, and the associated peptide intensities were summed and log transformed (log_2_). Summed intensity values were normalized by median centering within conditions (*normalizeD* function of the R package *DAPAR*) (Wieczorek *et al*, 2017). Proteins without any iBAQ value in a condition, while present in another, have been considered as proteins quantitatively present in a condition and absent in the other. Next, missing values were imputed using the *impute.mle* function of the R package *imp4p* (preprint: Giai Gianetto *et al*, 2020). Proteins with a fold‐change inferior to 1.5 (i.e., log_2_ (FC) < 0.58) are considered not significantly differentially abundant between conditions. Statistical testing of proteins with a FC superior to 1.5 was conducted using a limma *t*‐test thanks to the R package *limma* (Smyth, 2004). An adaptive Benjamini–Hochberg procedure was applied on the resulting *P*‐values thanks to the *adjust.P* function of the R package *cp4p* (Pounds & Cheng, 2006) using the robust method described in (Giai Gianetto *et al*, 2016) to estimate the proportion of true null hypotheses among the set of statistical tests. The proteins associated to an adjusted *P*‐value inferior to an FDR level of 1% have been considered as significantly differentially abundant proteins. Thus, proteins of interest are therefore those considered to be present in one condition and absent in another, and those proteins identified based on pairwise comparison of intensities according to the adjusted *P*‐value and FC thresholds.

### Bioinformatics analysis

UniProt identifiers/accession numbers of the proteins of interest (specifically those related to the actin cytoskeleton) were inputted into the online Search Tool for Recurring Instances of Neighbouring Genes (STRING) database (Szklarczyk *et al*, 2021) to generate an interaction network visualized using Cytoscape. Medium confidence (minimum score, 0.4) interactions were sourced from databases, experiments, co‐expression, neighbourhood and gene fusion evidence. The proteins in the network were grouped manually according to their function, and the networks were then exported as .svg files for colour coding in Adobe Illustrator for easier visualization. Gene Ontology (GO) analysis of biological processes was performed using PANTHER 17.0, employing Fisher's Exact test with a false discovery rate (FDR) correction. The whole *Mus musculus* gene database was used as a reference list. Output parameters for a given GO term include: (i) the protein count in the input; (ii) a reference count (Ref count) for the total number of proteins in the mouse database assigned to a given GO term; (iii) the fold enrichment value (observed/expected) which is determined from the protein count divided by the number of proteins expected to be annotated in a randomly generated network of the same size; and (iv) the FDR that measures the significance of the fold enrichment.

### Statistical analysis

Statistical analysis was performed in GraphPad Prism. *P* < 0.05 was considered statistically significant, and all *P* values are indicated in the figures. The specific statistical tests performed, and the number of independent experiments and the total number of samples analysed are indicated in the figure captions.

## Author contributions

**J Michael Henderson:** Conceptualization; data curation; formal analysis; supervision; funding acquisition; investigation; visualization; methodology; writing – original draft; writing – review and editing. **Nina Ljubojevic:** Data curation; formal analysis; investigation; visualization; methodology; writing – original draft; writing – review and editing. **Sevan Belian:** Formal analysis; investigation; visualization; writing – review and editing. **Thibault Chaze:** Formal analysis; investigation. **Daryl Castaneda:** Investigation. **Aude Battistella:** Resources. **Quentin Giai Gianetto:** Formal analysis; investigation. **Mariette Matondo:** Resources. **Stéphanie Descroix:** Resources. **Patricia Bassereau:** Conceptualization; supervision; funding acquisition; project administration; writing – review and editing. **Chiara Zurzolo:** Conceptualization; supervision; funding acquisition; project administration; writing – review and editing.

## Disclosure and competing interests statement

The authors declare that they have no conflict of interest.

## Supporting information



Appendix

Expanded View Figures PDF

Movie EV1

Movie EV2

Movie EV3

Movie EV4

Movie EV5

Movie EV6

Movie EV7

Movie EV8

Movie EV9

Movie EV10

Movie EV11

Movie EV12

Movie EV13

Movie EV14

Movie EV15

Movie EV16

Movie EV17

Movie EV18

Movie EV19

PDF+

Source Data for Figure 1

Source Data for Figure 2

Source Data for Figure 3

Source Data for Figure 4

Source Data for Figure 5

Source Data for Figure 6

## Data Availability

All data needed to evaluate the conclusions in the paper are present in the main text and the Appendix. The mass spectrometry proteomics data have been deposited to the ProteomeXchange Consortium via the PRIDE (Perez‐Riverol *et al*, 2022) partner repository with the dataset identifier PXD035976 (http://www.ebi.ac.uk/pride/archive/projects/PXD035976).
