# Supplementary material for: Tunnelling nanotube formation is driven by Eps8/IRSp53‐dependent linear actin polymerization
Source: EMBO J. 2023 Nov 27;42(24):e113761. doi: 10.15252/embj.2023113761 (PMC10711657; doi:10.15252/embj.2023113761)
Supplement: Supplementary file 1 — Appendix [file EMBJ-42-e113761-s023.pdf]

Appendix

Tunnelling nanotube formation is driven by Eps8/IRSp53-dependent linear actin polymerization

Table of contents

Appendix Figure S1: Micropatterning approach for assessing TNT formation. 2
Appendix Figure S2: Functional TNTs connecting micropatterned cells permit vesicle transfer. 3
Appendix Figure S3: Single-cell analysis of TNT origin. 4
Appendix Figure S4: Gallery of representative images for DMSO- and CK-666-treated CAD cells adhered on micropatterns of increasing separation distances. 5
Appendix Figure S5: Gallery of representative images of Scramble control and Actr3 knockdown CAD cells adhered on micropatterns of increasing separation distances............ 6
Appendix Figure S6: Linear F-actin promotion leads to TNT-like structure formation. 7
Appendix Figure S7: Immunofluorescence reveals Eps8 and IRSp53 are endogenously present in TNTs... 8
Appendix Figure S8: Eps8 and IRSp53 are recruited to form longer protrusions upon Arp2/3 inhibition.. 10
Appendix Figure S9: Eps8 and IRSp53 are recruited into actin-positive optically pulled nanotube.. 10
Appendix Figure S10: Secretion-based transfer of DiD-labelled vesicles is invariant to CK-666 treatment. 11
Appendix Figure S11: Eps8-related proteomic data relevant for Figure 7.......................... 12
Appendix Figure S12: IRSp53-related proteomic data relevant for Figure 8...................... 13
Appendix Figure S13: Screen of formins in TNT formation.. 14

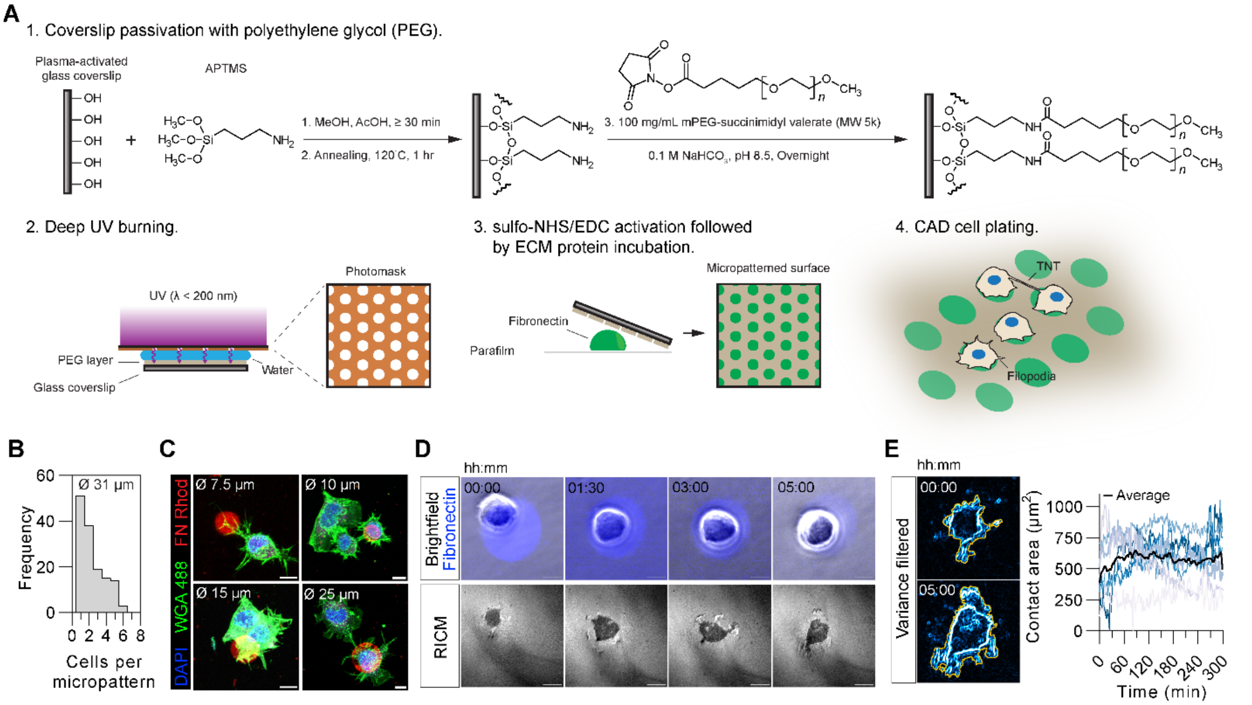

Appendix Figure S1: Micropatterning approach for assessing TNT formation.

(**A**) Scheme depicting the fabrication process of the micropatterned surfaces, including (1) coverslip passivation with covalently attached PEG molecules, (2) deep UV printing using a chrome-quartz photomask, (3) covalent attachment of fibronectin (FN) using N-sulfohydroxysuccinimide (sulfo-NHS)/1-Ethyl-3-(3-dimethylaminopropyl)carbodiimide (EDC) amine coupling chemistry, and finally (4) CAD cell plating to better discriminate TNT-connected cells from morphologically similar and shorter filopodia. (**B, C**) Micropatterns having a diameter (Ø) of 31 µm ($A \sim 750$ µm^2), which permitted more than one cell to adhere per micropattern on average, were the most optimal for CAD cell adherence and their long-term immobilization, as decreasing diameters resulted in poorer cell patterning and frequent cell infiltration in the surrounding PEG region. (**B**) Histogram of the number of cells per micropattern following overnight culture ($n = 140$ micropatterns). (**C**) Screen of different micropattern diameters for CAD cell adherence. CAD cells exhibited poorer micropatterning at Ø < 31 µm (blue, DNA labelling with DAPI; green, membrane labelling with wheat germ agglutinin (WGA); red, FN Rhodamine). (**D, E**) Reflection interference contrast microscopy (RICM) was used to measure the contact area of CAD cells through time as a proxy for their surface adhesion. (**D**) Representative brightfield and RICM images of early CAD cell adherence (over 5 hours) on FN micropatterns 31 µm in diameter (blue, FN Rhodamine). The darker contrast in RICM images indicates shorter distances between the glass surface and the adhering plasma membrane of the cell. (**E**) Left: RICM images were subjected to a variance filter and then segmented using a Weka-trained model to obtain the adhered contact area of the cell (yellow contours). Right: Plot of the contact area measured over time ($n = 7$ cells). The average contact area begins to plateau near 60 minutes (and remains stable afterwards) indicating cells have formed stable adhesions to the FN micropatterns.

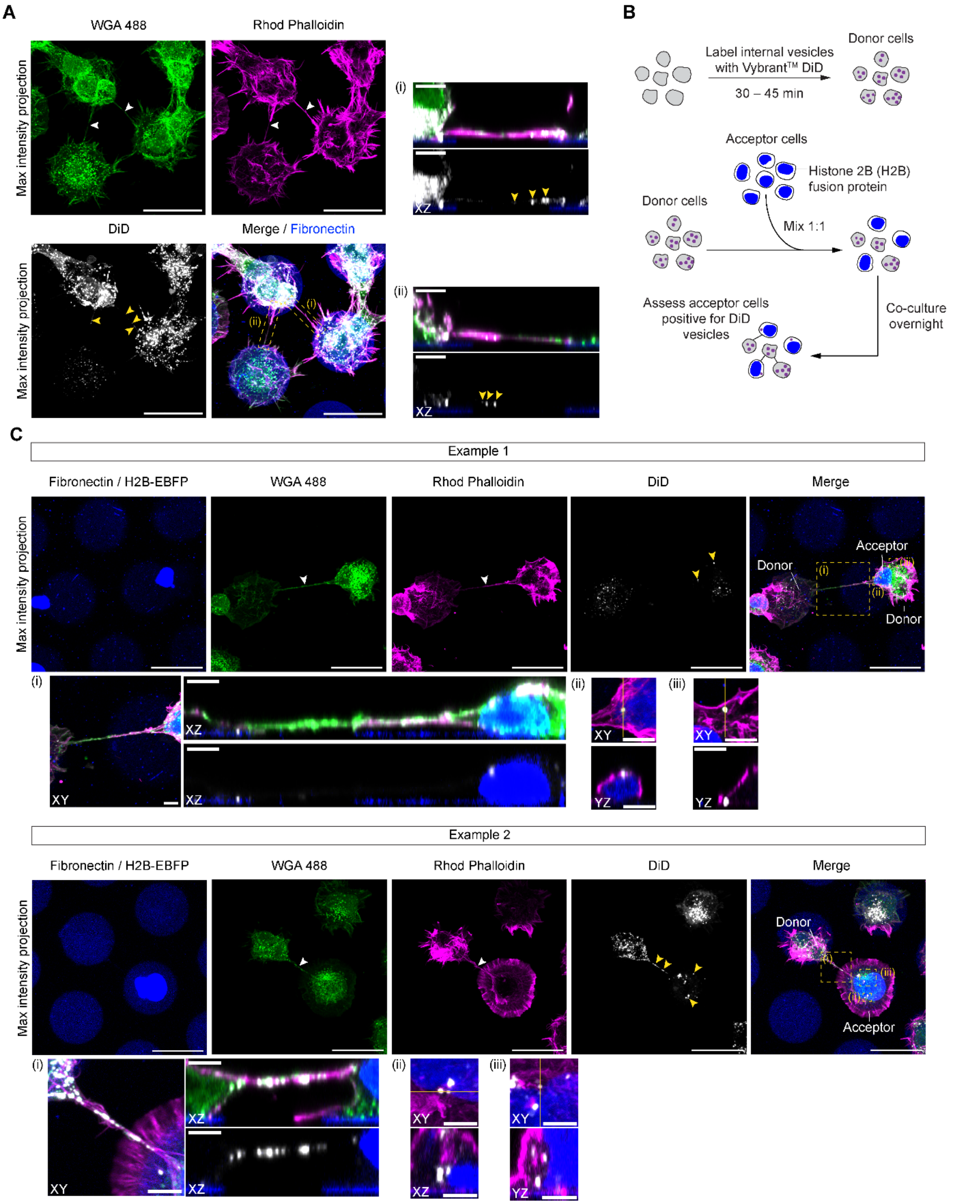

Appendix Figure S2: Functional TNTs connecting micropatterned cells permit vesicle transfer.

(**A**) Representative max intensity projections of TNTs connecting neighbouring CAD cells cultured on *D*15 micropatterns (fibronectin labelled with Alexa Flour 405) that contain DiD-labelled vesicles within them. Subpanels (i, ii) show the XZ projections through the axis of the

TNTs indicated in the dashed yellow boxes. (**B**) Scheme depicting the co-culture experiment for assessing TNT functionality through DiD-labelled vesicle transfer between donor cells to acceptor cells expressing a fluorescently tagged histone 2b (H2B) fusion protein. (**C**) Representative examples of co-cultured CAD cells plated on *D*15 micropatterns (fibronectin labelled with Alexa Flour 405). Example 1 highlights a TNT linking a donor cell with an acceptor cell positive for several donor-derived vesicles. Example 2 shows a vesicle-positive TNT connecting a DiD-labelled donor cell and a H2B-EBFP expressing acceptor cell having received donor-derived vesicles. Dashed yellow boxes correspond to labelled subpanels (i–iii) showcasing zoom-ins and orthogonal projections of TNTs and DiD vesicles within acceptor cells; XZ projections of TNTs were made through the axis of the connection. TNTs are annotated with white arrowheads; yellow arrowheads point to DiD-labelled vesicles. Scale bars, 30 µm; Insets, 5 µm.

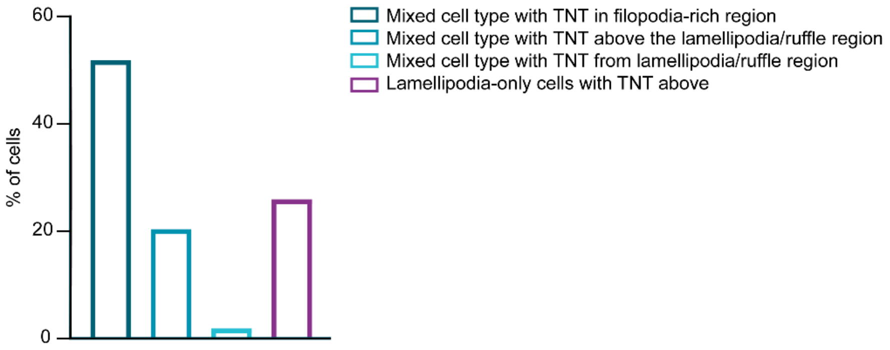

Appendix Figure S3: Single-cell analysis of TNT origin.

Bar graph showing the categorisation of TNTs and the cellular vicinity from which the TNT was originating. Single-cell analysis was performed on those cells classified in Fig. 2A and B with mixed (hairy and lamellipodial/ruffled) and lamellipodial-only phenotypes; TNTs were categorised as emanating from a filopodia-rich region, directly from a lamellipodia/ruffle region, or spatially separated above a lamellipodia/ruffle region.

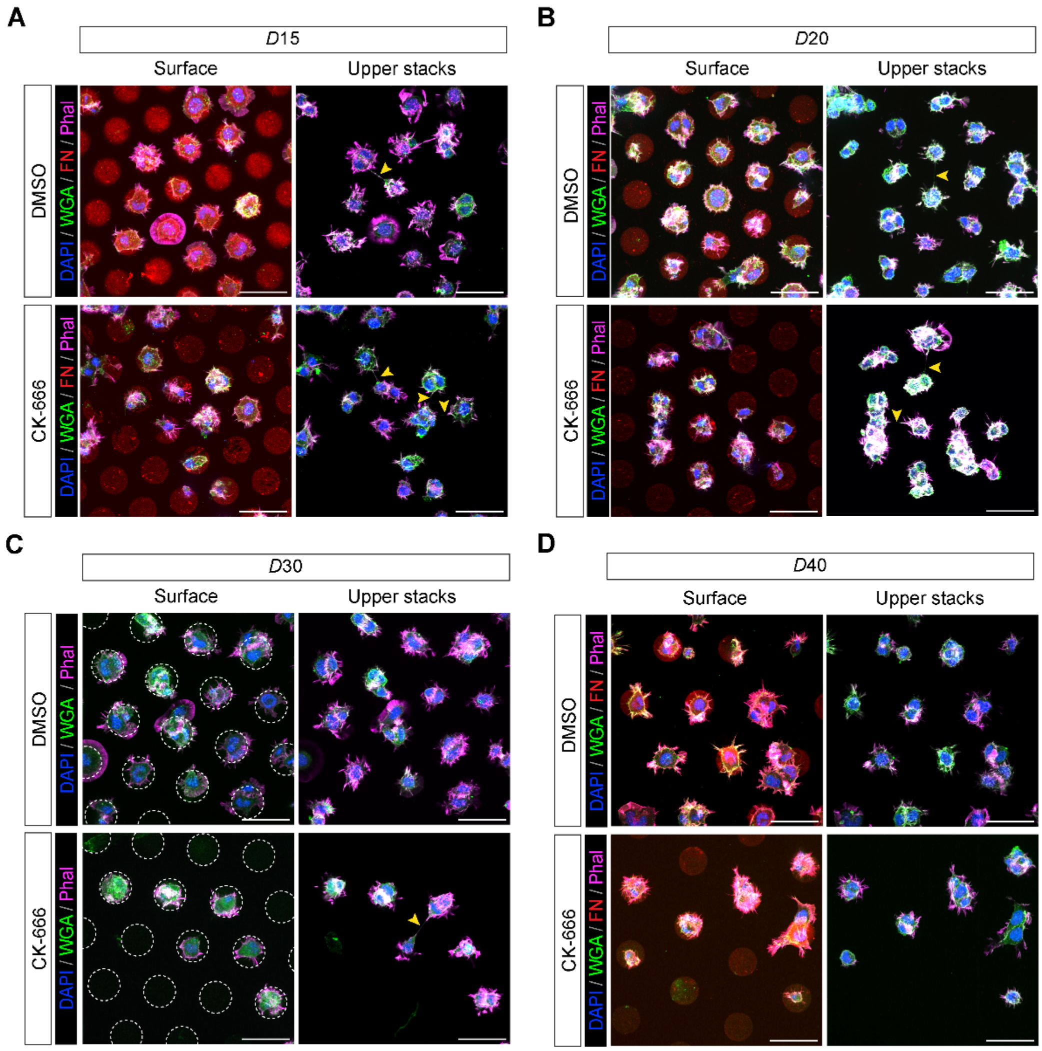

Appendix Figure S4: Gallery of representative images for DMSO- and CK-666-treated CAD cells adhered on micropatterns of increasing separation distances.
(**A**–**D**) Surface and upper stack maximum intensity projections of CAD cells plated on *D*15 (**A**), *D*20 (**B**), *D*30 (**C**) and *D*40 (**D**) fibronectin (FN) micropatterns. Upper panels show DMSO-treated cells while lower panels show CK-666-treated (50 µM) cells. Scale bars, 50 µm. TNTs connecting cells on two different micropatterns are annotated with yellow arrowheads. Cells were fixed and stained with DAPI (blue), AX-488 WGA (green) and AX-647 Phalloidin (magenta); micropatterns were generally visualized using Rhodamine FN (red). When non-fluorescent fibronectin was used, micropatterns were visible upon over-saturation in the green channel and outlined with dotted white circles for clarity.

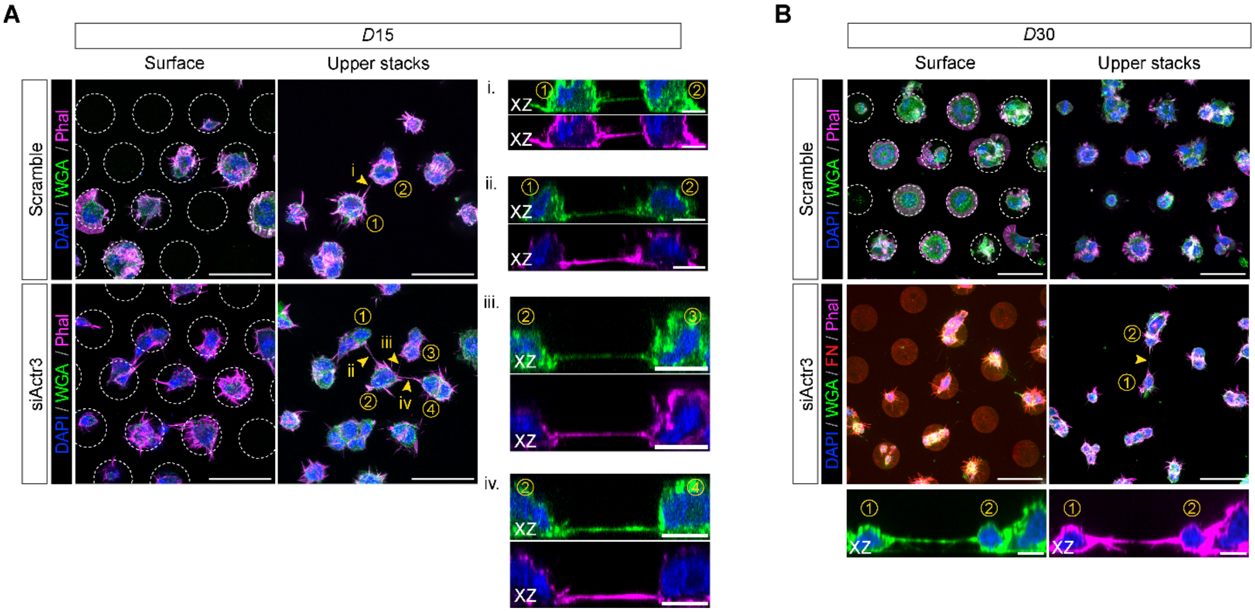

Appendix Figure S5: Gallery of representative images of Scramble control and Actr3 knockdown CAD cells adhered on micropatterns of increasing separation distances.
(**A**, **B**) Surface and upper stack maximum intensity projections of Scramble control and siActr3 CAD cells plated on *D*15 (**A**) and *D*30 (**B**) fibronectin (FN) micropattern. Scale bars, 50 µm. Subpanels (i–iv) show XZ projections made through the long axis of the indicated TNTs (yellow arrowheads); Scale bars, 10 µm. Cells were fixed and stained with DAPI (blue), AX-488 WGA (green) and AX-647 Phalloidin (magenta); micropatterns were visualized using Rhodamine FN (red). When non-fluorescent fibronectin was used, micropatterns were visible upon over-saturation in the green channel and outlined with dotted white circles for clarity.

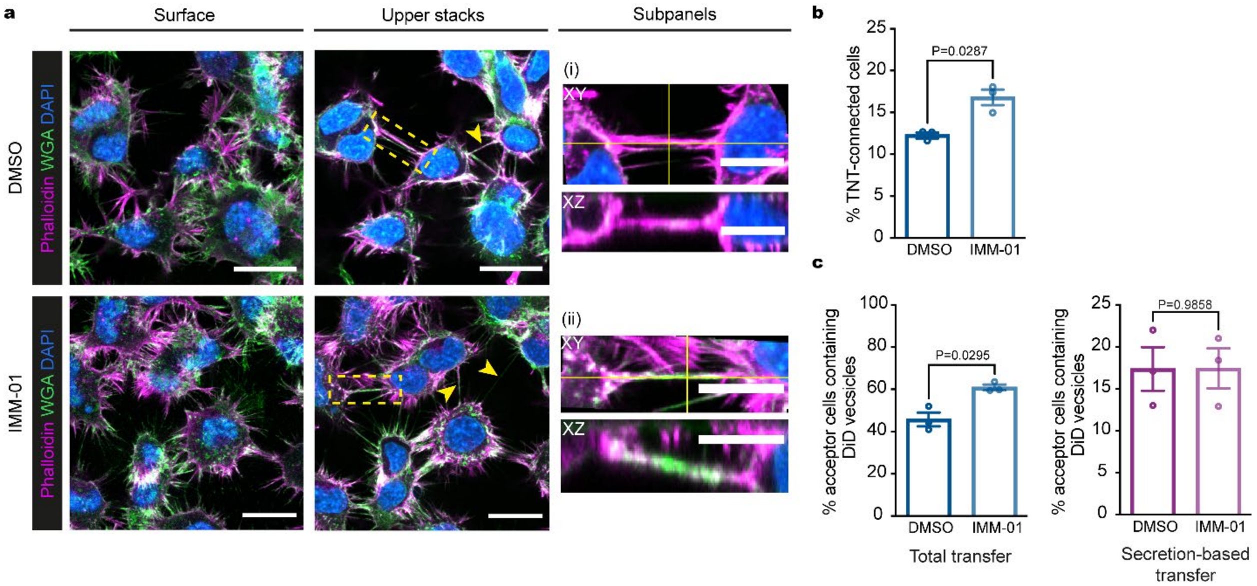

Appendix Figure S6: Linear F-actin promotion leads to TNT-like structure formation. (**A**) Representative images of surface and upper stacks of DMSO- and 1 µM IMM-01-treated cells plated on non-patterned surfaces. Yellow arrowheads annotate TNT-like protrusions. Subpanels (i, ii) show the XY and XZ projections through the axis of the TNTs indicated in the dashed yellow boxes. (**B**) Bar graph showing the quantification of TNT-like protrusions in DMSO (785 cells analysed; 12.3 ± 0.3%) and IMM-01 conditions (801 cells analysed; 16.8 ± 0.9%). Data are from 3 individual experiments and are represented as a mean ± SEM. Statistical analysis was performed using a t-test with Welch's correction, P = 0.0287. (**C**) Left: Bar graph showing the quantification of acceptor cells containing DiD-stained vesicles in the 1 µM IMM-01 treated co-culture. Data are from 3 individual experiments and are represented as a mean ± SEM. Statistical analysis was performed using a t-test with Welch's correction to compare IMM-01 (201 acceptor cells analysed; 61.0 ± 1.3%) with DMSO total co-culture transfer (202 acceptor cells analysed; 45.8 ± 3.3%), P = 0.0295. Right: Bar graph showing the quantification of acceptor cells containing DiD-stained vesicles obtained through secretion-based transfer (i.e., acceptor cells were cultured in conditioned media from DMSO- or IMM-01-treated donor cells). Data are from 3 individual experiments and are represented as a mean ± SEM. Statistical analysis was performed using a t-test with Welch's correction to compare DMSO (360 cells analysed; 17.4 ± 2.6%) versus IMM-01 (265 cells analysed; 17.4 ± 2.4%) conditions, P = 0.9858. Scale bars, 20 µm; Subpanels, 10 µm.

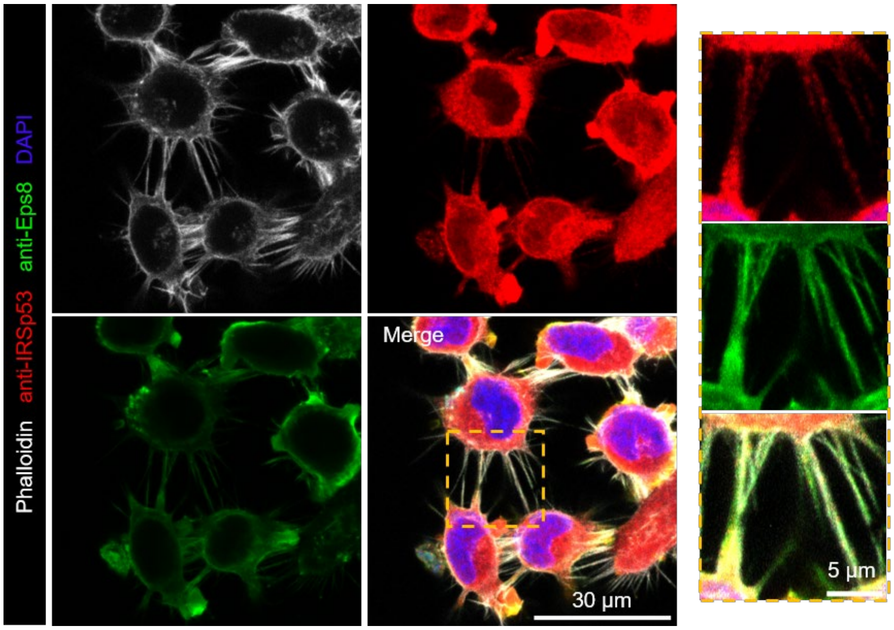

Appendix Figure S7: Immunofluorescence reveals Eps8 and IRSp53 are endogenously present in TNTs.
Representative confocal images of the upper stacks of CAD cells connected by TNTs positive for endogenous Eps8 and IRSp53. CAD cells were plated on non-micropatterned Ibidi dishes the day before sample processing. Cells were fixed in 4% PFA, permeabilized with 0.05% Saponin, and blocked in 2% BSA. Primaries: 1:100 anti-Eps8 (Mouse IgG_1, BD Biosciences, 610143); 1:500 anti-IRSp53 (Rabbit polyclonal, Atlas, HPA023310). Dashed yellow box correspond to zoom-in subpanels on the right.

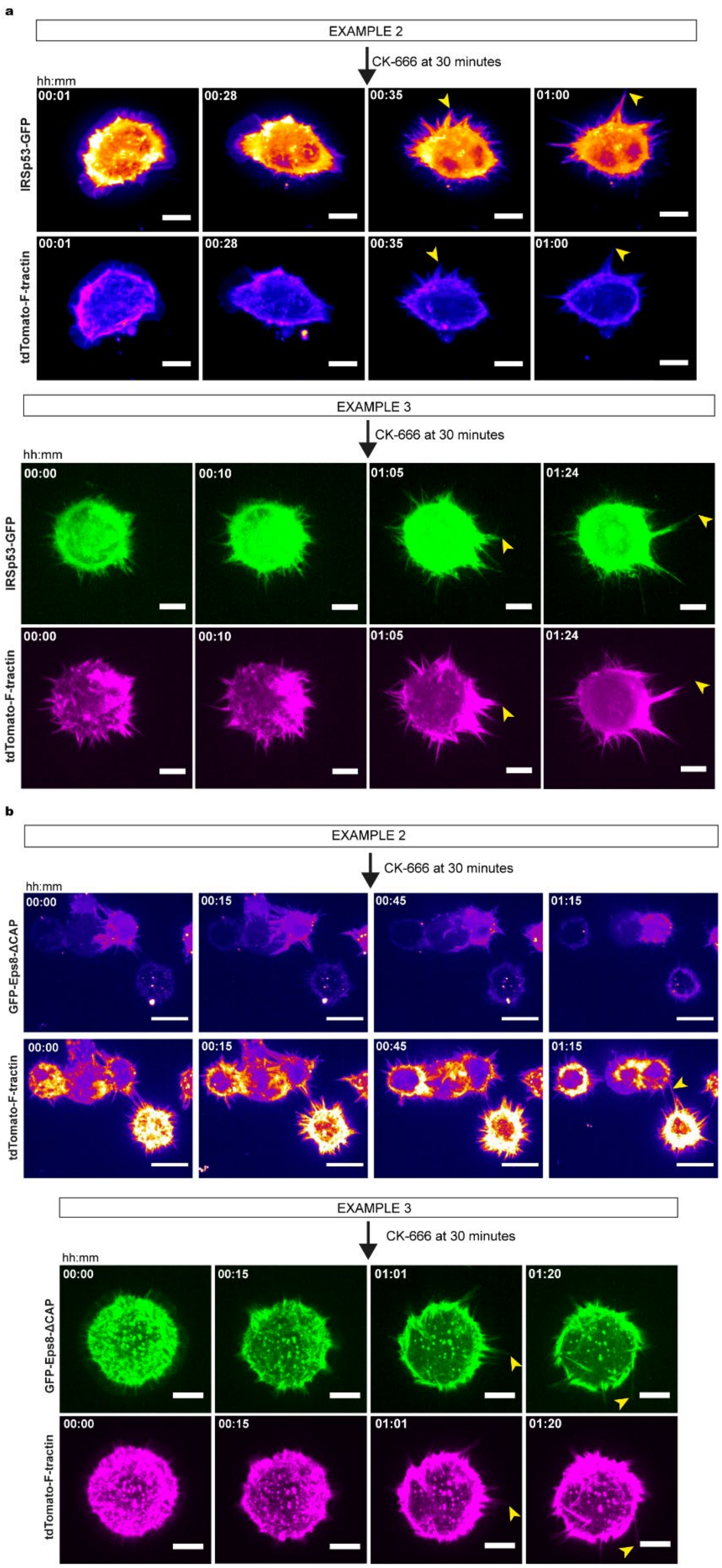
a
EXAMPLE 2
CK-666 at 30 minutes
hh:mm
IRSp53-GFP
tdTomato-F-tractin
00:01
00:28
00:35
01:00
EXAMPLE 3
CK-666 at 30 minutes
hh:mm
IRSp53-GFP
tdTomato-F-tractin
00:00
00:10
01:05
01:24
b
EXAMPLE 2
CK-666 at 30 minutes
hh:mm
GFP-Eps8-ΔCAP
tdTomato-F-tractin
00:00
00:15
00:45
01:15
EXAMPLE 3
CK-666 at 30 minutes
hh:mm
GFP-Eps8-ΔCAP
tdTomato-F-tractin
00:00
00:15
01:01
01:20

Appendix Figure S8: Eps8 and IRSp53 are recruited to form longer protrusions upon Arp2/3 inhibition.

(**a**) Representative time-lapse images of protrusion formation in IRSp53-transfected cells before and after the addition of CK-666 (at 30 min). Example 2 and Example 3 corresponds to Movie EV11 and Movie EV12, respectively. **(b)** Representative time-lapse images of protrusion formation in Eps8-ΔCAP-transfected cells before and after the addition of CK-666 (at 30 min). Example 2 and Example 3 corresponds to Movie EV13 and Movie EV14, respectively. Both IRSp53- and Eps8-ΔCAP-transfected cells additionally expressed the F-actin label tdTomato-F-tractin. Yellow arrowheads throughout show protrusions that are formed after CK-666 addition. Scale bars, 10 µm.

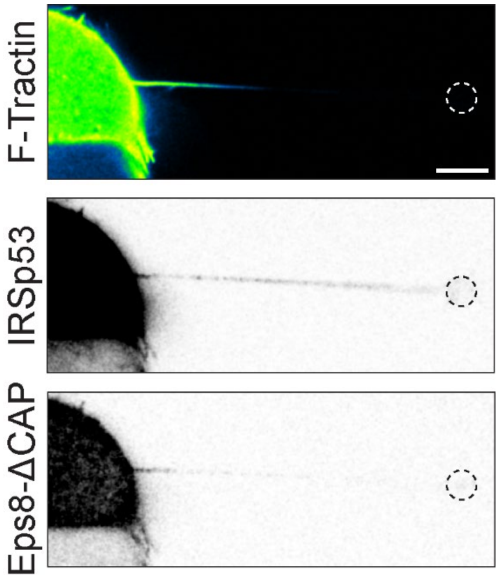

Appendix Figure S9: Eps8 and IRSp53 are recruited into actin-positive optically pulled nanotube.

Representative confocal images of a nanotube pulled form a CK-666-treated (50 µM) CAD cell triple expressing EGFP F-Tractin, IRSp53-mCherry and iRFP670-Eps8-ΔCAP. Displayed intensities for IRSp53 and Eps8 were set between 0–5 and 0–3 photon counts, respectively, for better visualization. All images were Gaussian blurred ($\sigma = 2$) to help remove spurious pixels in the background. The trapped bead is annotated with a dotted circle. Scale bar, 5 µm.

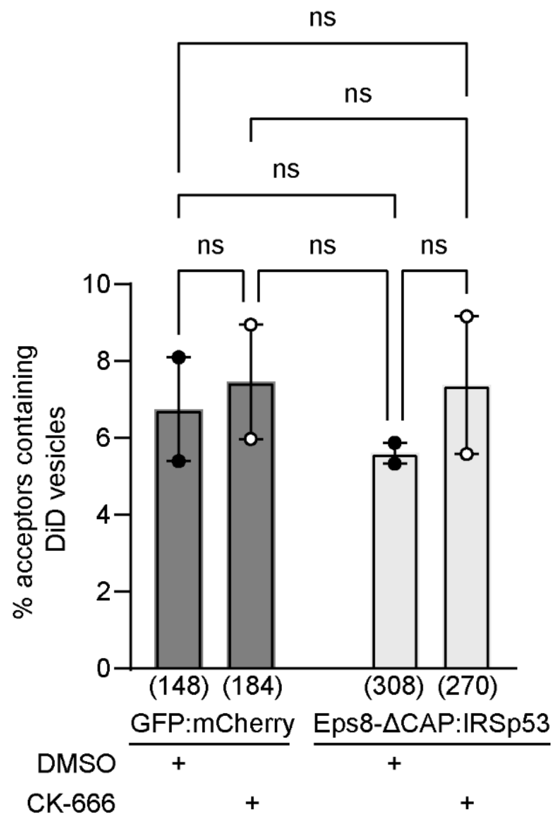

Appendix Figure S10: Secretion-based transfer of DiD-labelled vesicles is invariant to CK-666 treatment.

Plot of the percentage of acceptor cells containing DiD-labelled vesicles from secretion-based transfer experiments for GFP:mCherry + DMSO (6.8 ± 1.4%), GFP:mCherry + CK-666 (7.5 ± 1.5%), Eps8-ΔCAP:IRSp53 + DMSO (5.6 ± 0.3%), and Eps8-ΔCAP:IRSp53 + CK-666 (7.4 ± 1.8%) (mean ± SEM). Data was from two individual experiments and the total number of acceptor cells analysed in each condition is indicated below. Statistical analysis was performed using a Kruskal-Wallis multiple comparison test. P value for all comparisons > 0.9999 except GFP:mCherry + CK-666 vs. Eps8-ΔCAP:IRSp53 + DMSO was $P = 0.9183$; ns = non-significant.

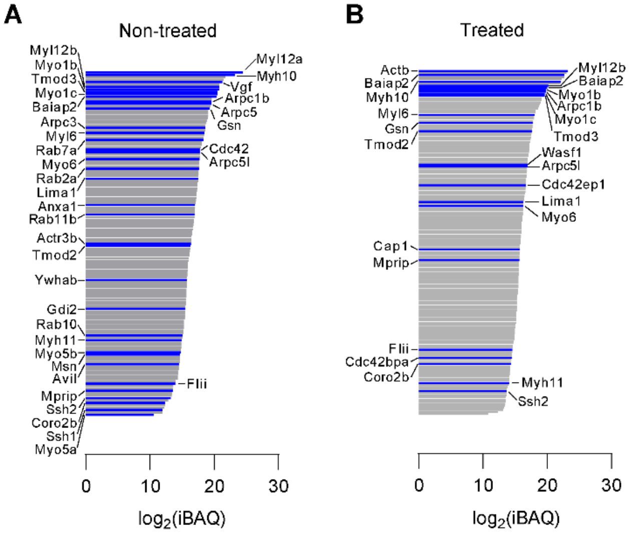

Appendix Figure S11: Eps8-related proteomic data relevant for Figure 7.
(**A**) Actin-related proteins identified as differentially abundant in Eps8-WT as compared to the negative GFP control for non-treated (DMSO) cells. (**B**) Actin-related proteins identified as differentially abundant in Eps8-WT as compared to the negative GFP control for CK-666-treated cells. Intensity-based absolute quantification (iBAQ) values were generated from the sum of peak intensities of all peptides corresponding to a specific protein divided by the number of observable peptides.

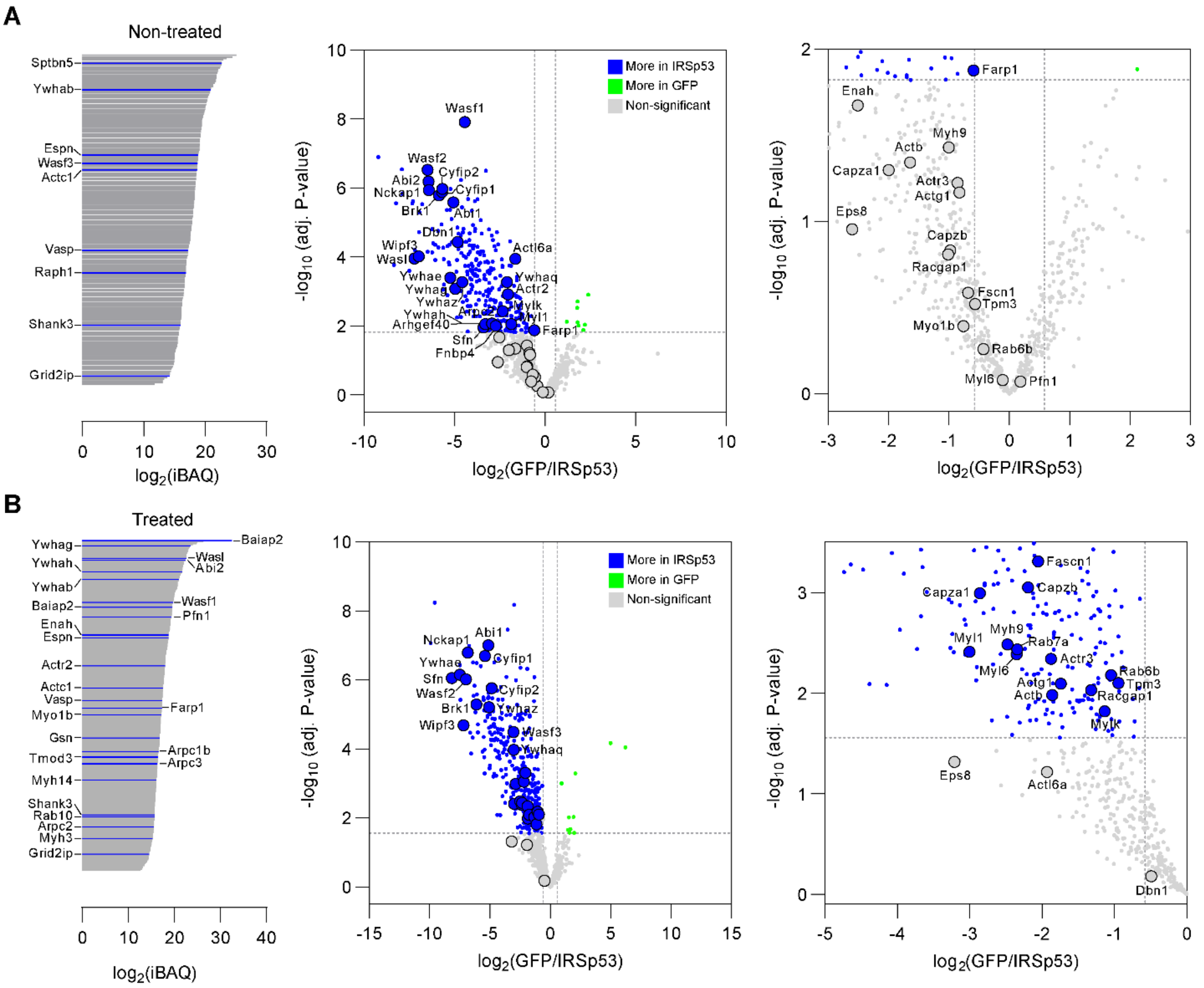

Appendix Figure S12: IRSp53-related proteomic data relevant for Figure 8.
Actin-related proteins identified in IRSp53 as compared to the negative GFP control for non-treated (DMSO) (**A**) and CK-666-trreated (**B**) CAD cells. iBAQ plots present proteins only found to be present in their respective IRSp53 samples as compared to the negative GFP control. Volcano plots show the differential abundance of identified proteins that are more present in respective IRSp53 samples.

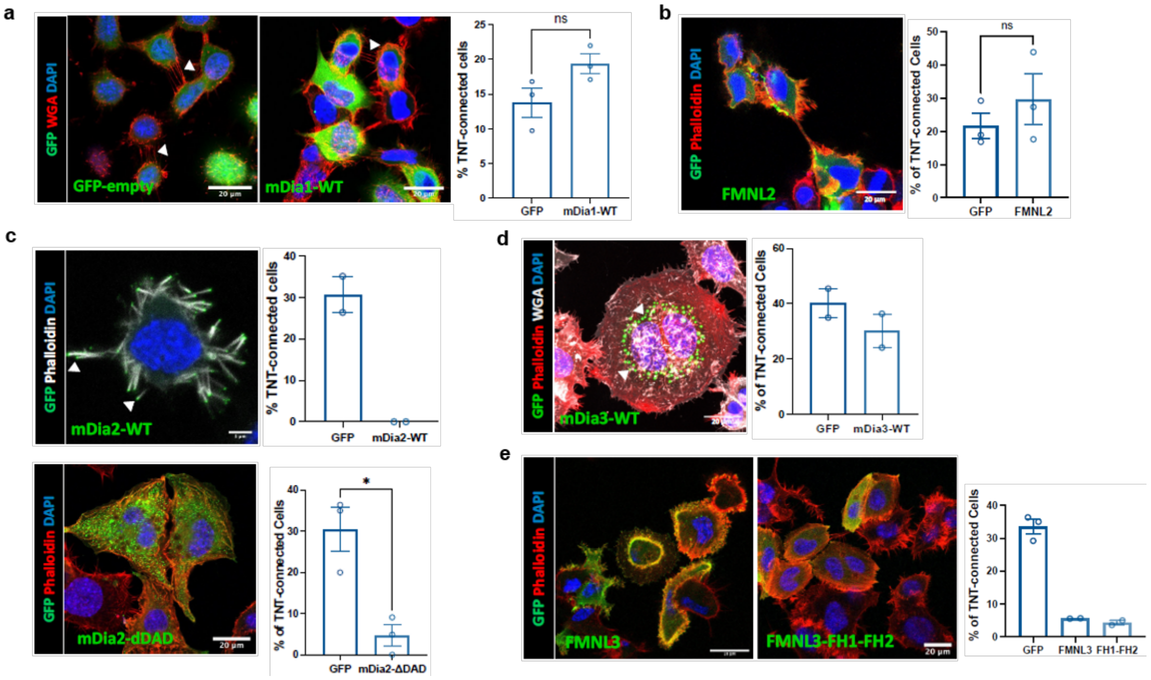

Appendix Figure S13: Screen of formins in TNT formation.

(a) Left: Representative images of the upper stacks of control GFP-empty vector expressing cells and GFP-mDia1-WT expressing cells. White arrowheads point to TNTs. Right: Bar graph (n = 3 experiments) showing the quantification of TNT-connected cells in GFP control (13.8 ± 2.1%) and in GFP-mDia1-WT (19.4 ± 1.4%). Total cells analyzed: GFP ctrl, 367; GFP-mDia1-WT, 396. Data are represented as a mean ± SEM. Statistical analysis was performed using a t-test with Welch's correction, p = 0.1048. **(b)** Left: Representative image of the upper stacks of FMNL2-GFP expressing cells. Right: Bar graph (n = 3 experiments) showing the quantification of TNT-connected cells in GFP ctrl (21.6 ± 3.8%) and in FMNL2-GFP (29.6 ± 7.6%). Total cells analyzed: GFP ctrl, 335; FMNL2-GFP, 223. Data are represented as mean ± SEM. Statistical analysis was performed using a t-test with Welch's correction, p = 0.4223. **(c)** Top: Representative images of the upper stacks of GFP-mDia2-WT expressing cells, along with a bar graph (n = 2 experiments) showing the quantification of TNT-connected cells in GFP ctrl (30.6 ± 4.4%) and in GFP-mDia2-WT (0.0 ± 0.0%). Total cells analyzed: GFP ctrl, 59; GFP-mDia2-WT, 40. Data are represented as mean ± SEM. White arrowheads indicate the mDia2 formin at the tip of filopodia. Bottom: Representative images of the maximum stack projection of GFP-mDia2-dDAD (i.e., activated mDia2) expressing cells, along with a bar graph (n = 3 experiments) showing the quantification of TNT-connected cells in GFP ctrl (30.5 ± 5.2%) and in mDia2-dDAD (4.7 ± 2.6%). Total cells analyzed: GFP ctrl, 144; GFP-mDia2-dDAD, 140. Data are represented as a mean ± SEM. Statistical analysis was performed using a t-test with Welch's correction, p = 0.0227. **(d)** Left: Representative image of the maximum stack projection of GFP-mDia3-WT expressing cells. White arrowheads indicate the accumulation of mDia3 in vesicular compartments. Right: Bar graph (n = 2 experiments) showing the quantification of TNT-connected cells in GFP ctrl (40.2 ± 5.2%) and in GFP-mDia3-WT (30.2 ± 6.1%). Total cells analyzed: GFP ctrl, 95; GFP-mDia3-WT,

102. Data are represented as a mean ± SEM. **(e)** Left: Representative images of the maximum stack projection of FMNL3-GFP and GFP-FMNL3-FH1-FH2 (i.e., an activated FMNL3 construct containing only the FH1 and FH2 domains) expressing cells. Right: Bar graph from at least two experiments showing the quantification of TNT-connected cells in GFP ctrl (33.5 ± 2.2%), FMNL3-GFP (5.6 ± 0.05%) and in GFP-FMNL3-FH1-FH2 (4.3 ± 0.7%). Total cells analyzed: GFP ctrl, 146; FMNL3-GFP, 71; GFP-FMNL3-FH1-FH2, 95. Data are represented as a mean ± SEM.
