# Supplementary material for: Tunnelling nanotube formation is driven by Eps8/IRSp53‐dependent linear actin polymerization
Source: EMBO J. 2023 Nov 27;42(24):e113761. doi: 10.15252/embj.2023113761 (PMC10711657; doi:10.15252/embj.2023113761)
Supplement: Supplementary file 2 — Expanded View Figures PDF [file EMBJ-42-e113761-s025.pdf]

Expanded View Figures

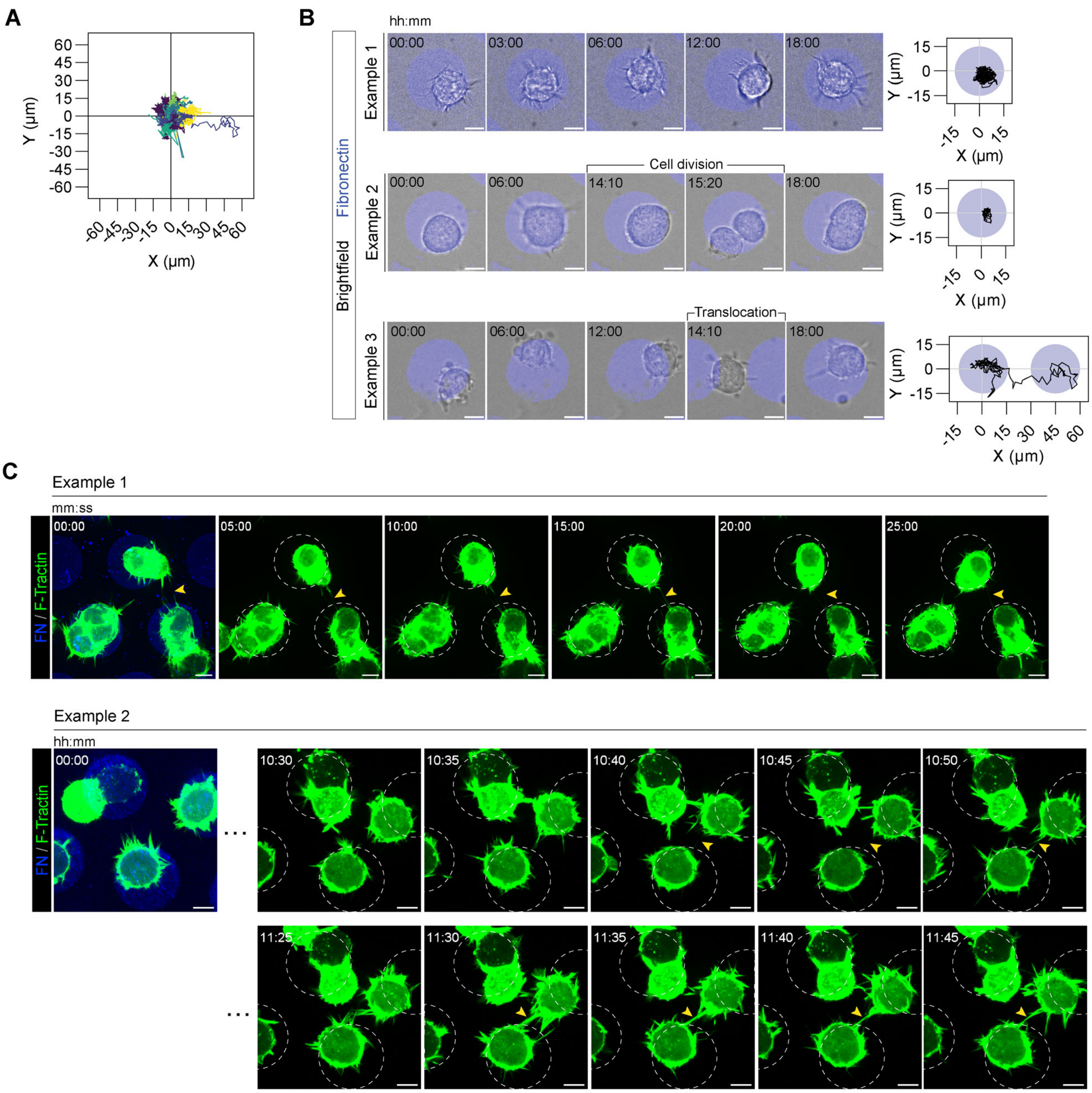

Figure EV1. TNT formation between micropatterned cells does not occur through cell dislodgment.

A, B Assessment of CAD cell confinement over an extended period of time (18 h) on *D*15 fibronectin micropatterns. (A) Plot of individual cell trajectories normalized with respect to the centre of their micropattern ($n = 56$ trajectories). (B) Representative time-lapse images of CAD cells and their corresponding trajectories. Fibronectin micropatterns are false-coloured in blue (Rhodamine fibronectin). Scale bars, 10 µm.

C Selected time frames from a 30 min (Example 1, Movie EV5) and an overnight (Example 2, Movie EV6) acquisition showing TNT-like protrusion formation between *D*15 micropatterned cells (expressing F-Tractin, green) occurs through actin-based protrusions. Images of the F-Tractin channel are max intensity projections of the upper stacks in the acquired Z range and were overlayed with the fibronectin channel to highlight cell residency to the micropatterns. White dotted circles annotate the AX-405-labelled fibronectin patterns (blue) shown on the left, and yellow arrowheads point to representative TNTs. Scale bars, 10 µm.

© 2023 The Authors

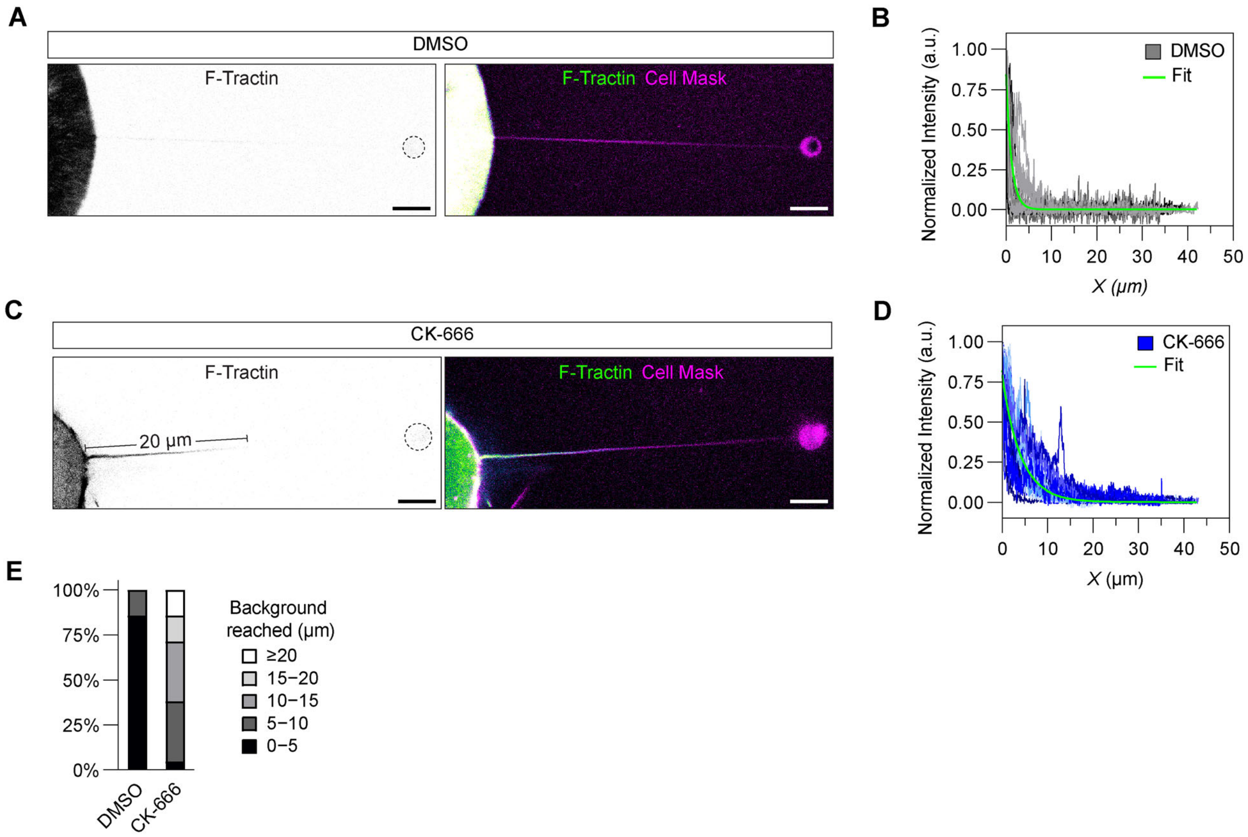

Figure EV2. Inhibition of Arp2/3 activity increases the frequency of actin polymerization occurring at extreme distances.

A Representative nanotube pulled to an extreme length from a DMSO-treated CAD cell showing little to no actin polymerization within. Displayed F-Tractin intensities were set between 0 and 10 photon counts for better visualization. The trapped bead is annotated with a dotted white circle when not clearly visible. Scale bars, 5 µm.

B Plot of actin profiles within pulled nanotubes of extreme lengths for DMSO-treated CAD cells ($n = 21$ tubes). The solid green line is an exponential fit to the combined data.

C Representative nanotube pulled to an extreme length from a CK-666-treated (50 µM) CAD cell showing actin growth 20 µm within the nanotube. Displayed F-Tractin intensities were set between 0 and 10 photon counts for better visualization. The trapped bead is annotated with a dotted white circle when not clearly visible. Scale bars, 5 µm.

D Plot of actin profiles within pulled nanotubes of extreme lengths for CK-666-treated CAD cells ($n = 21$ tubes). The solid green line is an exponential fit to the combined data.

E Categorization of the percentage of pulled nanotubes with F-actin profiles becoming indistinguishable from background intensity levels within a given micron range for DMSO- and CK-666-treated CAD cells.

 © 2023 The Authors

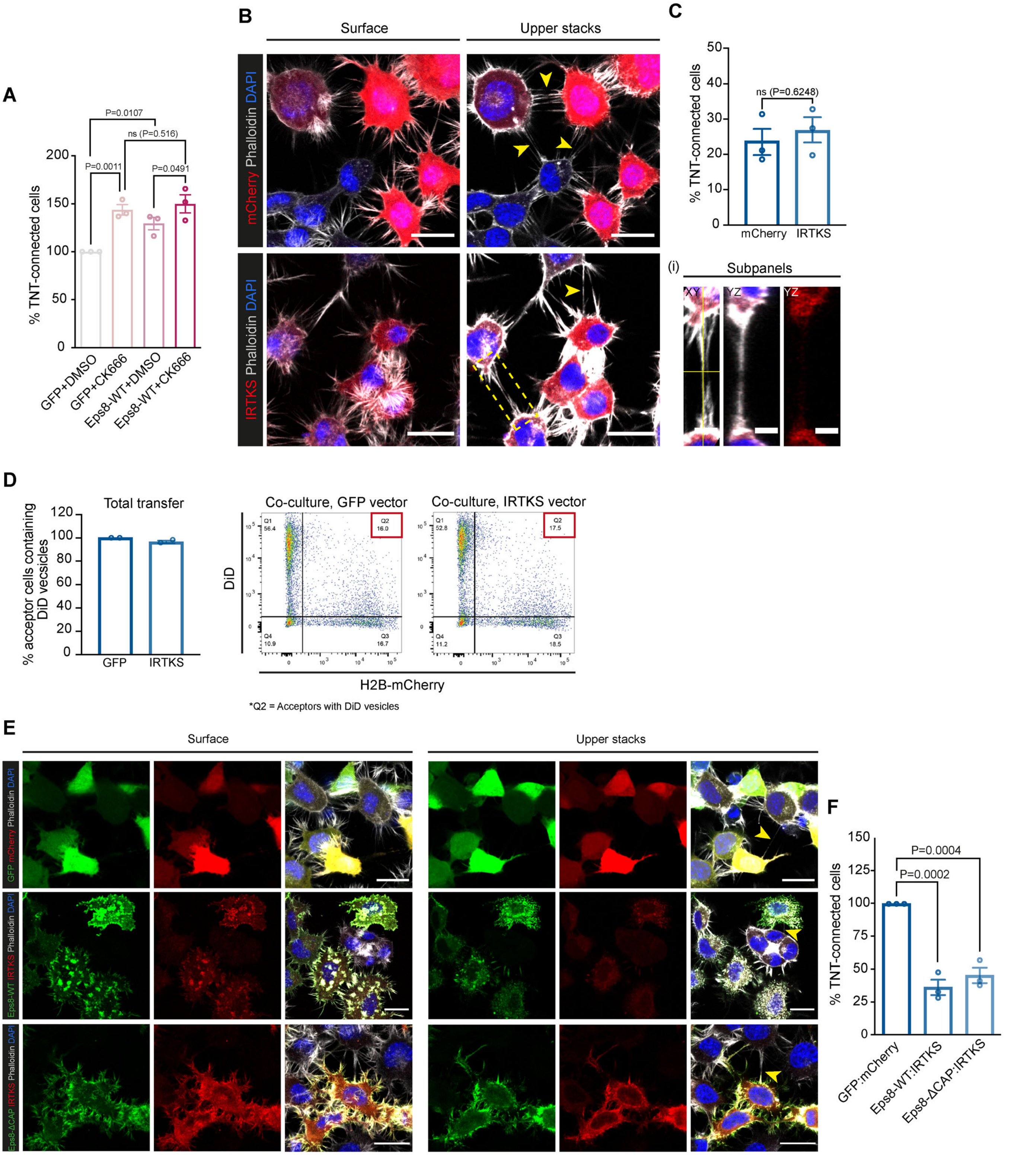
A
% TNT-connected cells
P=0.0107
ns (P=0.516)
P=0.0011
P=0.0491
GFP+DMSO
GFP+CK666
Eps8-WT+DMSO
Eps8-WT+CK666
B
Surface
Upper stacks
mCherry Phalloidin DAPI
IRTKS Phalloidin DAPI
C
% TNT-connected cells
ns (P=0.6248)
mCherry
IRTKS
(i)
Subpanels
XY
YZ
YZ
D
Total transfer
% acceptor cells containing DiD vescicles
GFP
IRTKS
Co-culture, GFP vector
Co-culture, IRTKS vector
DiD
H2B-mCherry
*Q2 = Acceptors with DiD vesicles
E
Surface
Upper stacks
GFP mCherry Phalloidin DAPI
Eps8-WT IRTKS Phalloidin DAPI
Eps8-ΔCAP IRTKS Phalloidin DAPI
F
% TNT-connected cells
P=0.0004
P=0.0002
GFP:mCherry
Eps8-WT:IRTKS
Eps8-ΔCAP:IRTKS

Figure EV3.

◀ **Figure EV3. IRTKS does not promote TNT formation, but rather decreases it when co-expressed with Eps8.**

A Bar graph showing the quantification of TNT-connected cells prior to immunoprecipitation for GFP + DMSO (297 cells analysed; 100%), GFP + CK-666 (229 cells analysed; 144.0 ± 5.4%), GFP-Eps8-WT + DMSO (224 cells analysed; 129.4 ± 6.2%) and GFP-Eps8-WT + CK-666 cells (246 cells analysed; 150.0 ± 9.5%). Data are from three individual experiments and are represented as a mean ± SEM. Statistical analysis was performed using an ordinary ANOVA with Tukey's multiple comparison test. *P* values for each comparison are stated on the bar graph.

B Representative images of surface and upper stacks of mCherry-transfected control cells and IRTKS-mCherry transfected cells plated on nonpatterned surfaces. Yellow arrowheads annotate TNT-like protrusions. Subpanels (i) show the XY and YZ projections through the axis of the TNT indicated in the dashed yellow box.

C Bar graph showing quantification of TNT-connected cells in mCherry control (309 cells analysed; 23.6 ± 4%) and in IRTKS-mCherry cells (268 cells analysed; 26.5 ± 3.9%). Data are from three individual experiments and are represented as a mean ± SEM. Statistical analysis was performed using a *t*-test with Welch's correction, $P = 0.6248$.

D Left: Bar graph showing total transfer analysis in GFP control co-culture (100%) and IRTKS-GFP co-culture (96.5 ± 1.3%). Data are from two individual experiments and are represented as a mean ± SEM. Right: Gating strategy for flow cytometry measurements of total transfer. Q2 represents H2B-mCherry-labelled acceptor cells containing donor-derived DiD vesicles.

E Left: Representative surface images of GFP:mCherry (control), GFP-Eps8-WT:IRTKS-mCherry and GFP-Eps8-ΔCAP:IRTKS-mCherry co-transfected cells. Right: Representative images of upper stacks of GFP:mCherry, GFP-Eps8-WT:IRTKS-mCherry and GFP-Eps8-ΔCAP:IRTKS-mCherry co-transfected cells. Yellow arrowheads annotate TNT-like protrusions. Cells were plated on nonpatterned surfaces.

F Bar graph showing the quantification of TNT-connected cells in GFP:mCherry (453 cells analysed; 100%), Eps8-WT:IRTKS (203 cells analysed; 36.0 ± 5.9%) and in Eps8-ΔCAP:IRTKS (252 cells analysed; 45.1 ± 5.9%) co-transfected control cells. Data are from three individual experiments and are represented as a mean ± SEM. Statistical analysis was performed using an ordinary ANOVA with Dunnett's multiple comparison test. *P* values for each comparison are stated on the bar graph.

Data information: In (B) and (E), large panel images have scale bars representing 20 μm, while the subpanel images have scale bars representing 5 μm.

 © 2023 The Authors

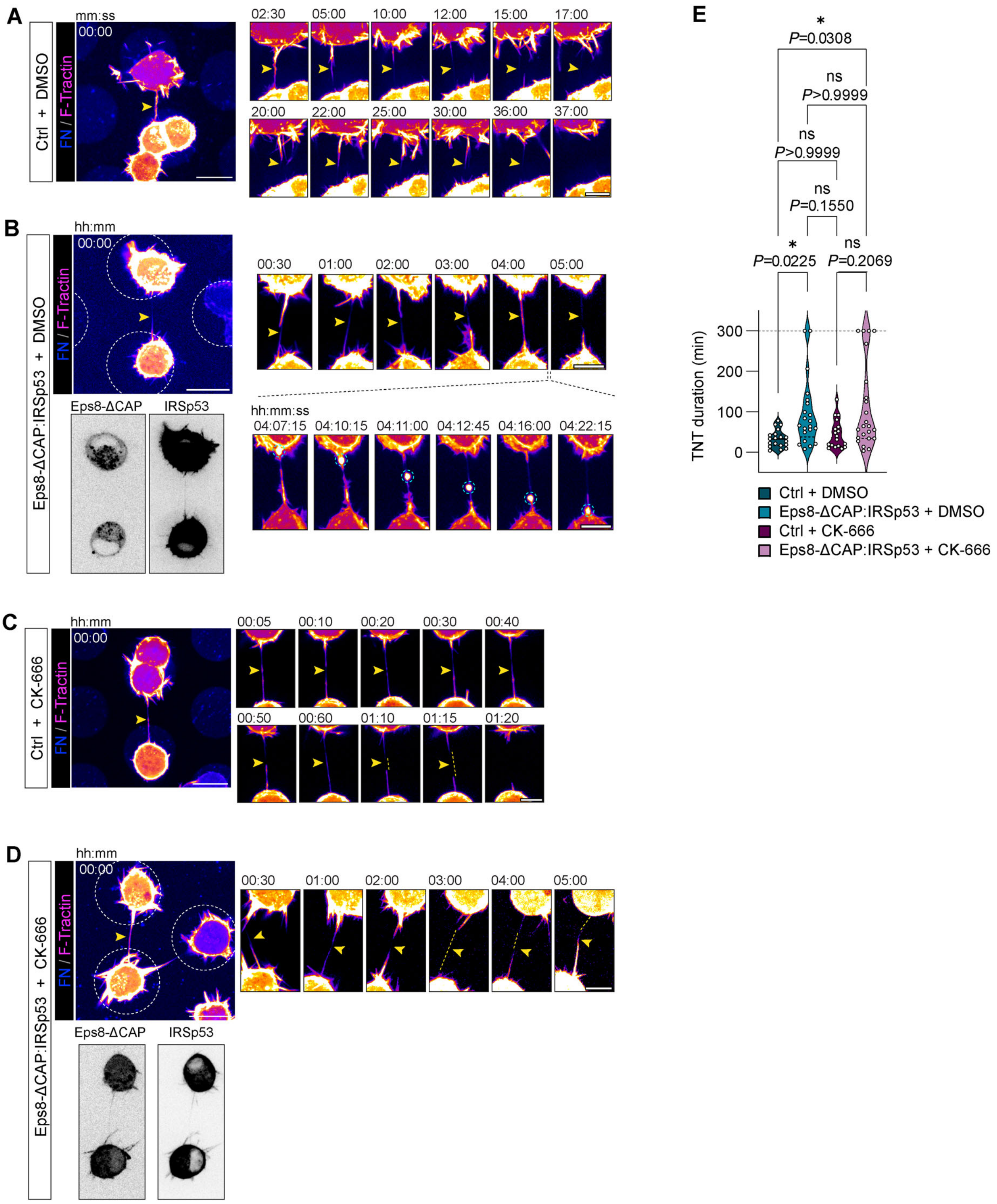
A
Ctrl + DMSO
FN / F-Tractin
mm:ss
00:00
02:30 05:00 10:00 12:00 15:00 17:00
20:00 22:00 25:00 30:00 36:00 37:00
B
Eps8-ΔCAP:IRSp53 + DMSO
FN / F-Tractin
hh:mm
00:00
00:30 01:00 02:00 03:00 04:00 05:00
Eps8-ΔCAP
IRSp53
hh:mm:ss
04:07:15 04:10:15 04:11:00 04:12:45 04:16:00 04:22:15
C
Ctrl + CK-666
FN / F-Tractin
hh:mm
00:00
00:05 00:10 00:20 00:30 00:40
00:50 00:60 01:10 01:15 01:20
D
Eps8-ΔCAP:IRSp53 + CK-666
FN / F-Tractin
hh:mm
00:00
00:30 01:00 02:00 03:00 04:00 05:00
Eps8-ΔCAP
IRSp53
E
*
P=0.0308
ns
P>0.9999
ns
P>0.9999
ns
P=0.1550
*
P=0.0225
ns
P=0.2069
TNT duration (min)
300
200
100
0
Ctrl + DMSO
Eps8-ΔCAP:IRSp53 + DMSO
Ctrl + CK-666
Eps8-ΔCAP:IRSp53 + CK-666

Figure EV4.

◀ **Figure EV4. The co-expression of Eps8 and IRSp53 alone increases the stability of TNTs.**

A Selected time frames from Movie EV15 showing the duration of a TNT connecting EGFP F-Tractin expressing control cells treated with DMSO. Displayed images of the F-Tractin channel (false coloured in the "Fire" lookup table) are max intensity projections of the upper stacks in the acquired Z range. At the initial time point (left large panel), the *D*15 micropatterns (AX-405 FN, blue) visualized at the sample surface are overlayed for reference. Yellow arrowheads point to the TNT throughout its lifetime. Scale bars, 20 μm (large panel) and 10 μm (subpanels).

B Selected time frames from Movies EV16 and EV17 showing the duration of a TNT connecting iRFP670-Eps8-ΔCAP, IRSp53-mCherry, and EGFP F-Tractin expressing CAD cells treated with DMSO. Displayed images of the F-Tractin channel (false coloured in the "Fire" lookup table) are max intensity projections of the upper stacks in the acquired Z range. At the initial time point, the *D*15 micropatterns (AX-405 FN, blue) visualized at the sample surface are overlayed with the F-Tractin channel and outlined with white dotted circles for reference (top large panel); additionally, images confirming Eps8 and IRSp53 expression are presented (bottom panels with inverted greyscales). Yellow arrowheads point to the TNT throughout its lifetime. Cyan dotted circles annotate an observed transfer event (see Movie EV17). Scale bars, 20 μm (large panel) and 10 μm (subpanels).

C Selected time frames from Movie EV18 showing the duration of a TNT connecting EGFP F-Tractin expressing control cells treated with 50 μM CK-666. Displayed images of the F-Tractin channel (false coloured in the "Fire" lookup table) are max intensity projections of the upper stacks in the acquired Z range. At the initial time point (left large panel), the *D*15 micropatterns (AX-405 FN, blue) visualized at the sample surface are overlayed for reference. Yellow arrowheads point to the TNT throughout its lifetime. Yellow dotted lines annotate sections of the TNT with weak F-Tractin fluorescence. Scale bars, 20 μm (large panel) and 10 μm (subpanels).

D Selected time frames from Movie EV19 showing the duration of a TNT connecting i670-Eps8-ΔCAP, IRSp53-mCherry, and EGFP F-Tractin expressing CAD cells treated with 50 μM CK-666. Displayed images of the F-Tractin channel (false coloured in the "Fire" lookup table) are max intensity projections of the upper stacks in the acquired Z range. At the initial time point, the *D*15 micropatterns (AX-405 FN, blue) visualized at the sample surface are overlayed with the F-Tractin channel and outlined with white dotted circles for reference (top large panel); additionally, images confirming Eps8 and IRSp53 expression are presented (bottom panels with inverted greyscales). Yellow arrowheads point to the TNT throughout its lifetime. Yellow dotted lines annotate sections of the TNT with weak F-Tractin fluorescence. Scale bars, 20 μm (large panel) and 10 μm (subpanels).

E Violin plot of TNT durations for control cells (those only expressing EGFP F-Tractin) and cells additionally co-expressing i670-Eps8-ΔCAP and IRSp53-mCherry mock treated with DMSO or treated with 50 μM CK-666. Median TNT durations were: Ctrl + DMSO, 33.50 min (n = 19); Eps8-ΔCAP:IRSp53 + DMSO, 66.50 min (n = 21); Ctrl + CK-666, 36.13 min (n = 18); and Eps8-ΔCAP:IRSp53 + CK-666, 64.75 min (n = 23). The black dotted line marks TNTs remaining up until the maximum allotted observational time. Statistical analysis was performed using a Kruskal Wallis test with Dunn's multiple comparison test. Adjusted P values for each comparison are provided on the plot.

 © 2023 The Authors

A

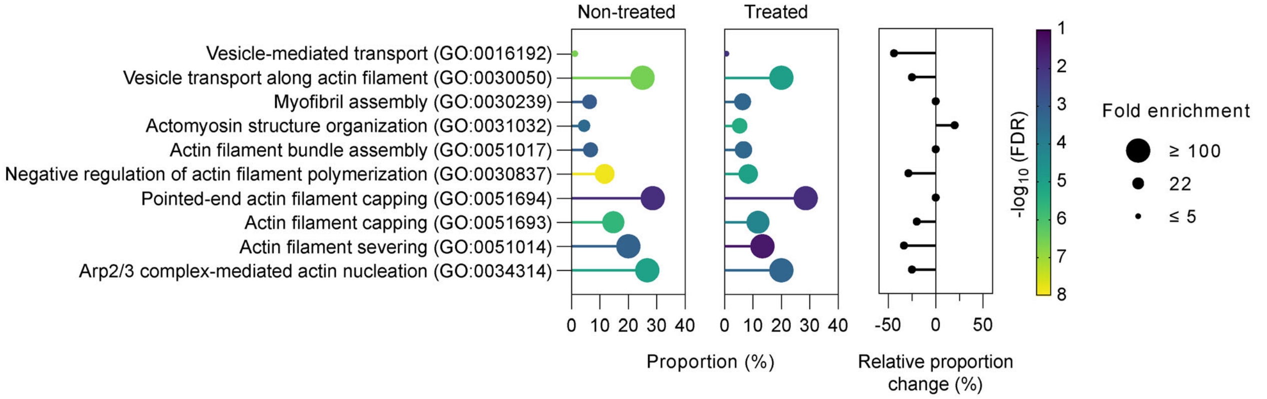

B

GO Biological Process	Ref Count	Proteins
Vesicle-mediated transport (GO:0016192)	1383	Actb, Anxa1, Cdc42, Gsn, Cap1, Myo1b, Myo5a, Myo5b, Myo6 Myh9, Myh10, Gdi2, Rab2a, Rab7a, Rab10, Rab11b, Wasf1
Vesicle transport along actin filament (GO:0030050)	20	Myo1b, Myo1c, Myo5a, Myo5b, Myo6
Myofibril assembly (GO:0030239)	63	Flii, Tmod2, Tmod3, Myh10
Actomyosin structure organization (GO:0031032)	113	Flii, Tmod2, Tmod3, Myh9, Myh10, Cdc42bpa
Actin filament bundle assembly (GO:0051017)	60	Myh10, Lima1, Myo1b, Baiap2
Negative regulation of actin filament polymerization (GO:0030837)	60	Ssh1, Ssh2, Flii, Gsn, Avil, Tmod2, Tmod3
Pointed-end actin filament capping (GO:0051694)	7	Tmod2, Tmod3
Actin filament capping (GO:0051693)	34	Flii, Gsn, Avil, Tmod2, Tmod3
Actin filament severing (GO:0051014)	15	Flii, Gsn, Avil
Arp2/3 complex-mediated actin nucleation (GO:0034314)	15	Arpc1b, Arpc3, Arpc5, Arpc5l

Non-treated only | Treated only | Common

Figure EV5. Gene ontology (GO) biological process term analysis for nontreated and CK-666-treated Eps8 samples.

A Left: Proportion of protein hits for a given GO term. The number of protein hits in the network for a given GO term was normalized by the total number of proteins assigned to a given GO term using the whole mouse genome as a reference (Ref count in B). The graph is coloured by the false discovery rate (FDR). Sizes reflect the fold enrichment of the number proteins in the network divided by the number of proteins expected to be annotated with a given GO term in a randomly generated network of the same size. Right: Relative change in the proportion of proteins in a GO term when comparing CK-666-treated to nontreated Eps8-WT expressing CAD cells.

B Table summarizing mapped proteins to their corresponding GO term for (A). The number of reference proteins in the mouse genome for a given GO term is provided (Ref count). Colour code: Teal, proteins only present in the nontreated (DMSO) Eps8 pull down; Magenta, proteins only present in the CK-666-treated Eps8 pull down; Black, proteins common to both pull-downs.
