# Supplementary figures and images for: Tunnelling nanotube formation is driven by Eps8/IRSp53‐dependent linear actin polymerization (part 1 of 2)
Source: EMBO J. 2023 Nov 27;42(24):e113761. doi: 10.15252/embj.2023113761 (PMC10711657; doi:10.15252/embj.2023113761)

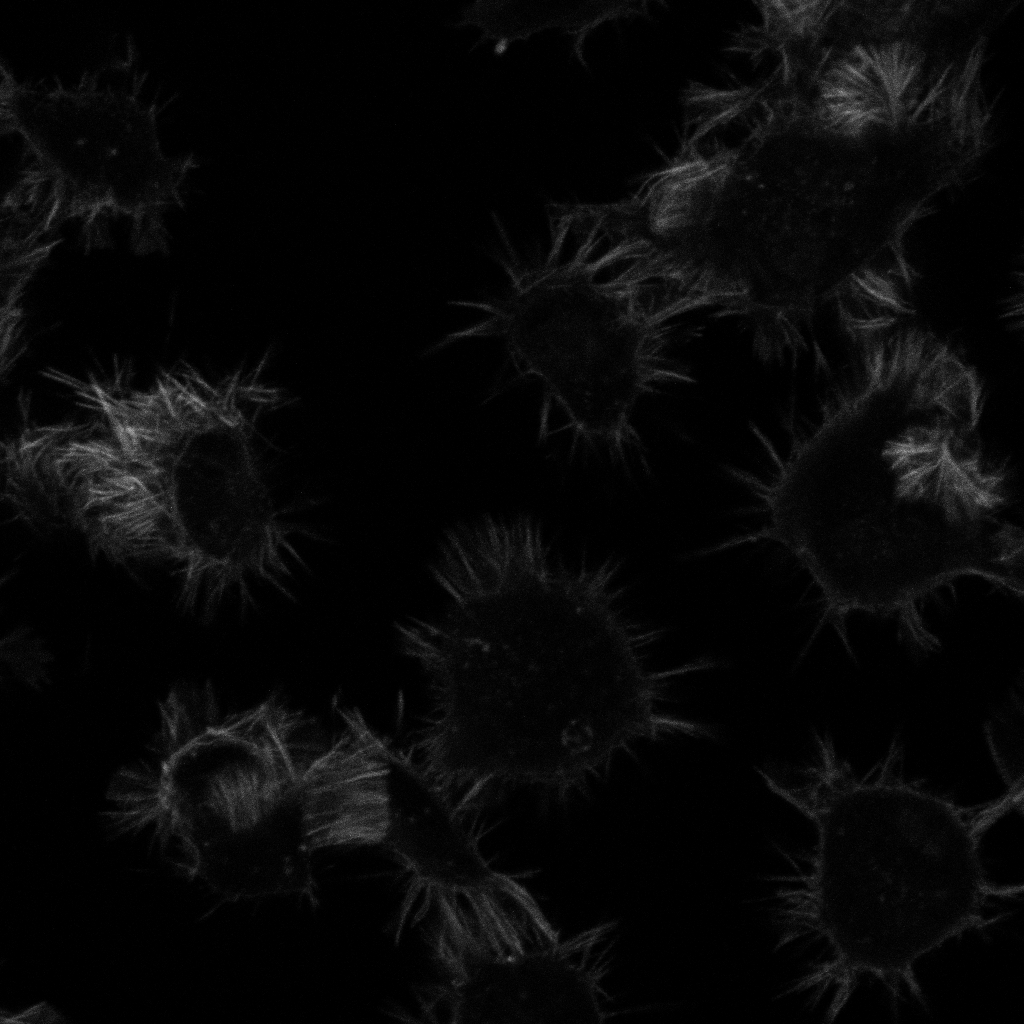

Supplement: Supplementary file 23 — Source Data for Figure 1 [file EMBJ-42-e113761-s010.zip › Figure 1/1C/Surface.tif]

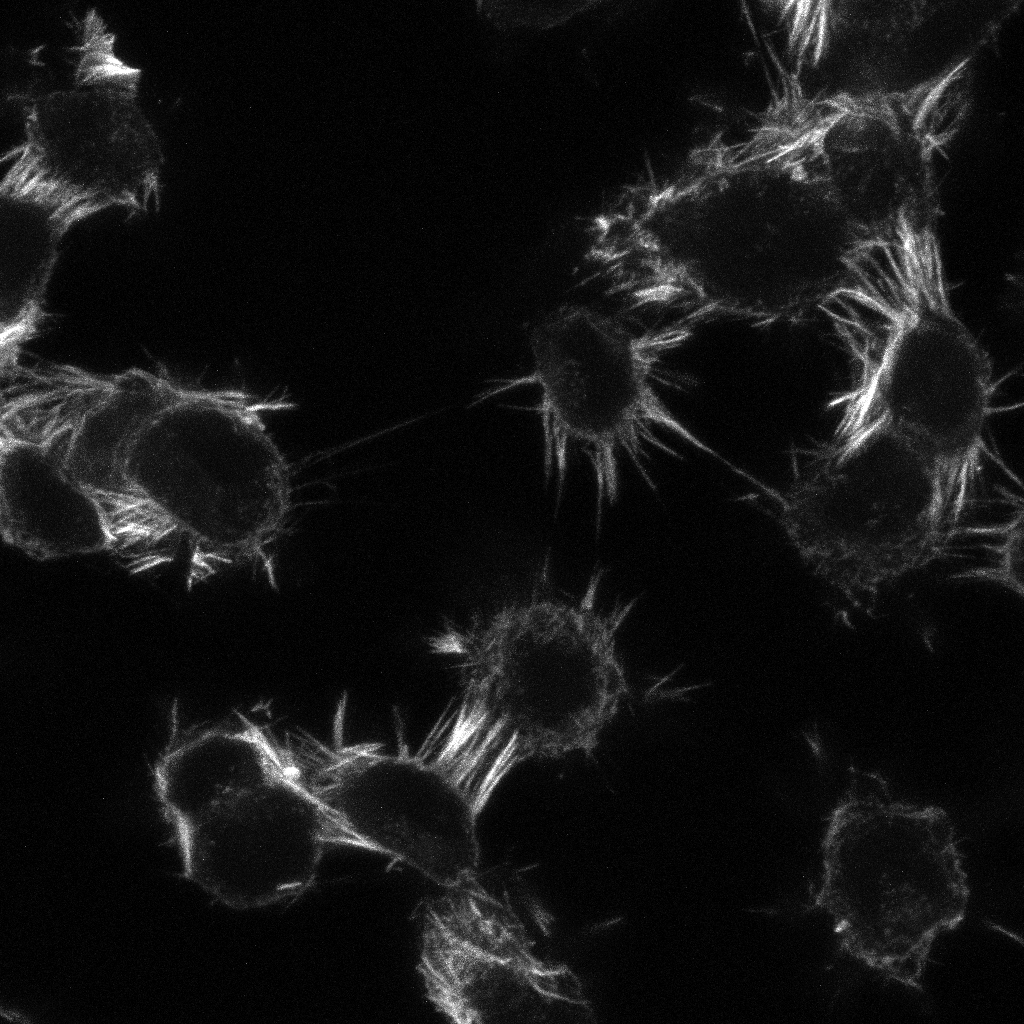

Supplement: Supplementary file 23 — Source Data for Figure 1 [file EMBJ-42-e113761-s010.zip › Figure 1/1C/Upper Stacks.tif]

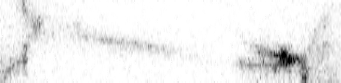

Supplement: Supplementary file 23 — Source Data for Figure 1 [file EMBJ-42-e113761-s010.zip › Figure 1/1C/XZ-i.tif]

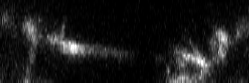

Supplement: Supplementary file 23 — Source Data for Figure 1 [file EMBJ-42-e113761-s010.zip › Figure 1/1C/XZ-ii.tif]

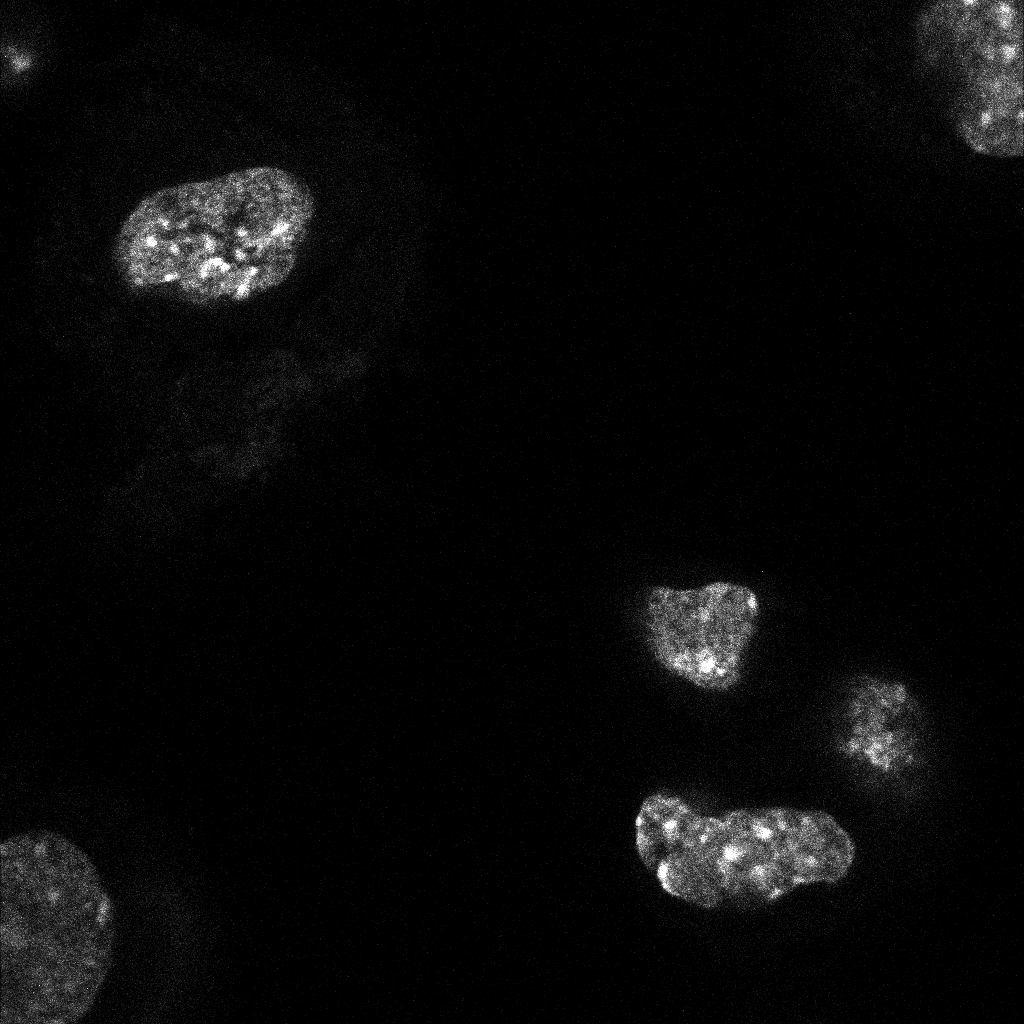

Supplement: Supplementary file 23 — Source Data for Figure 1 [file EMBJ-42-e113761-s010.zip › Figure 1/1D/Surface.tif]

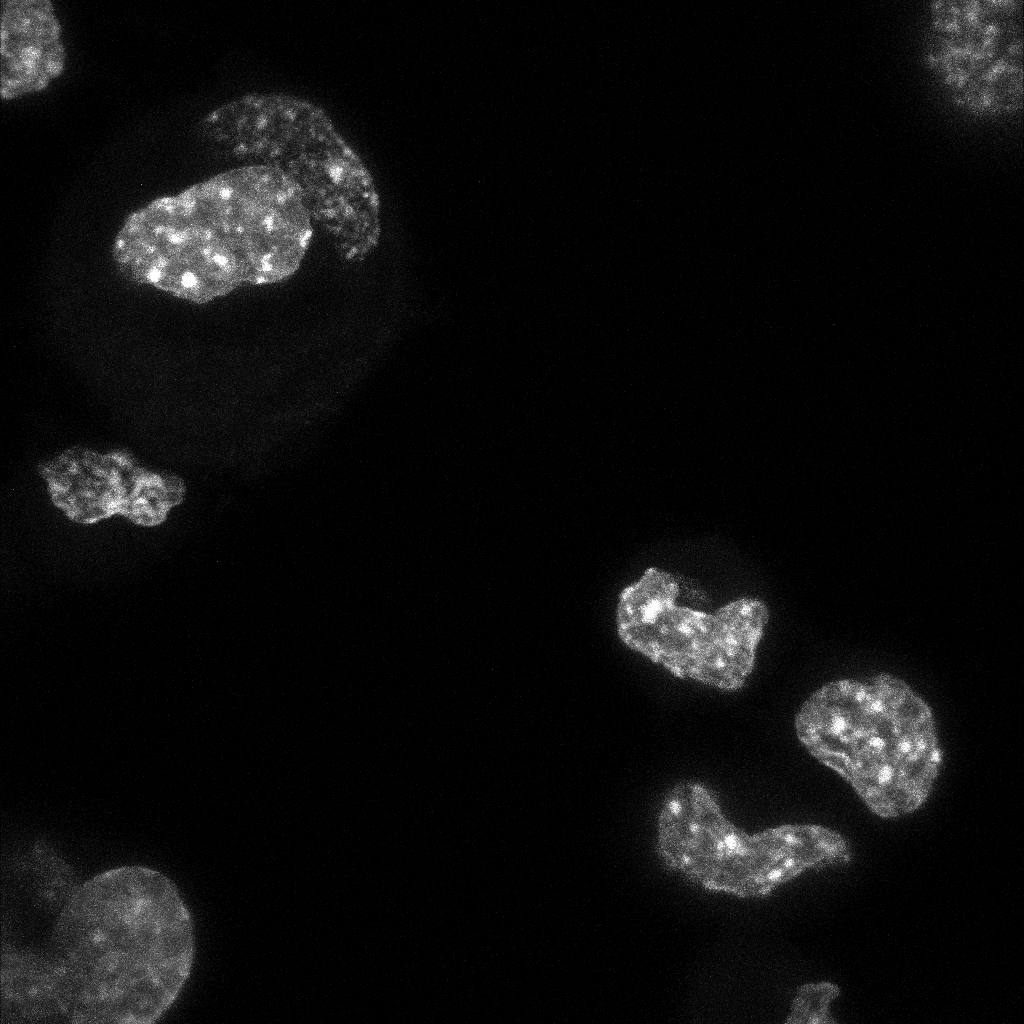

Supplement: Supplementary file 23 — Source Data for Figure 1 [file EMBJ-42-e113761-s010.zip › Figure 1/1D/Upper Stacks.tif]

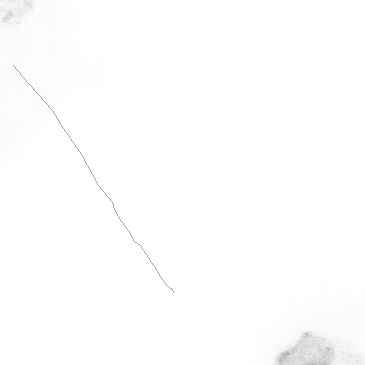

Supplement: Supplementary file 23 — Source Data for Figure 1 [file EMBJ-42-e113761-s010.zip › Figure 1/1D/XY-i.tif]

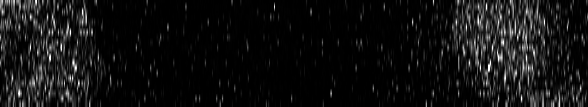

Supplement: Supplementary file 23 — Source Data for Figure 1 [file EMBJ-42-e113761-s010.zip › Figure 1/1D/XZ-i.tif]

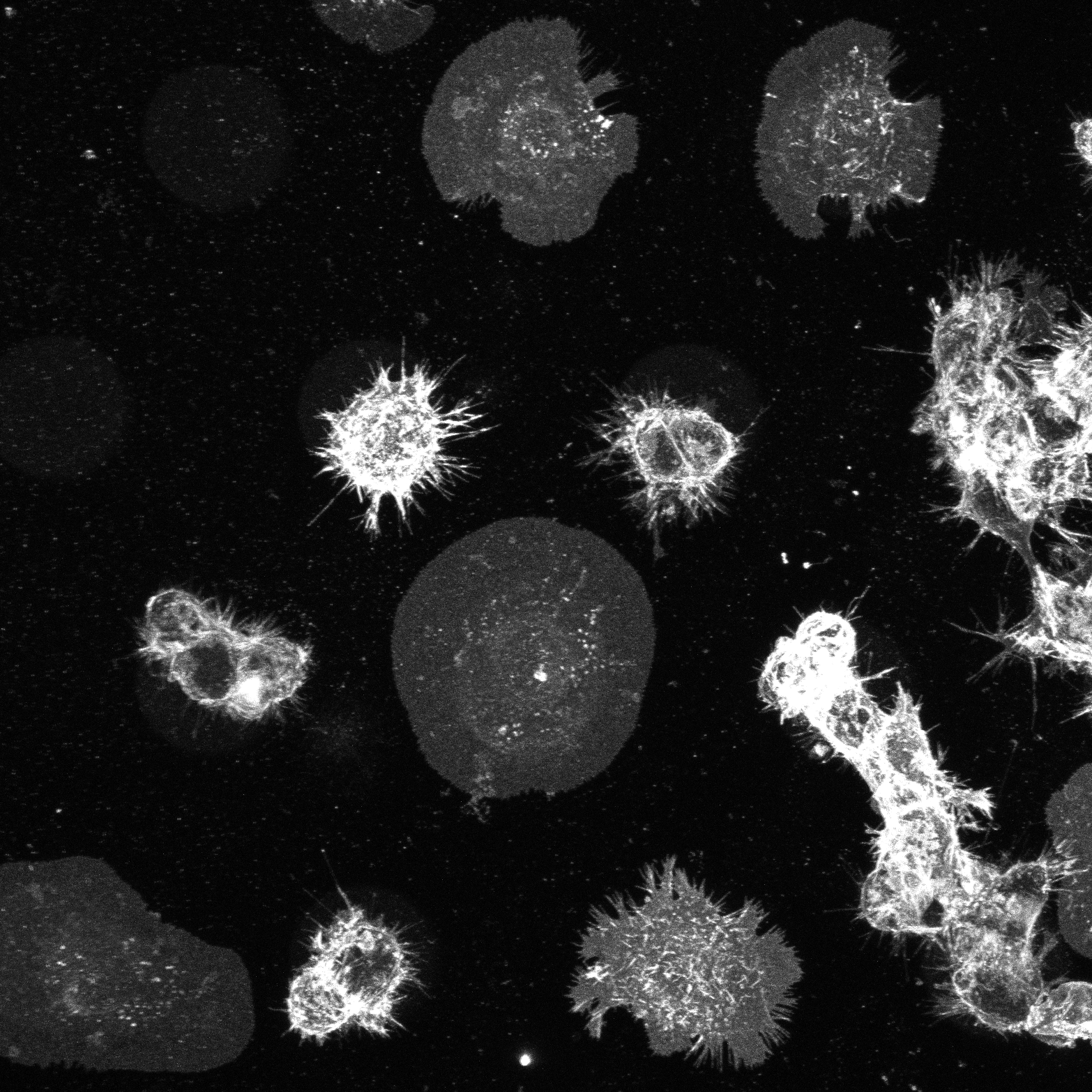

Supplement: Supplementary file 23 — Source Data for Figure 1 [file EMBJ-42-e113761-s010.zip › Figure 1/1E/MAX-D40.tif]

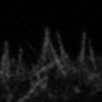

Supplement: Supplementary file 23 — Source Data for Figure 1 [file EMBJ-42-e113761-s010.zip › Figure 1/1G/Images/1.tif]

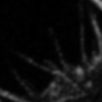

Supplement: Supplementary file 23 — Source Data for Figure 1 [file EMBJ-42-e113761-s010.zip › Figure 1/1G/Images/2.tif]

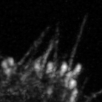

Supplement: Supplementary file 23 — Source Data for Figure 1 [file EMBJ-42-e113761-s010.zip › Figure 1/1G/Images/3.tif]

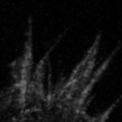

Supplement: Supplementary file 23 — Source Data for Figure 1 [file EMBJ-42-e113761-s010.zip › Figure 1/1G/Images/4.tif]

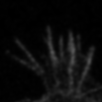

Supplement: Supplementary file 23 — Source Data for Figure 1 [file EMBJ-42-e113761-s010.zip › Figure 1/1G/Images/5.tif]

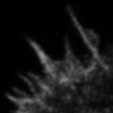

Supplement: Supplementary file 23 — Source Data for Figure 1 [file EMBJ-42-e113761-s010.zip › Figure 1/1G/Images/6.tif]

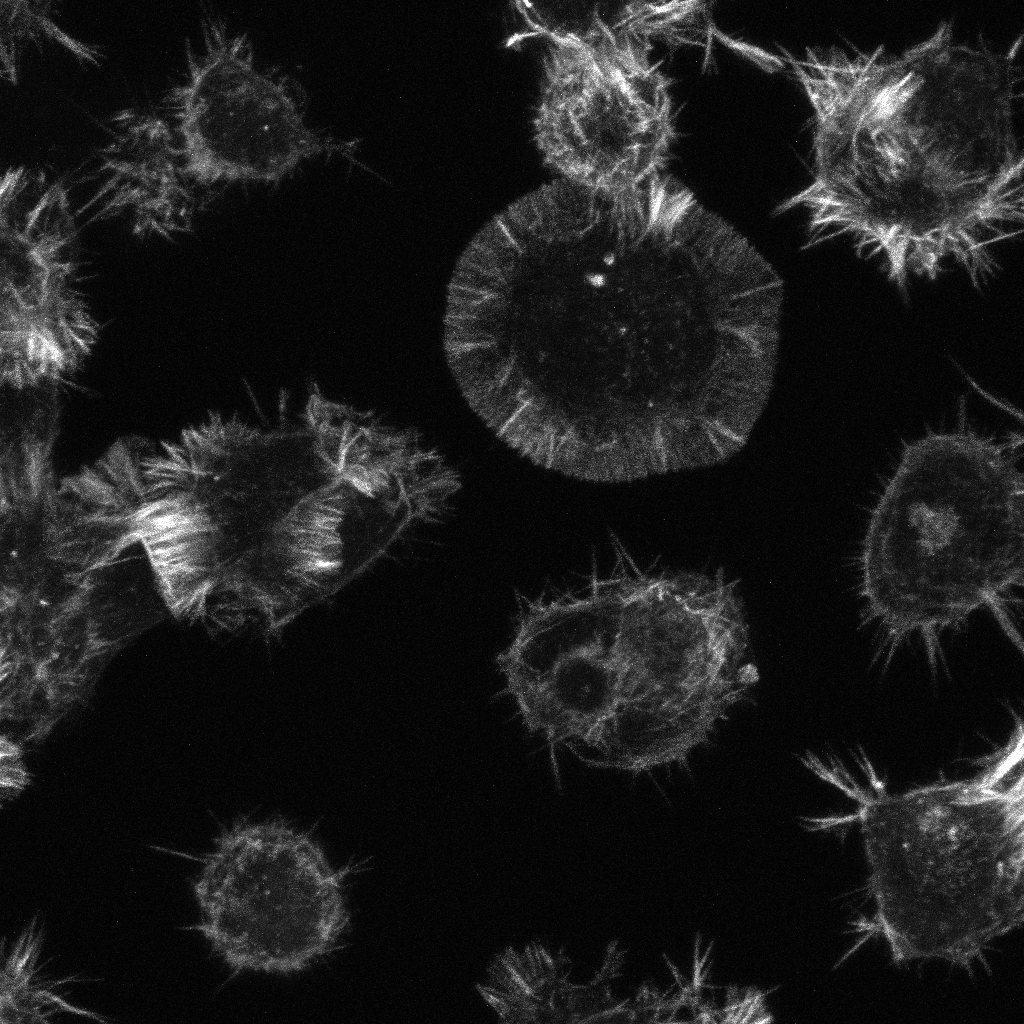

Supplement: Supplementary file 24 — Source Data for Figure 2 [file EMBJ-42-e113761-s017.zip › Figure 2/2A/iii-No Lamellipodia example/MAX.tif]

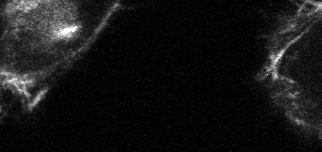

Supplement: Supplementary file 24 — Source Data for Figure 2 [file EMBJ-42-e113761-s017.zip › Figure 2/2A/iii-No Lamellipodia example/XY.tif]

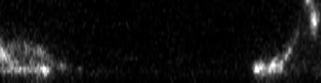

Supplement: Supplementary file 24 — Source Data for Figure 2 [file EMBJ-42-e113761-s017.zip › Figure 2/2A/iii-No Lamellipodia example/XZ.tif]

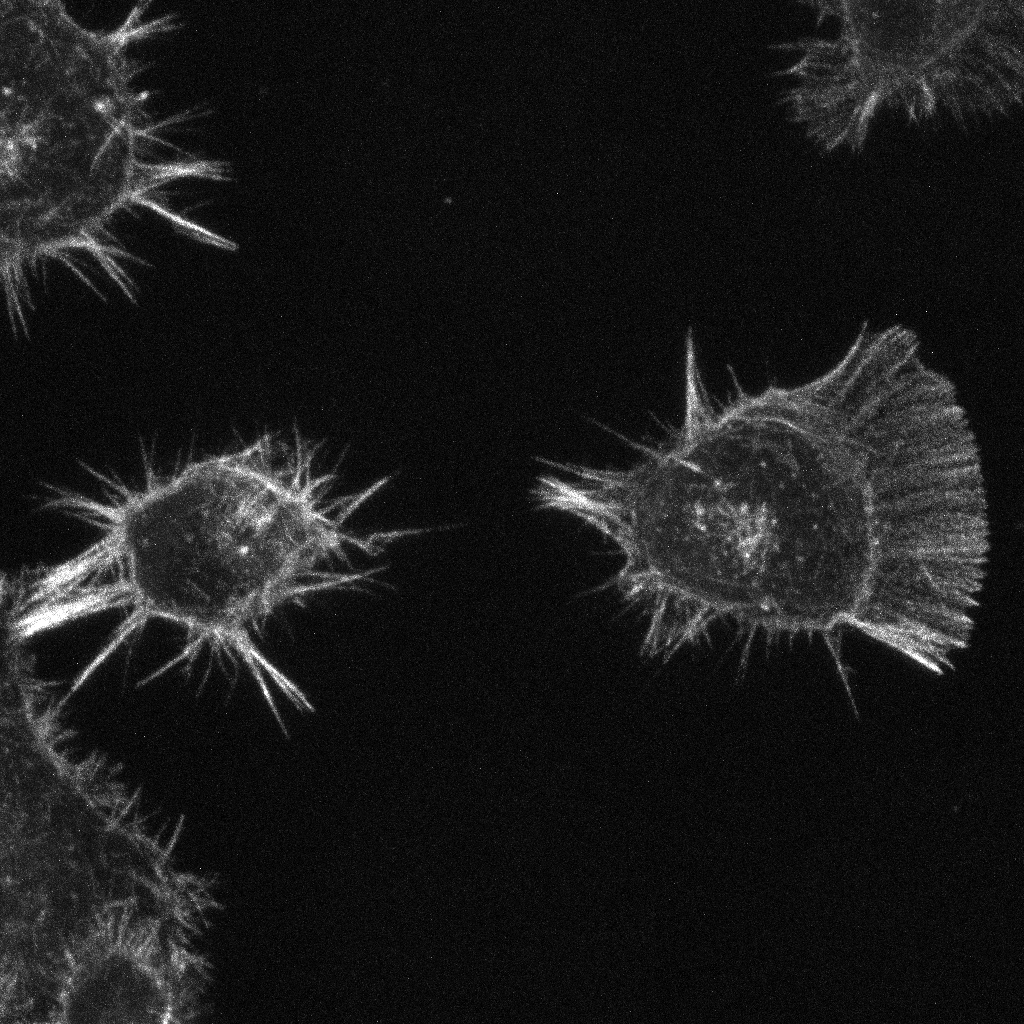

Supplement: Supplementary file 24 — Source Data for Figure 2 [file EMBJ-42-e113761-s017.zip › Figure 2/2A/ii-Mixed examples/1/MAX-1.tif]

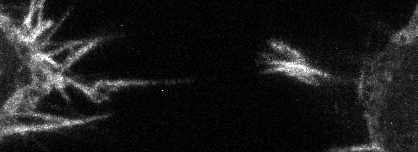

Supplement: Supplementary file 24 — Source Data for Figure 2 [file EMBJ-42-e113761-s017.zip › Figure 2/2A/ii-Mixed examples/1/XY-1.tif]

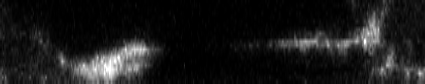

Supplement: Supplementary file 24 — Source Data for Figure 2 [file EMBJ-42-e113761-s017.zip › Figure 2/2A/ii-Mixed examples/1/XZ-1.tif]

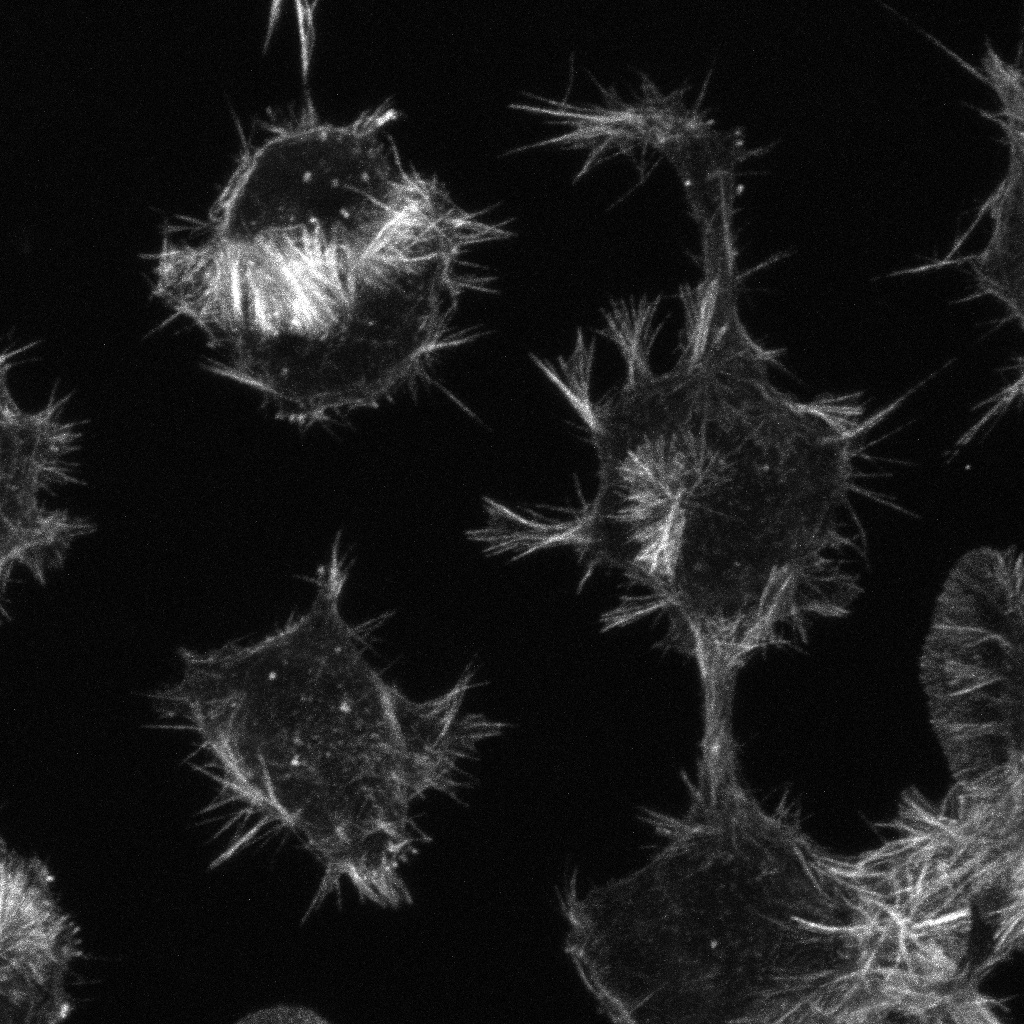

Supplement: Supplementary file 24 — Source Data for Figure 2 [file EMBJ-42-e113761-s017.zip › Figure 2/2A/ii-Mixed examples/2/MAX-2.tif]

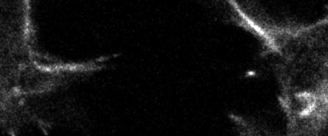

Supplement: Supplementary file 24 — Source Data for Figure 2 [file EMBJ-42-e113761-s017.zip › Figure 2/2A/ii-Mixed examples/2/XY-2.tif]

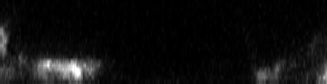

Supplement: Supplementary file 24 — Source Data for Figure 2 [file EMBJ-42-e113761-s017.zip › Figure 2/2A/ii-Mixed examples/2/XZ-2.tif]

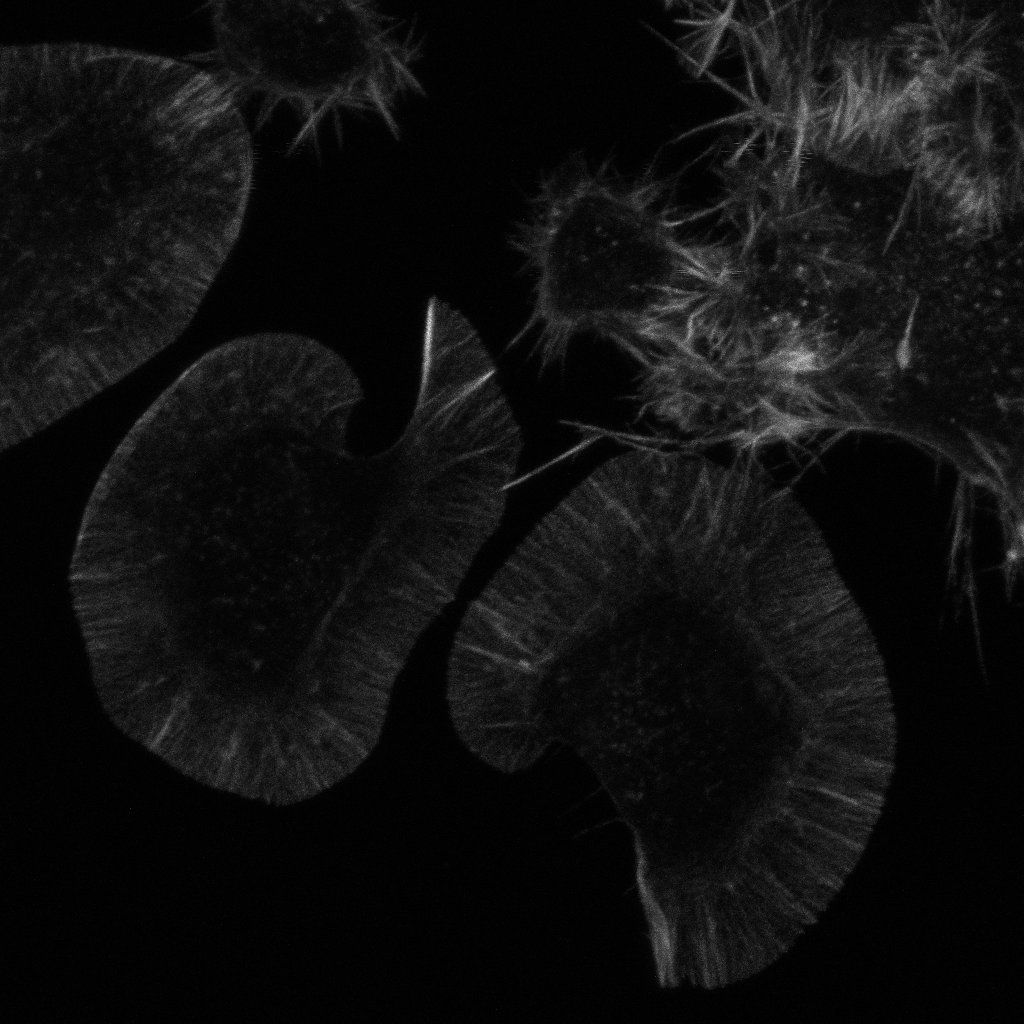

Supplement: Supplementary file 24 — Source Data for Figure 2 [file EMBJ-42-e113761-s017.zip › Figure 2/2A/i-Lamellipodia example/MAX.tif]

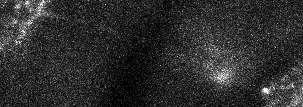

Supplement: Supplementary file 24 — Source Data for Figure 2 [file EMBJ-42-e113761-s017.zip › Figure 2/2A/i-Lamellipodia example/XY.tif]

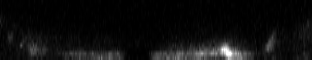

Supplement: Supplementary file 24 — Source Data for Figure 2 [file EMBJ-42-e113761-s017.zip › Figure 2/2A/i-Lamellipodia example/XZ.tif]

# D30 Experiments

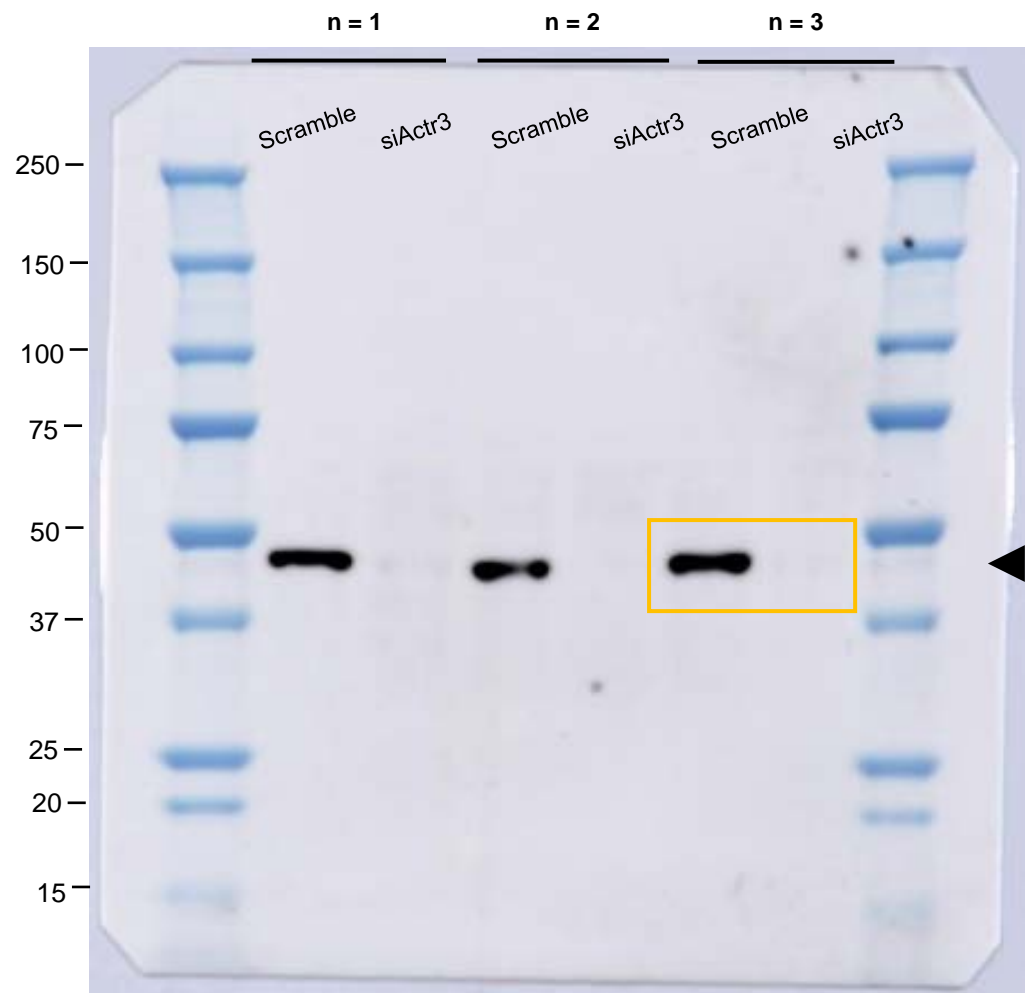

**Actr3 (Arp3)**  
Expected at 47 kDa

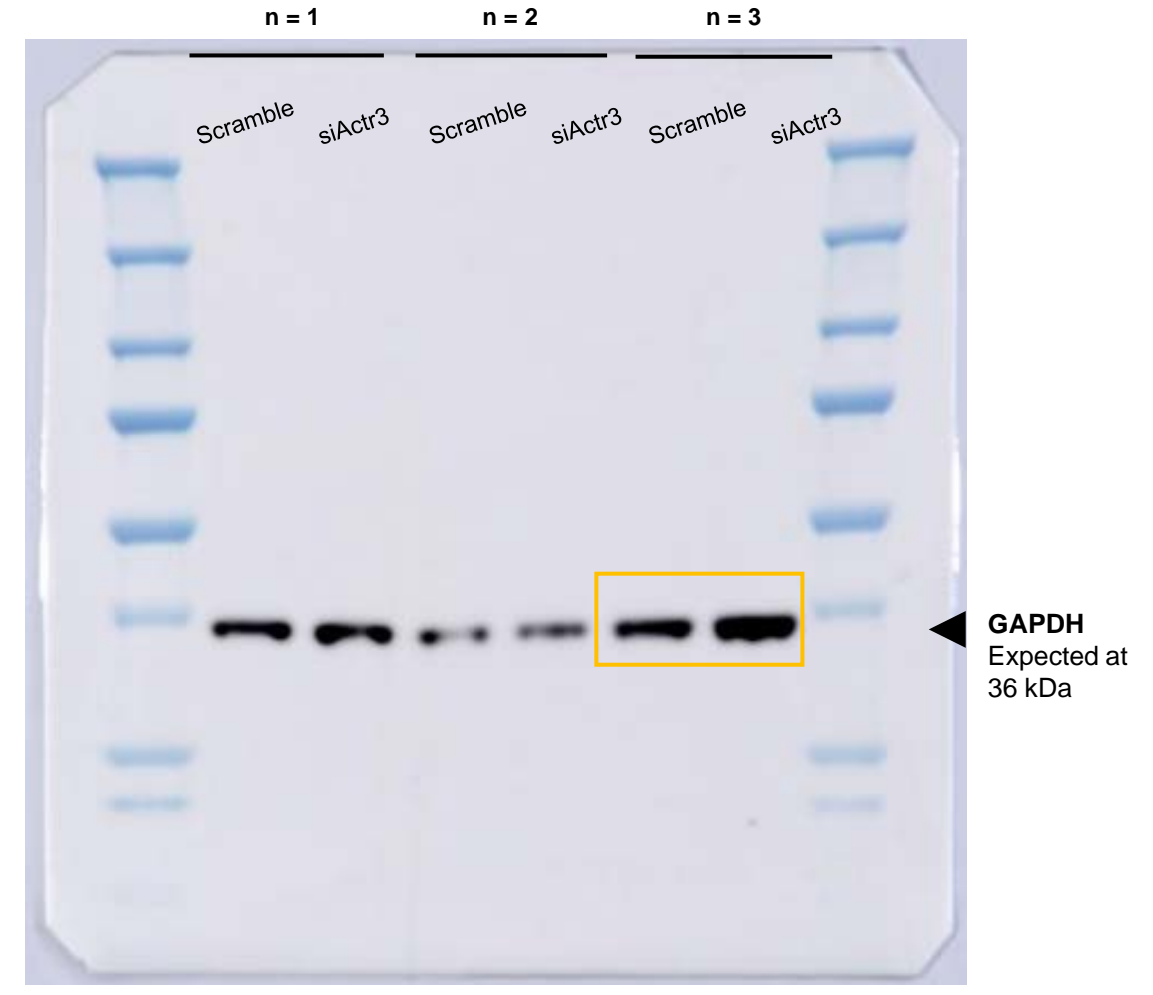

**GAPDH**  
Expected at 36 kDa

Supplement: Supplementary file 24 — Source Data for Figure 2 [file EMBJ-42-e113761-s017.zip › Figure 2/2D/WB/Annotated blot.pdf]

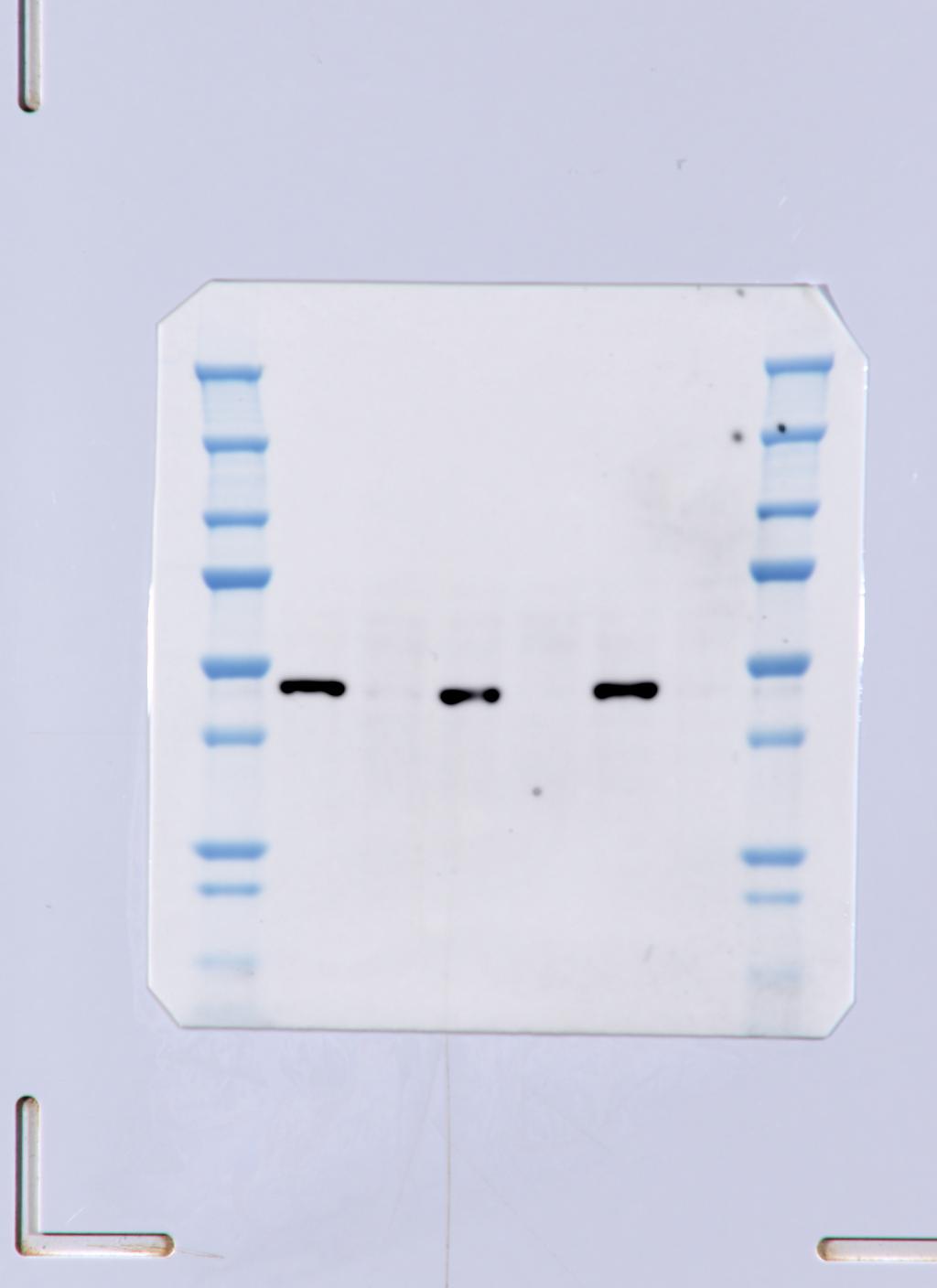

Supplement: Supplementary file 24 — Source Data for Figure 2 [file EMBJ-42-e113761-s017.zip › Figure 2/2D/WB/Ch+Ladder/Actr3-2min-8x-Ch+Ladder.jpg]

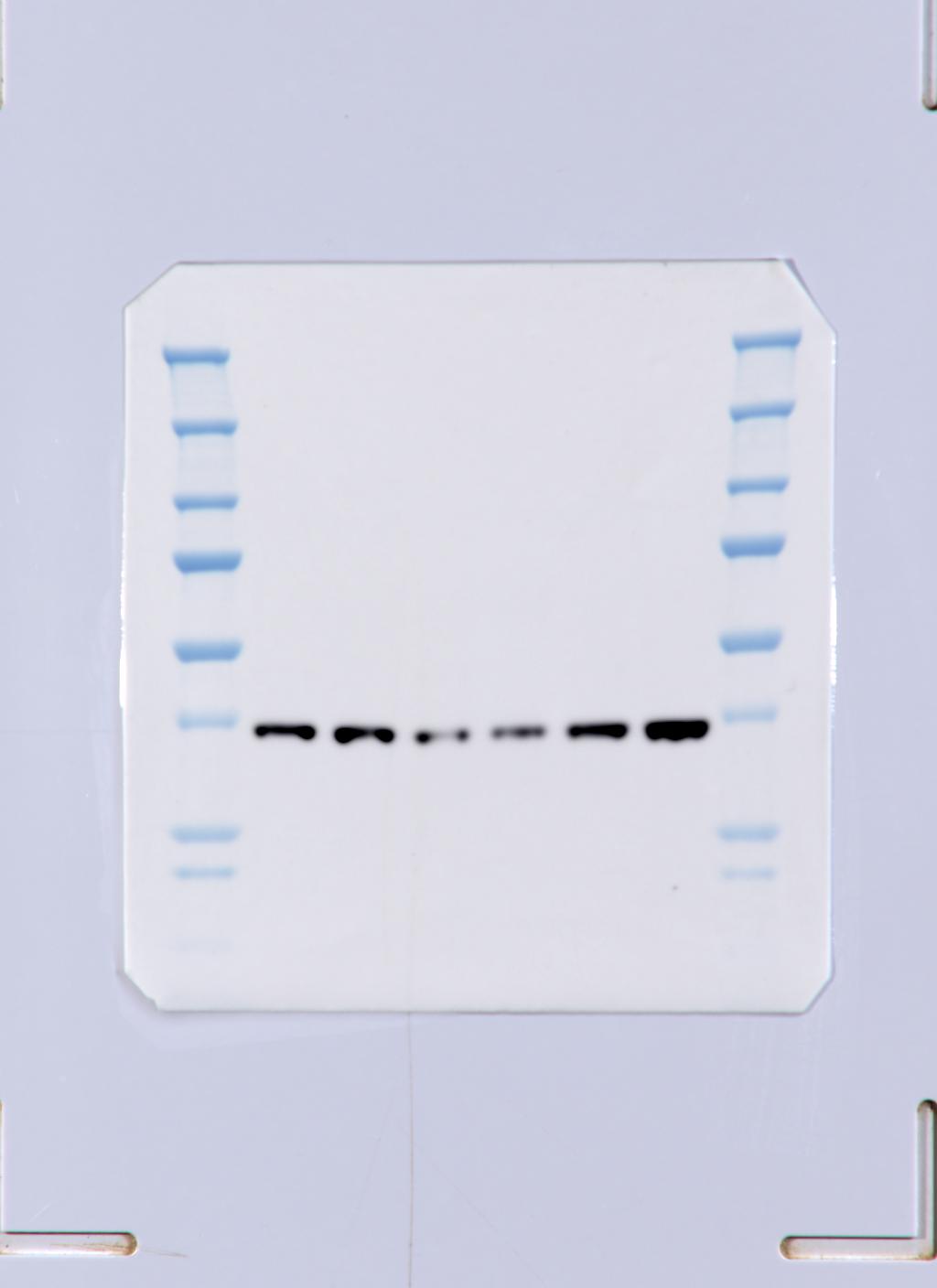

Supplement: Supplementary file 24 — Source Data for Figure 2 [file EMBJ-42-e113761-s017.zip › Figure 2/2D/WB/Ch+Ladder/GAPDH-54sec-8x-Ch+Marker.jpg]

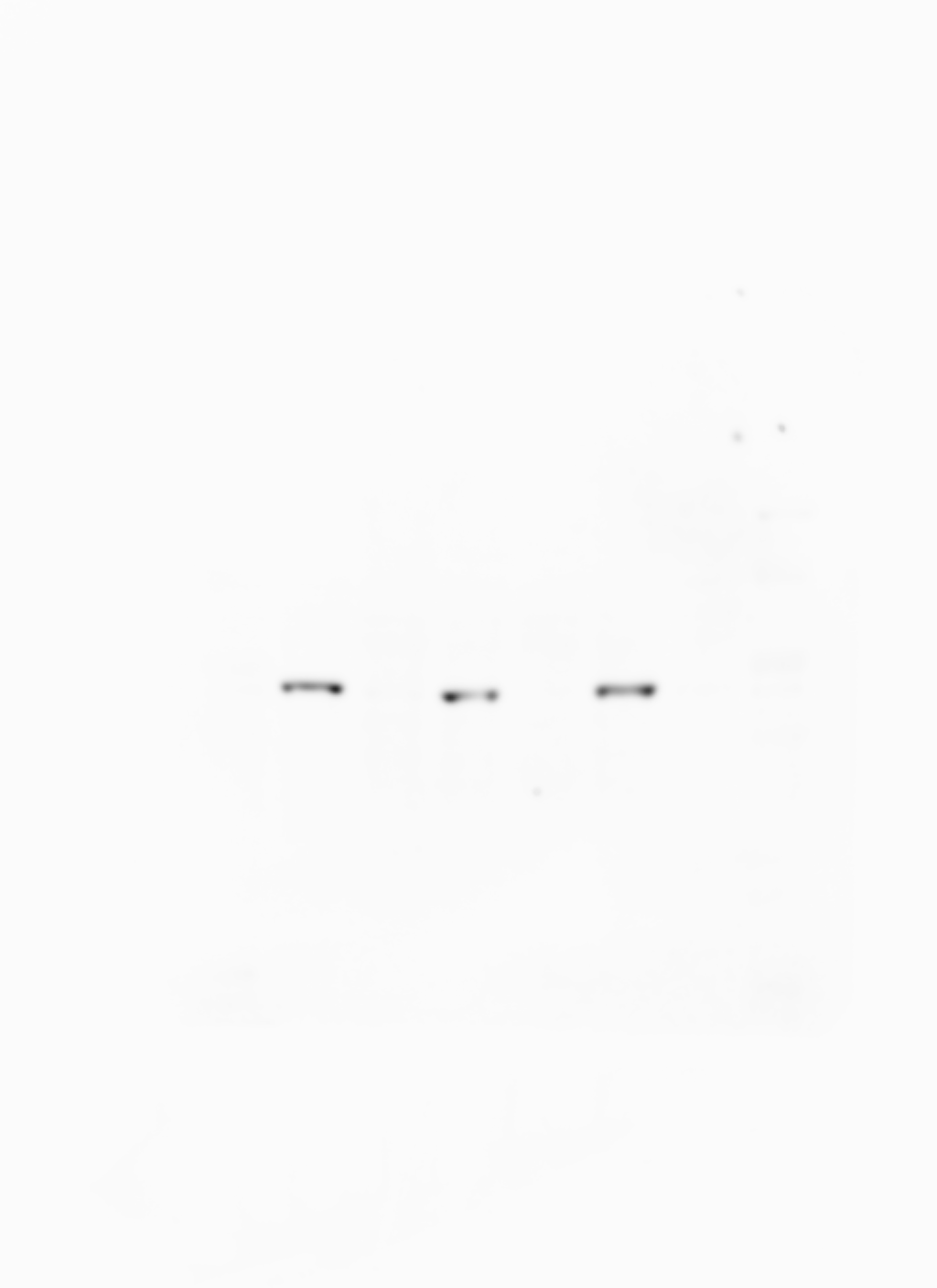

Supplement: Supplementary file 24 — Source Data for Figure 2 [file EMBJ-42-e113761-s017.zip › Figure 2/2D/WB/Raw TIFs/Actr3.tif]

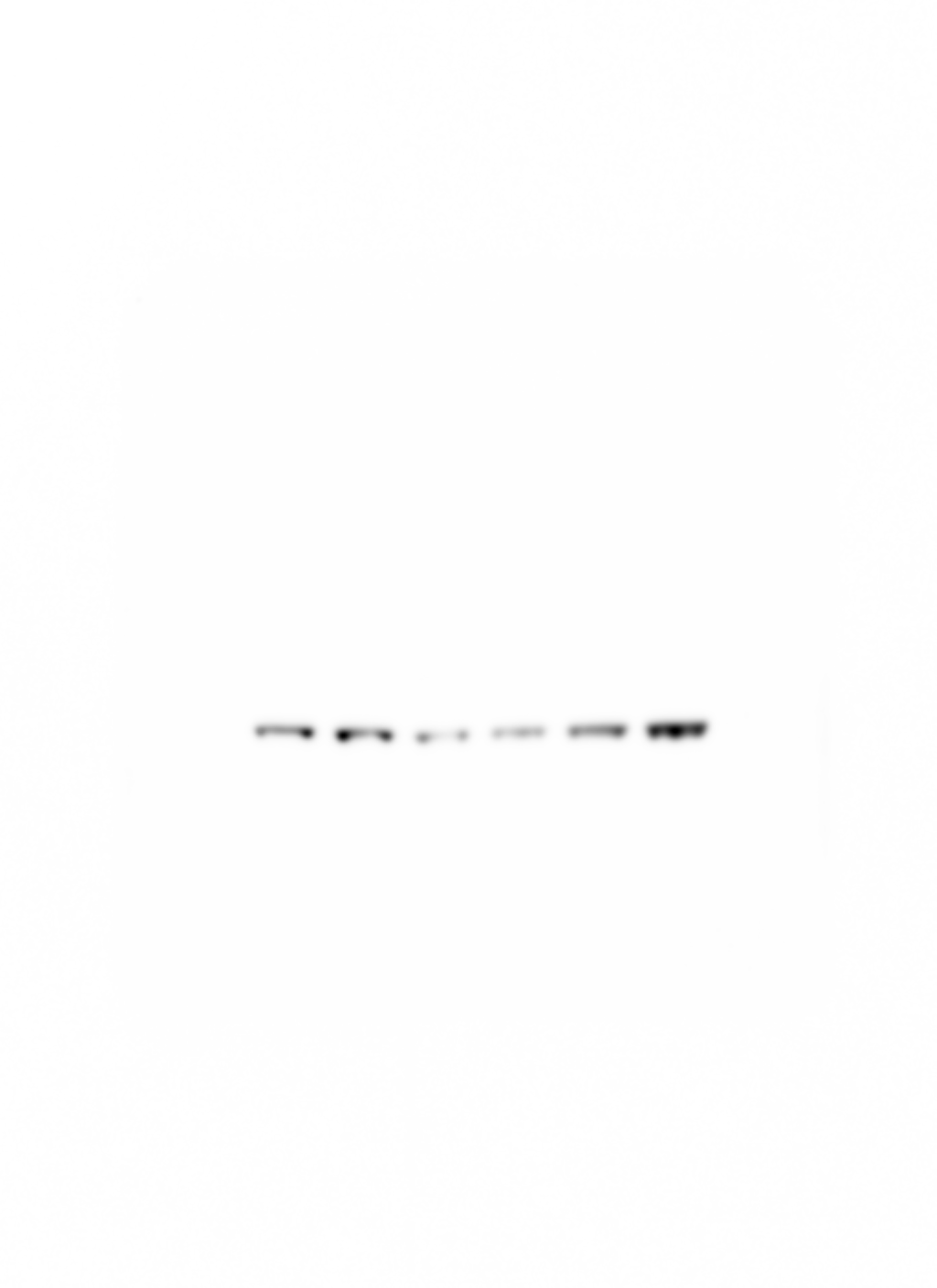

Supplement: Supplementary file 24 — Source Data for Figure 2 [file EMBJ-42-e113761-s017.zip › Figure 2/2D/WB/Raw TIFs/GAPDH.tif]

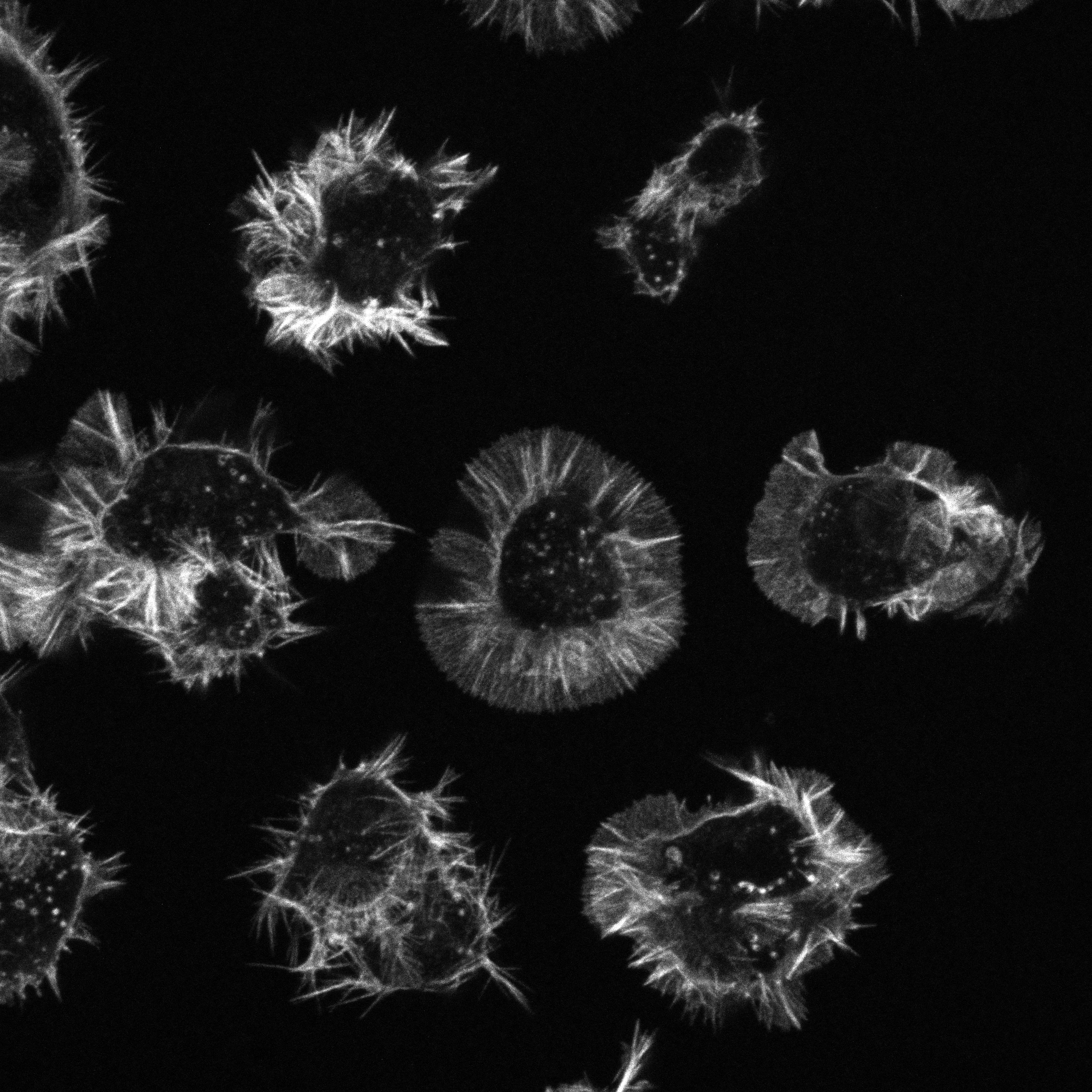

Supplement: Supplementary file 24 — Source Data for Figure 2 [file EMBJ-42-e113761-s017.zip › Figure 2/2E/Surface.tif]

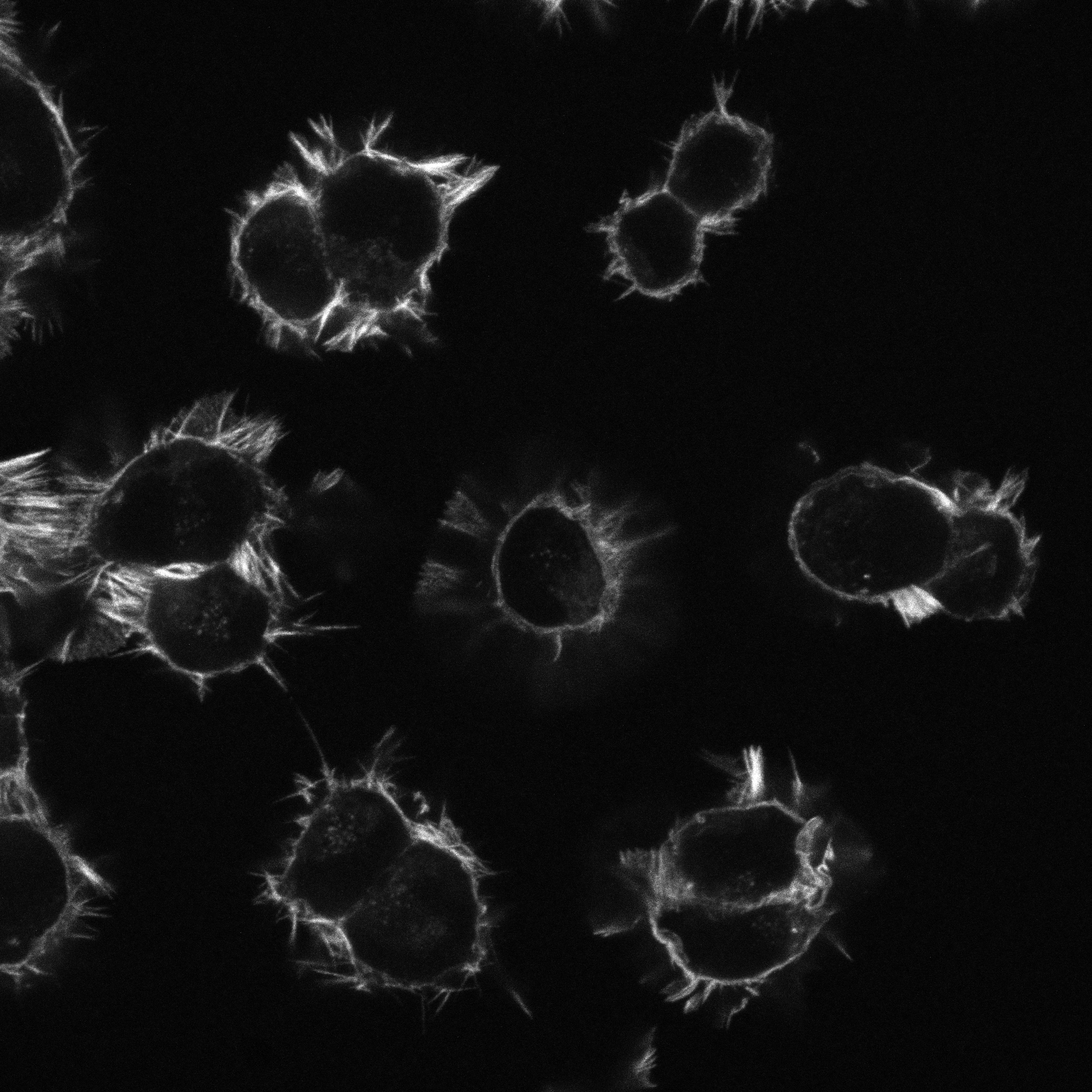

Supplement: Supplementary file 24 — Source Data for Figure 2 [file EMBJ-42-e113761-s017.zip › Figure 2/2E/Upper stacks.tif]

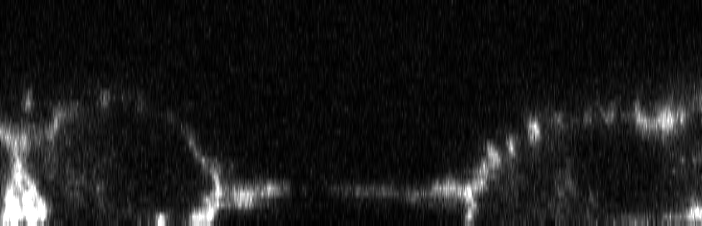

Supplement: Supplementary file 24 — Source Data for Figure 2 [file EMBJ-42-e113761-s017.zip › Figure 2/2E/XZ.tif]

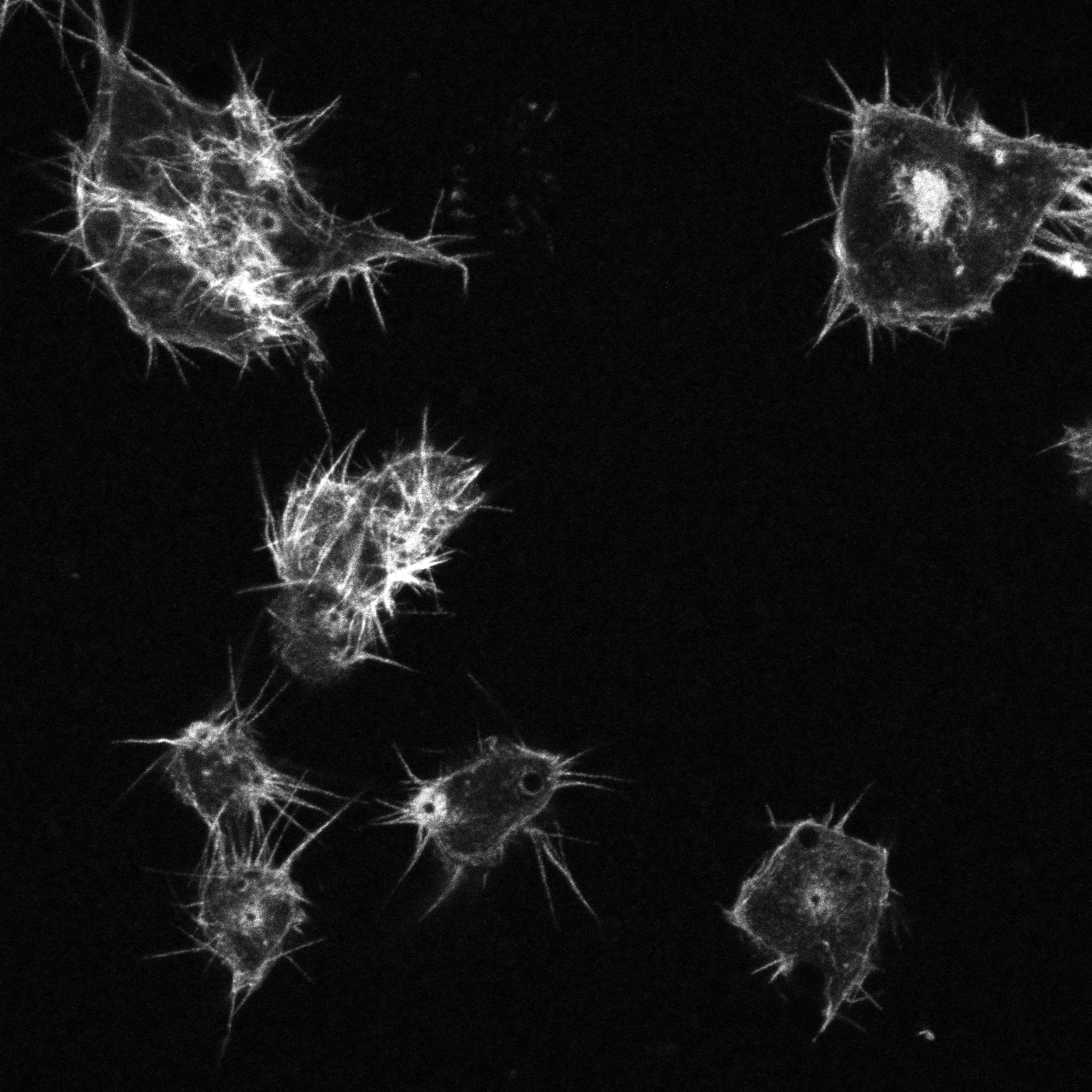

Supplement: Supplementary file 24 — Source Data for Figure 2 [file EMBJ-42-e113761-s017.zip › Figure 2/2F/Surface.tif]

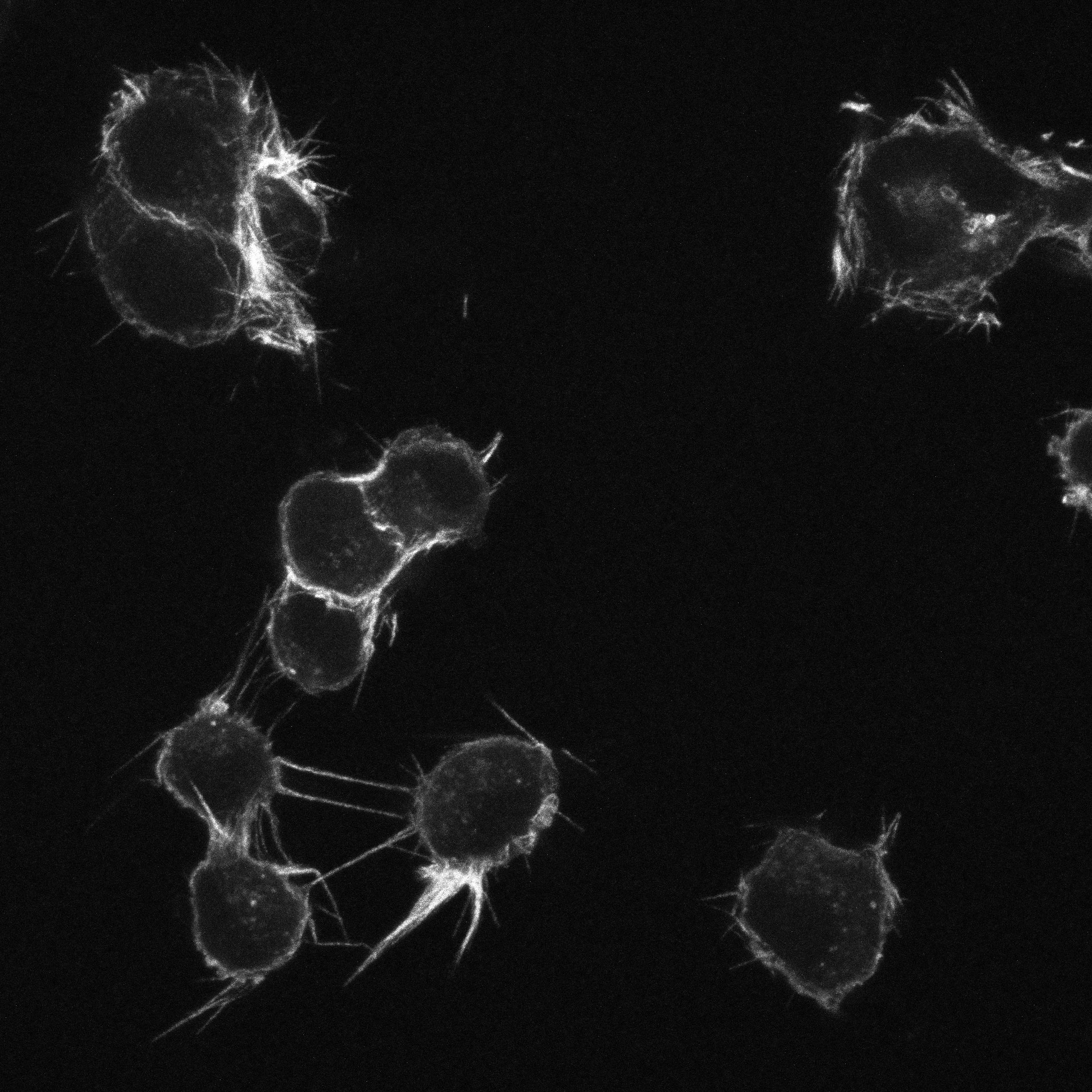

Supplement: Supplementary file 24 — Source Data for Figure 2 [file EMBJ-42-e113761-s017.zip › Figure 2/2F/Upper Stacks.tif]

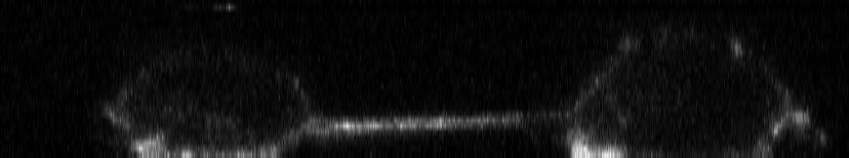

Supplement: Supplementary file 24 — Source Data for Figure 2 [file EMBJ-42-e113761-s017.zip › Figure 2/2F/XZ-i.tif]

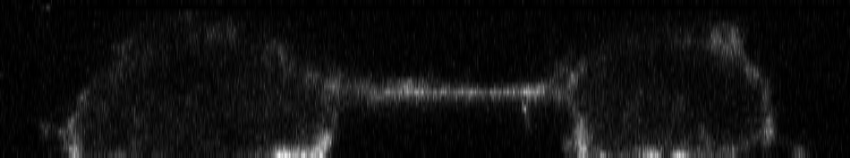

Supplement: Supplementary file 24 — Source Data for Figure 2 [file EMBJ-42-e113761-s017.zip › Figure 2/2F/XZ-ii.tif]

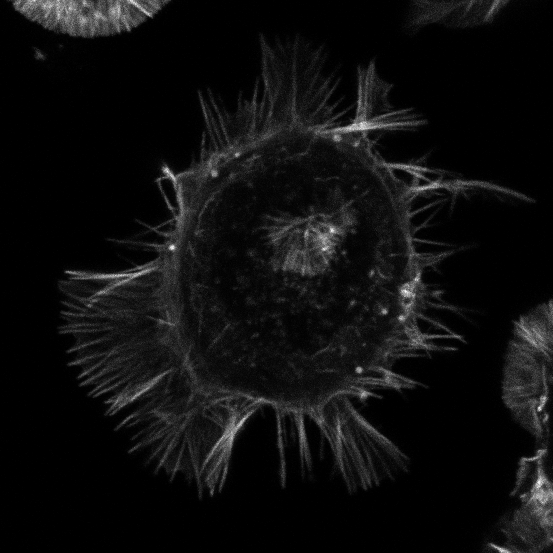

Supplement: Supplementary file 24 — Source Data for Figure 2 [file EMBJ-42-e113761-s017.zip › Figure 2/2H/Inset.tif]

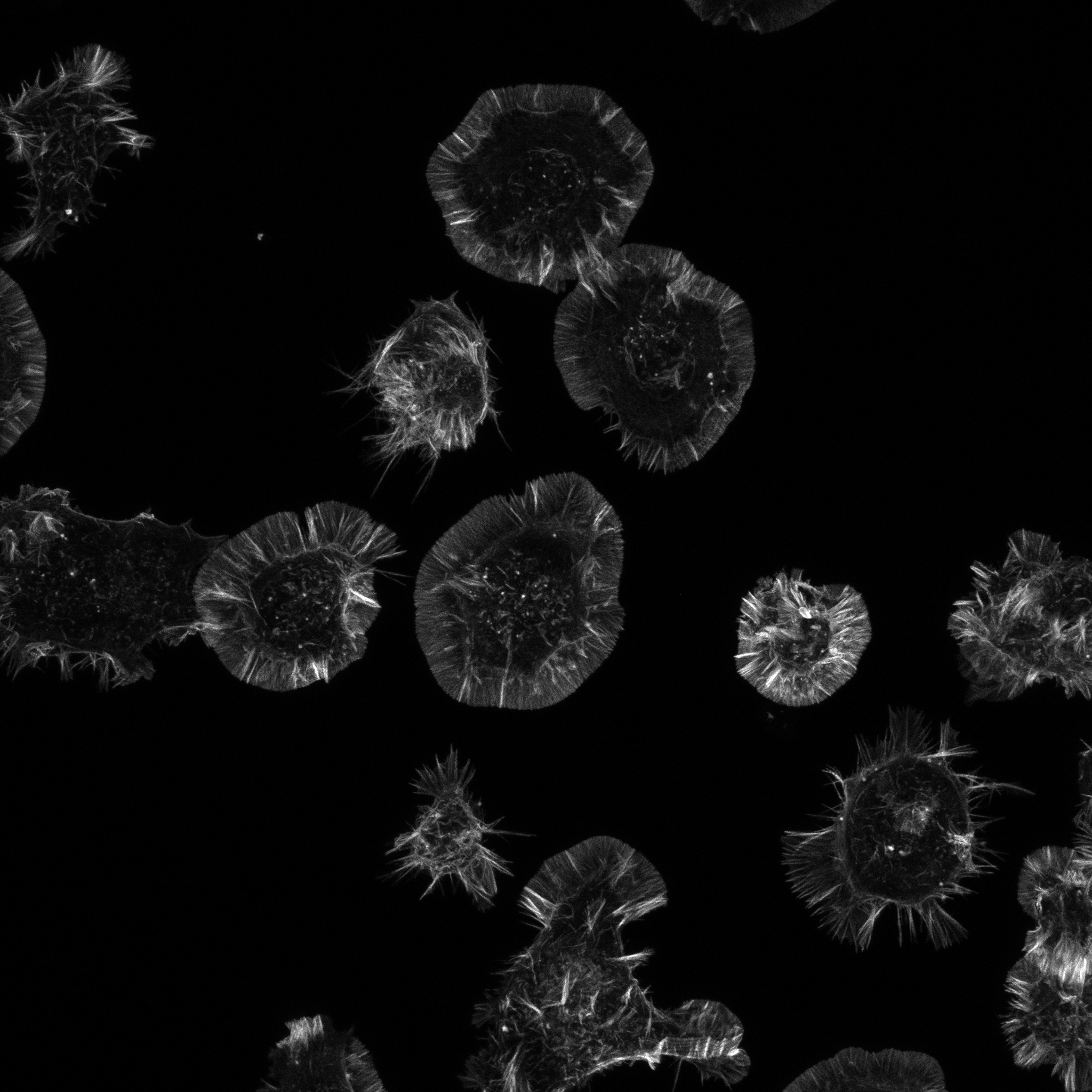

Supplement: Supplementary file 24 — Source Data for Figure 2 [file EMBJ-42-e113761-s017.zip › Figure 2/2H/MAX.tif]

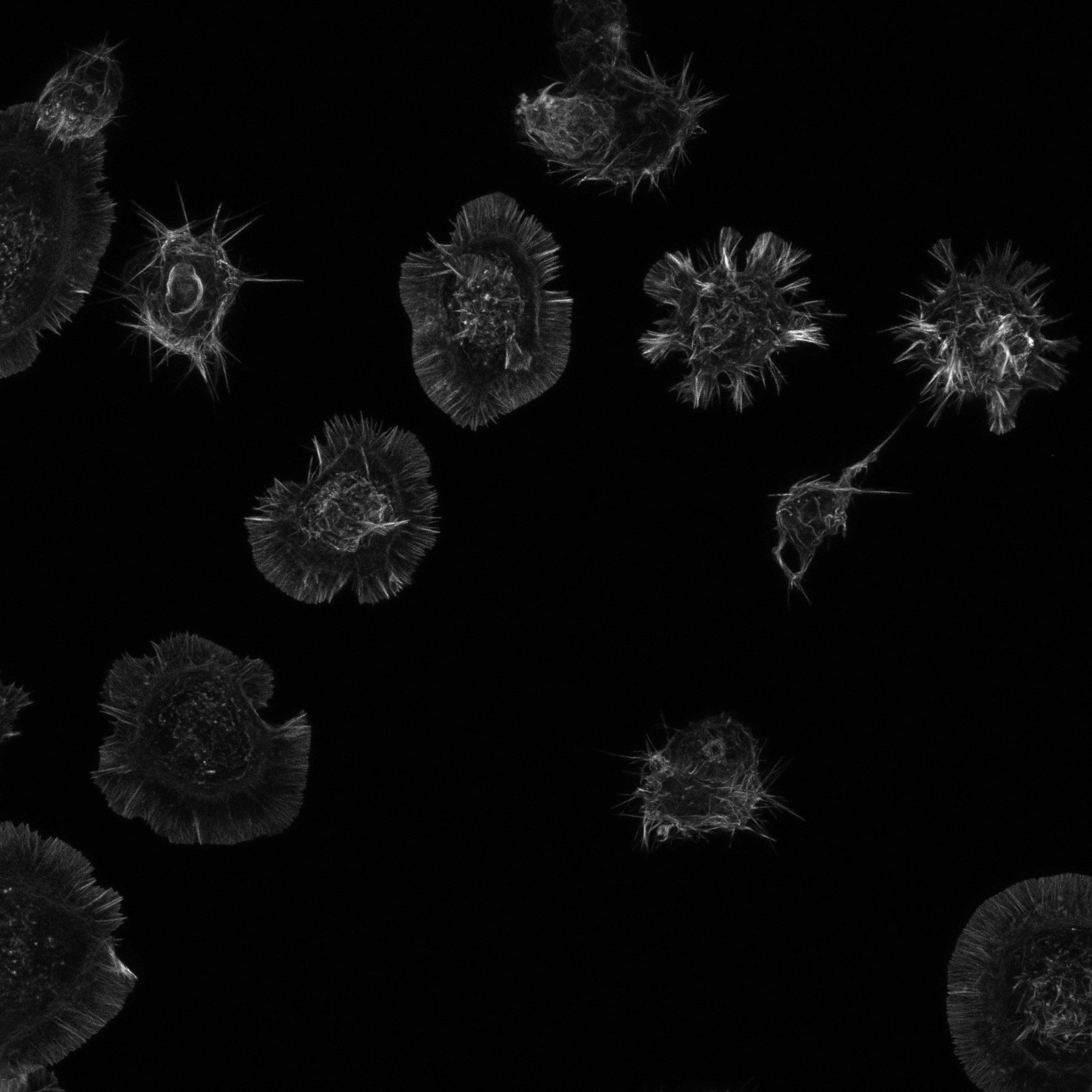

Supplement: Supplementary file 24 — Source Data for Figure 2 [file EMBJ-42-e113761-s017.zip › Figure 2/2I/Full MAX_siActr3 Co-culture_2.tif]

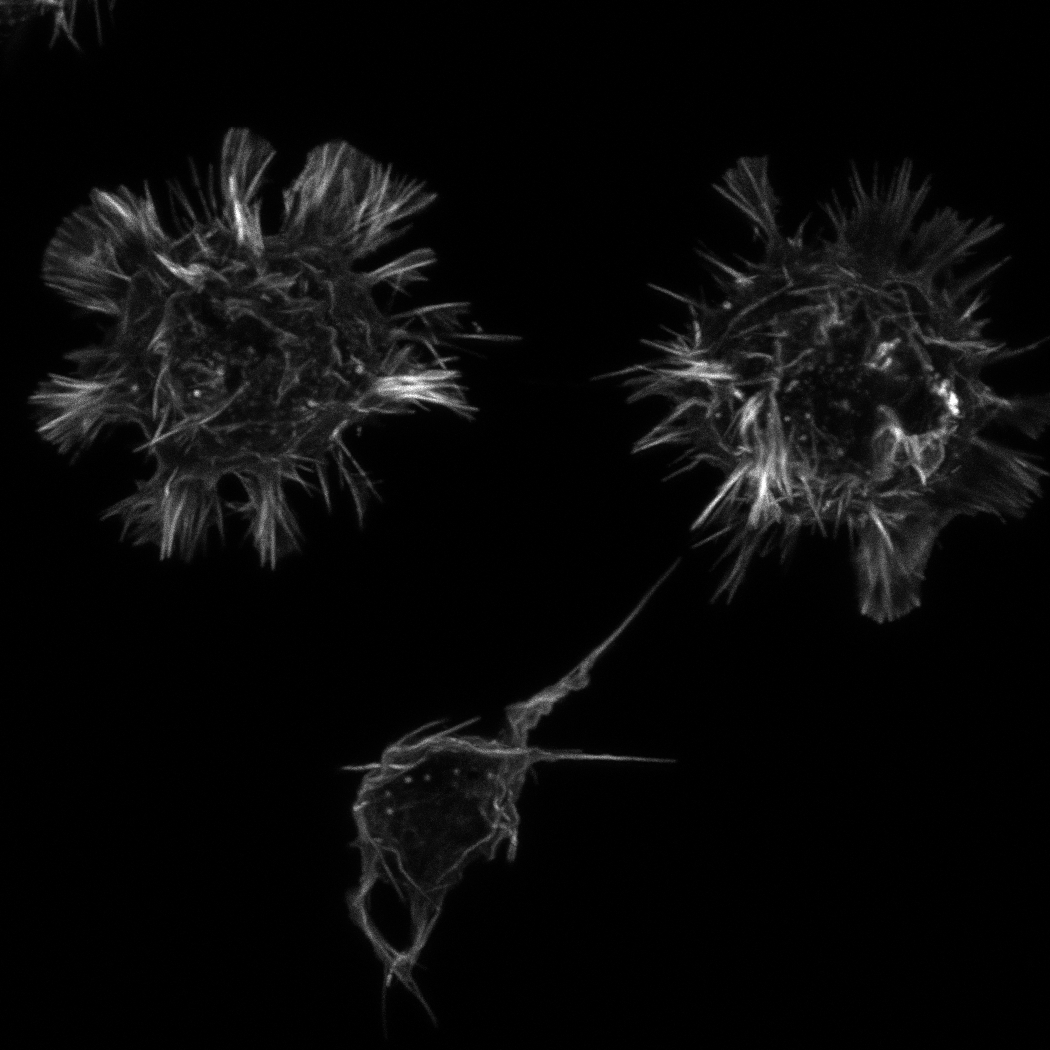

Supplement: Supplementary file 24 — Source Data for Figure 2 [file EMBJ-42-e113761-s017.zip › Figure 2/2I/i/Zoom-In.tif]

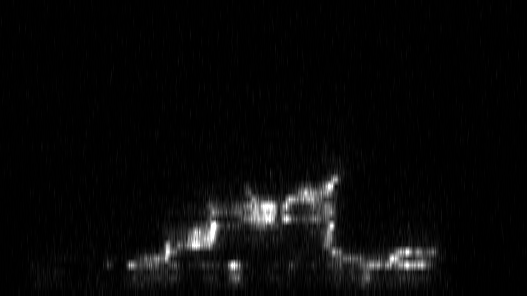

Supplement: Supplementary file 24 — Source Data for Figure 2 [file EMBJ-42-e113761-s017.zip › Figure 2/2I/ii/XZ.tif]

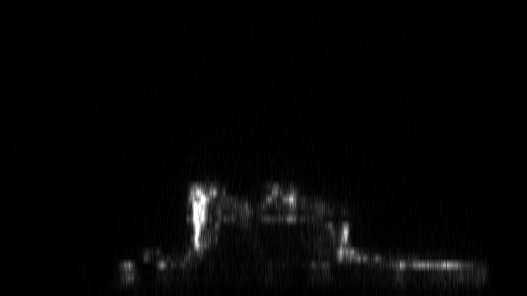

Supplement: Supplementary file 24 — Source Data for Figure 2 [file EMBJ-42-e113761-s017.zip › Figure 2/2I/ii/YZ.tif]

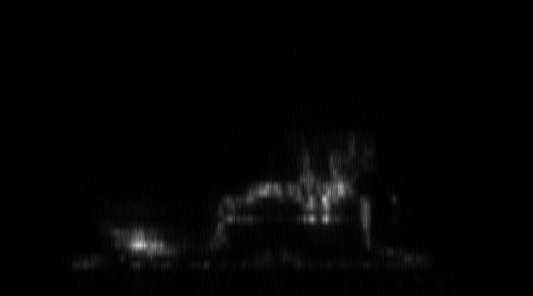

Supplement: Supplementary file 24 — Source Data for Figure 2 [file EMBJ-42-e113761-s017.zip › Figure 2/2I/iii/XZ.tif]

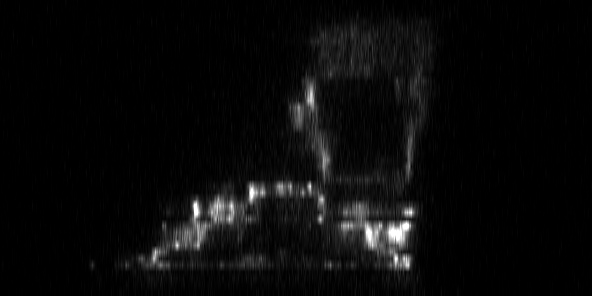

Supplement: Supplementary file 24 — Source Data for Figure 2 [file EMBJ-42-e113761-s017.zip › Figure 2/2I/iii/YZ.tif]

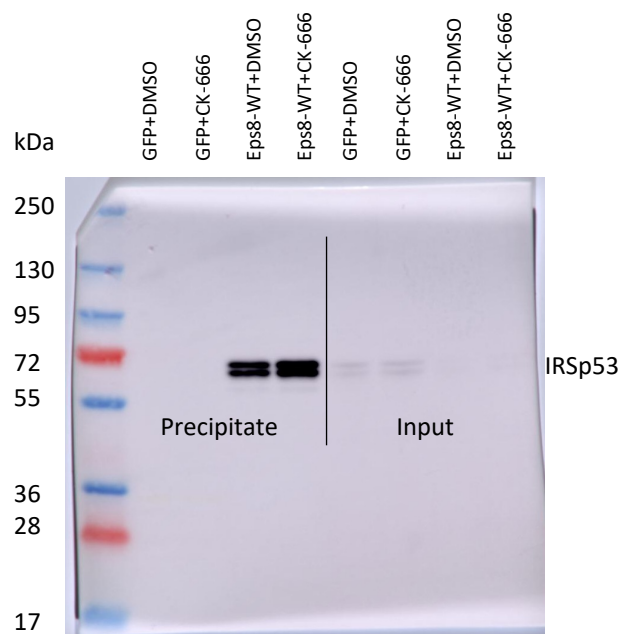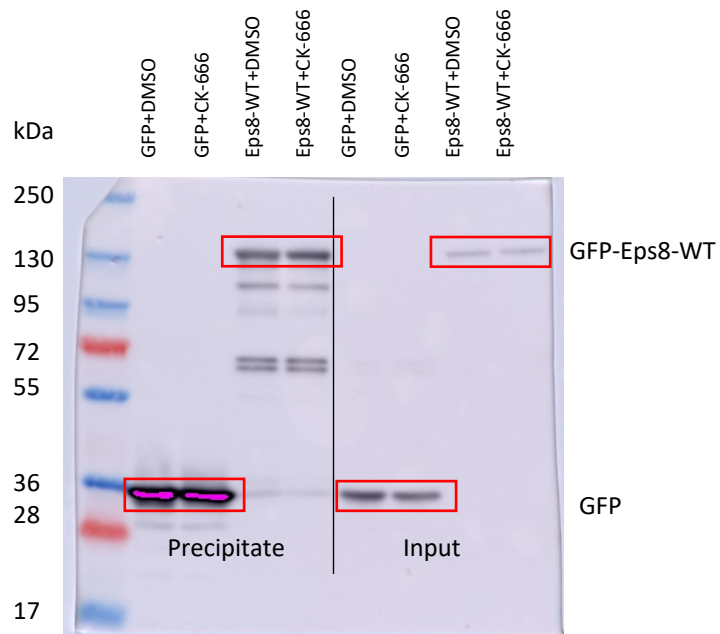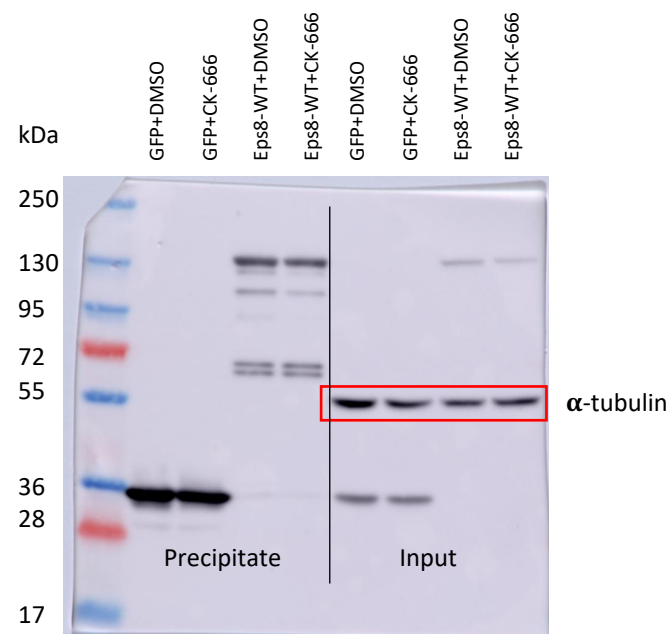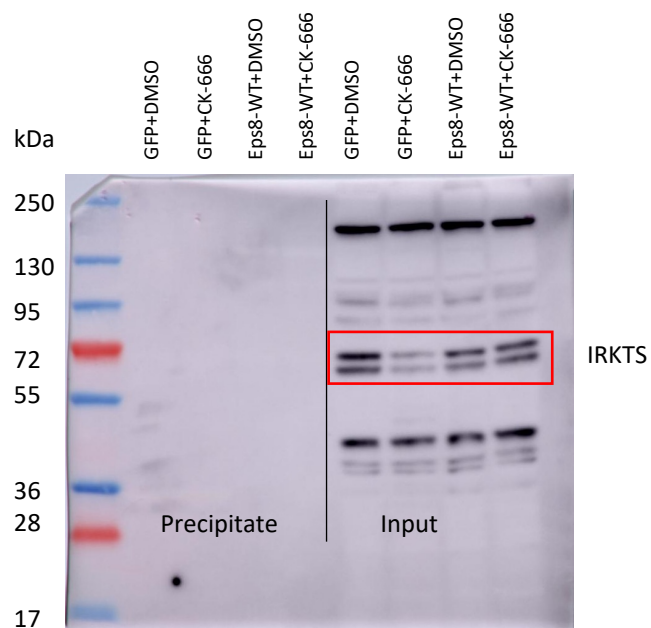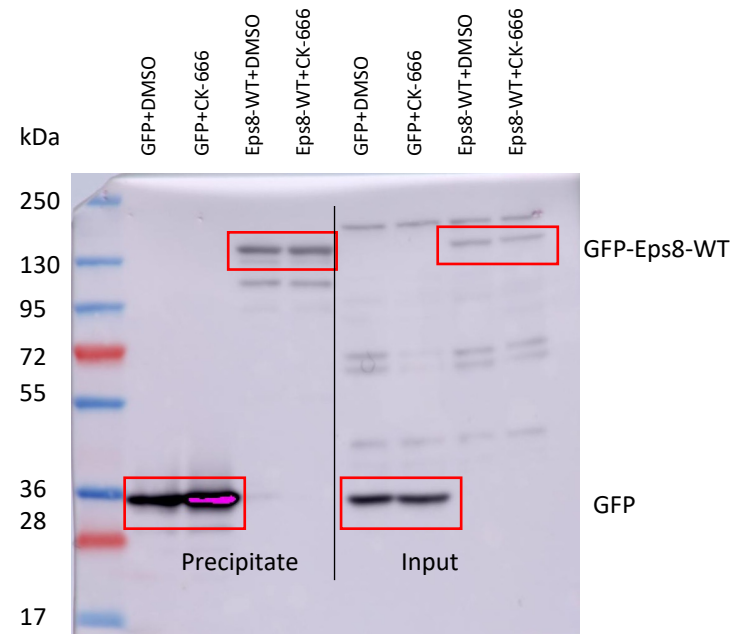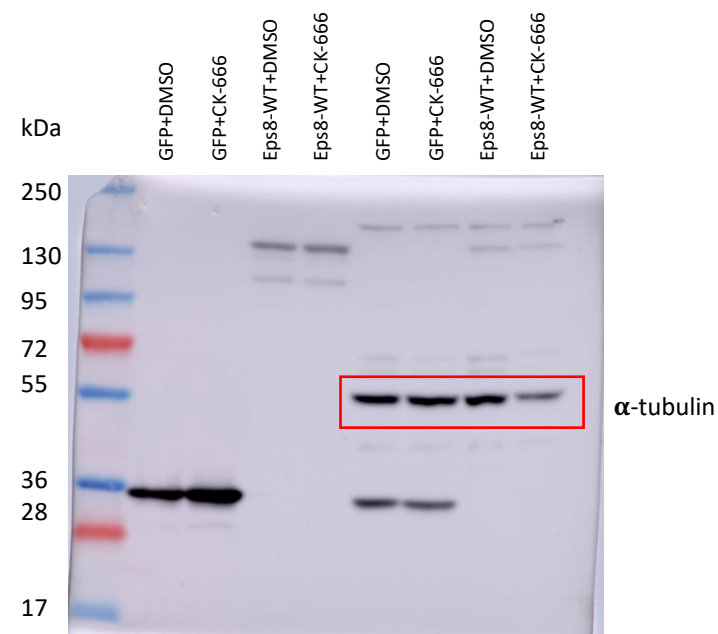

Supplement: Supplementary file 26 — Source Data for Figure 4 [file EMBJ-42-e113761-s029.zip › Figure 4/4A/Annotated blots.pdf]

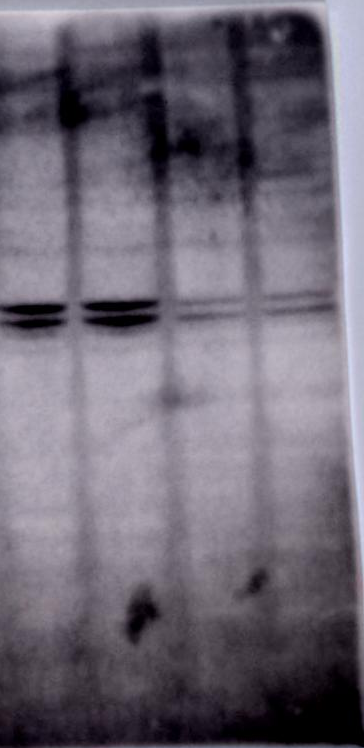

Supplement: Supplementary file 26 — Source Data for Figure 4 [file EMBJ-42-e113761-s029.zip › Figure 4/4A/Ch+Ladder/input anti IRSp53 in GFP and Eps8-wt with DMSO and CK-666_2 (IRSp53).tif]

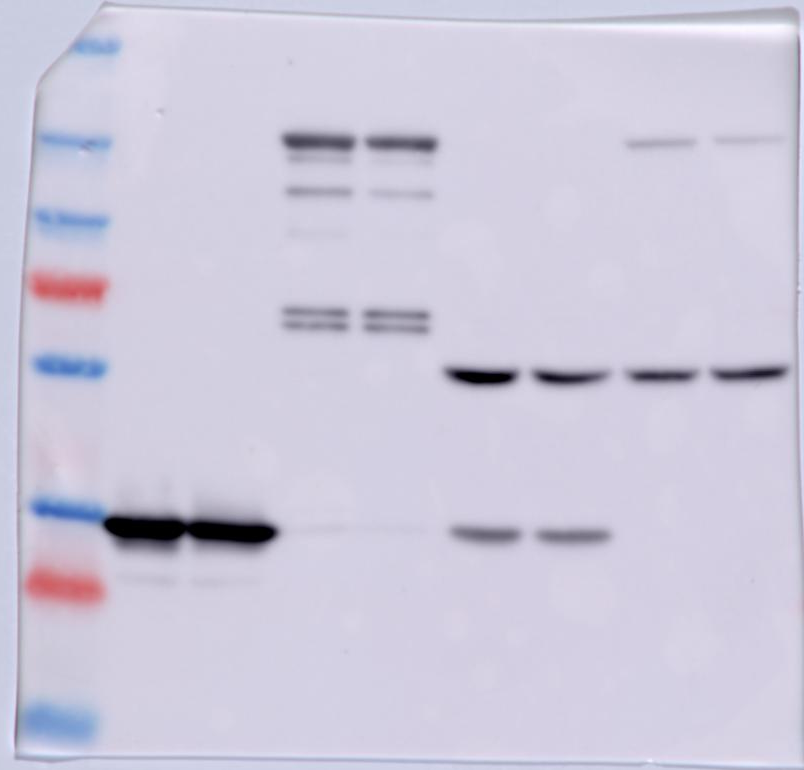

Supplement: Supplementary file 26 — Source Data for Figure 4 [file EMBJ-42-e113761-s029.zip › Figure 4/4A/Ch+Ladder/input anti tubulin in GFP and Eps8-wt with DMSO and CK-666 (IRSp53).tif]

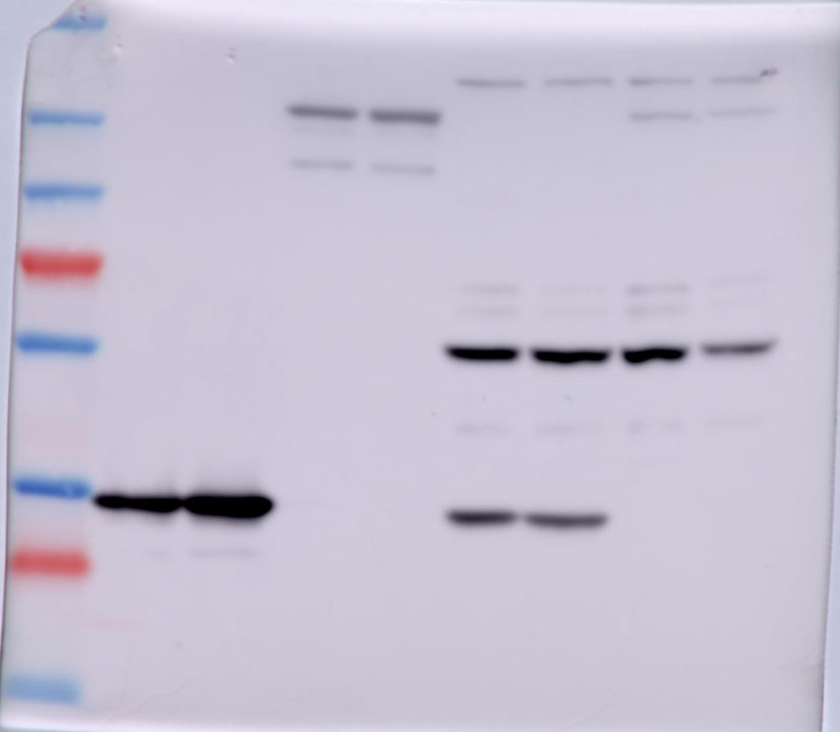

Supplement: Supplementary file 26 — Source Data for Figure 4 [file EMBJ-42-e113761-s029.zip › Figure 4/4A/Ch+Ladder/input anti tubulin in GFP and Eps8-wt with DMSO and CK-666 (IRTKS).tif]

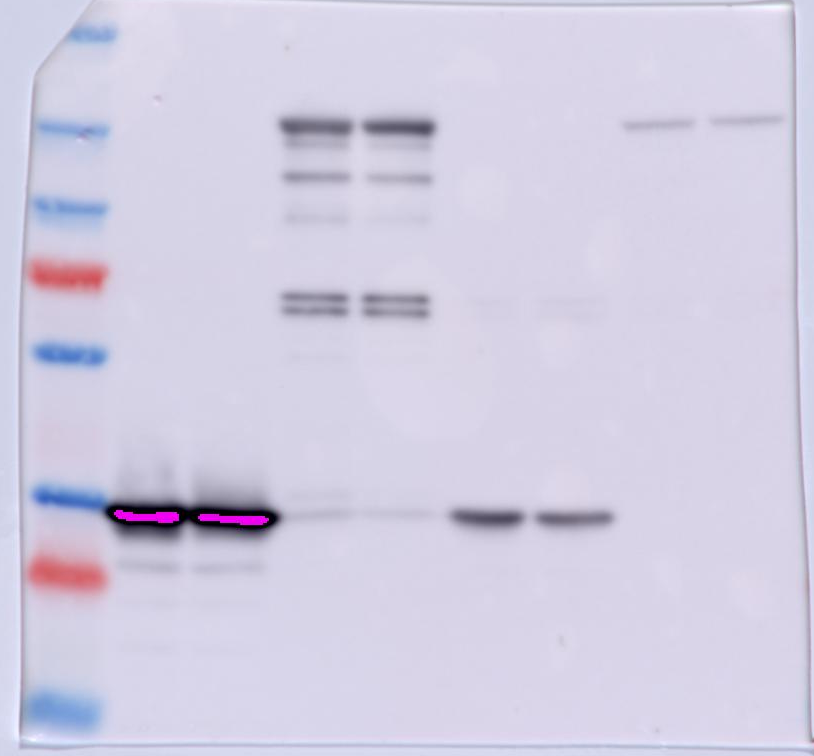

Supplement: Supplementary file 26 — Source Data for Figure 4 [file EMBJ-42-e113761-s029.zip › Figure 4/4A/Ch+Ladder/precipitate + input anti GFP in GFP and Eps8-wt with DMSO and CK-666 (IRSp53).tif]

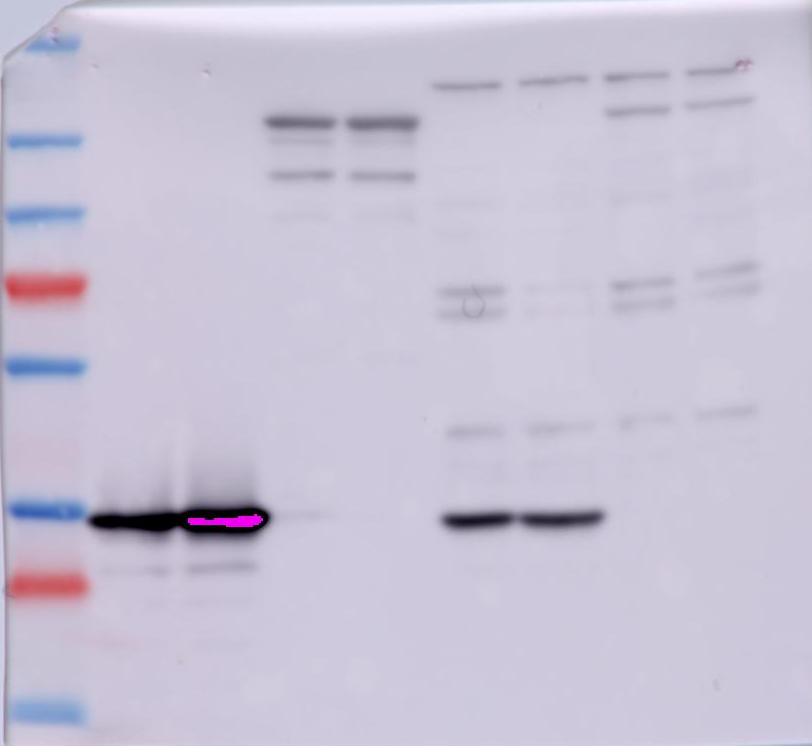

Supplement: Supplementary file 26 — Source Data for Figure 4 [file EMBJ-42-e113761-s029.zip › Figure 4/4A/Ch+Ladder/precipitate + input anti GFP in GFP and Eps8-wt with DMSO and CK-666 (IRTKS).tif]

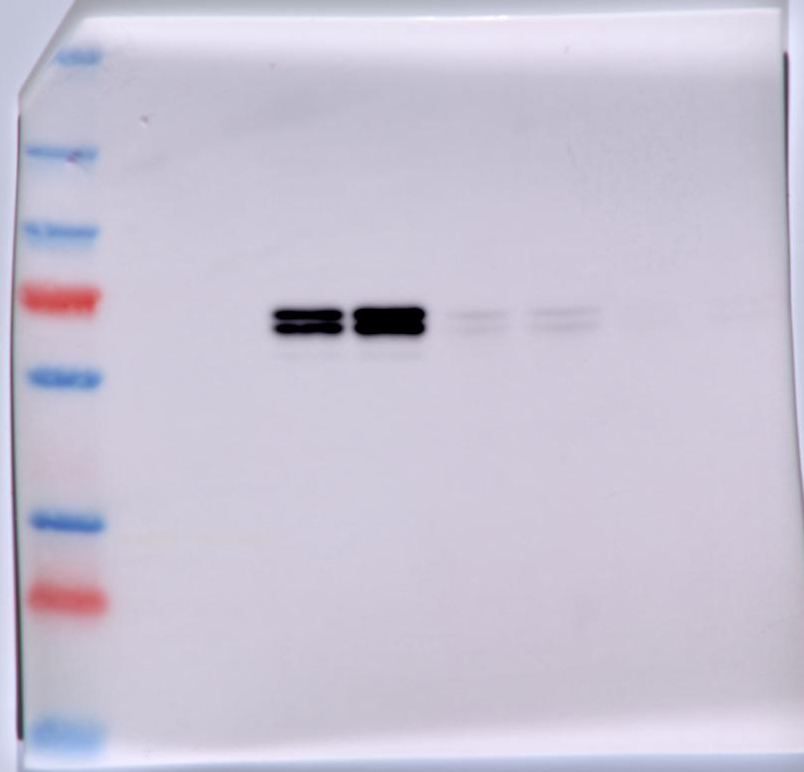

Supplement: Supplementary file 26 — Source Data for Figure 4 [file EMBJ-42-e113761-s029.zip › Figure 4/4A/Ch+Ladder/precipitate + input anti IRSp53 in GFP and Eps8-wt with DMSO and CK-666 (IRSp53).tif]

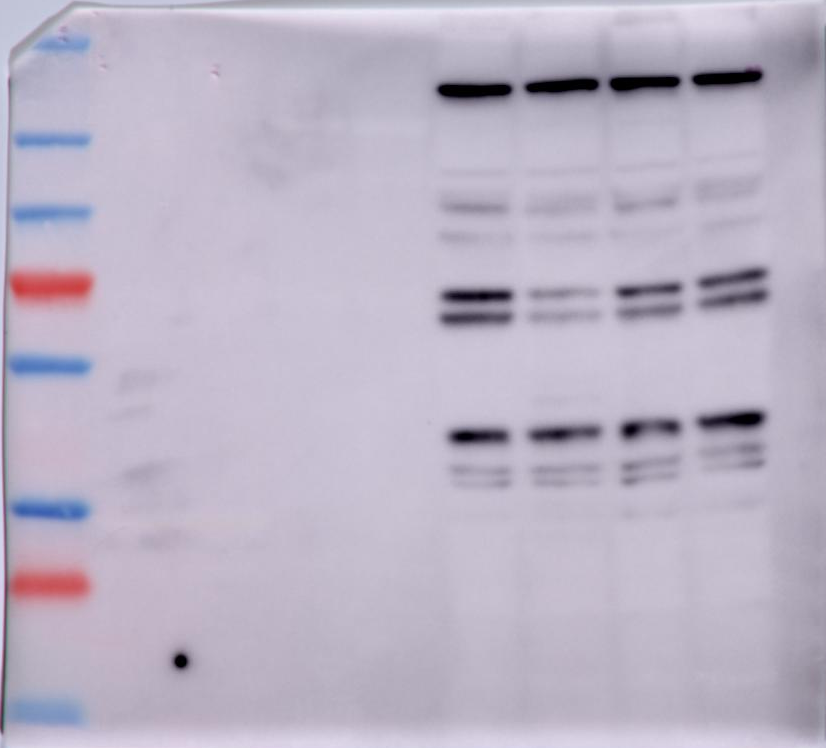

Supplement: Supplementary file 26 — Source Data for Figure 4 [file EMBJ-42-e113761-s029.zip › Figure 4/4A/Ch+Ladder/precipitate + input anti IRTKS in GFP and Eps8-wt with DMSO and CK-666 (IRTKS).tif]

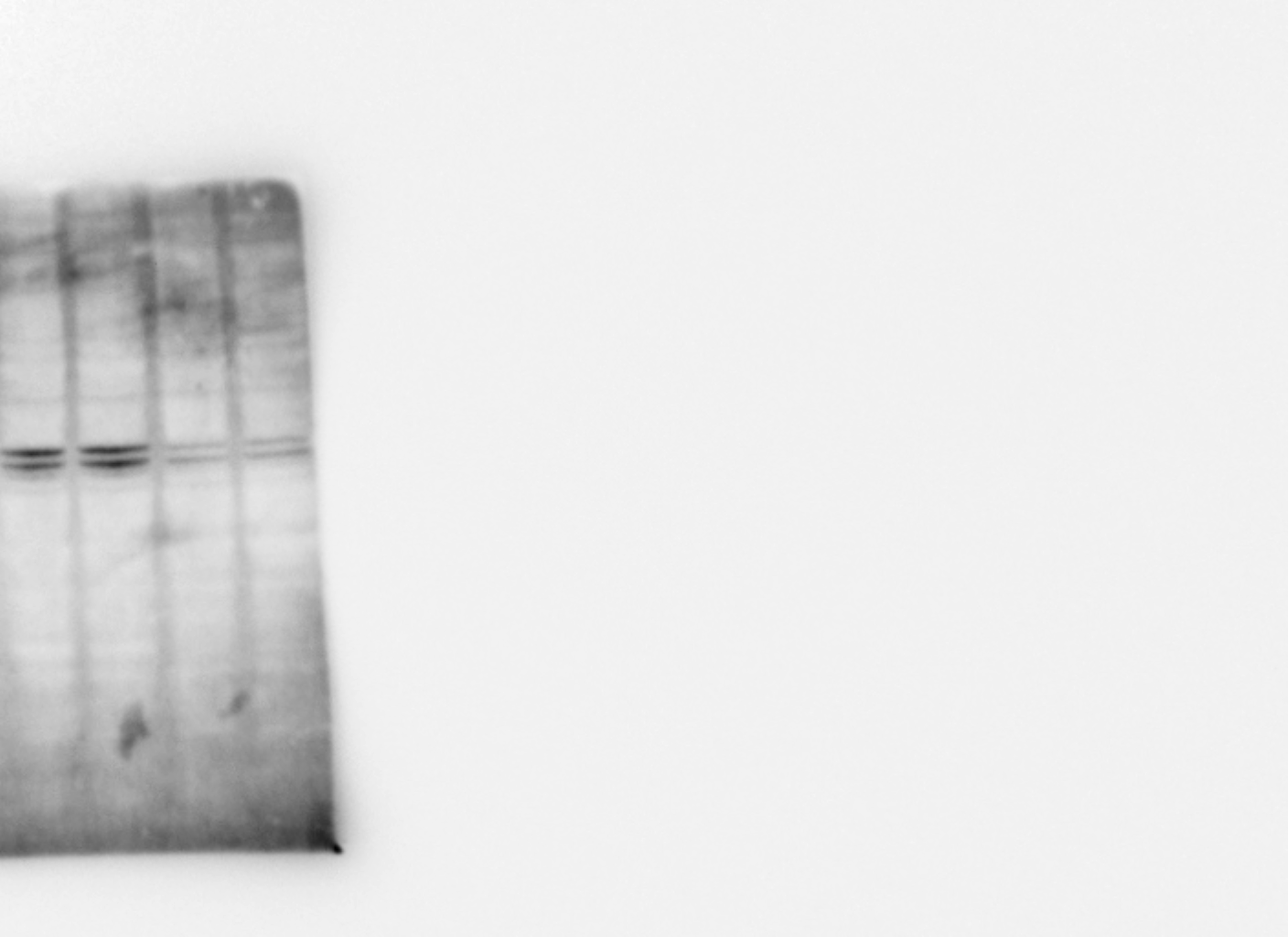

Supplement: Supplementary file 26 — Source Data for Figure 4 [file EMBJ-42-e113761-s029.zip › Figure 4/4A/Raw TIFFs/western lysate anti IRSp53 in GFP and Eps8-wt with DMSO and CK-666.tiff]

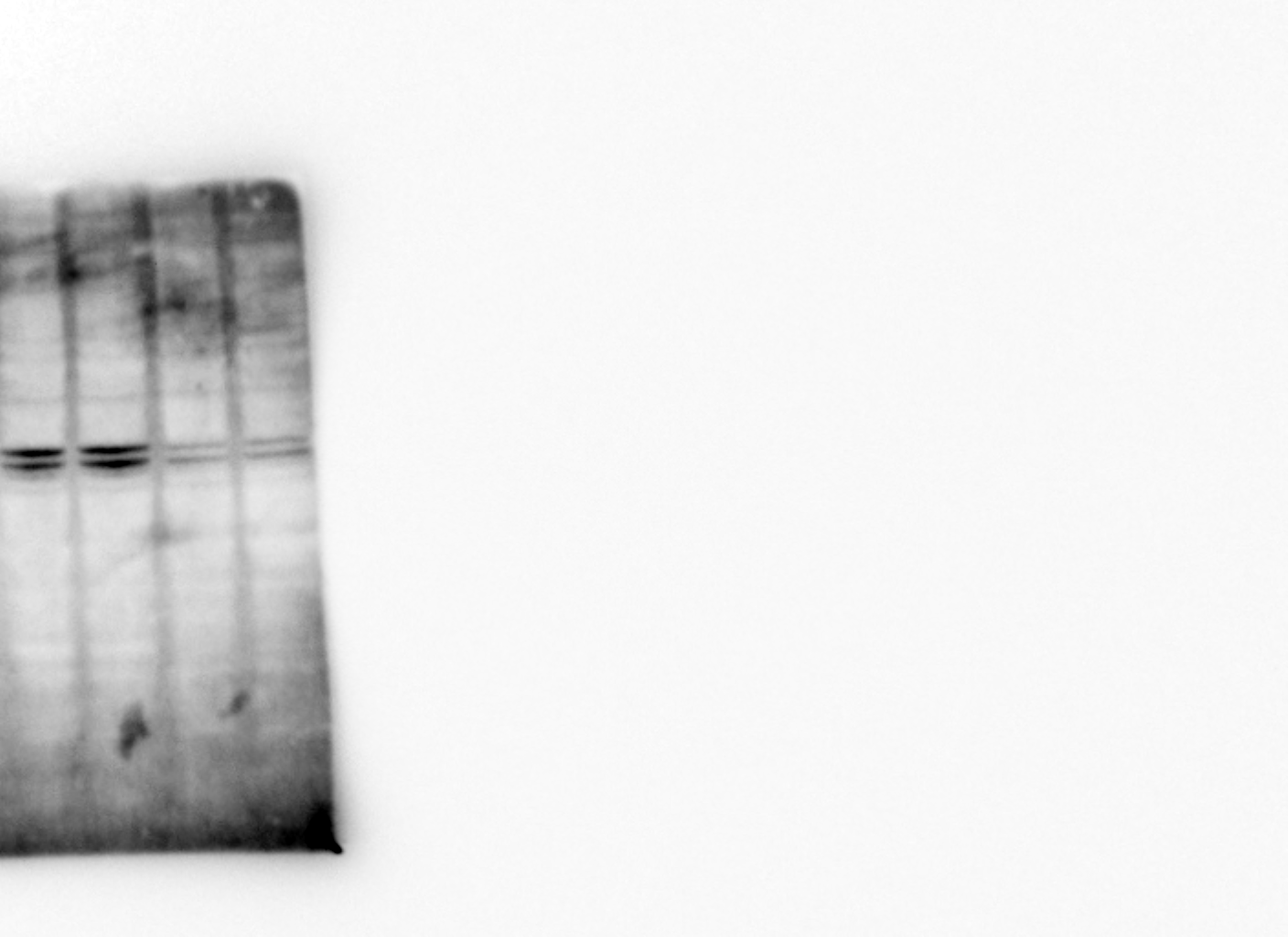

Supplement: Supplementary file 26 — Source Data for Figure 4 [file EMBJ-42-e113761-s029.zip › Figure 4/4A/Raw TIFFs/western lysate anti IRSp53 in GFP and Eps8-wt with DMSO and CK-666_2.tif]

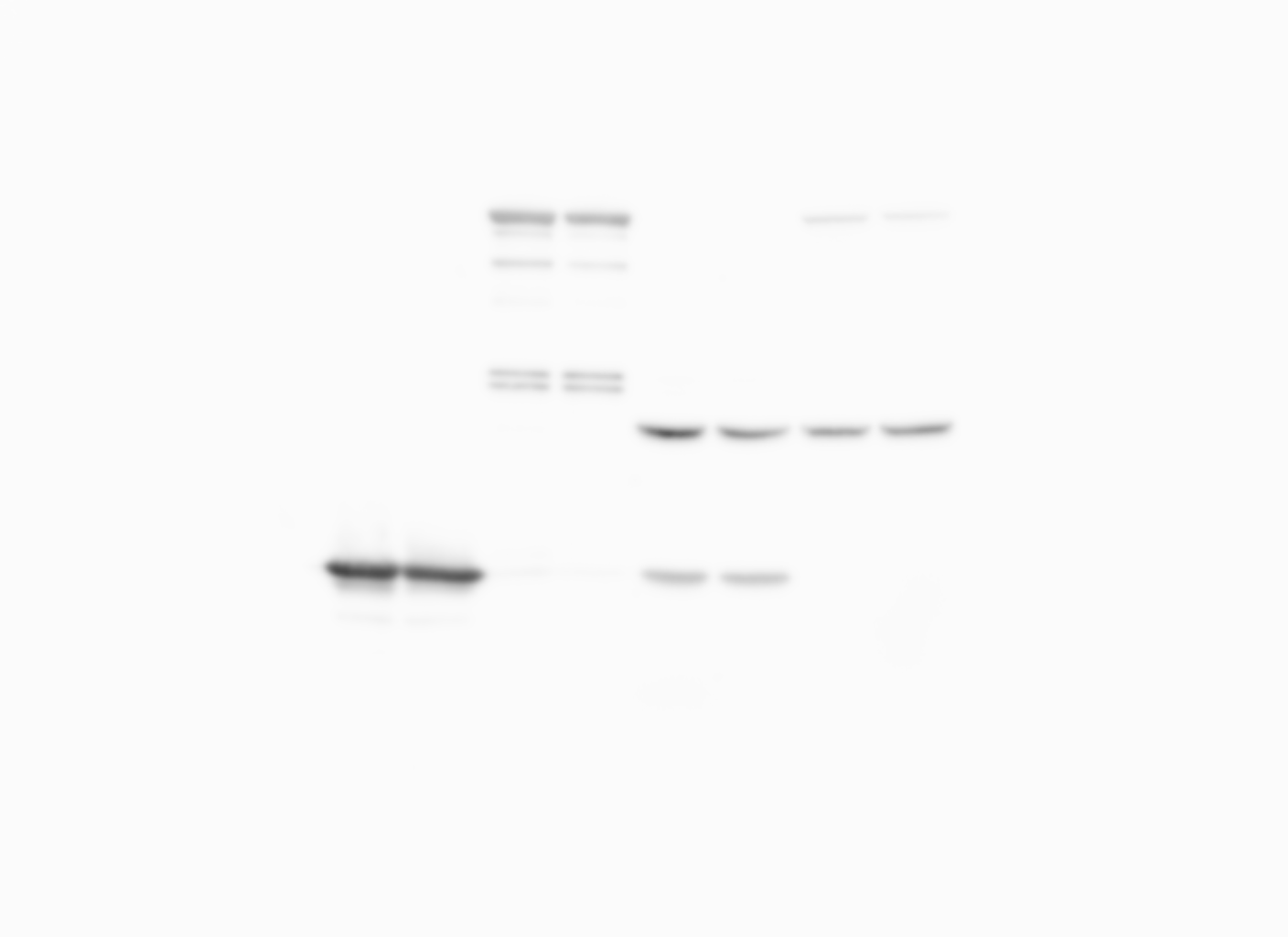

Supplement: Supplementary file 26 — Source Data for Figure 4 [file EMBJ-42-e113761-s029.zip › Figure 4/4A/Raw TIFFs/western lysate anti tubulin in GFP and Eps8-wt with DMSO and CK-666.tif]

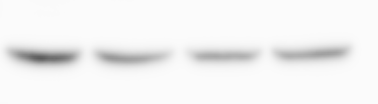

Supplement: Supplementary file 26 — Source Data for Figure 4 [file EMBJ-42-e113761-s029.zip › Figure 4/4A/Raw TIFFs/western lysate anti tubulin in GFP and Eps8-wt with DMSO and CK-666_2.tif]

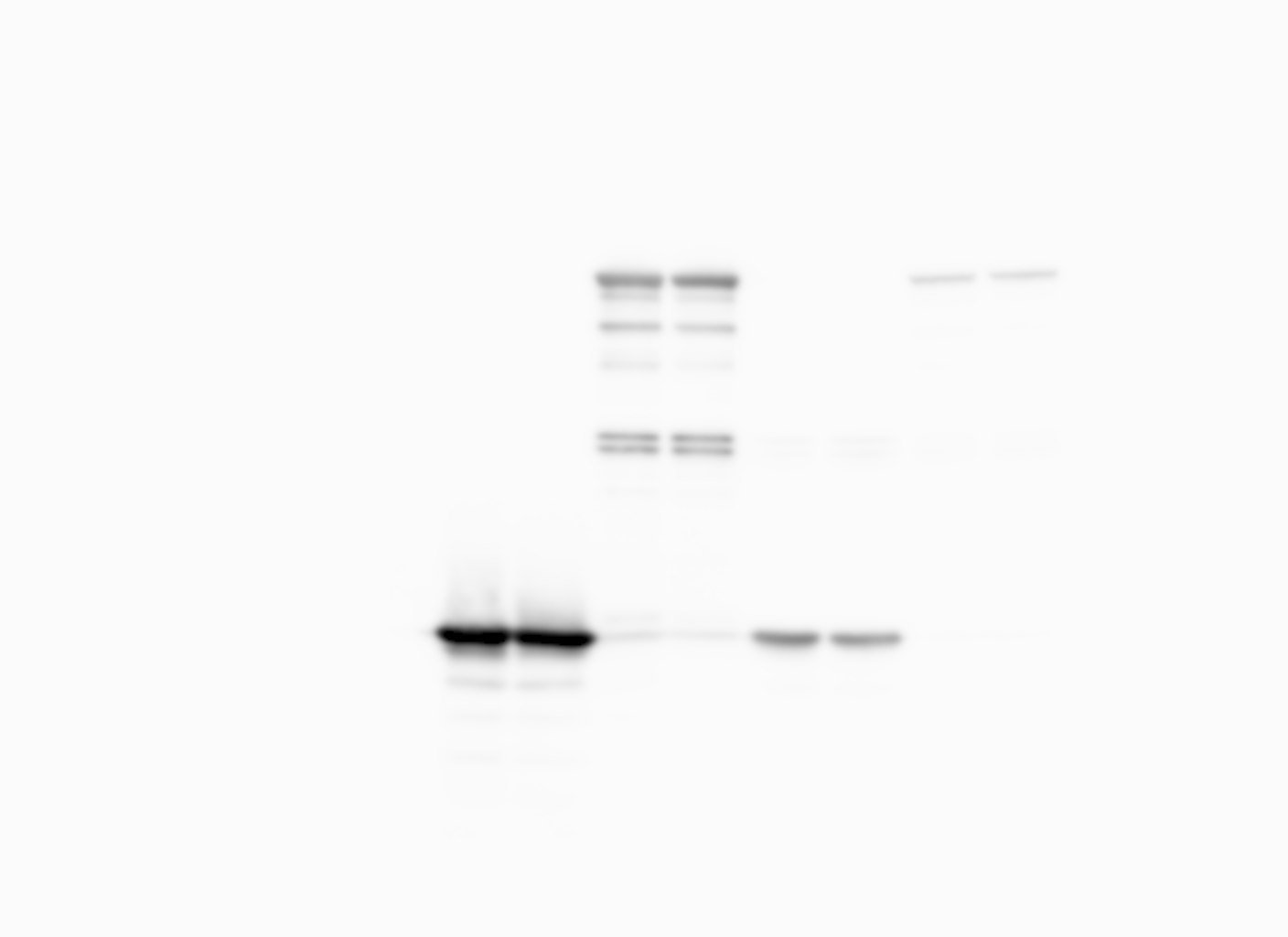

Supplement: Supplementary file 26 — Source Data for Figure 4 [file EMBJ-42-e113761-s029.zip › Figure 4/4A/Raw TIFFs/western precipitate + lysate anti GFP in GFP and Eps8-wt with DMSO and CK-666_2.tif]

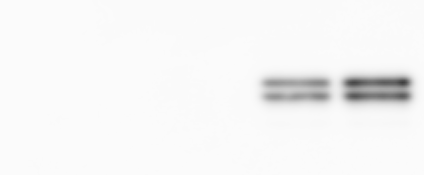

Supplement: Supplementary file 26 — Source Data for Figure 4 [file EMBJ-42-e113761-s029.zip › Figure 4/4A/Raw TIFFs/western precipitate anti IRSp53 in GFP and Eps8-wt with DMSO and CK-666.tif]

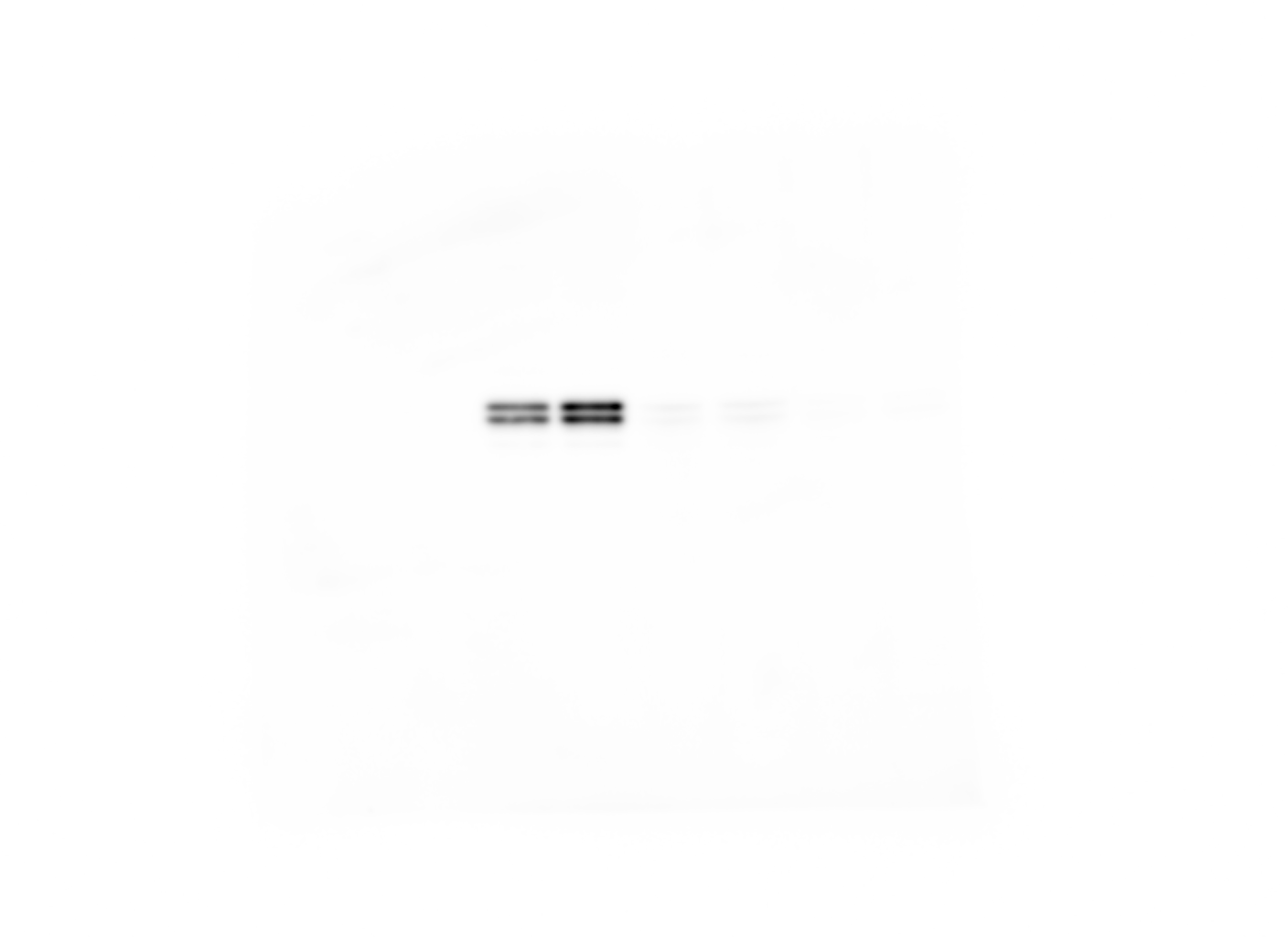

Supplement: Supplementary file 26 — Source Data for Figure 4 [file EMBJ-42-e113761-s029.zip › Figure 4/4A/Raw TIFFs/western precipitate anti IRSp53 in GFP and Eps8-wt with DMSO and CK-666_2.tif]

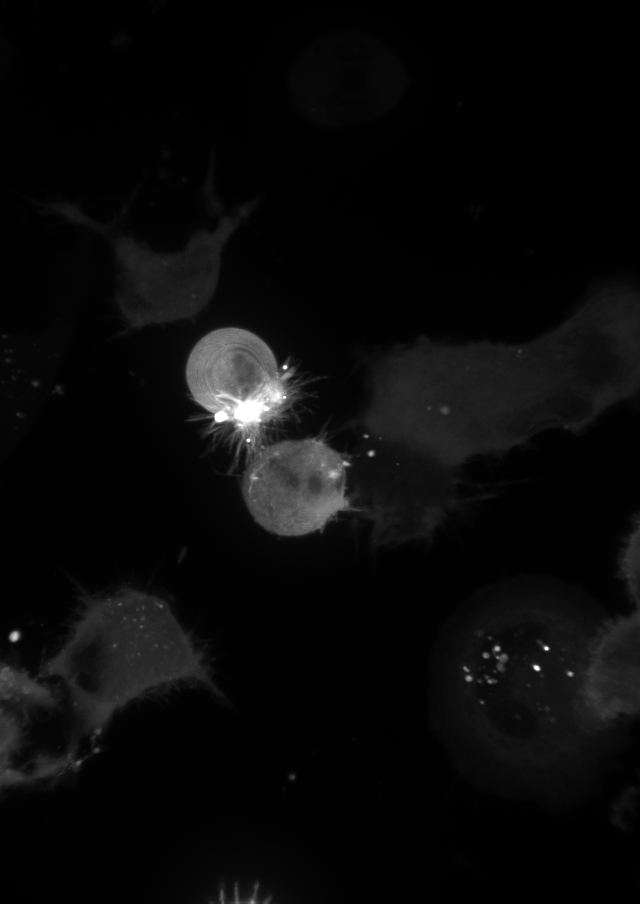

Supplement: Supplementary file 26 — Source Data for Figure 4 [file EMBJ-42-e113761-s029.zip › Figure 4/4C/MAX_IRSp53-GFP_2 min.tif]

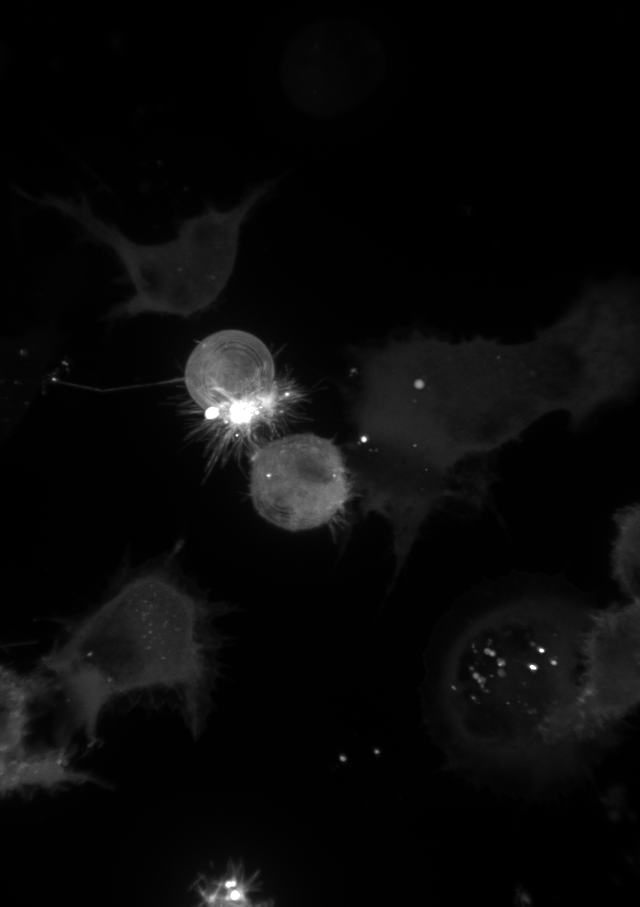

Supplement: Supplementary file 26 — Source Data for Figure 4 [file EMBJ-42-e113761-s029.zip › Figure 4/4C/MAX_IRSp53-GFP_36 min.tif]

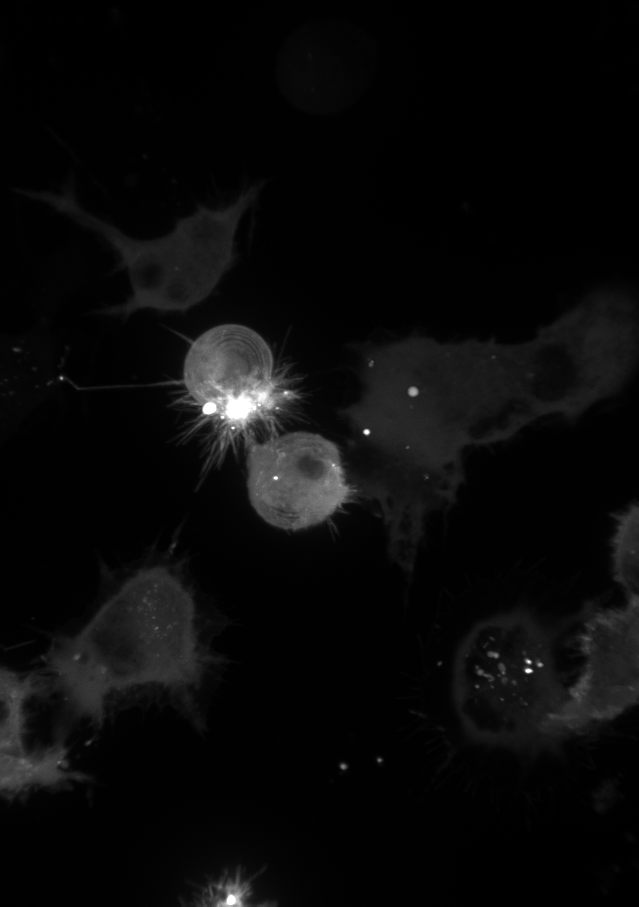

Supplement: Supplementary file 26 — Source Data for Figure 4 [file EMBJ-42-e113761-s029.zip › Figure 4/4C/MAX_IRSp53-GFP_45 min.tif]

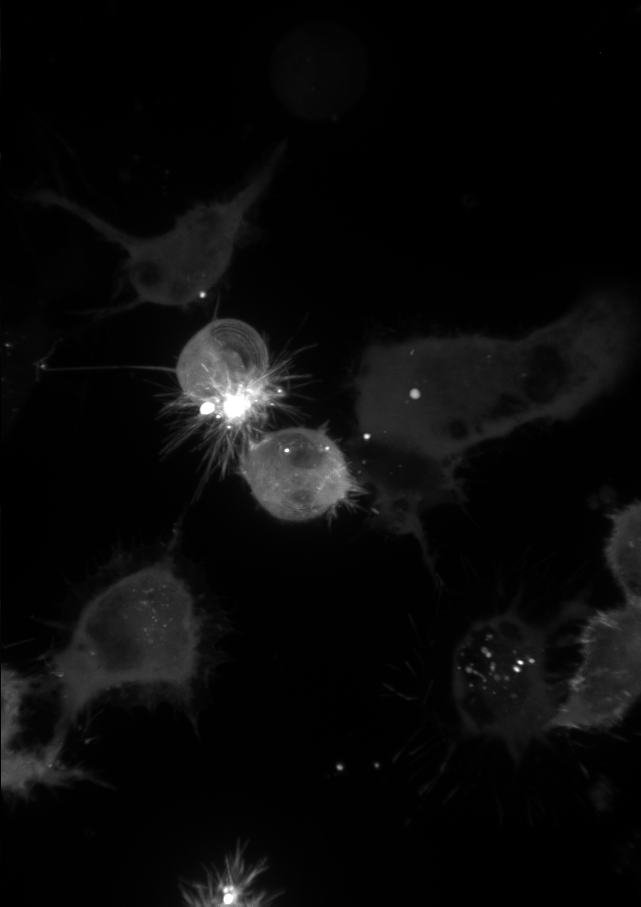

Supplement: Supplementary file 26 — Source Data for Figure 4 [file EMBJ-42-e113761-s029.zip › Figure 4/4C/MAX_IRSp53-GFP_56 min.tif]

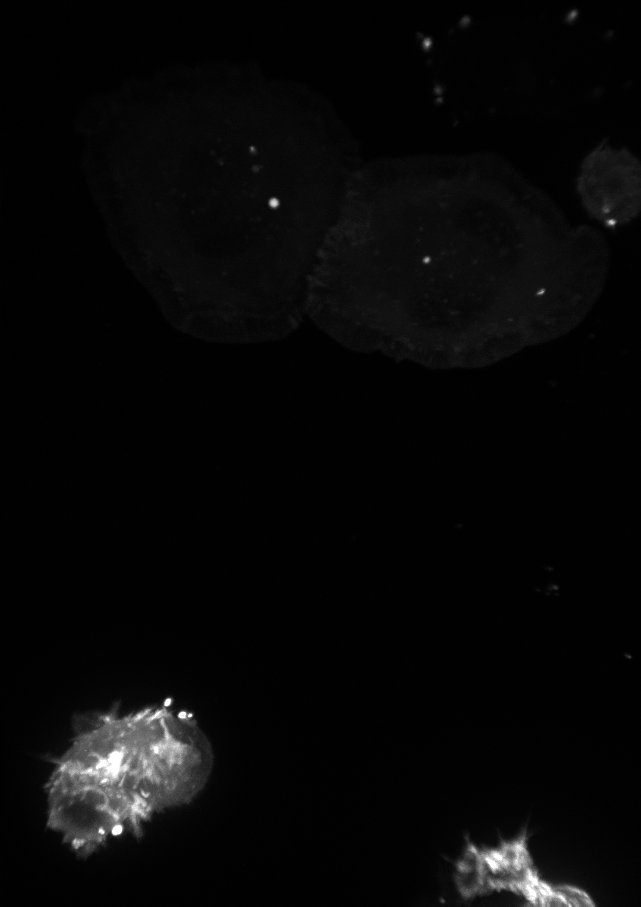

Supplement: Supplementary file 26 — Source Data for Figure 4 [file EMBJ-42-e113761-s029.zip › Figure 4/4F/MAX_GFP-Eps8-deltaCAP-0 min.tif]

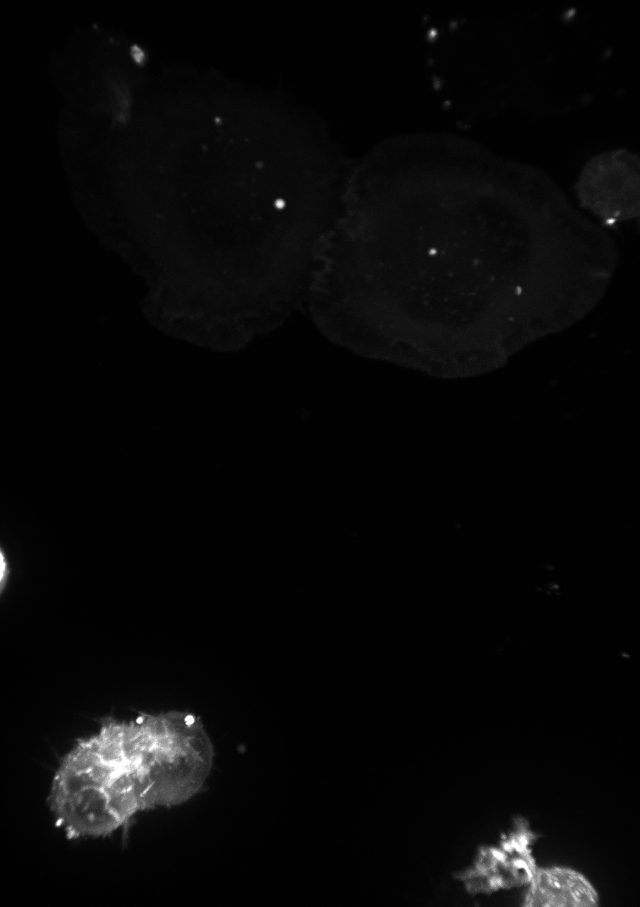

Supplement: Supplementary file 26 — Source Data for Figure 4 [file EMBJ-42-e113761-s029.zip › Figure 4/4F/MAX_GFP-Eps8-deltaCAP-15 min.tif]

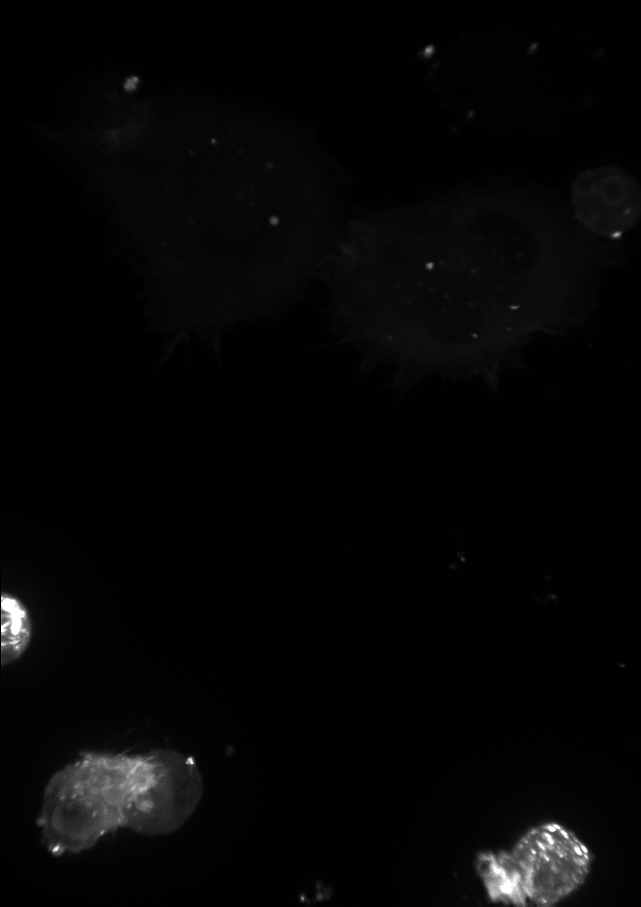

Supplement: Supplementary file 26 — Source Data for Figure 4 [file EMBJ-42-e113761-s029.zip › Figure 4/4F/MAX_GFP-Eps8-deltaCAP-45 min.tif]

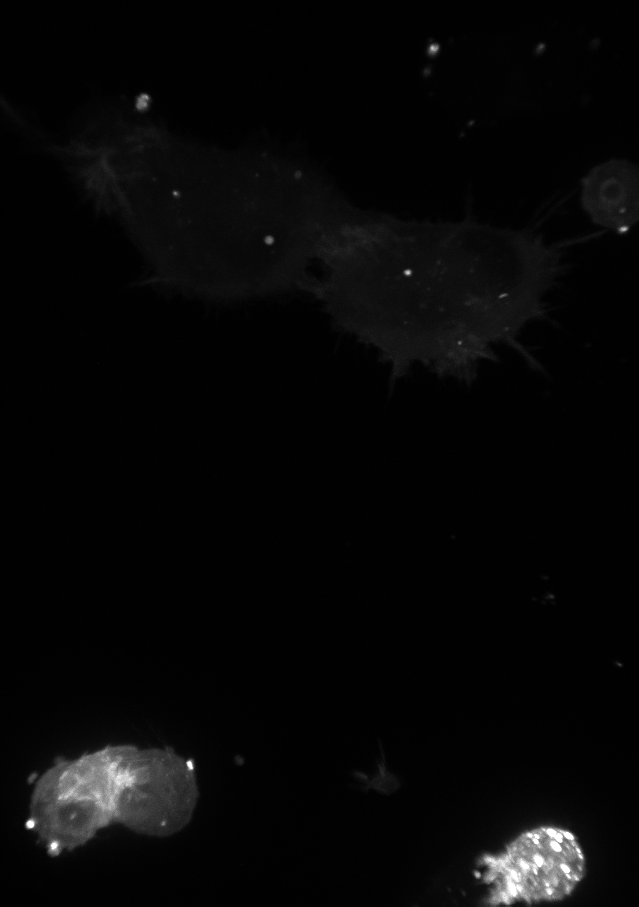

Supplement: Supplementary file 26 — Source Data for Figure 4 [file EMBJ-42-e113761-s029.zip › Figure 4/4F/MAX_GFP-Eps8-deltaCAP-65 min.tif]

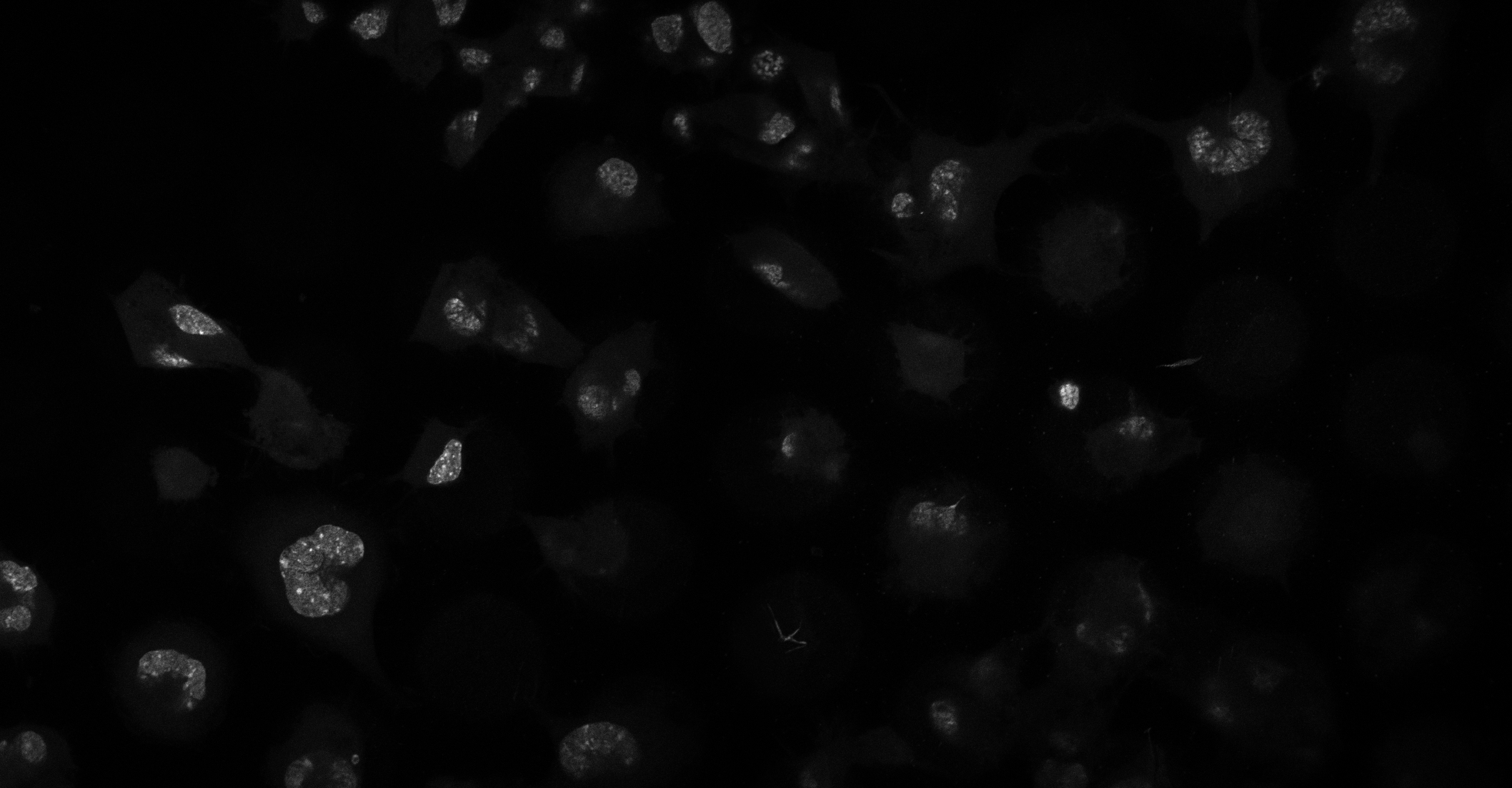

Supplement: Supplementary file 27 — Source Data for Figure 5 [file EMBJ-42-e113761-s005.zip › Figure 5/5A/CK-666/Eps8 + IRSp53/Surface/(Blue-DAPI and Fibronectin AF405)-MAX_Eps8deltaCAP-IRSp53-CK-666-stacks 6-7.tif]

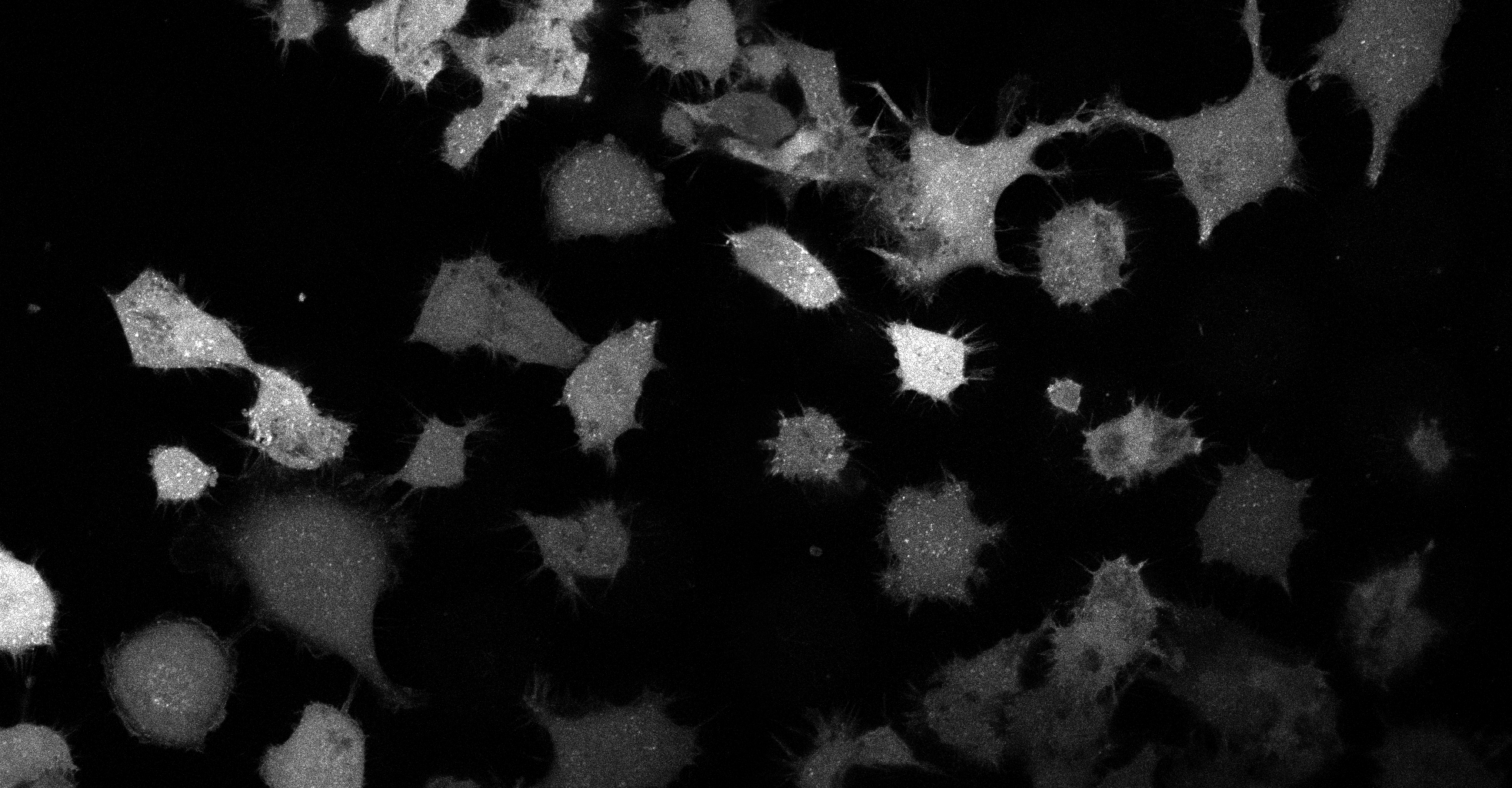

Supplement: Supplementary file 27 — Source Data for Figure 5 [file EMBJ-42-e113761-s005.zip › Figure 5/5A/CK-666/Eps8 + IRSp53/Surface/(Green-GFP-Eps8deltaCAP)-MAX_Eps8deltaCAP-IRSp53-CK-666-stacks 6-7.tif]

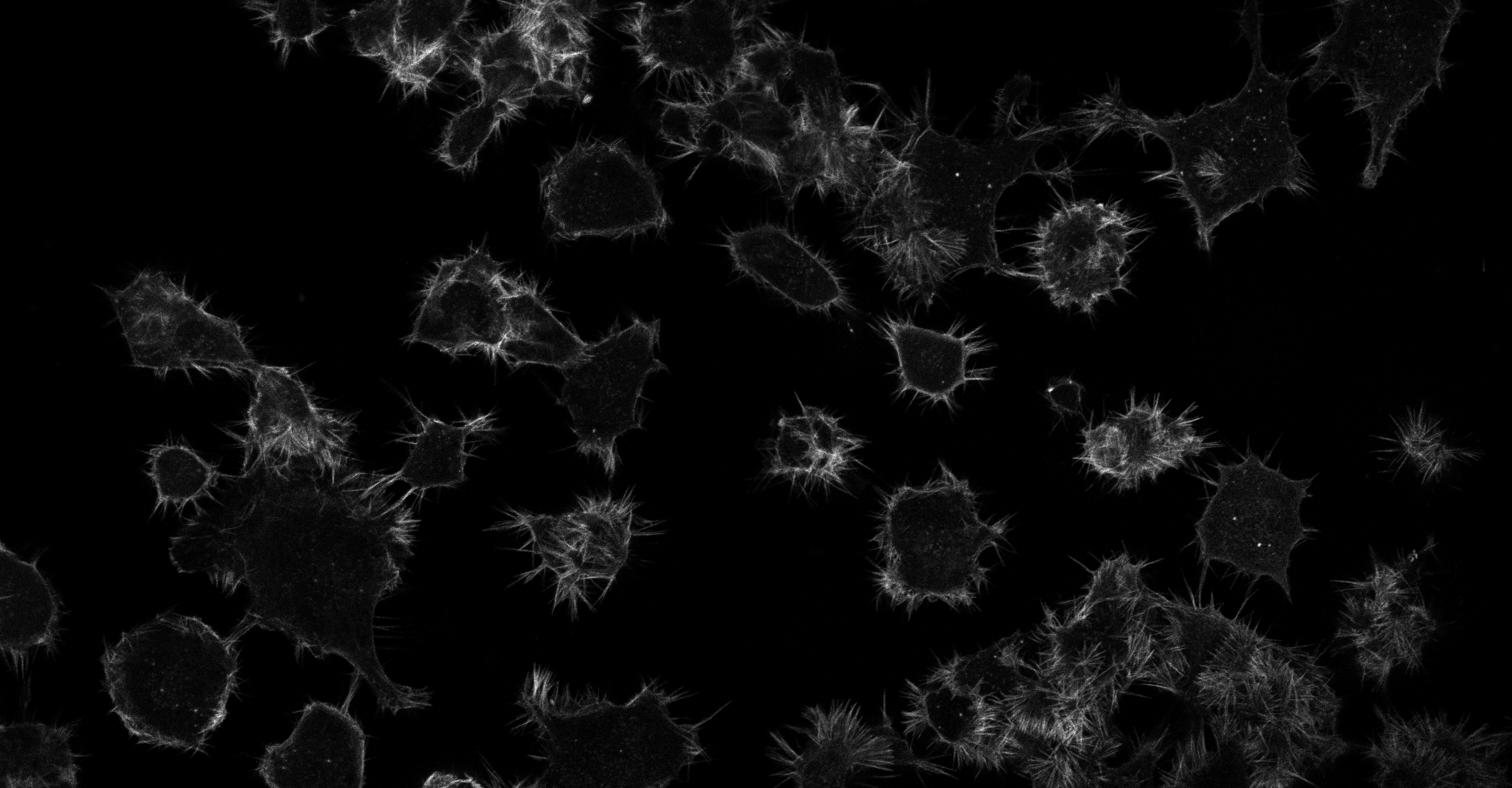

Supplement: Supplementary file 27 — Source Data for Figure 5 [file EMBJ-42-e113761-s005.zip › Figure 5/5A/CK-666/Eps8 + IRSp53/Surface/(Grey-Phalloidin AF647)-MAX_Eps8deltaCAP-IRSp53-CK-666-stacks 6-7.tif]

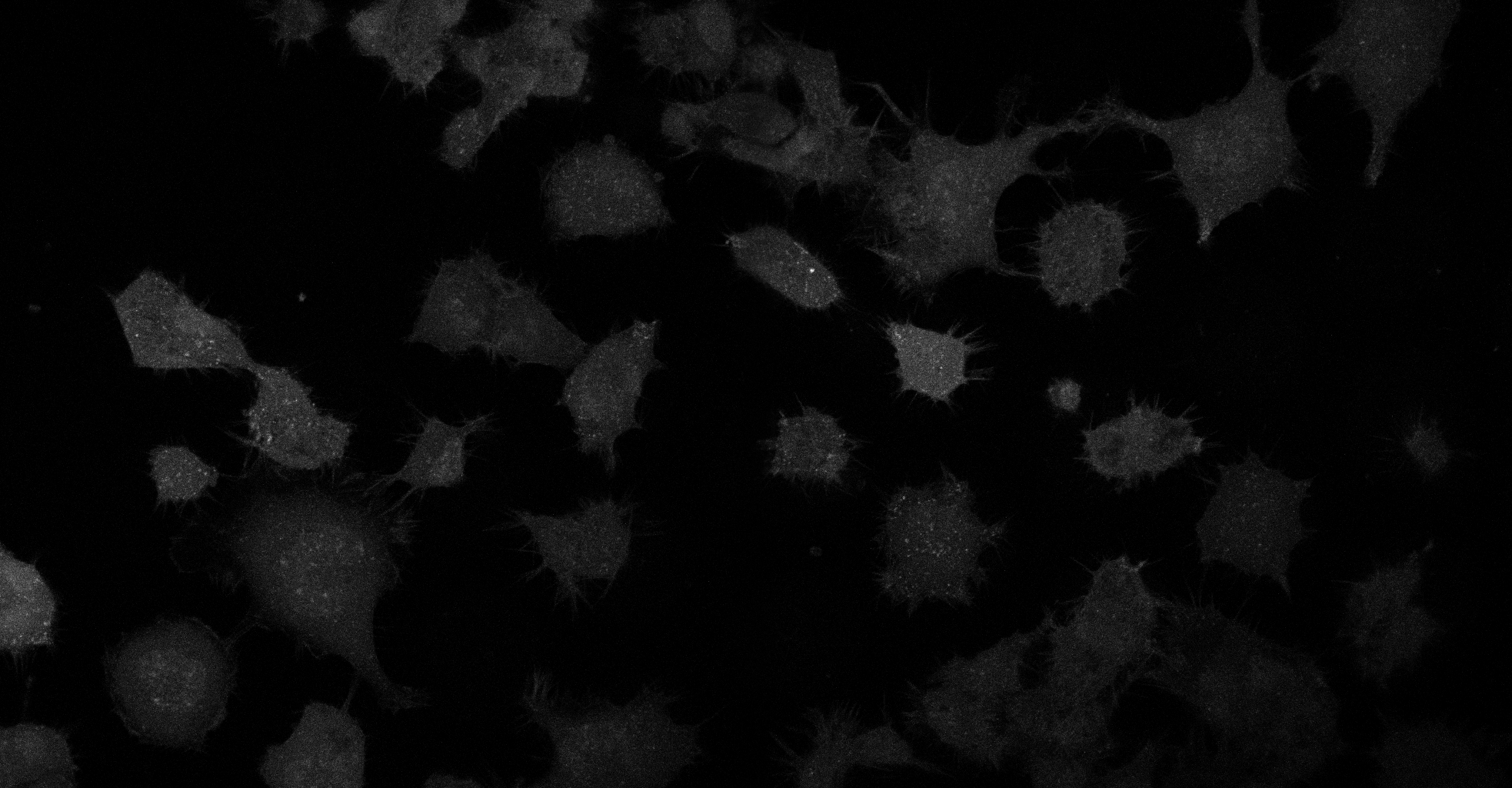

Supplement: Supplementary file 27 — Source Data for Figure 5 [file EMBJ-42-e113761-s005.zip › Figure 5/5A/CK-666/Eps8 + IRSp53/Surface/(Red-IRSp53-mCherry)-MAX_Eps8deltaCAP-IRSp53-CK-666-stacks 6-7.tif]

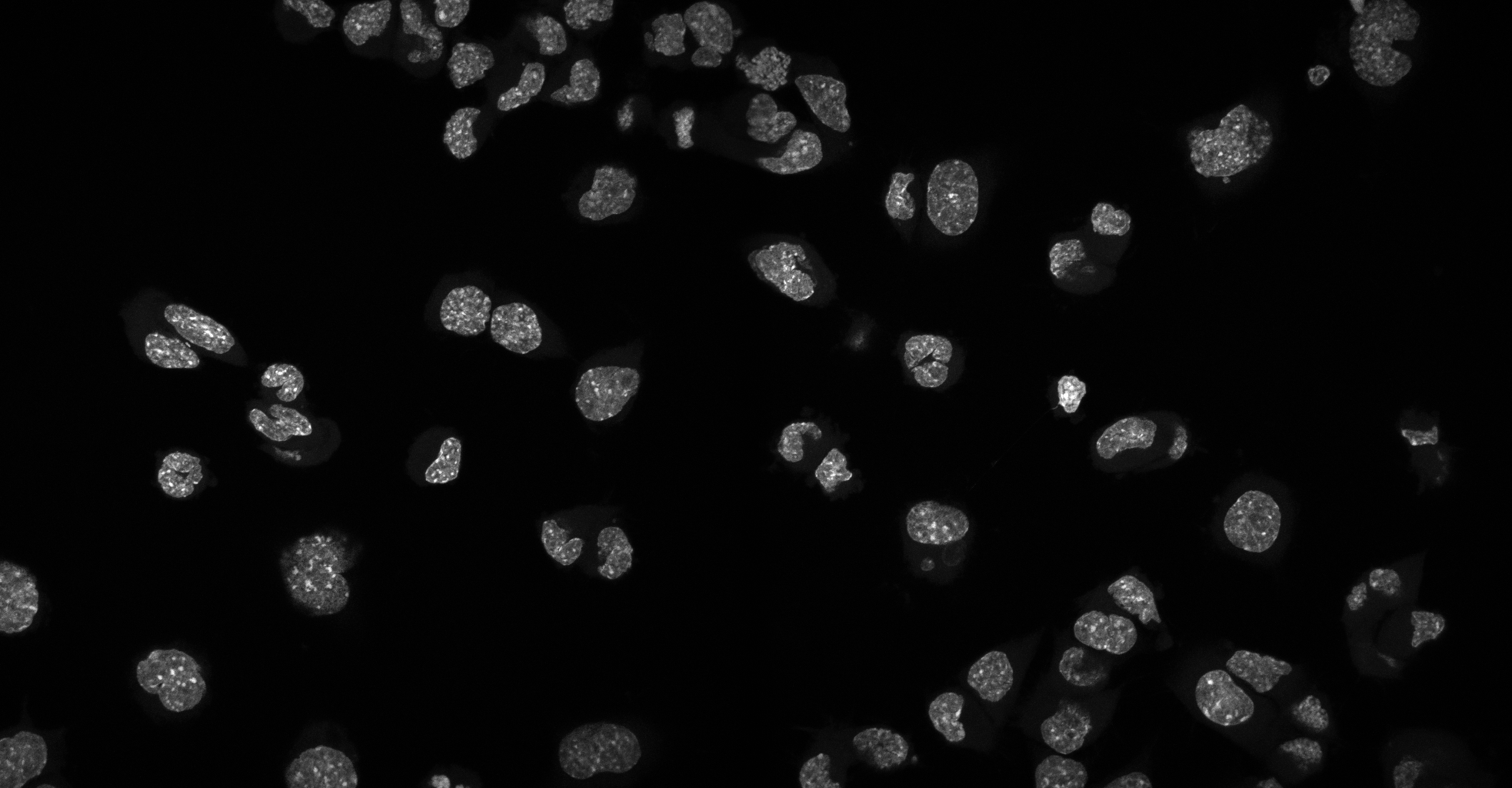

Supplement: Supplementary file 27 — Source Data for Figure 5 [file EMBJ-42-e113761-s005.zip › Figure 5/5A/CK-666/Eps8 + IRSp53/Upper Stacks/(Blue-DAPI and Fibronectin AF405)-MAX_Eps8deltaCAP-IRSp53-CK-666-stacks 11-16.tif]

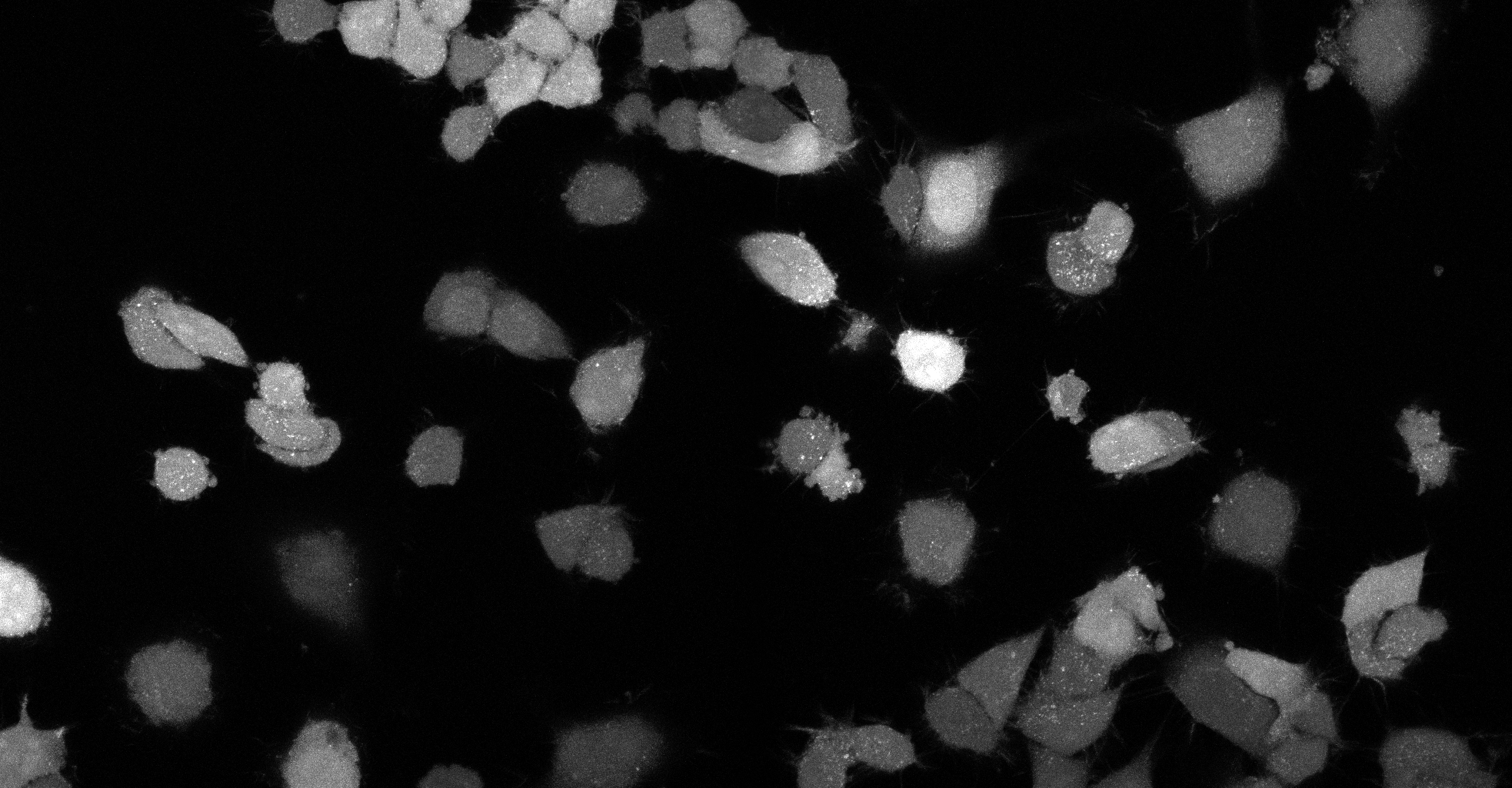

Supplement: Supplementary file 27 — Source Data for Figure 5 [file EMBJ-42-e113761-s005.zip › Figure 5/5A/CK-666/Eps8 + IRSp53/Upper Stacks/(Green-GFP-Eps8deltaCAP)-MAX_Eps8deltaCAP-IRSp53-CK-666-stacks 11-16.tif]

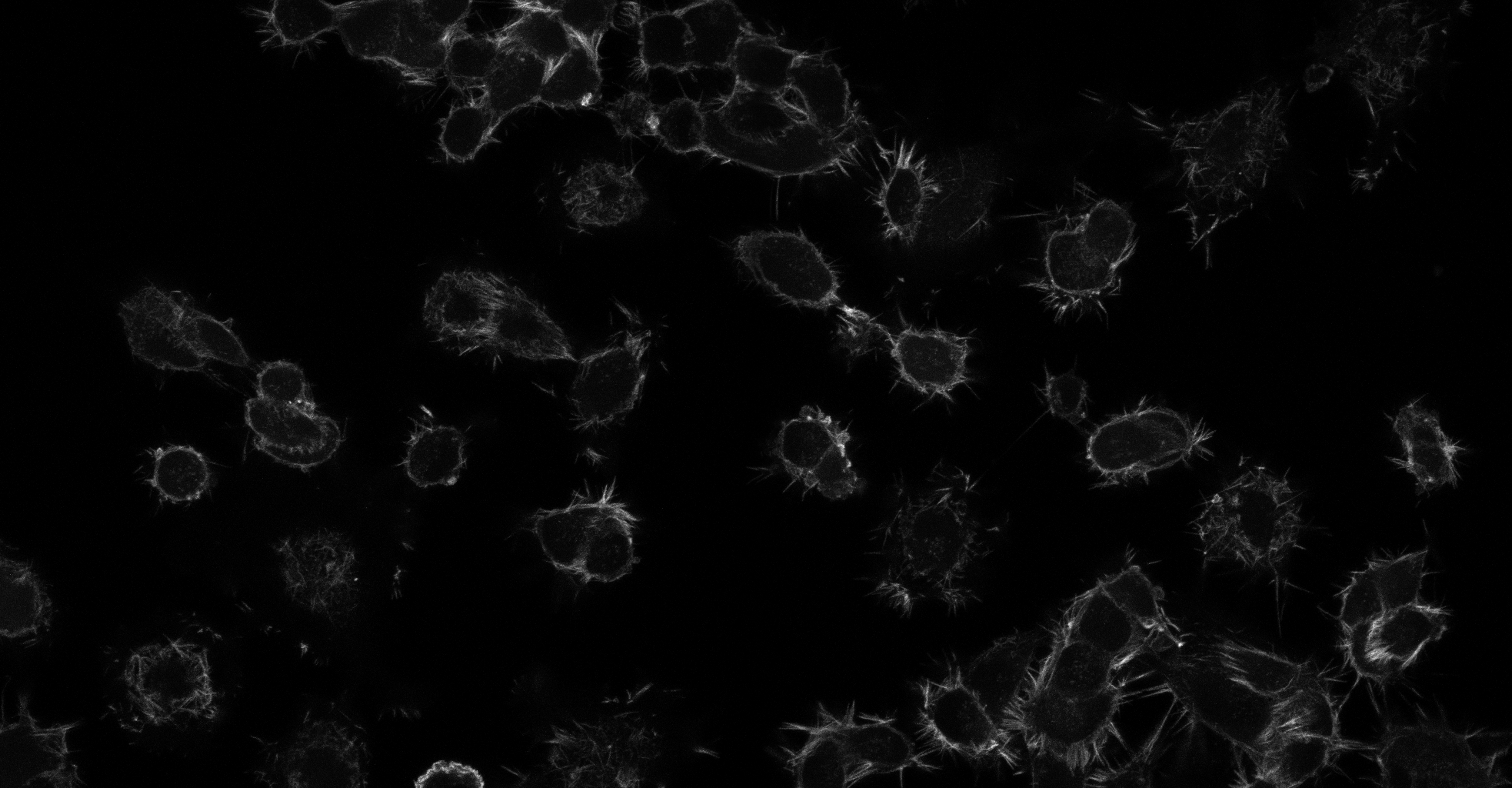

Supplement: Supplementary file 27 — Source Data for Figure 5 [file EMBJ-42-e113761-s005.zip › Figure 5/5A/CK-666/Eps8 + IRSp53/Upper Stacks/(Grey-Phalloidin AF647)-MAX_Eps8deltaCAP-IRSp53-CK-666-stacks 11-16.tif]

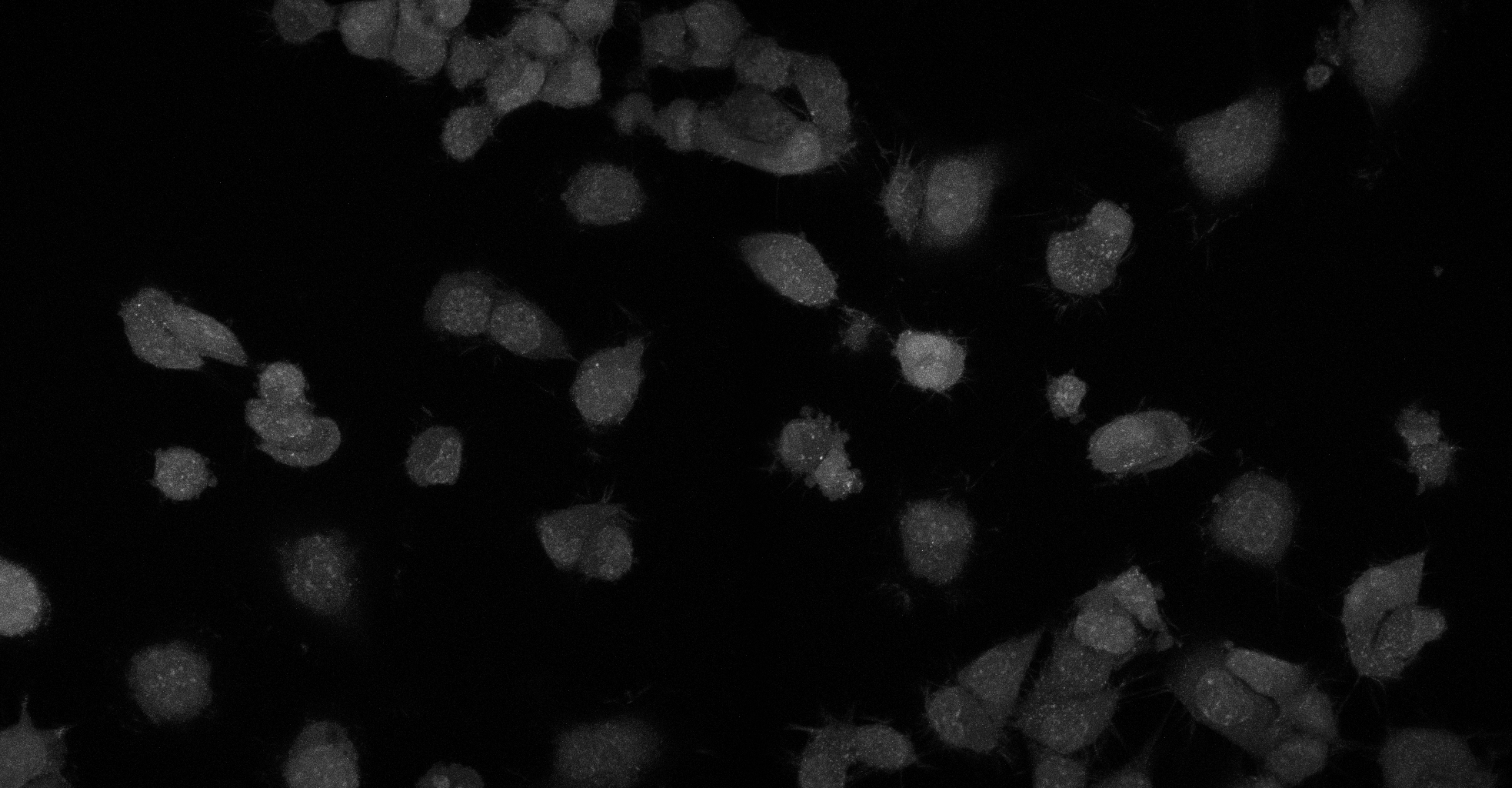

Supplement: Supplementary file 27 — Source Data for Figure 5 [file EMBJ-42-e113761-s005.zip › Figure 5/5A/CK-666/Eps8 + IRSp53/Upper Stacks/(Red-IRSp53-mCherry)-MAX_Eps8deltaCAP-IRSp53-CK-666-stacks 11-16.tif]

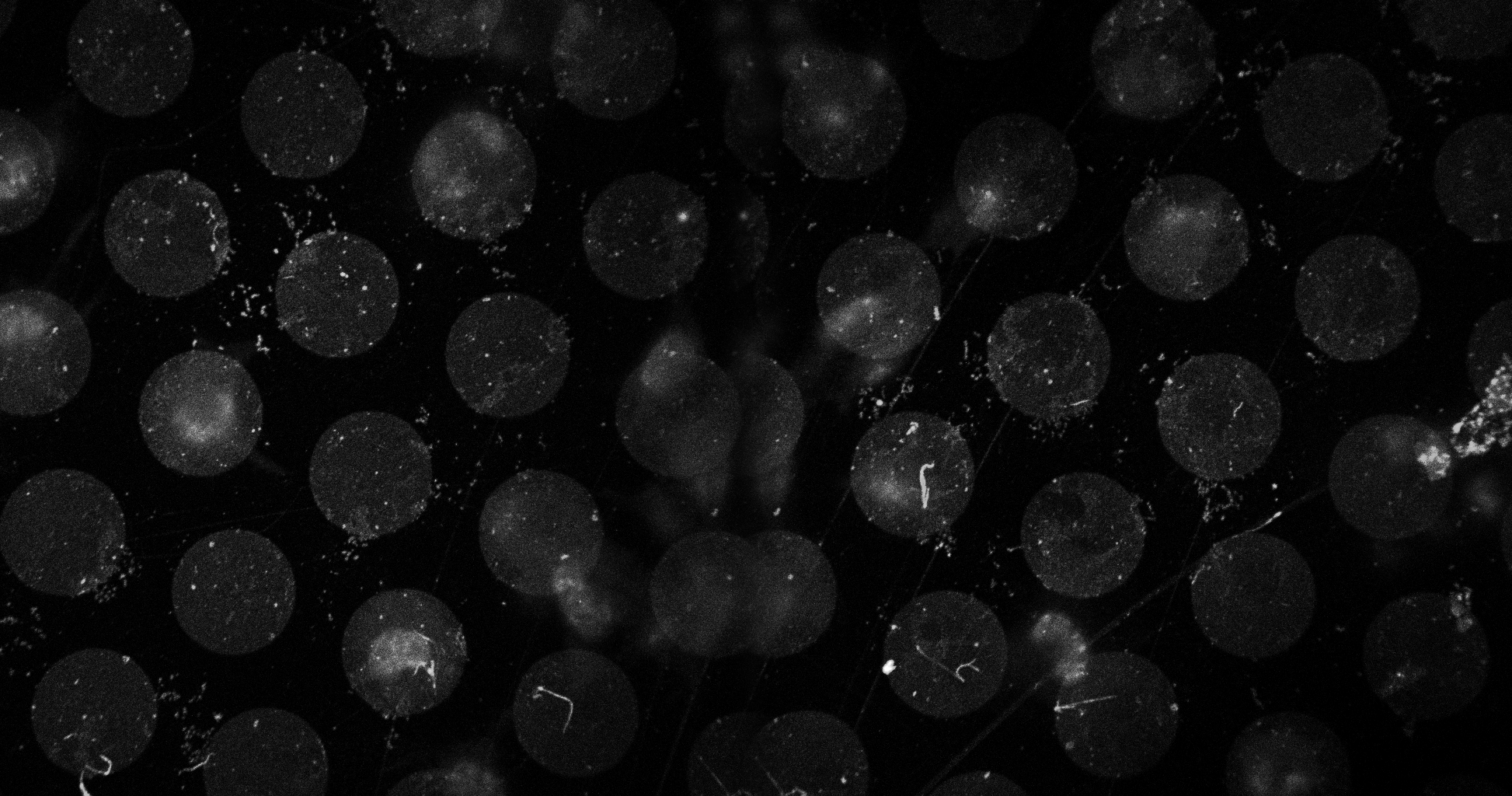

Supplement: Supplementary file 27 — Source Data for Figure 5 [file EMBJ-42-e113761-s005.zip › Figure 5/5A/CK-666/GFP + mCherry/Surface/(Blue-DAPI and Fibronectin AF405)-MAX_GFP-mCherry-CK-666-stacks 3-4.tif]

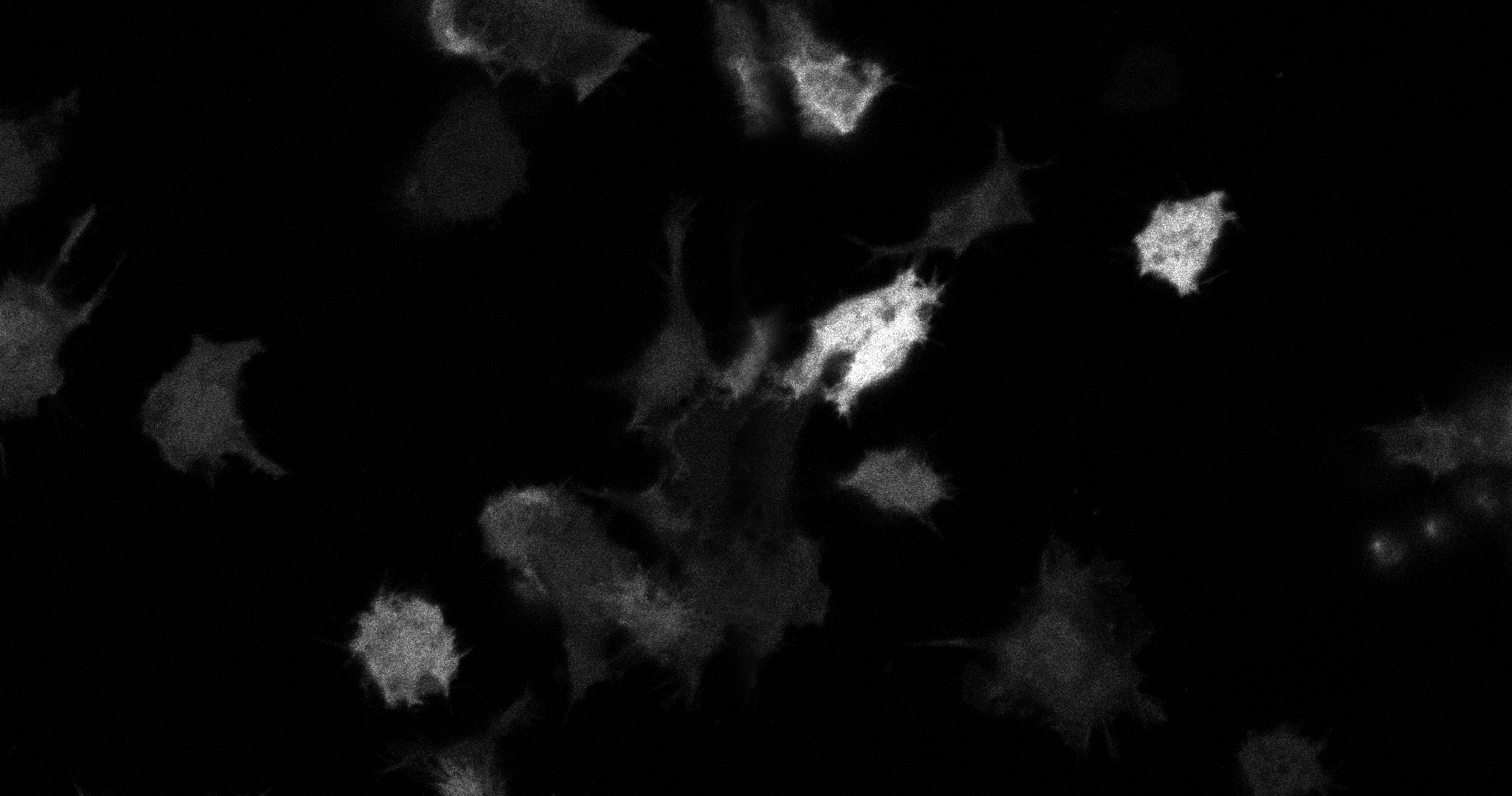

Supplement: Supplementary file 27 — Source Data for Figure 5 [file EMBJ-42-e113761-s005.zip › Figure 5/5A/CK-666/GFP + mCherry/Surface/(Green-GFP)-MAX_GFP-mCherry-CK-666-stacks 3-4.tif]

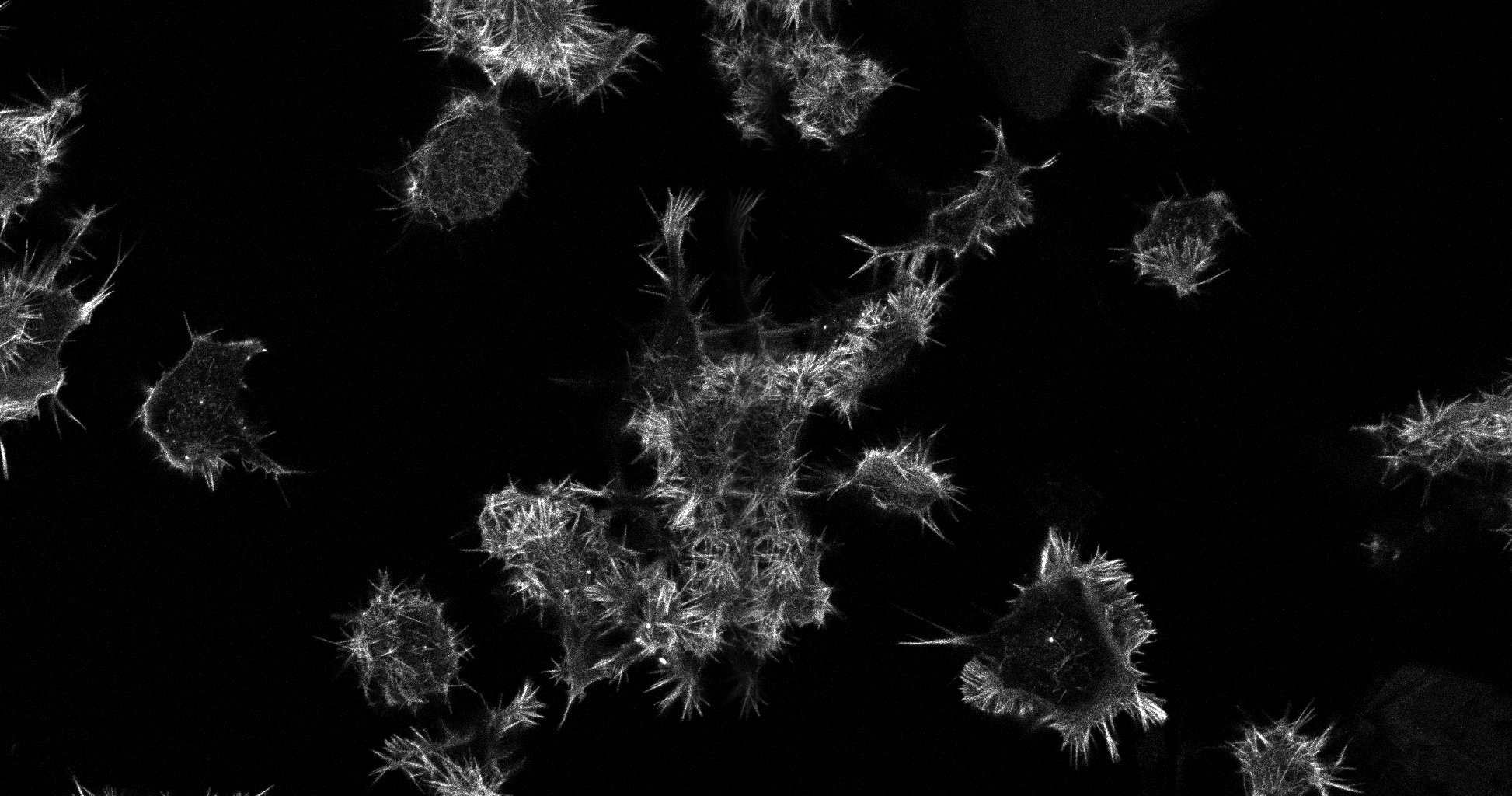

Supplement: Supplementary file 27 — Source Data for Figure 5 [file EMBJ-42-e113761-s005.zip › Figure 5/5A/CK-666/GFP + mCherry/Surface/(Grey-Phalloidin AF647)-MAX_GFP-mCherry-CK-666-stacks 3-4.tif]

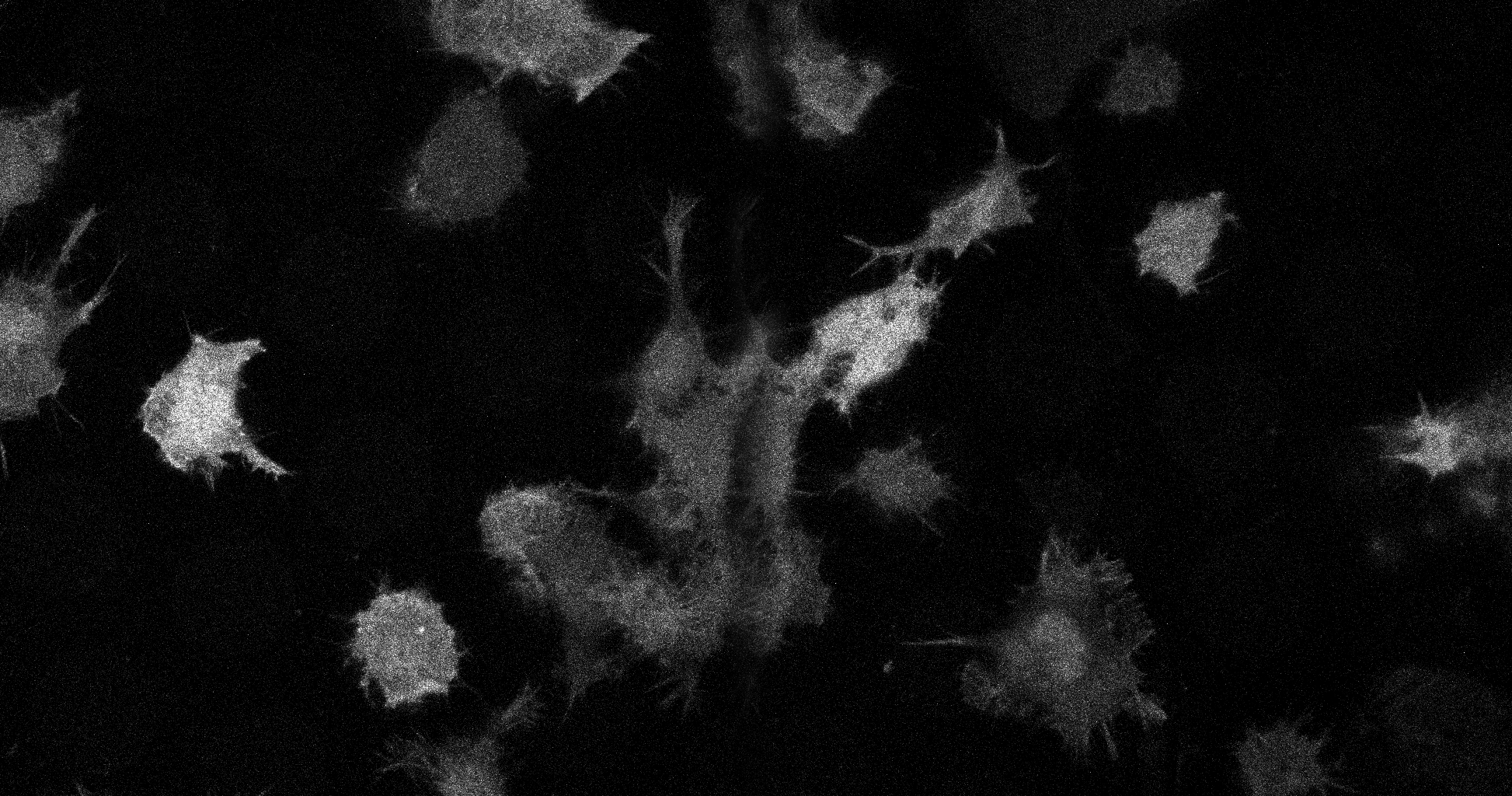

Supplement: Supplementary file 27 — Source Data for Figure 5 [file EMBJ-42-e113761-s005.zip › Figure 5/5A/CK-666/GFP + mCherry/Surface/(Red-mCherry)-MAX_GFP-mCherry-CK-666-stacks 3-4.tif]

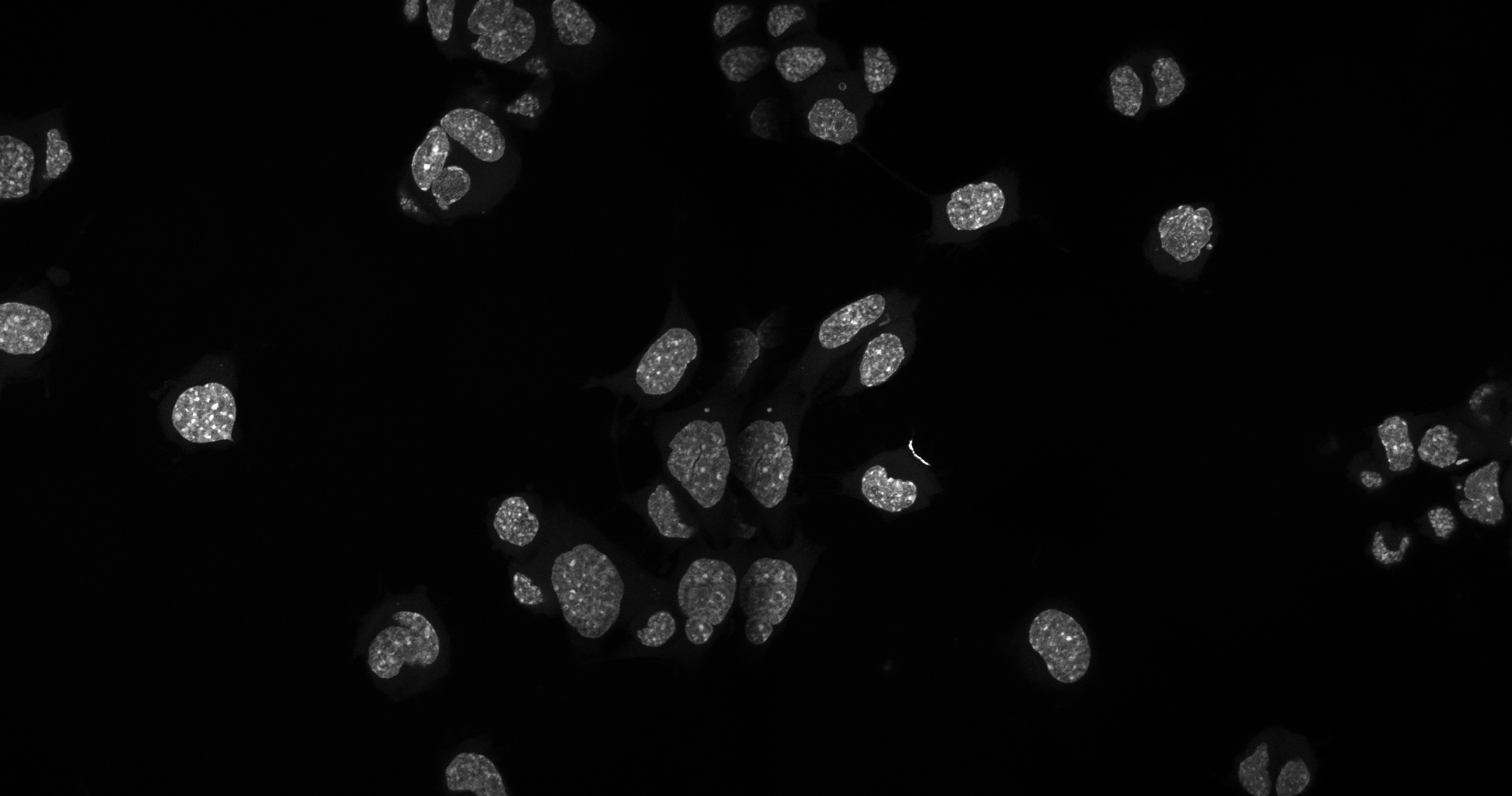

Supplement: Supplementary file 27 — Source Data for Figure 5 [file EMBJ-42-e113761-s005.zip › Figure 5/5A/CK-666/GFP + mCherry/Upper Stacks/(Blue-DAPI and Fibronectin AF405)-MAX_GFP-mCherry-CK-666-stacks 10-17.tif]

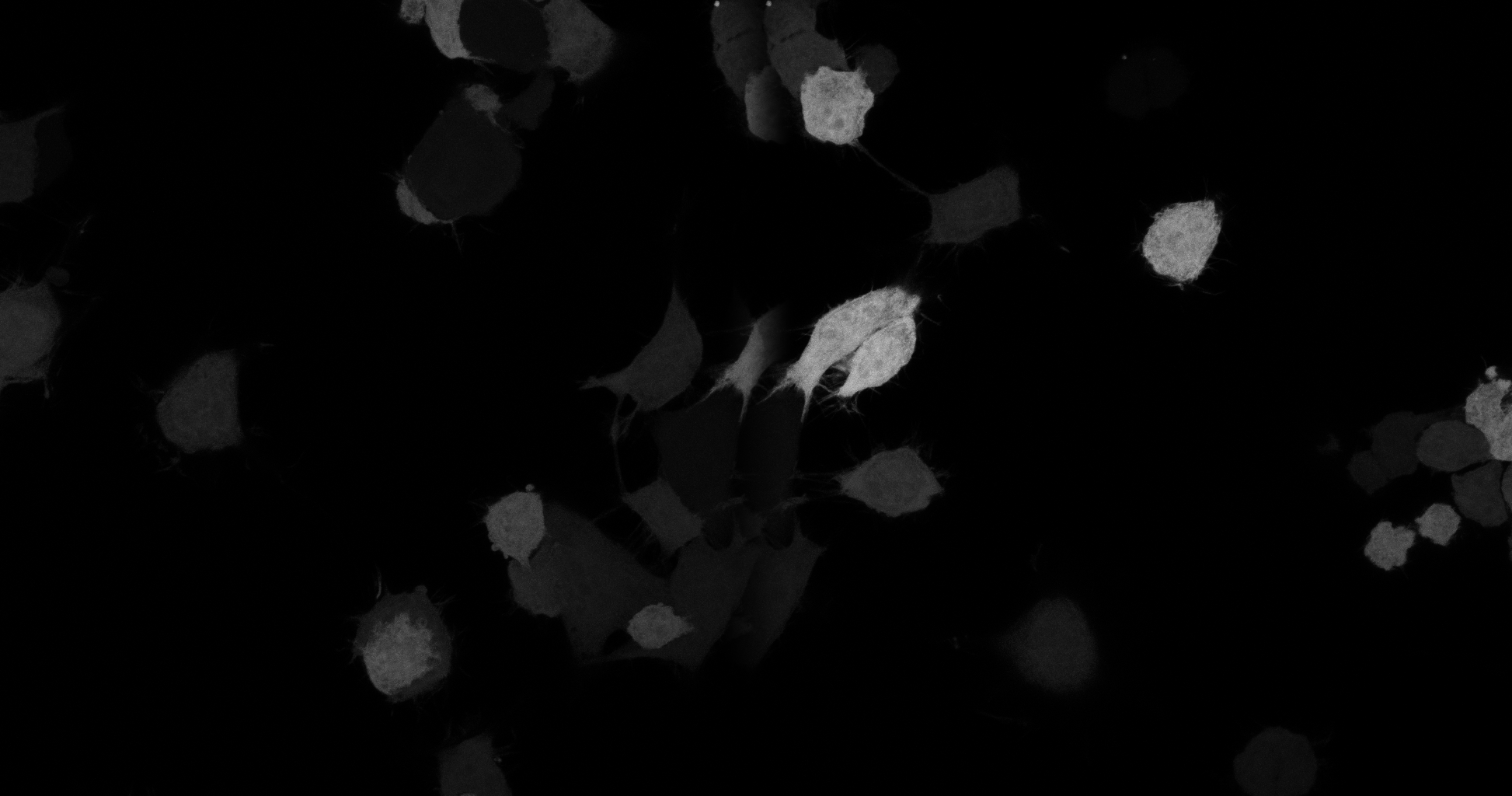

Supplement: Supplementary file 27 — Source Data for Figure 5 [file EMBJ-42-e113761-s005.zip › Figure 5/5A/CK-666/GFP + mCherry/Upper Stacks/(Green-GFP)-MAX_GFP-mCherry-CK-666-stacks 10-17.tif]

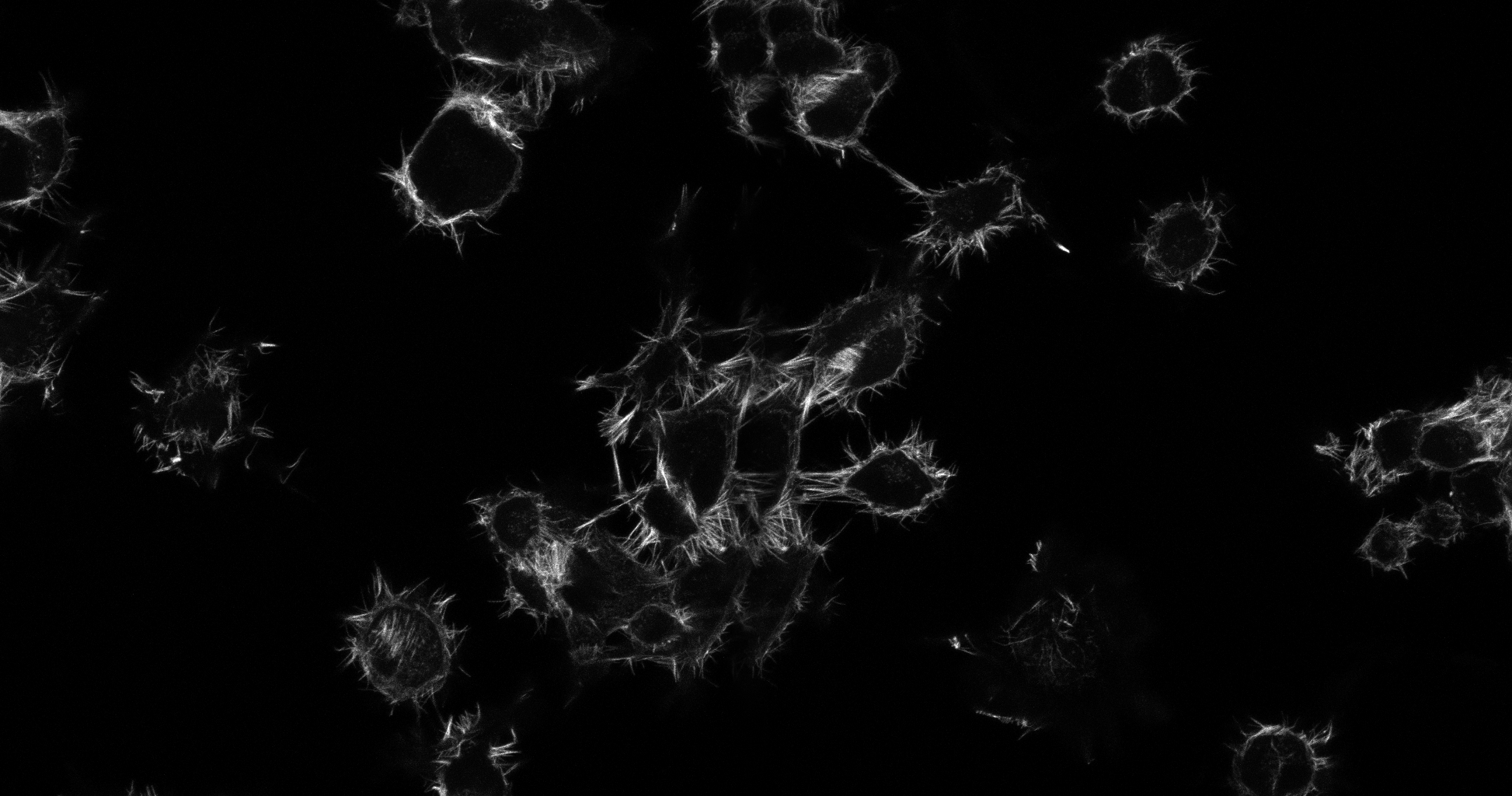

Supplement: Supplementary file 27 — Source Data for Figure 5 [file EMBJ-42-e113761-s005.zip › Figure 5/5A/CK-666/GFP + mCherry/Upper Stacks/(Grey-Phalloidin AF647)-MAX_GFP-mCherry-CK-666-stacks 10-17.tif]

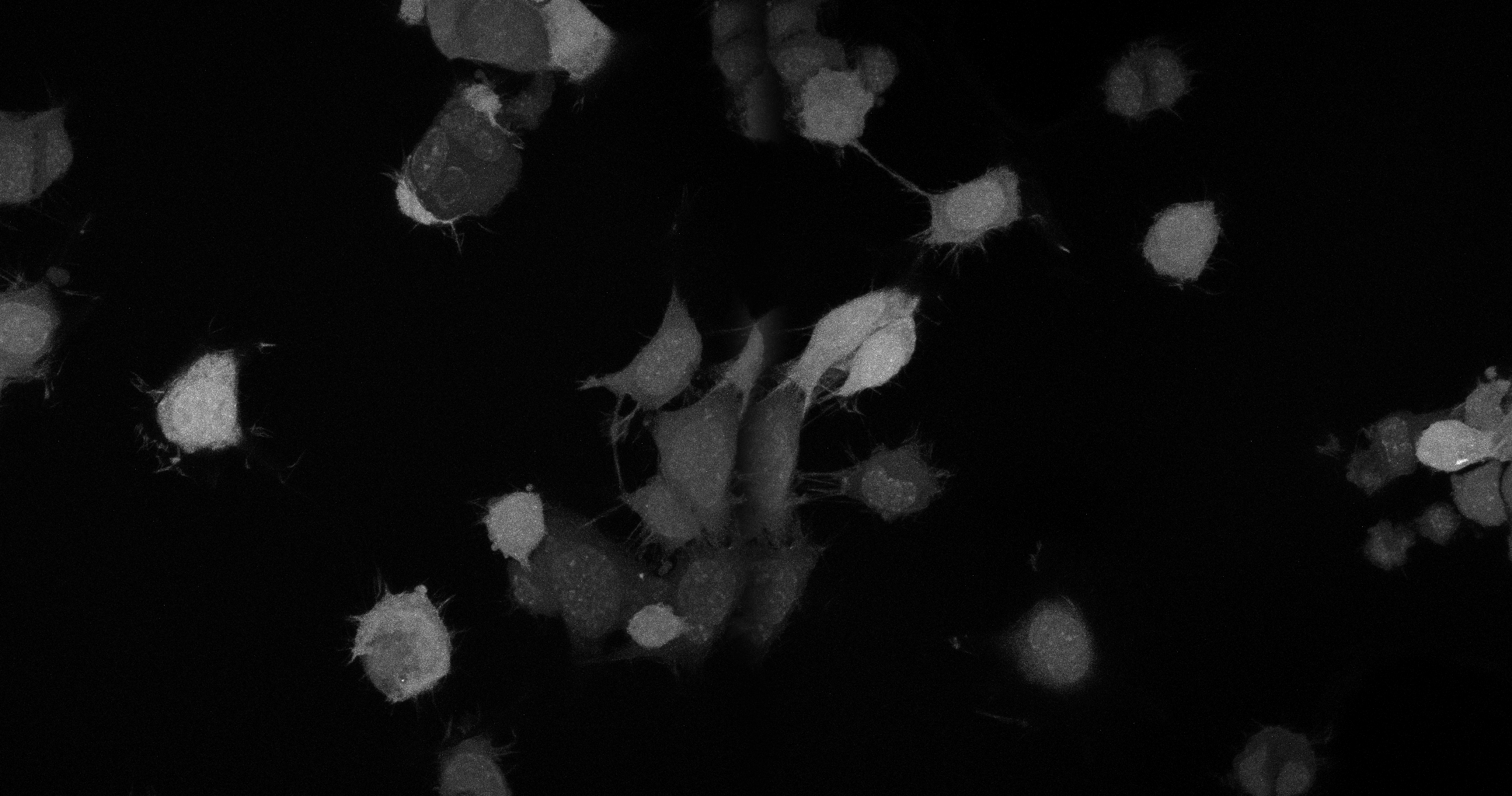

Supplement: Supplementary file 27 — Source Data for Figure 5 [file EMBJ-42-e113761-s005.zip › Figure 5/5A/CK-666/GFP + mCherry/Upper Stacks/(Red-mCherry)-MAX_GFP-mCherry-CK-666-stacks 10-17.tif]

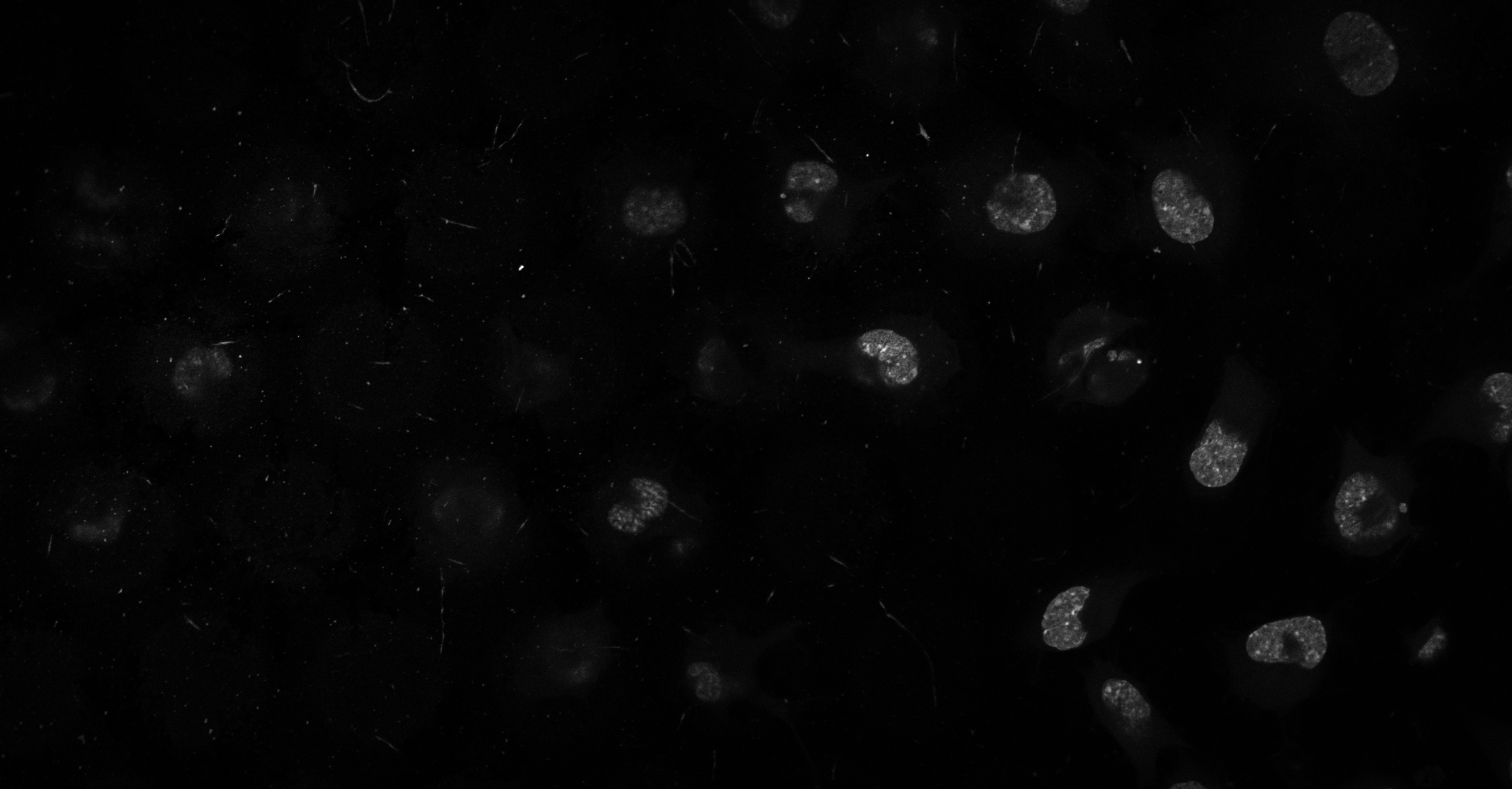

Supplement: Supplementary file 27 — Source Data for Figure 5 [file EMBJ-42-e113761-s005.zip › Figure 5/5A/DMSO/Eps8 + IRSp53/Surface/(Blue-DAPI and Fibronectin AF405)-MAX_Eps8deltaCAP-IRSp53-DMSO-stacks 6-7.tif]

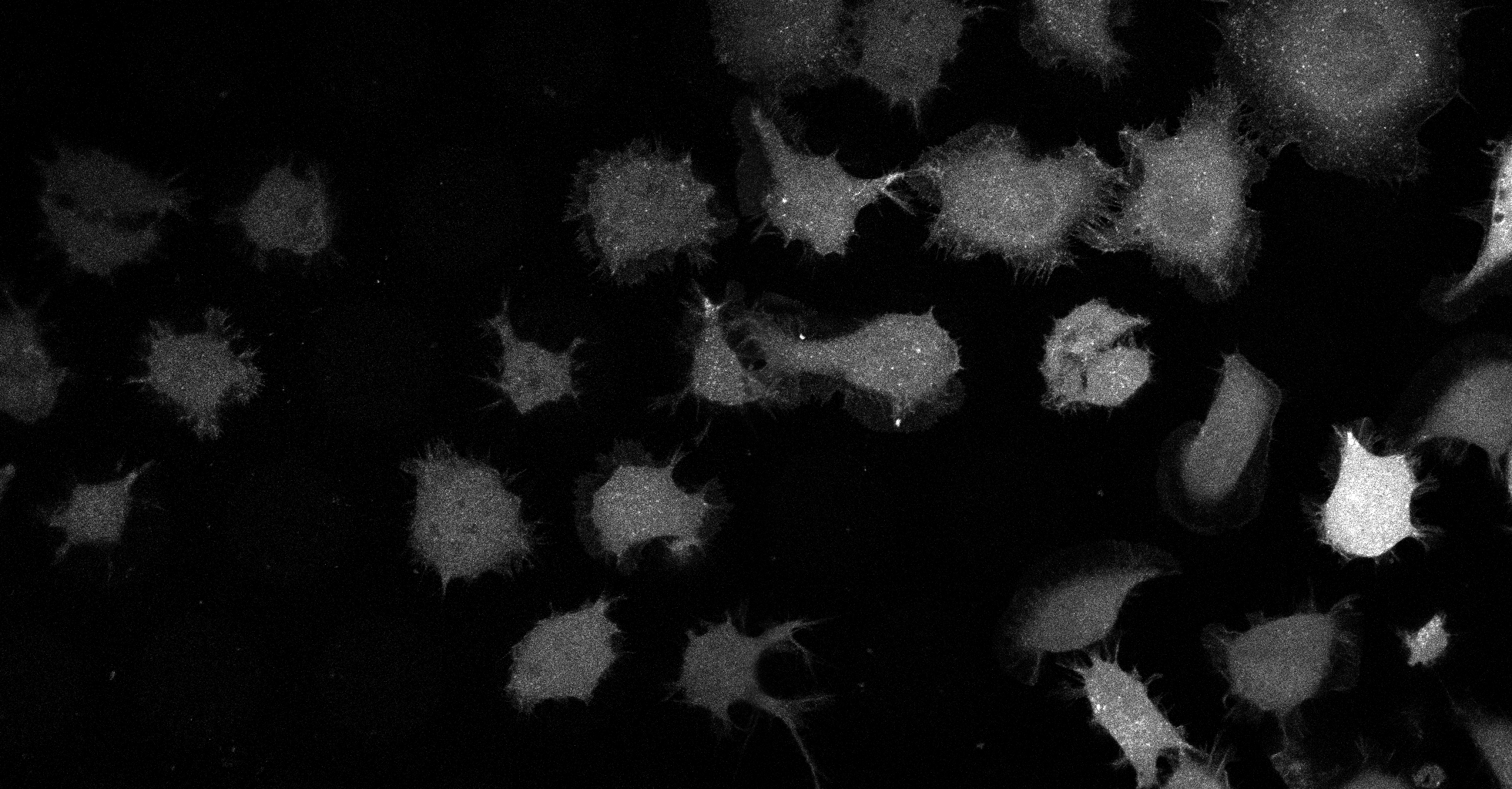

Supplement: Supplementary file 27 — Source Data for Figure 5 [file EMBJ-42-e113761-s005.zip › Figure 5/5A/DMSO/Eps8 + IRSp53/Surface/(Green-GFP-Eps8deltaCAP)-MAX_Eps8deltaCAP-IRSp53-DMSO-stacks 6-7.tif]

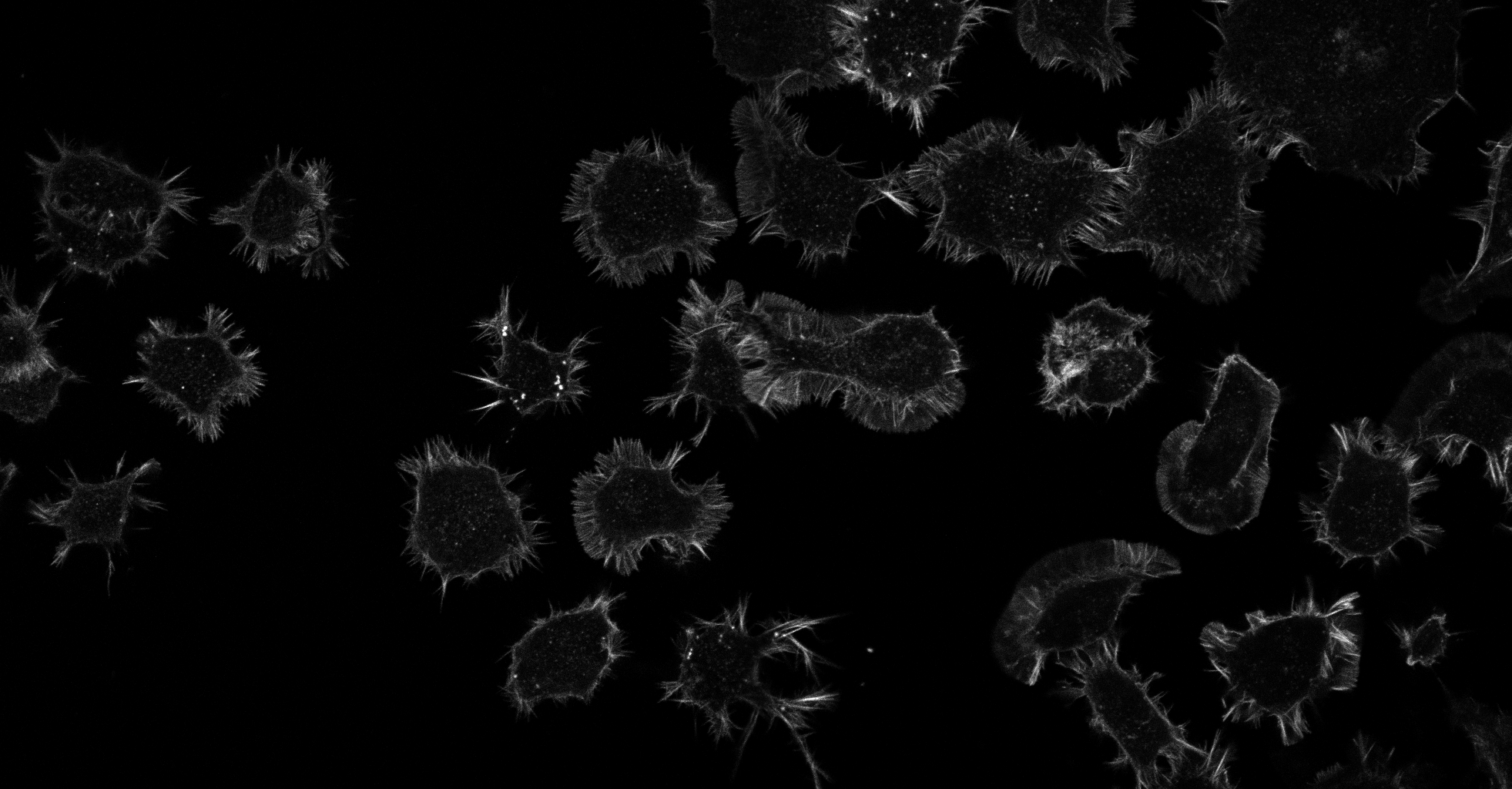

Supplement: Supplementary file 27 — Source Data for Figure 5 [file EMBJ-42-e113761-s005.zip › Figure 5/5A/DMSO/Eps8 + IRSp53/Surface/(Grey-Phalloidin AF647)-MAX_Eps8deltaCAP-IRSp53-DMSO-stacks 6-7.tif]

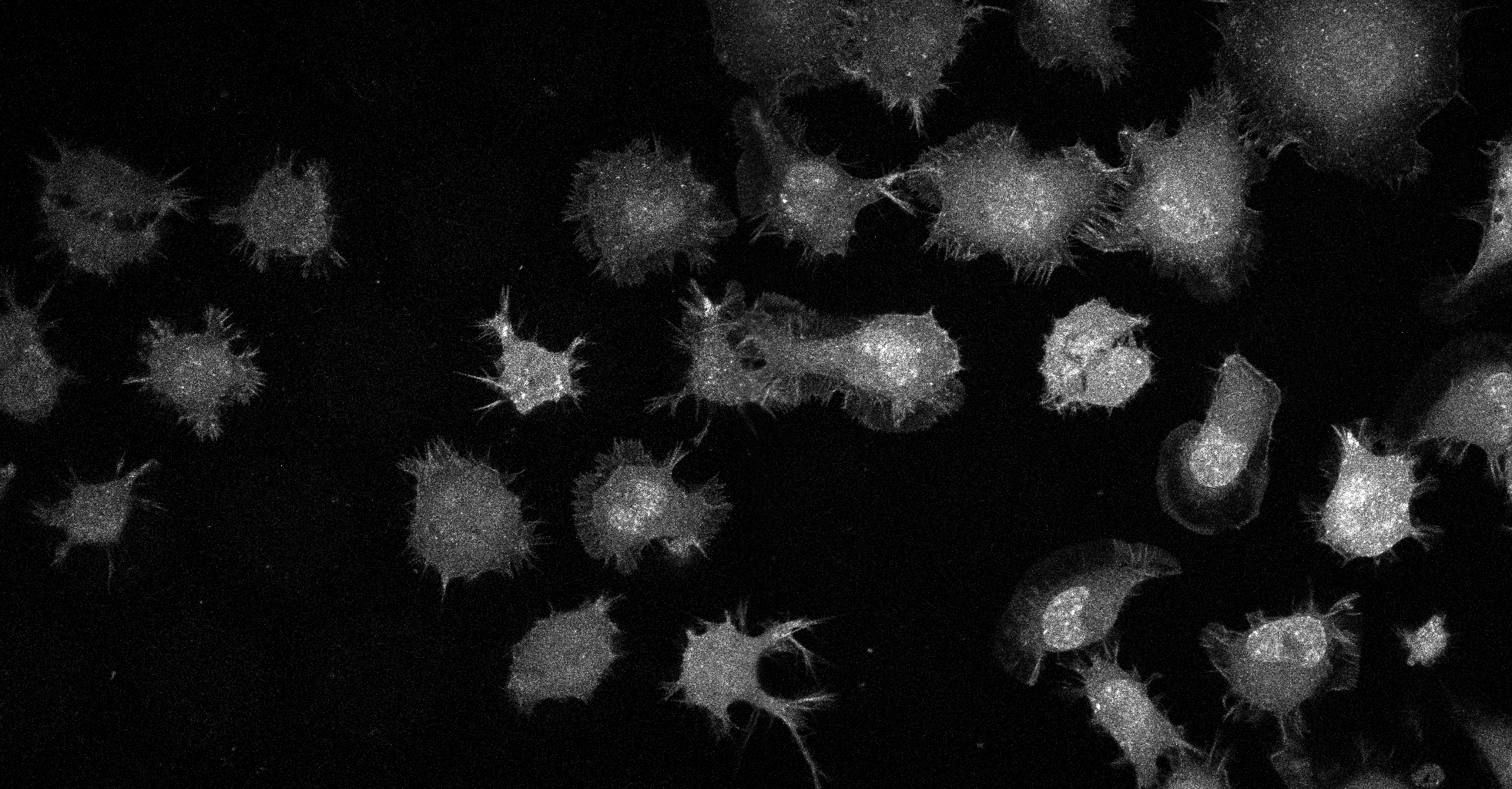

Supplement: Supplementary file 27 — Source Data for Figure 5 [file EMBJ-42-e113761-s005.zip › Figure 5/5A/DMSO/Eps8 + IRSp53/Surface/(Red-IRSp53-mCherry)-MAX_Eps8deltaCAP-IRSp53-DMSO-stacks 6-7.tif]

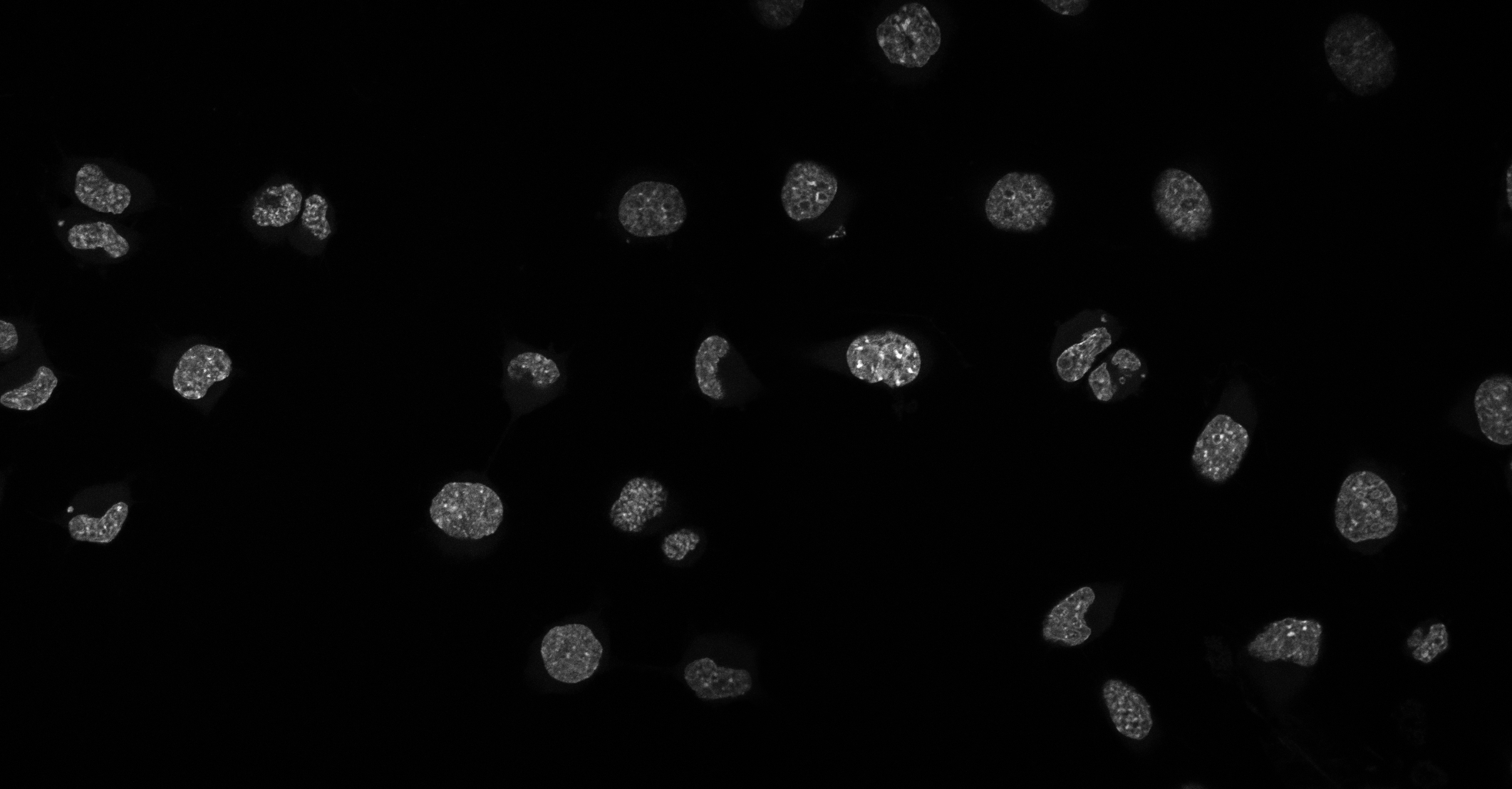

Supplement: Supplementary file 27 — Source Data for Figure 5 [file EMBJ-42-e113761-s005.zip › Figure 5/5A/DMSO/Eps8 + IRSp53/Upper Stacks/(Blue-DAPI and Fibronectin AF405)-MAX_Eps8deltaCAP-IRSp53-DMSO-stacks 11-13.tif]

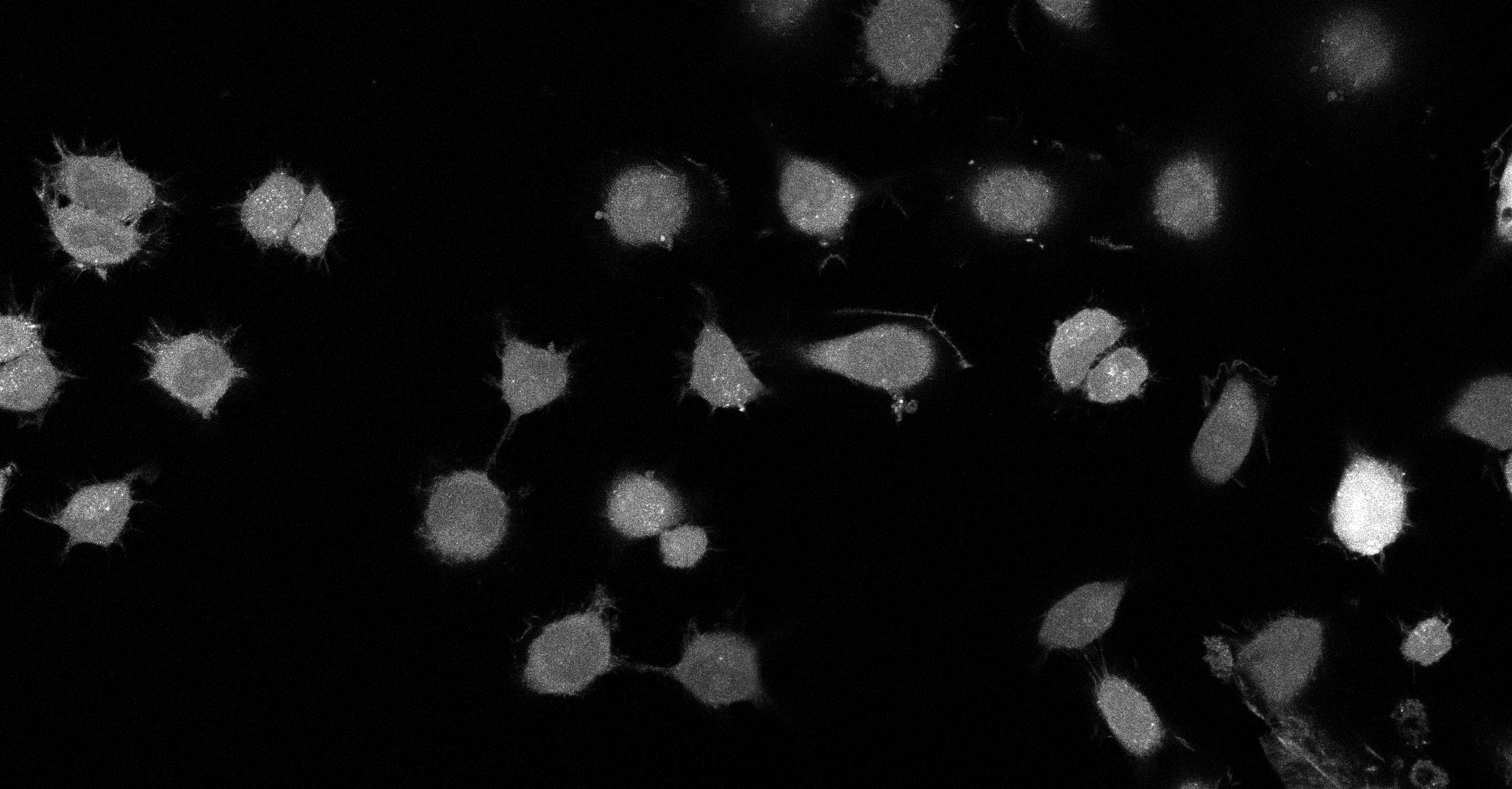

Supplement: Supplementary file 27 — Source Data for Figure 5 [file EMBJ-42-e113761-s005.zip › Figure 5/5A/DMSO/Eps8 + IRSp53/Upper Stacks/(Green-GFP-Eps8deltaCAP)-MAX_Eps8deltaCAP-IRSp53-DMSO-stacks 11-13.tif]

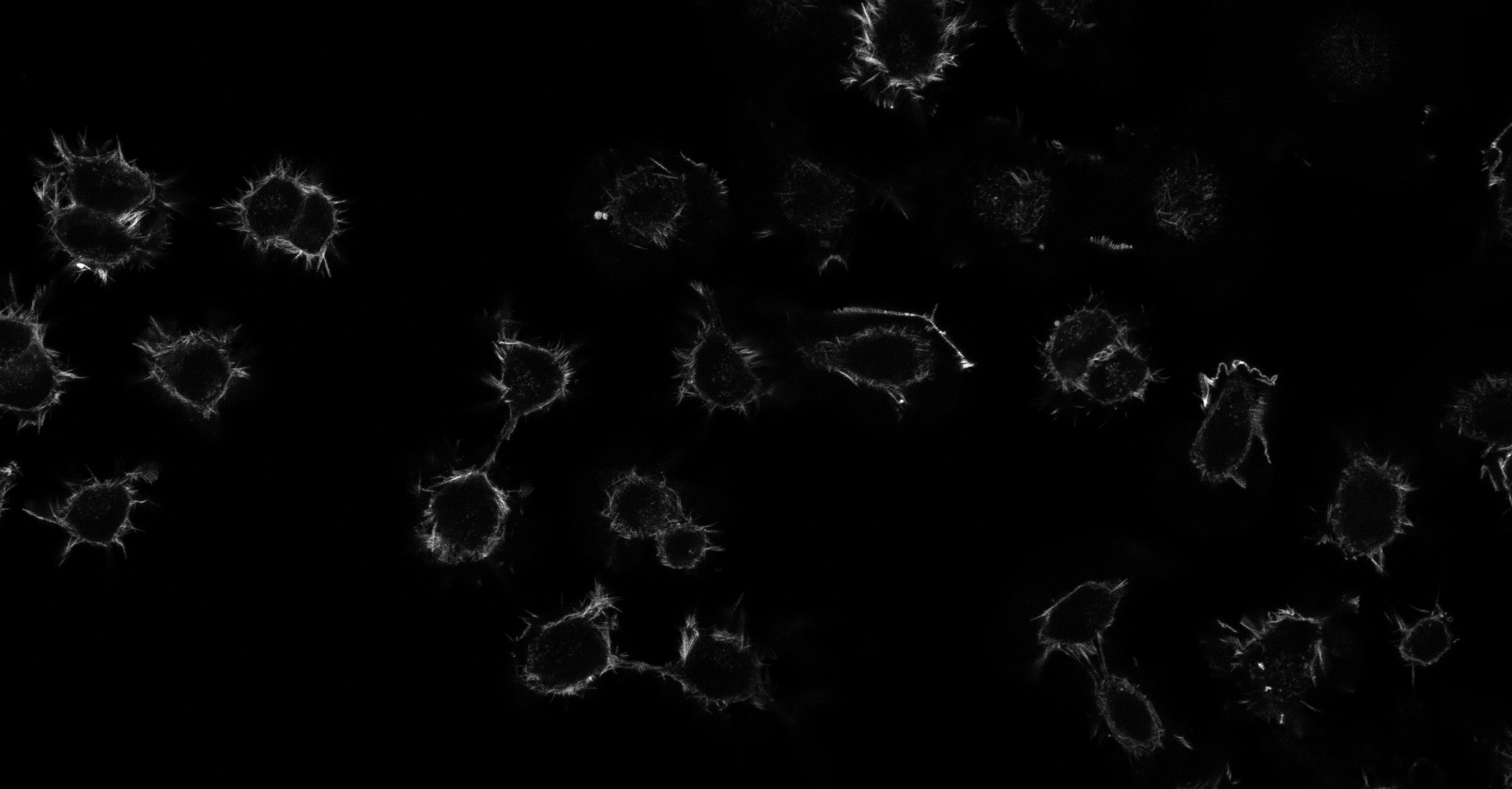

Supplement: Supplementary file 27 — Source Data for Figure 5 [file EMBJ-42-e113761-s005.zip › Figure 5/5A/DMSO/Eps8 + IRSp53/Upper Stacks/(Grey-Phalloidin AF647)-MAX_Eps8deltaCAP-IRSp53-DMSO-stacks 11-13.tif]

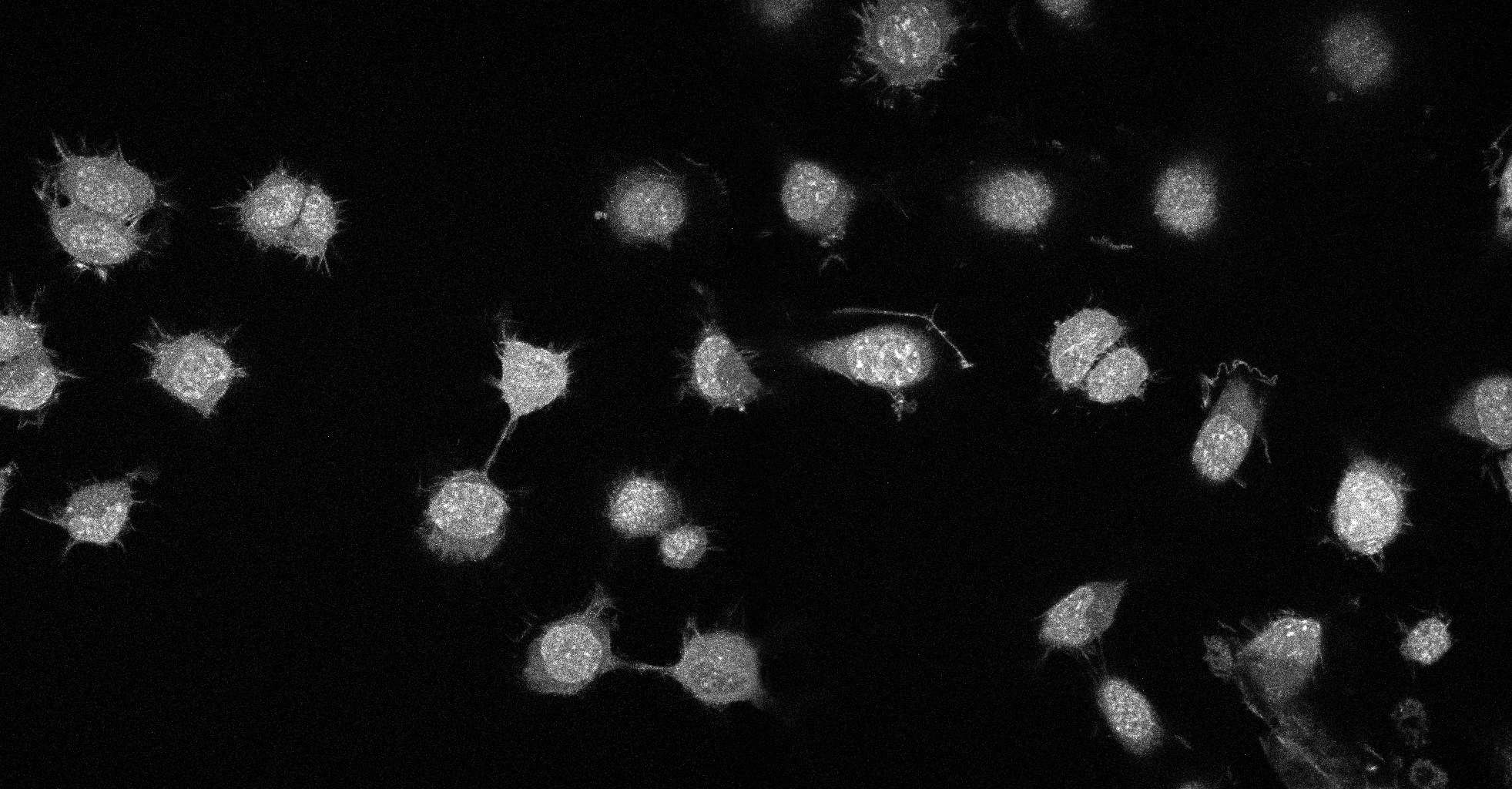

Supplement: Supplementary file 27 — Source Data for Figure 5 [file EMBJ-42-e113761-s005.zip › Figure 5/5A/DMSO/Eps8 + IRSp53/Upper Stacks/(Red-IRSp53-mCherry)-MAX_Eps8deltaCAP-IRSp53-DMSO-stacks 11-13.tif]

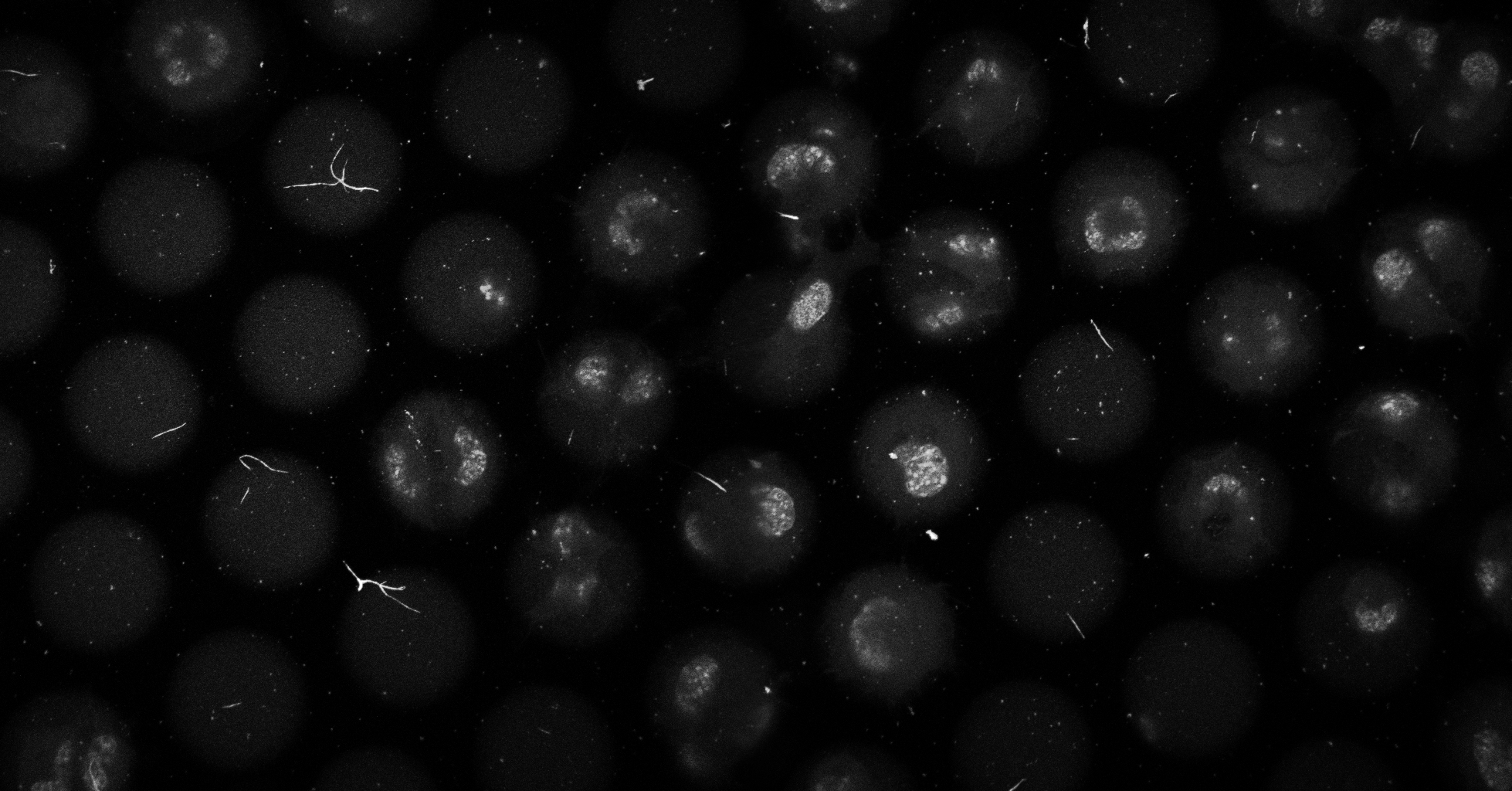

Supplement: Supplementary file 27 — Source Data for Figure 5 [file EMBJ-42-e113761-s005.zip › Figure 5/5A/DMSO/GFP + mCherry/Surface/(Blue-DAPI and Fibronectin AF405)-MAX_GFP-mCherry-DMSO-stacks 3-5.tif]

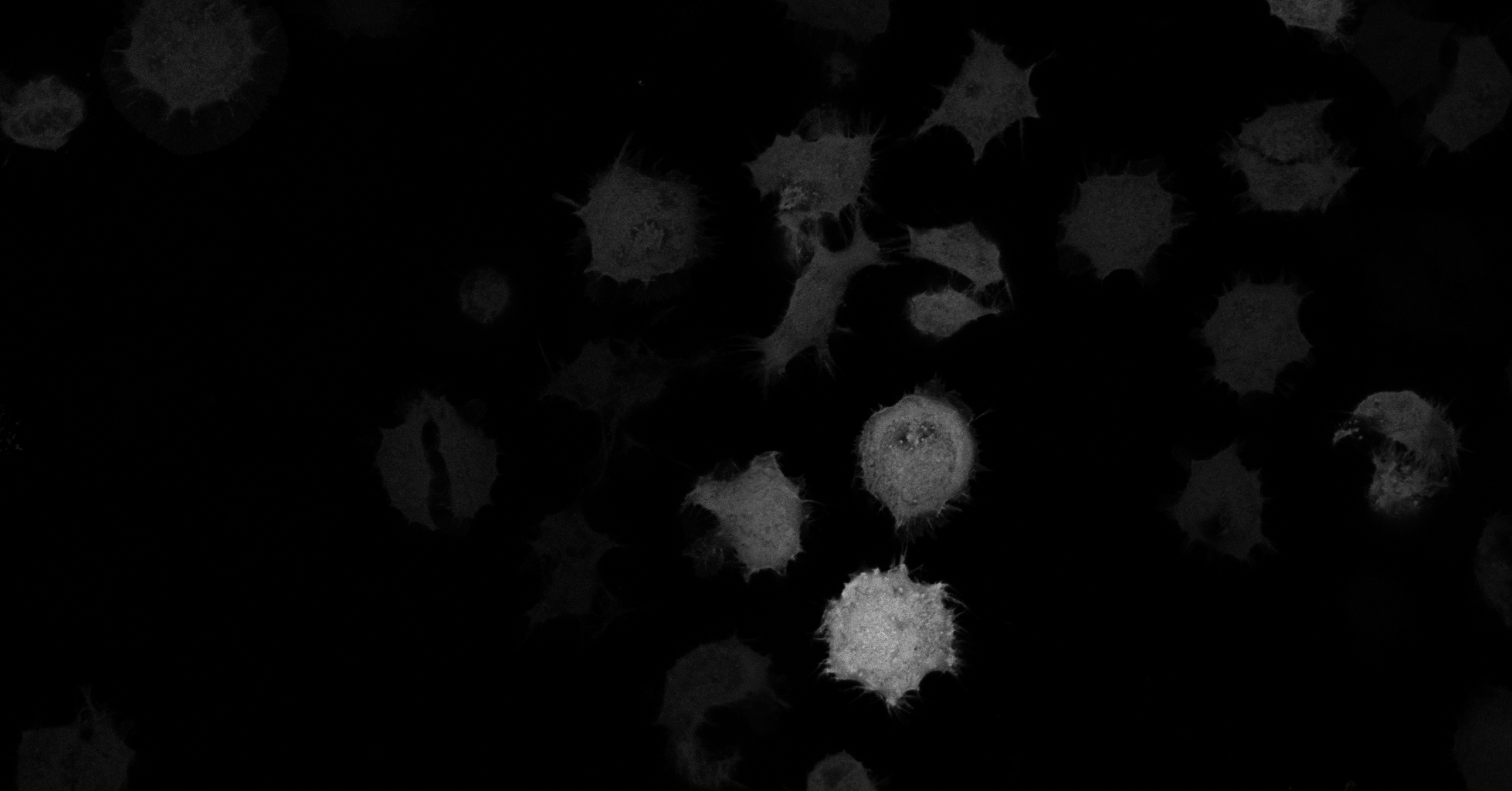

Supplement: Supplementary file 27 — Source Data for Figure 5 [file EMBJ-42-e113761-s005.zip › Figure 5/5A/DMSO/GFP + mCherry/Surface/(Green-GFP)-MAX_GFP-mCherry-DMSO-stacks 3-5.tif]

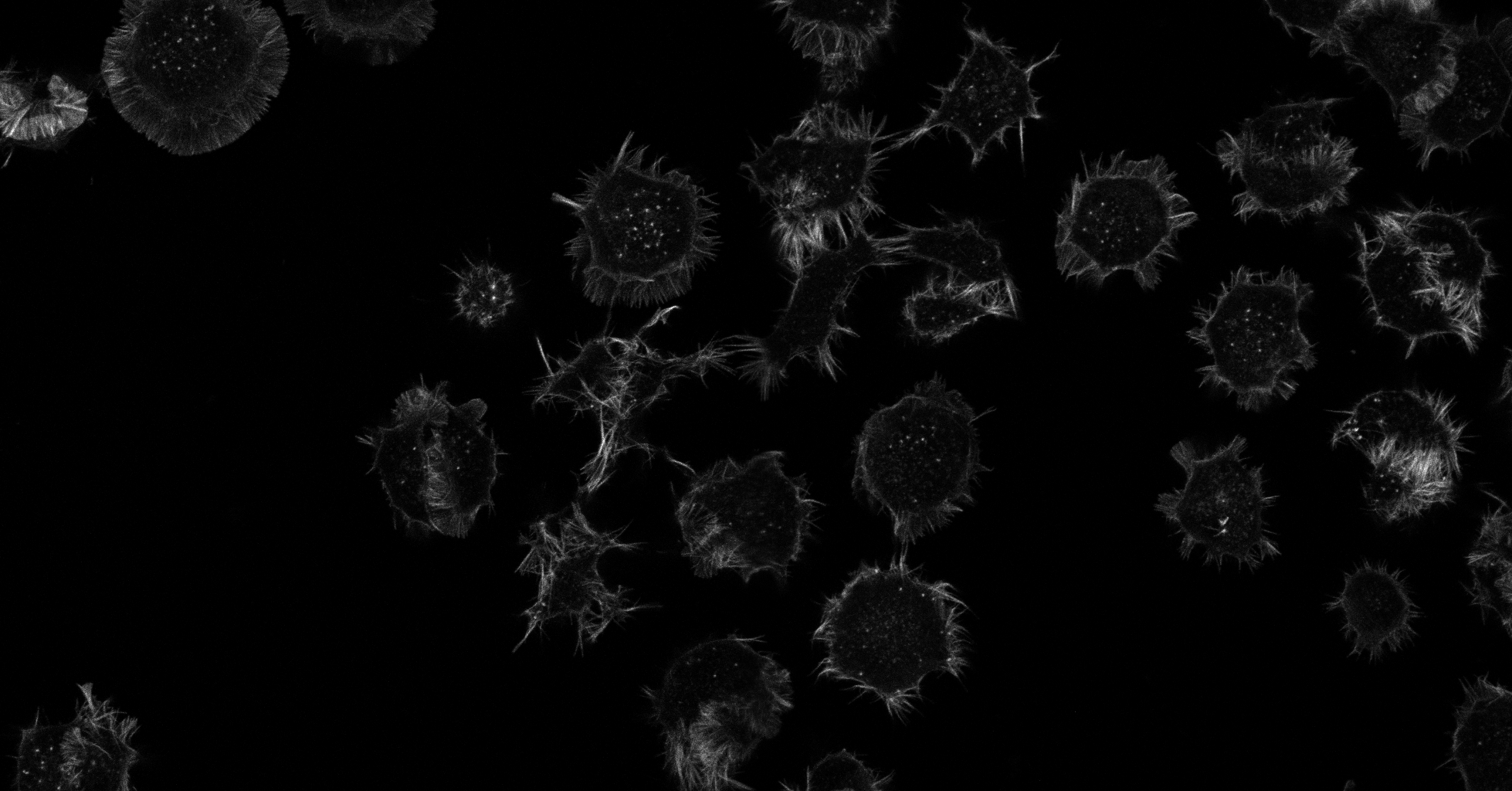

Supplement: Supplementary file 27 — Source Data for Figure 5 [file EMBJ-42-e113761-s005.zip › Figure 5/5A/DMSO/GFP + mCherry/Surface/(Grey-Phalloidin AF647)-MAX_GFP-mCherry-DMSO-stacks 3-5.tif]

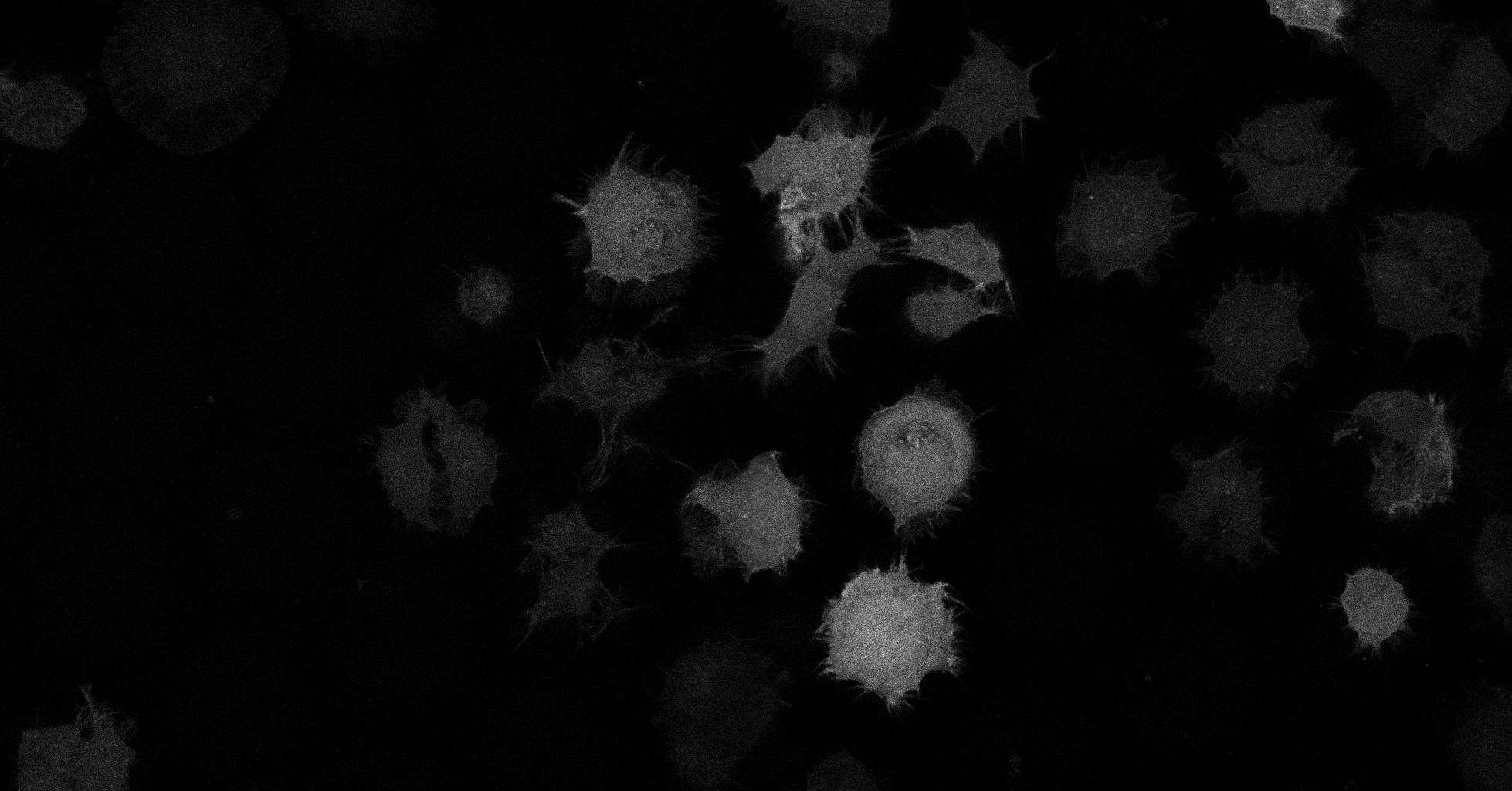

Supplement: Supplementary file 27 — Source Data for Figure 5 [file EMBJ-42-e113761-s005.zip › Figure 5/5A/DMSO/GFP + mCherry/Surface/(Red-mChery)-MAX_GFP-mCherry-DMSO-stacks 3-5.tif]

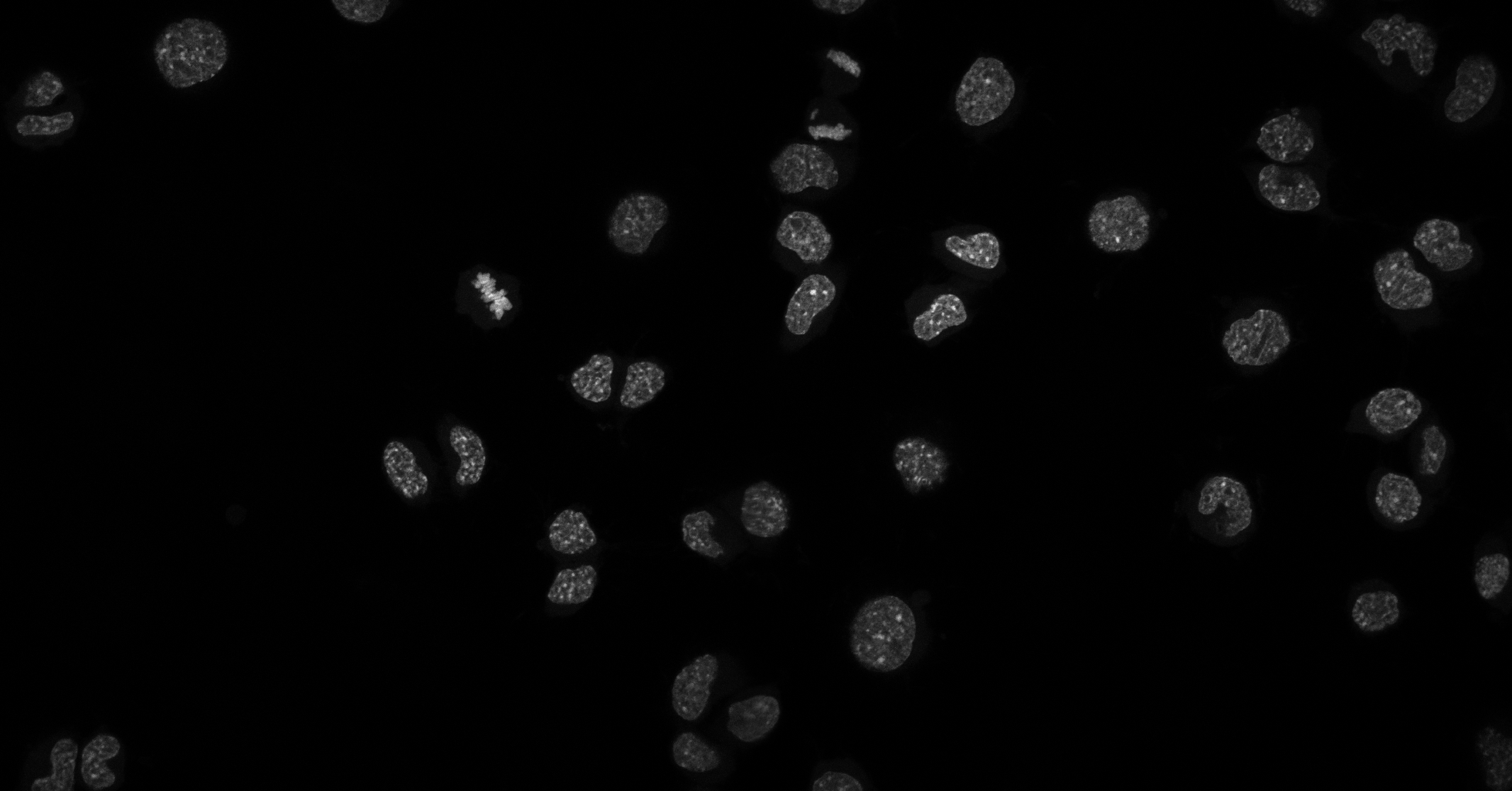

Supplement: Supplementary file 27 — Source Data for Figure 5 [file EMBJ-42-e113761-s005.zip › Figure 5/5A/DMSO/GFP + mCherry/Upper Stacks/(Blue-DAPI and Fibronectin AF405)-MAX_GFP-mCherry-DMSO-stacks 11-13.tif]

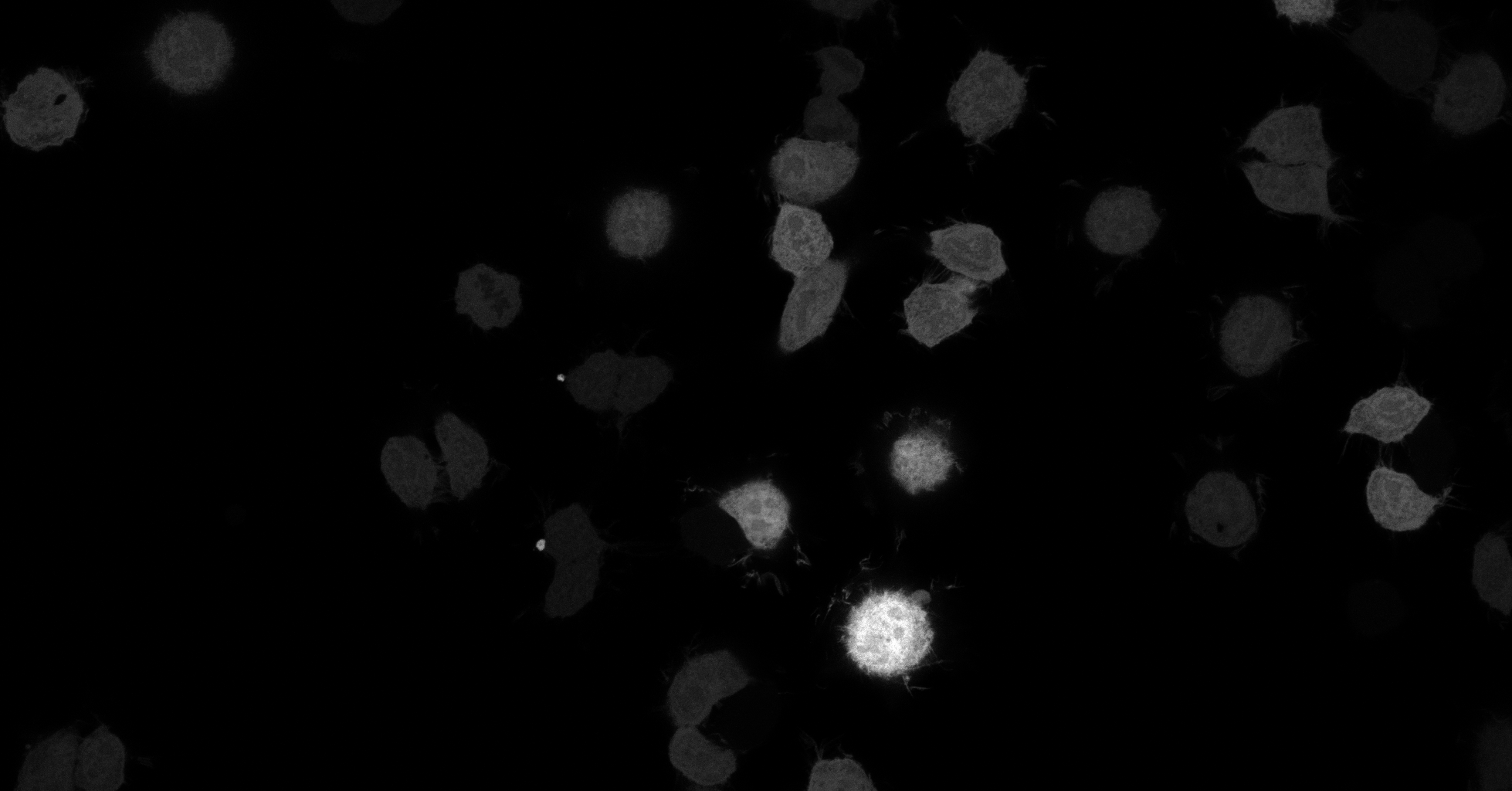

Supplement: Supplementary file 27 — Source Data for Figure 5 [file EMBJ-42-e113761-s005.zip › Figure 5/5A/DMSO/GFP + mCherry/Upper Stacks/(Green-GFP)-MAX_GFP-mCherry-DMSO-stacks 11-13.tif]
