# Supplementary figures and images for: Tunnelling nanotube formation is driven by Eps8/IRSp53‐dependent linear actin polymerization (part 2 of 2)
Source: EMBO J. 2023 Nov 27;42(24):e113761. doi: 10.15252/embj.2023113761 (PMC10711657; doi:10.15252/embj.2023113761)

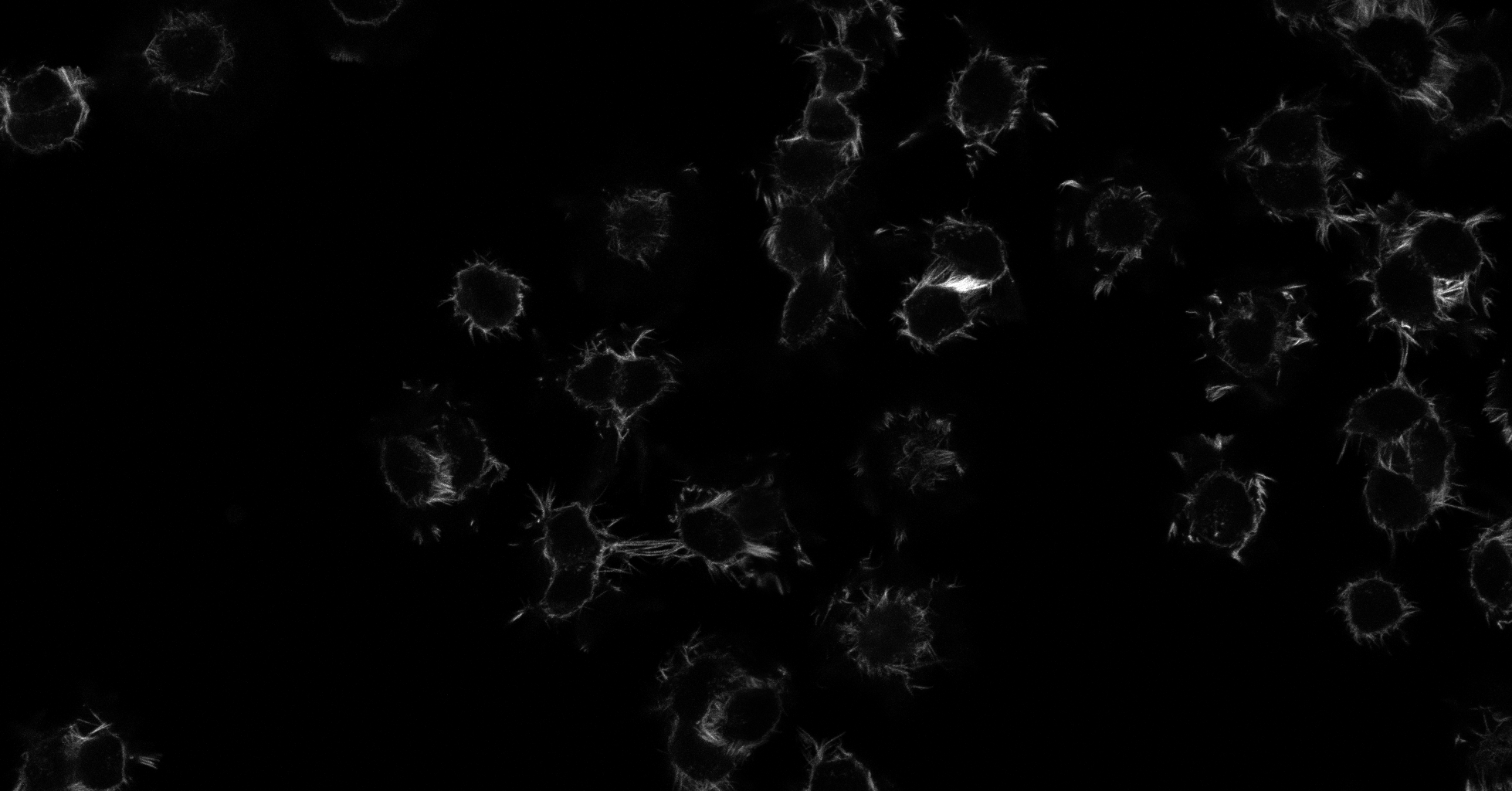

Supplement: Supplementary file 27 — Source Data for Figure 5 [file EMBJ-42-e113761-s005.zip › Figure 5/5A/DMSO/GFP + mCherry/Upper Stacks/(Grey-Phalloidin AF647)-MAX_GFP-mCherry-DMSO-stacks 11-13.tif]

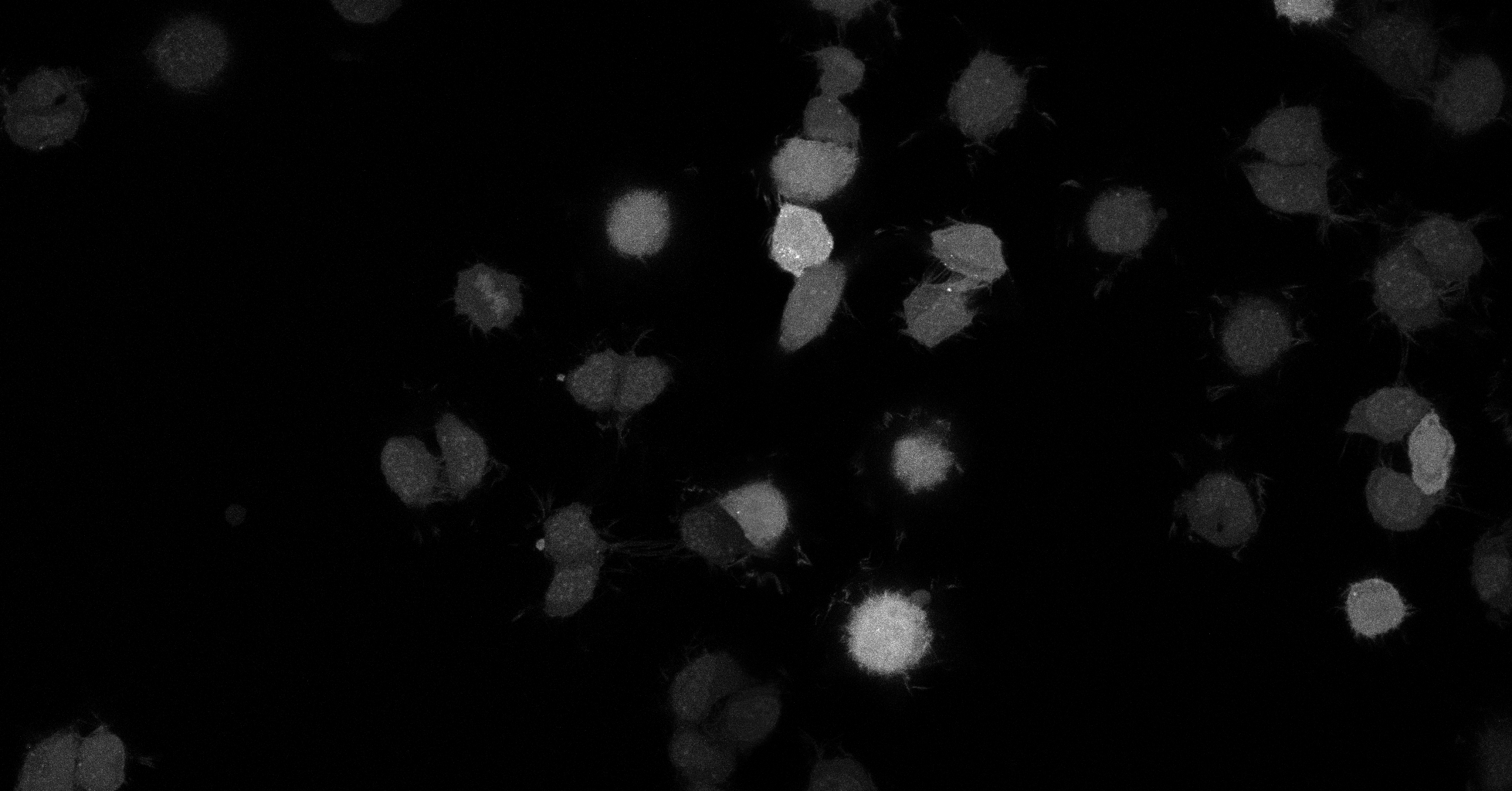

Supplement: Supplementary file 27 — Source Data for Figure 5 [file EMBJ-42-e113761-s005.zip › Figure 5/5A/DMSO/GFP + mCherry/Upper Stacks/(Red-mChery)-MAX_GFP-mCherry-DMSO-stacks 11-13.tif]

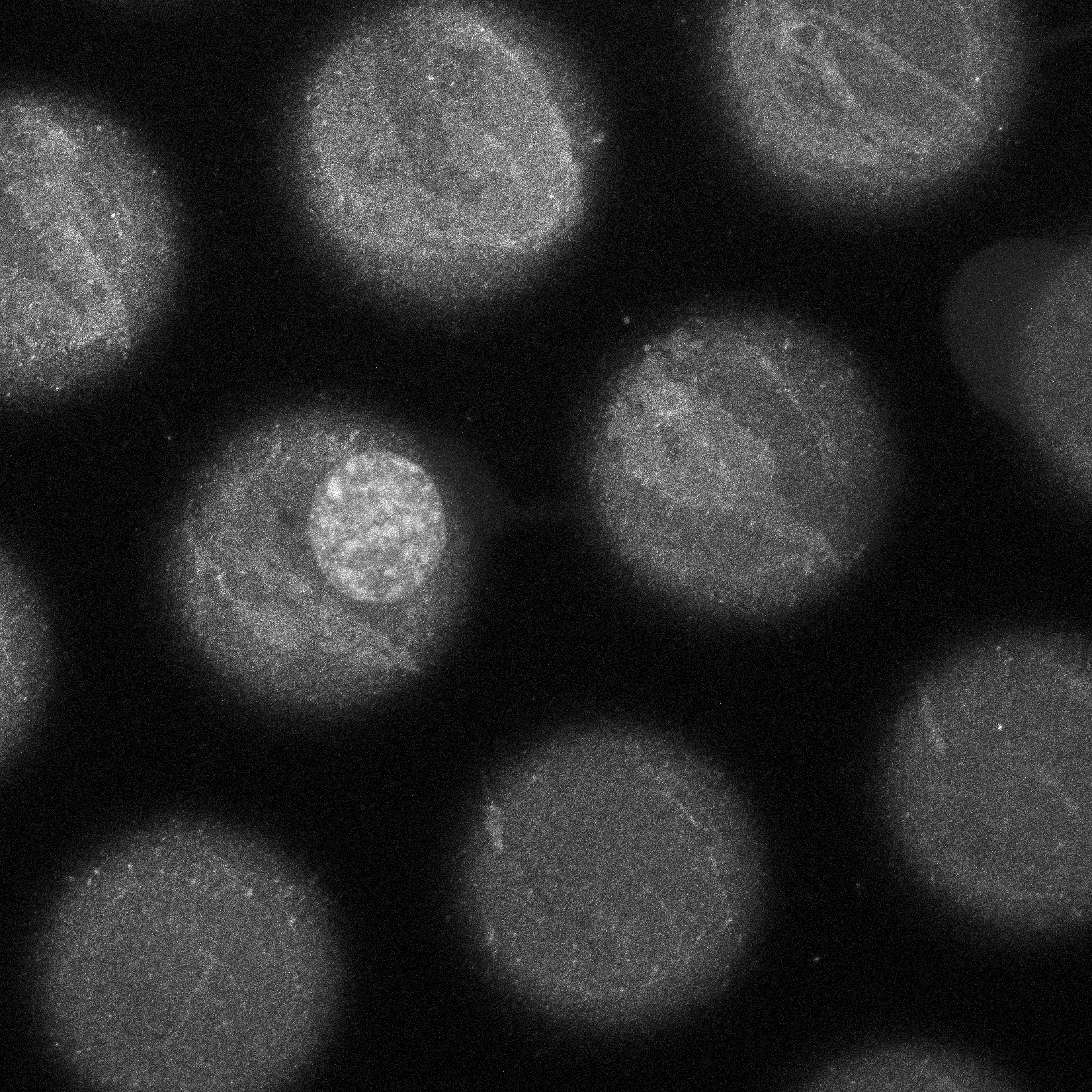

Supplement: Supplementary file 27 — Source Data for Figure 5 [file EMBJ-42-e113761-s005.zip › Figure 5/5C/CK-666/MAX_DiD-IRSp53mC-GFPEps8dcap-H2B BFP_AX405 FN_CoCulture.tif]

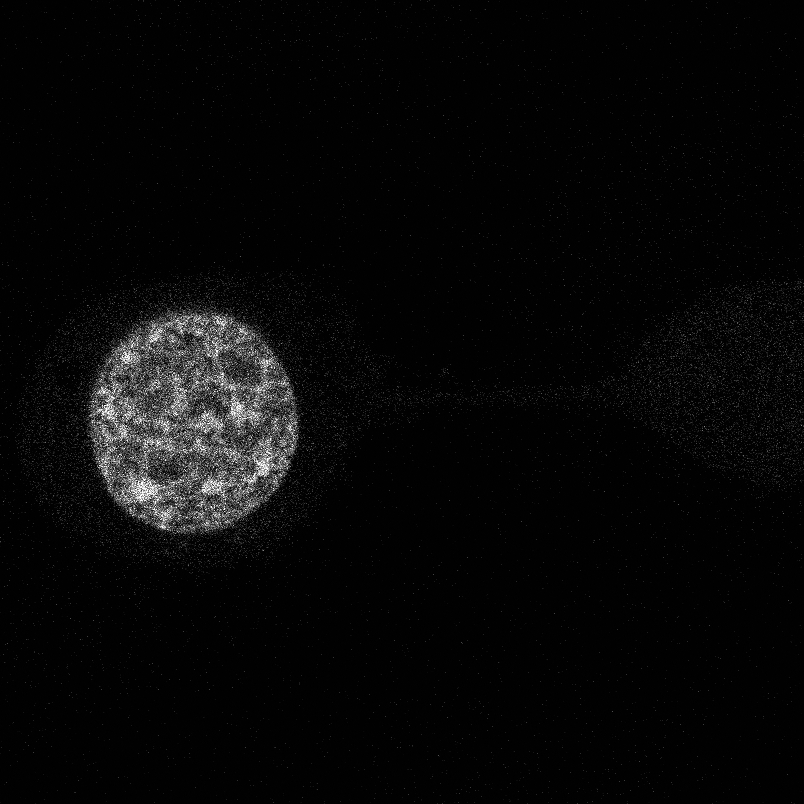

Supplement: Supplementary file 27 — Source Data for Figure 5 [file EMBJ-42-e113761-s005.zip › Figure 5/5C/CK-666/Subpanel-ii/XY.tif]

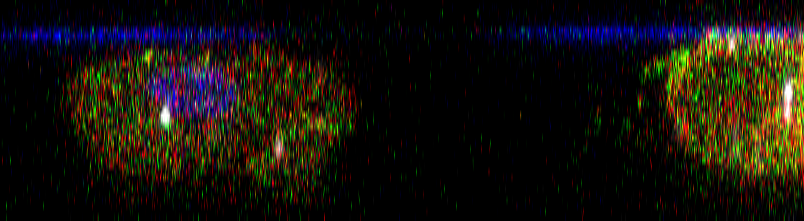

Supplement: Supplementary file 27 — Source Data for Figure 5 [file EMBJ-42-e113761-s005.zip › Figure 5/5C/CK-666/Subpanel-ii/XZ.tif]

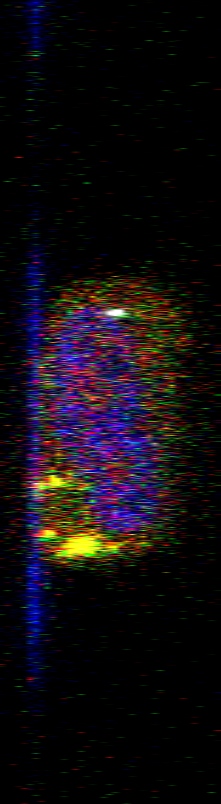

Supplement: Supplementary file 27 — Source Data for Figure 5 [file EMBJ-42-e113761-s005.zip › Figure 5/5C/CK-666/Subpanel-ii/YZ.tif]

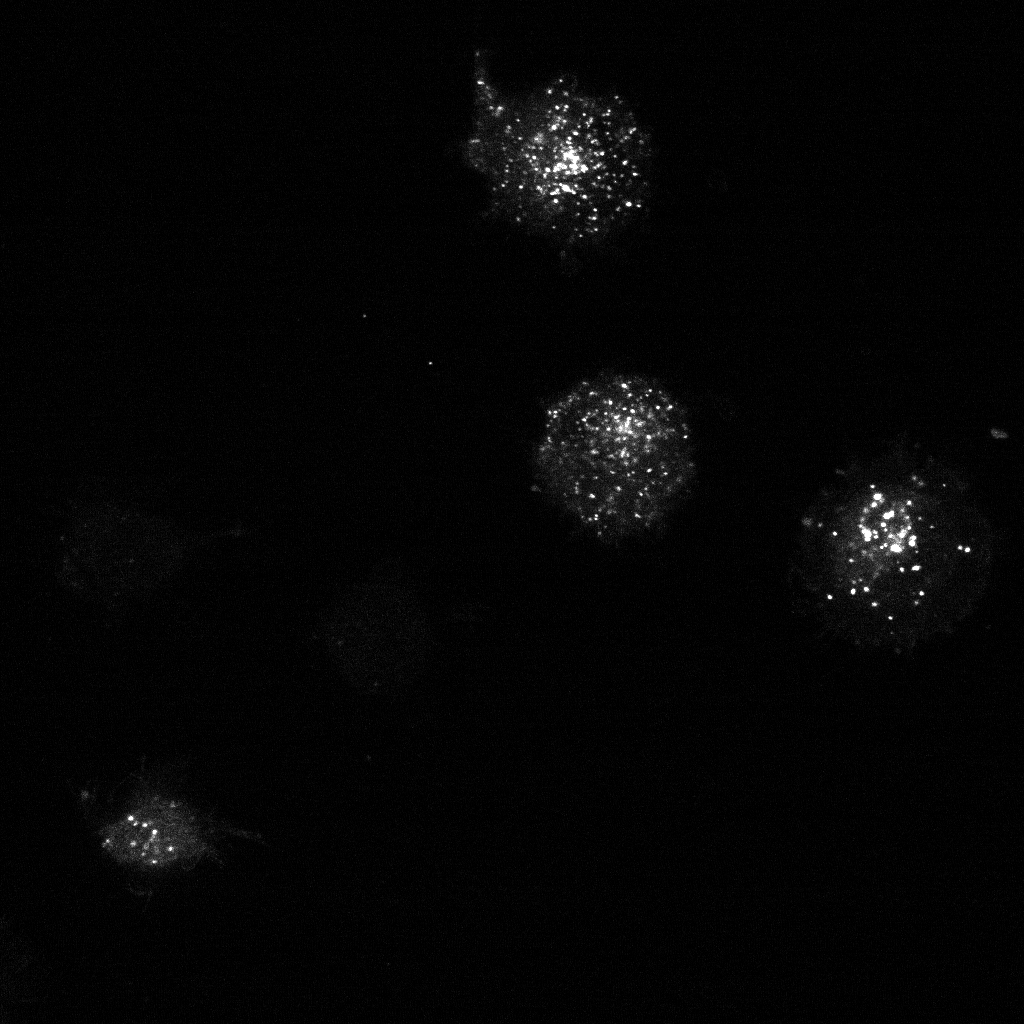

Supplement: Supplementary file 27 — Source Data for Figure 5 [file EMBJ-42-e113761-s005.zip › Figure 5/5C/DMSO/Surface/(DiD)_GFP-Eps8deltaCAP-IRSp53-mCherry_DMSO_stacks 4-5.tif]

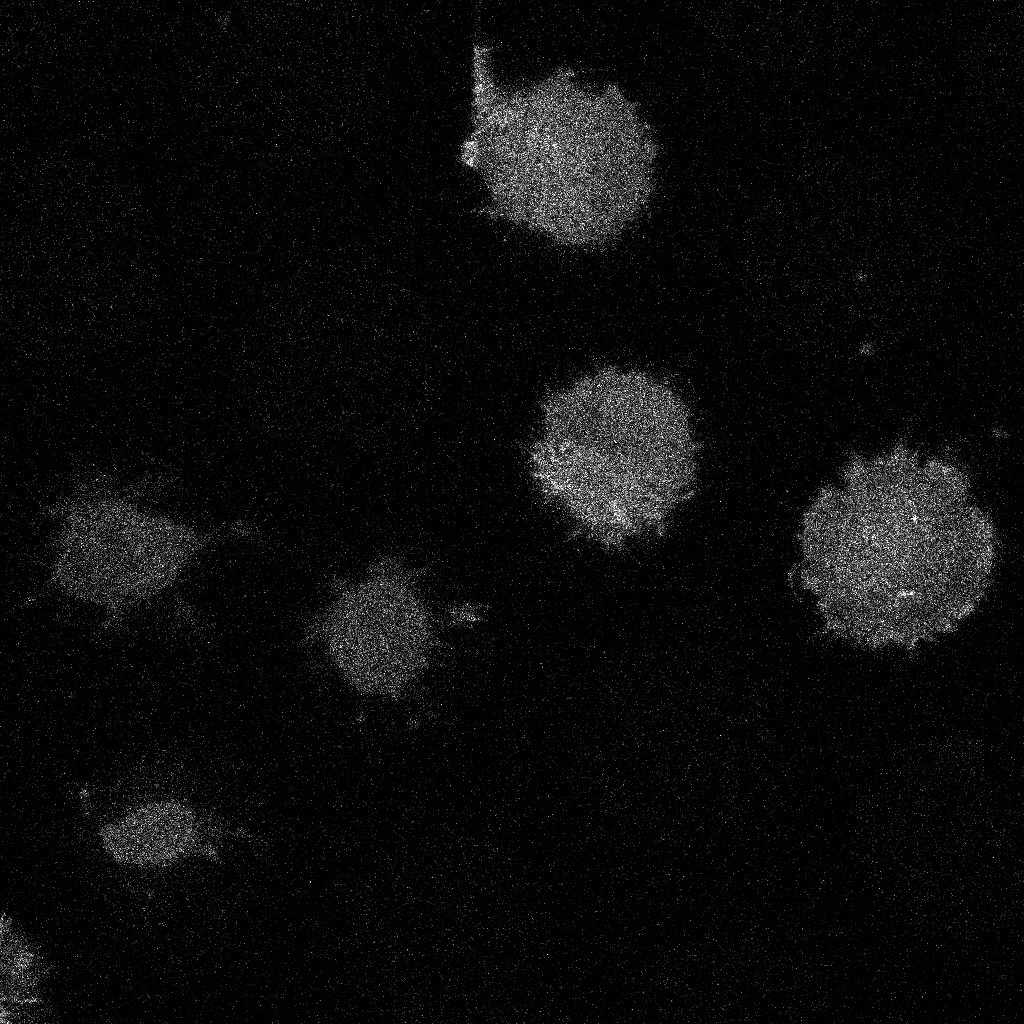

Supplement: Supplementary file 27 — Source Data for Figure 5 [file EMBJ-42-e113761-s005.zip › Figure 5/5C/DMSO/Surface/(GFP-Eps8deltaCAP)_GFP-Eps8deltaCAP-IRSp53-mCherry_DMSO_stacks 4-5.tif]

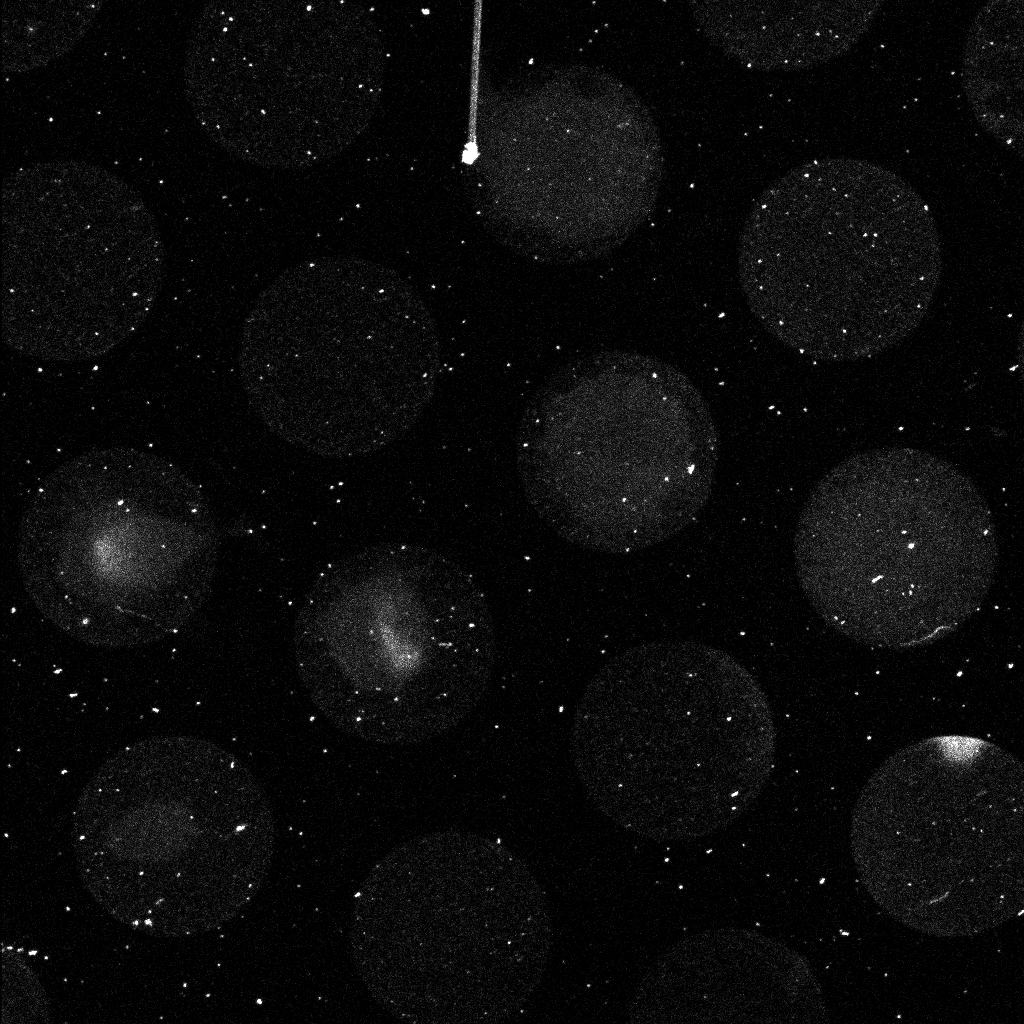

Supplement: Supplementary file 27 — Source Data for Figure 5 [file EMBJ-42-e113761-s005.zip › Figure 5/5C/DMSO/Surface/(H2B-BFP & Fibronectin AF 405)_GFP-Eps8deltaCAP-IRSp53-mCherry_DMSO_stacks 4-5.tif]

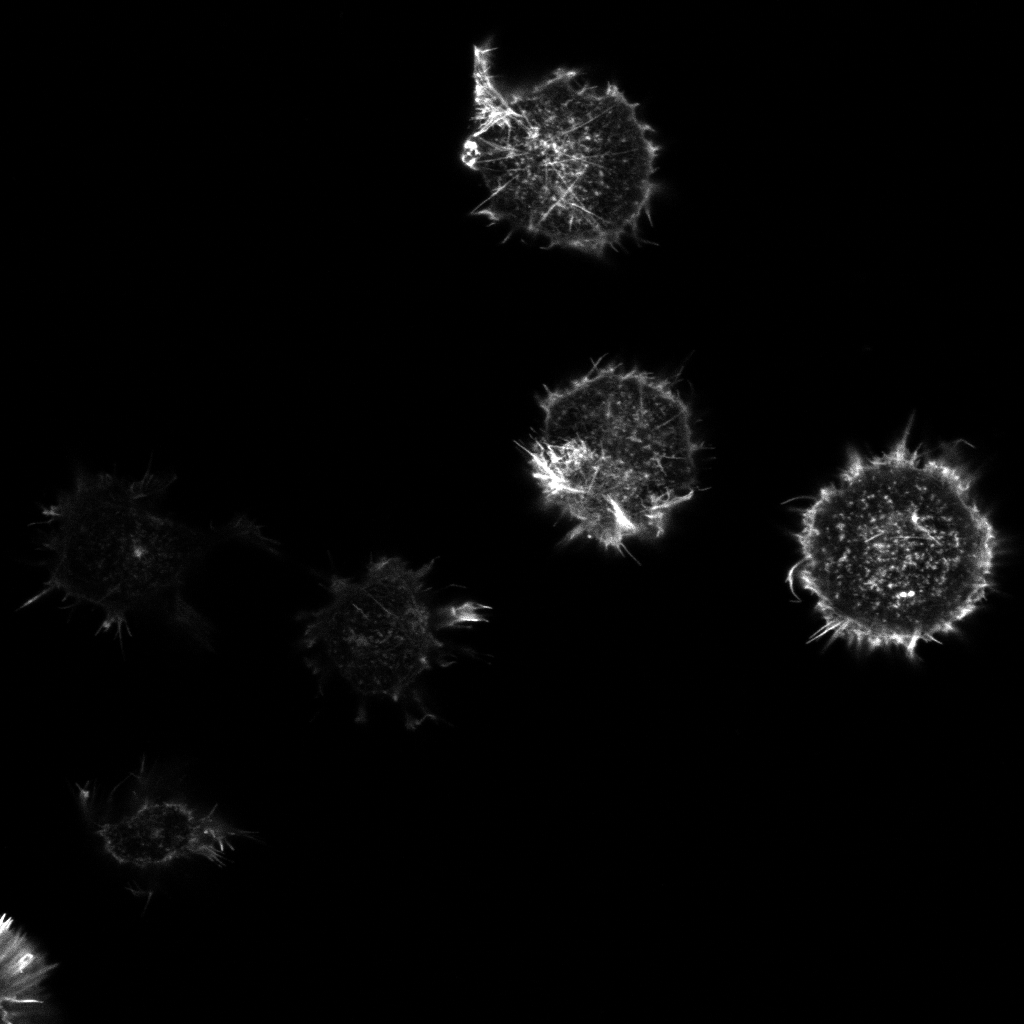

Supplement: Supplementary file 27 — Source Data for Figure 5 [file EMBJ-42-e113761-s005.zip › Figure 5/5C/DMSO/Surface/(Rhodamine Phalloidin & IRSp53-mCherry)_GFP-Eps8deltaCAP-IRSp53-mCherry_DMSO_stacks 4-5.tif]

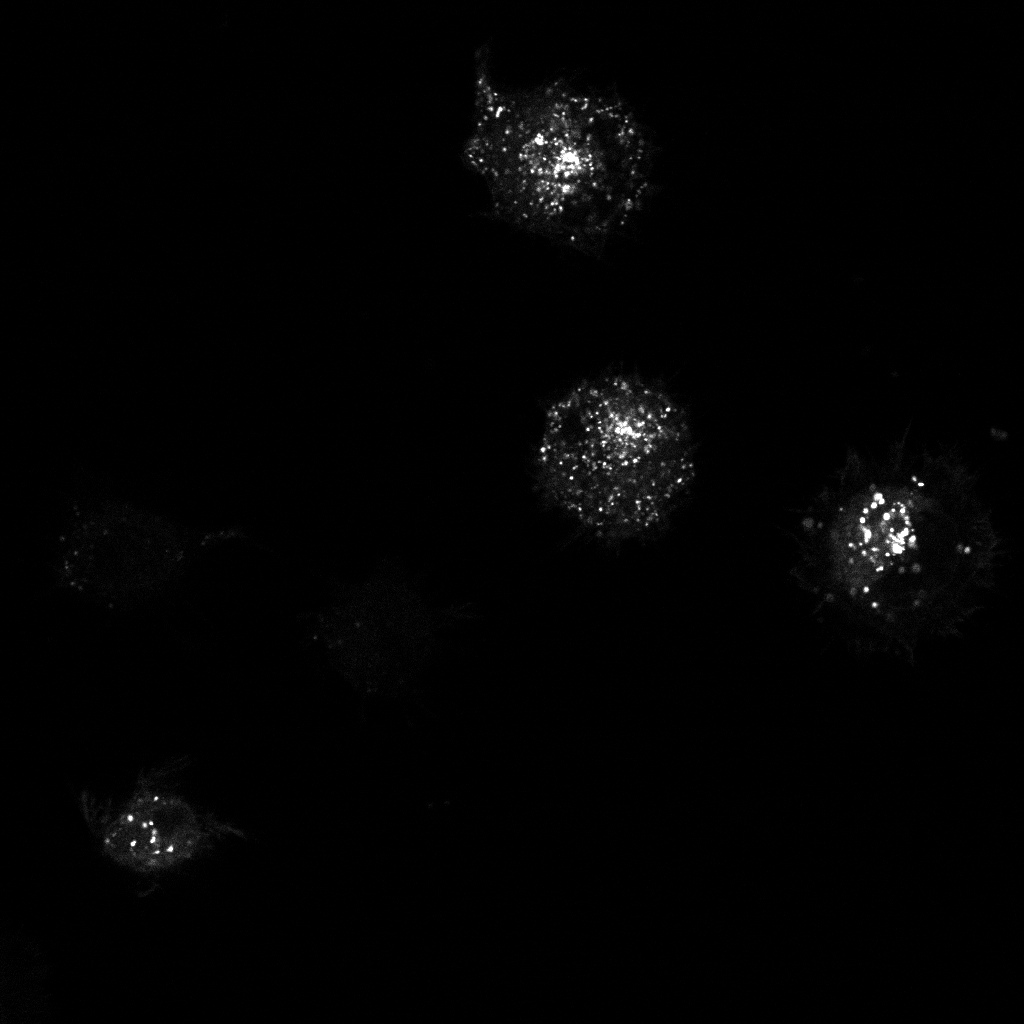

Supplement: Supplementary file 27 — Source Data for Figure 5 [file EMBJ-42-e113761-s005.zip › Figure 5/5C/DMSO/Upper stacks/(DiD)_GFP-Eps8deltaCAP-IRSp53-mCherry_DMSO_stacks 8.tif]

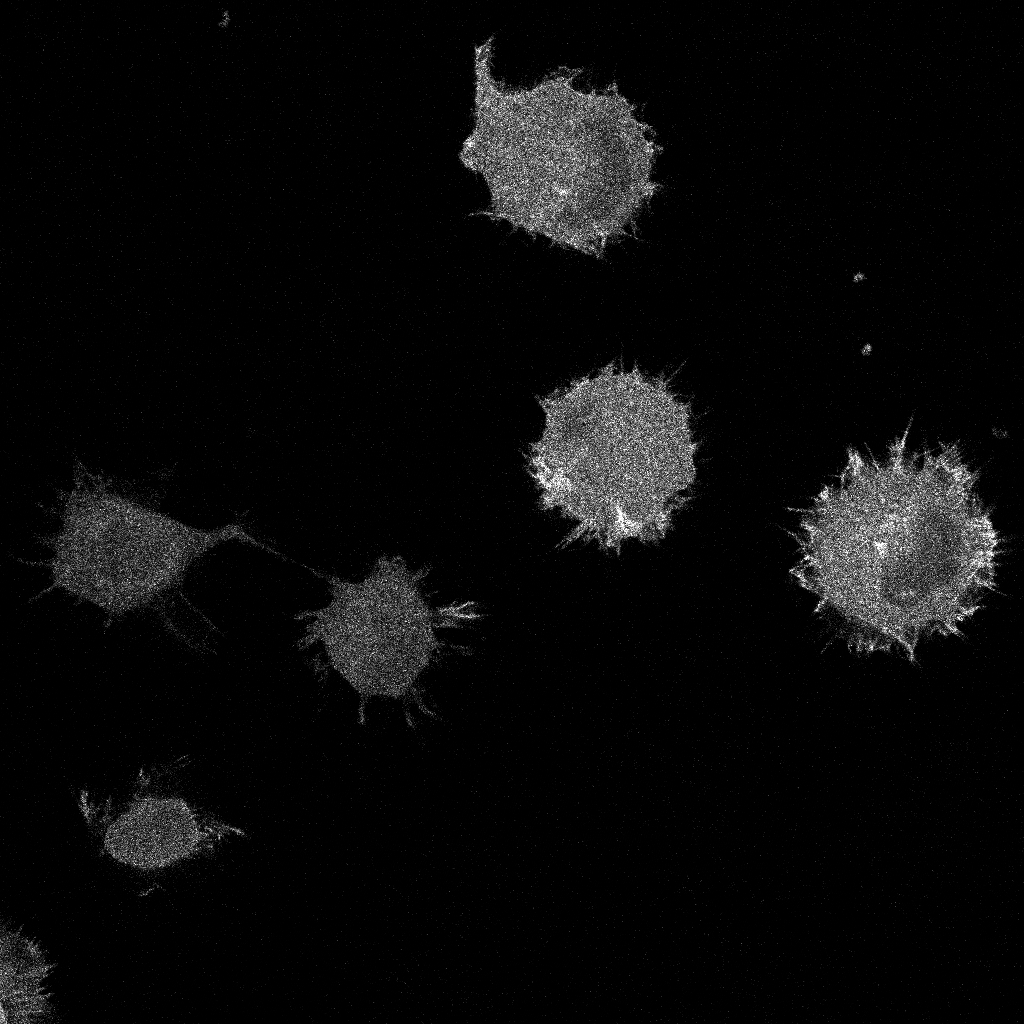

Supplement: Supplementary file 27 — Source Data for Figure 5 [file EMBJ-42-e113761-s005.zip › Figure 5/5C/DMSO/Upper stacks/(GFP-Eps8deltaCAP)_GFP-Eps8deltaCAP-IRSp53-mCherry_DMSO_stacks 8.tif]

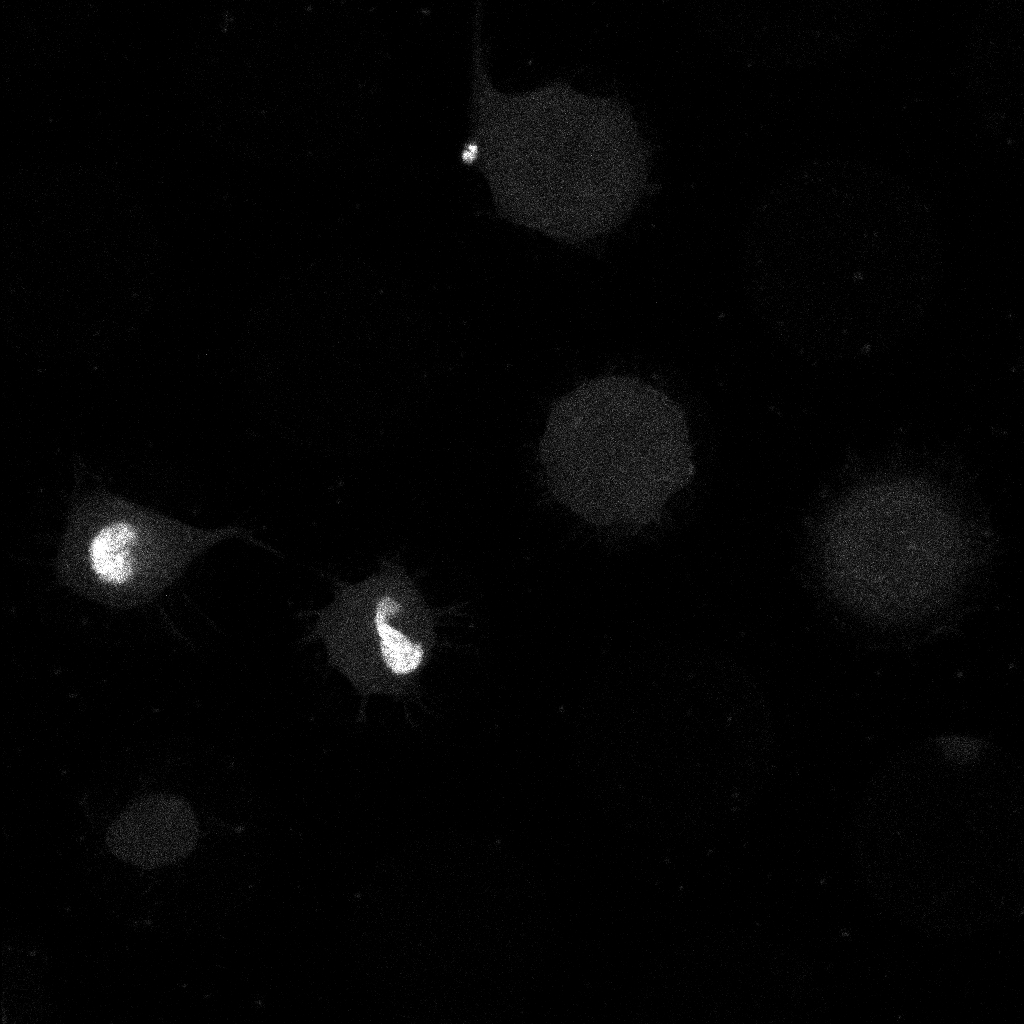

Supplement: Supplementary file 27 — Source Data for Figure 5 [file EMBJ-42-e113761-s005.zip › Figure 5/5C/DMSO/Upper stacks/(H2B-BFP & Fibronectin AF 405)_GFP-Eps8deltaCAP-IRSp53-mCherry_DMSO_stacks 8.tif]

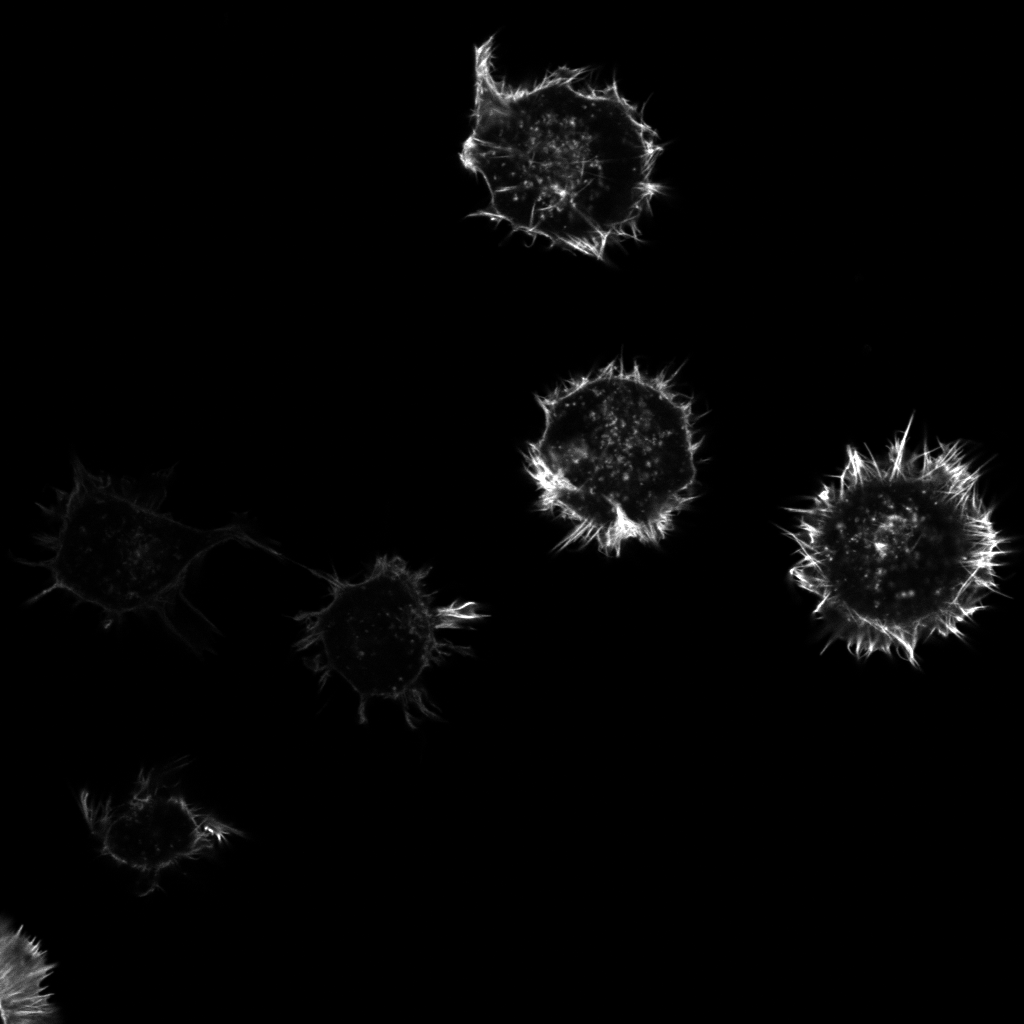

Supplement: Supplementary file 27 — Source Data for Figure 5 [file EMBJ-42-e113761-s005.zip › Figure 5/5C/DMSO/Upper stacks/(Rhodamine Phalloidin & IRSp53-mCherry)_GFP-Eps8deltaCAP-IRSp53-mCherry_DMSO_stacks 8.tif]

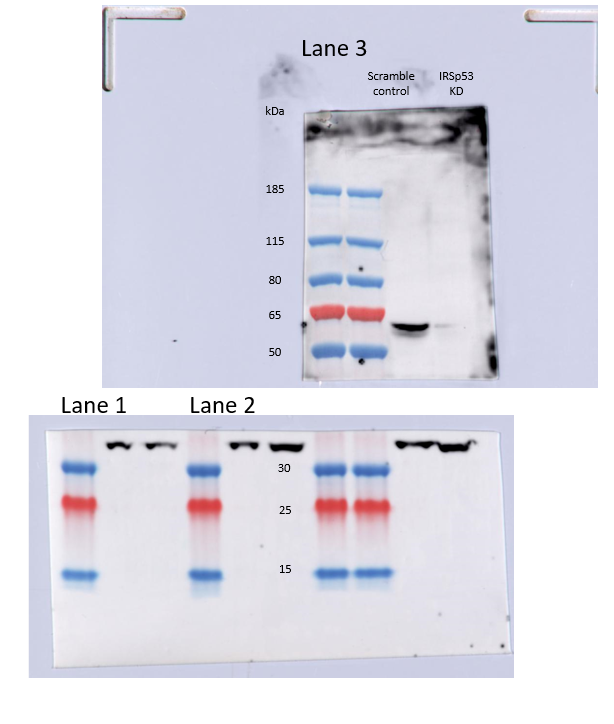

Supplement: Supplementary file 28 — Source Data for Figure 6 [file EMBJ-42-e113761-s024.zip › Figure 6/6A/WB/Annotated blots.PNG]

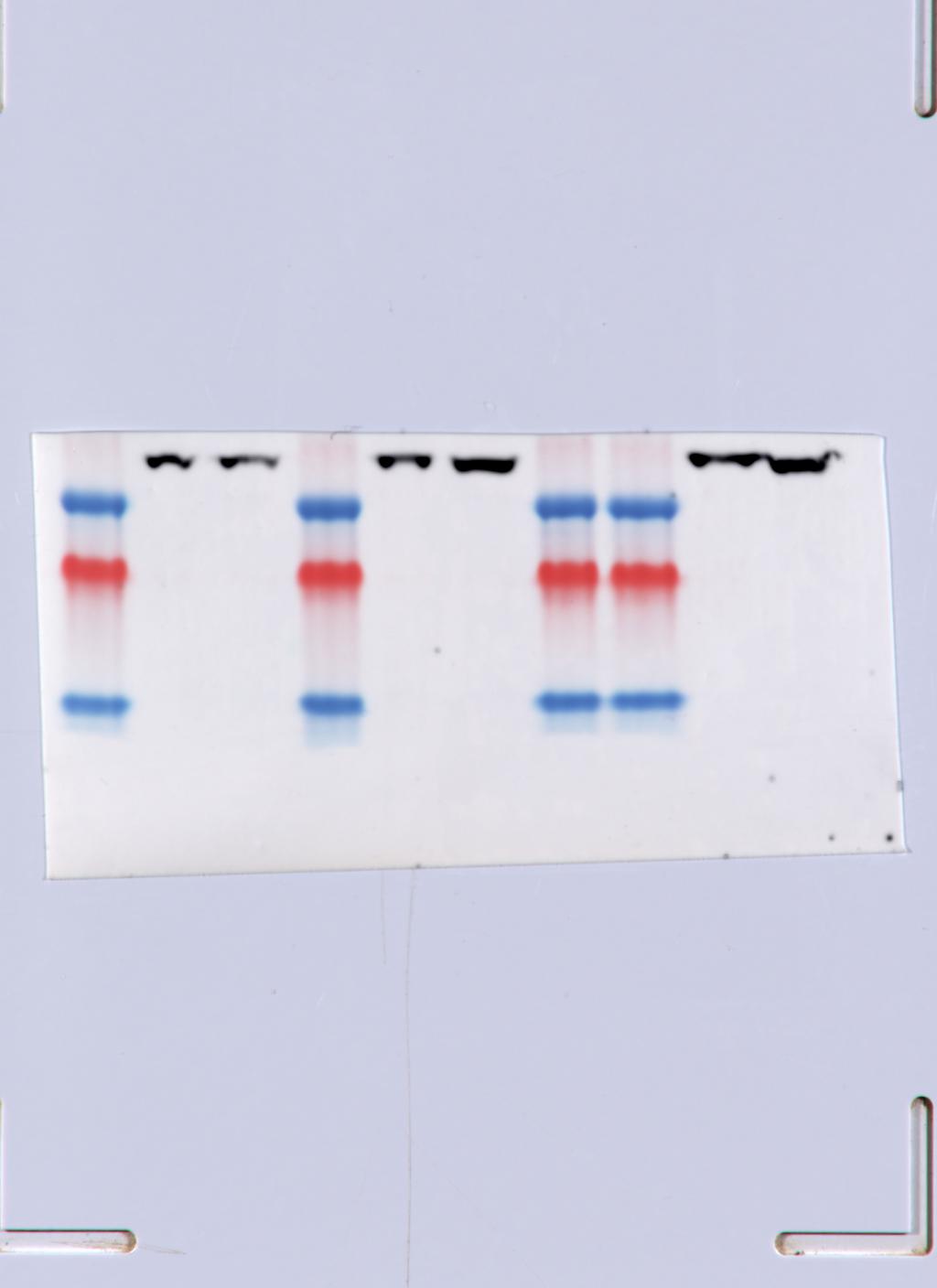

Supplement: Supplementary file 28 — Source Data for Figure 6 [file EMBJ-42-e113761-s024.zip › Figure 6/6A/WB/Ch+Ladder/GAPDH.jpg]

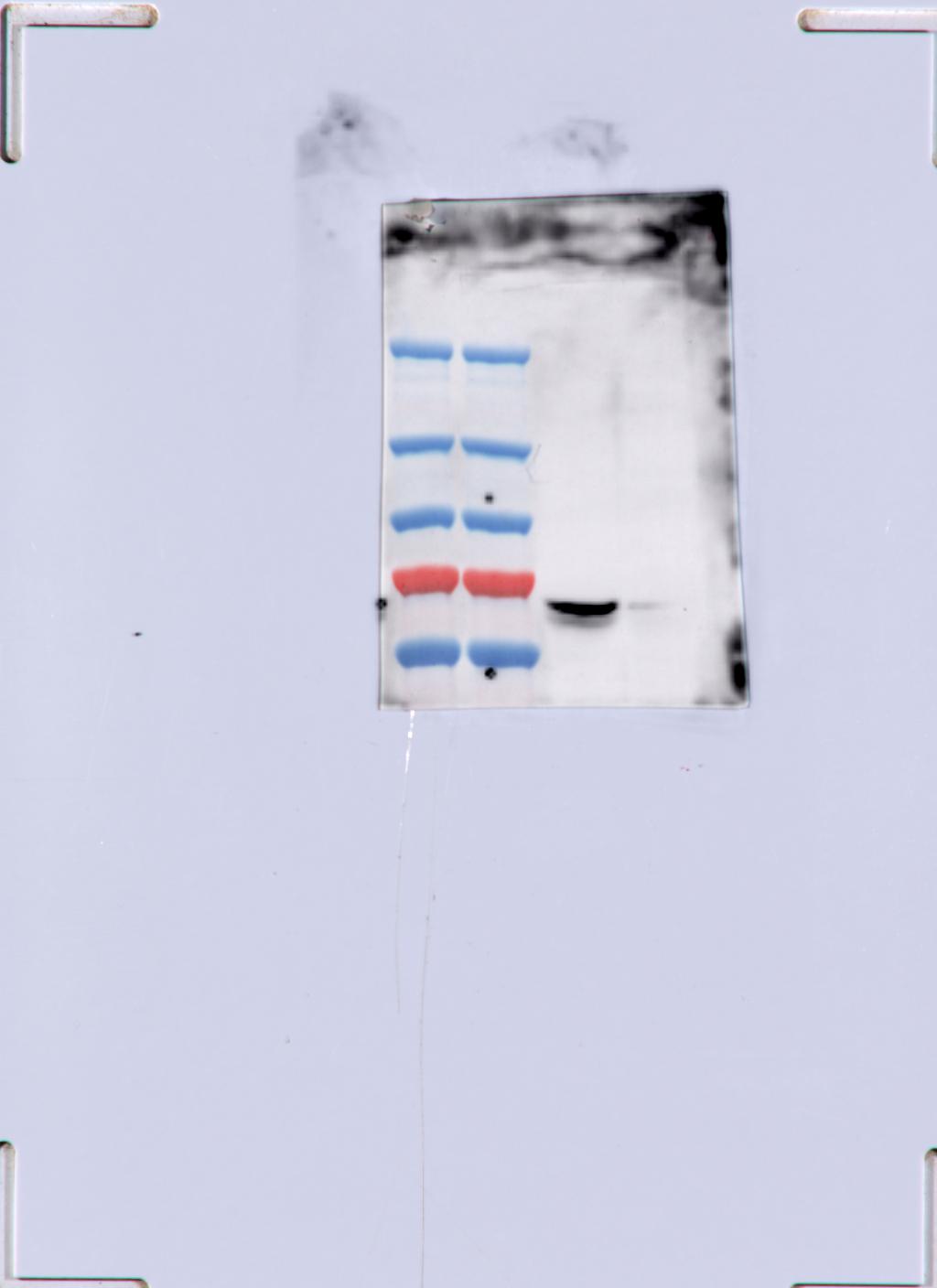

Supplement: Supplementary file 28 — Source Data for Figure 6 [file EMBJ-42-e113761-s024.zip › Figure 6/6A/WB/Ch+Ladder/IRSp53.jpg]

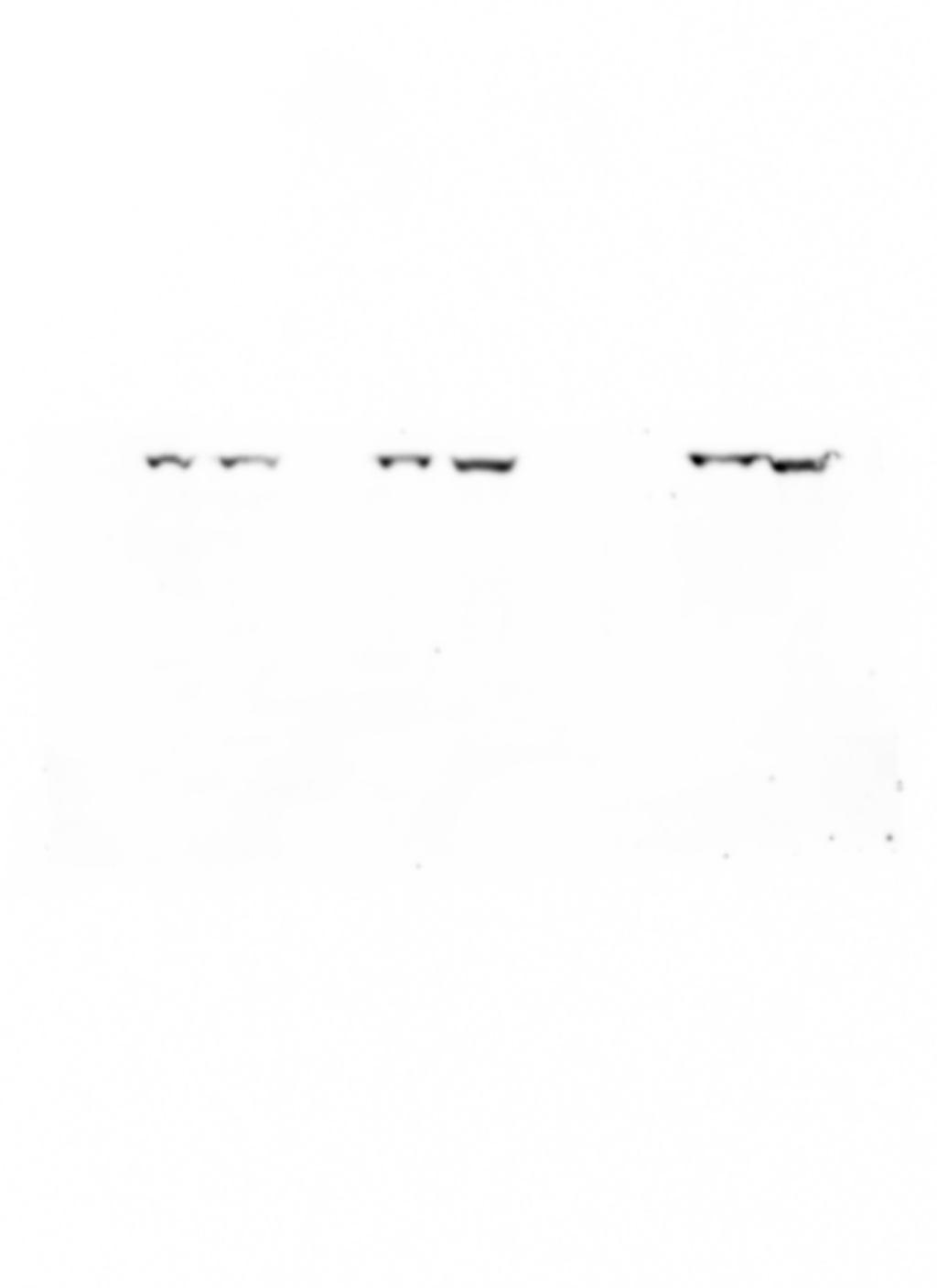

Supplement: Supplementary file 28 — Source Data for Figure 6 [file EMBJ-42-e113761-s024.zip › Figure 6/6A/WB/Raw TIFs/GAPDH.tif]

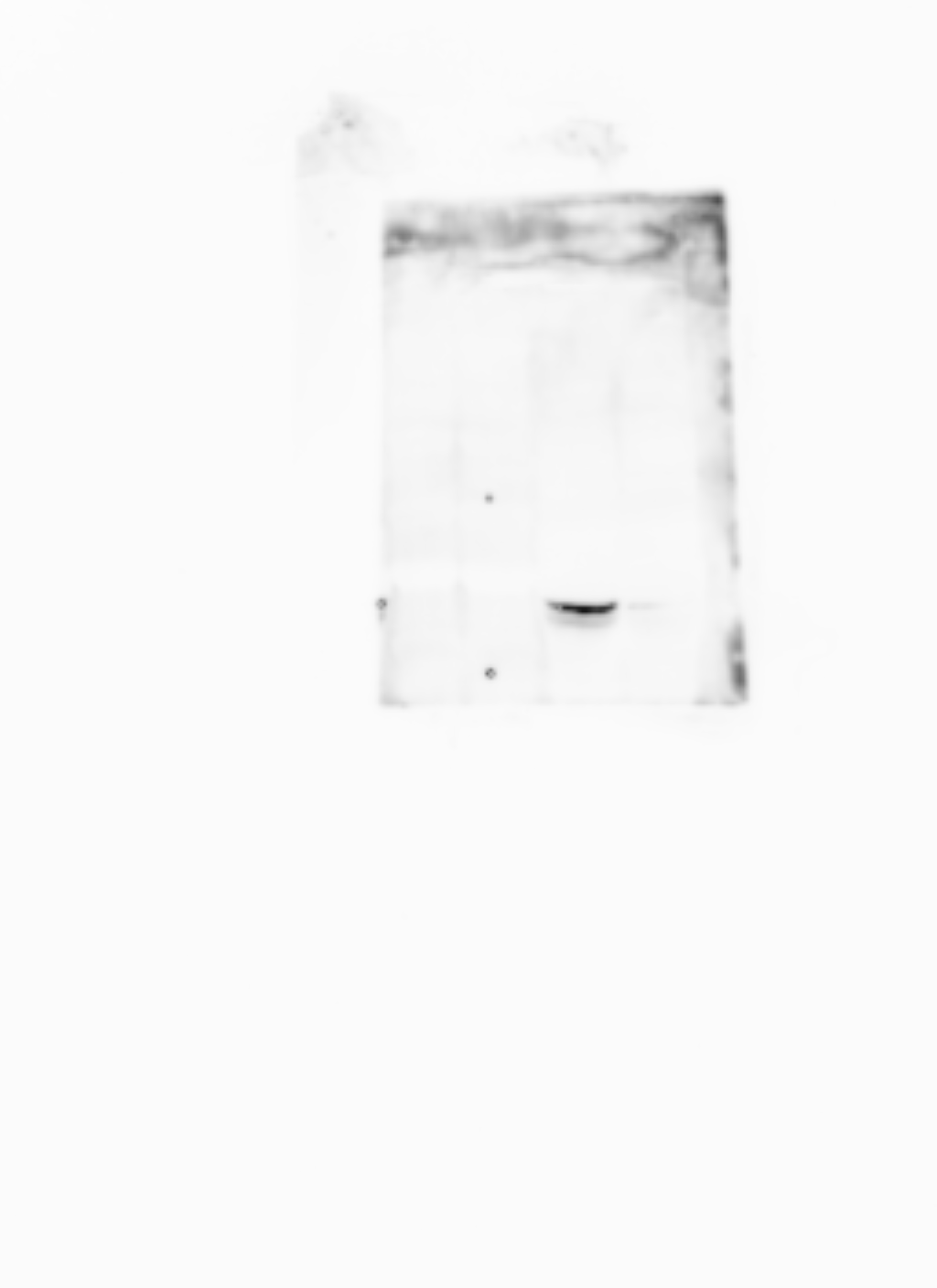

Supplement: Supplementary file 28 — Source Data for Figure 6 [file EMBJ-42-e113761-s024.zip › Figure 6/6A/WB/Raw TIFs/IRSp53.tif]

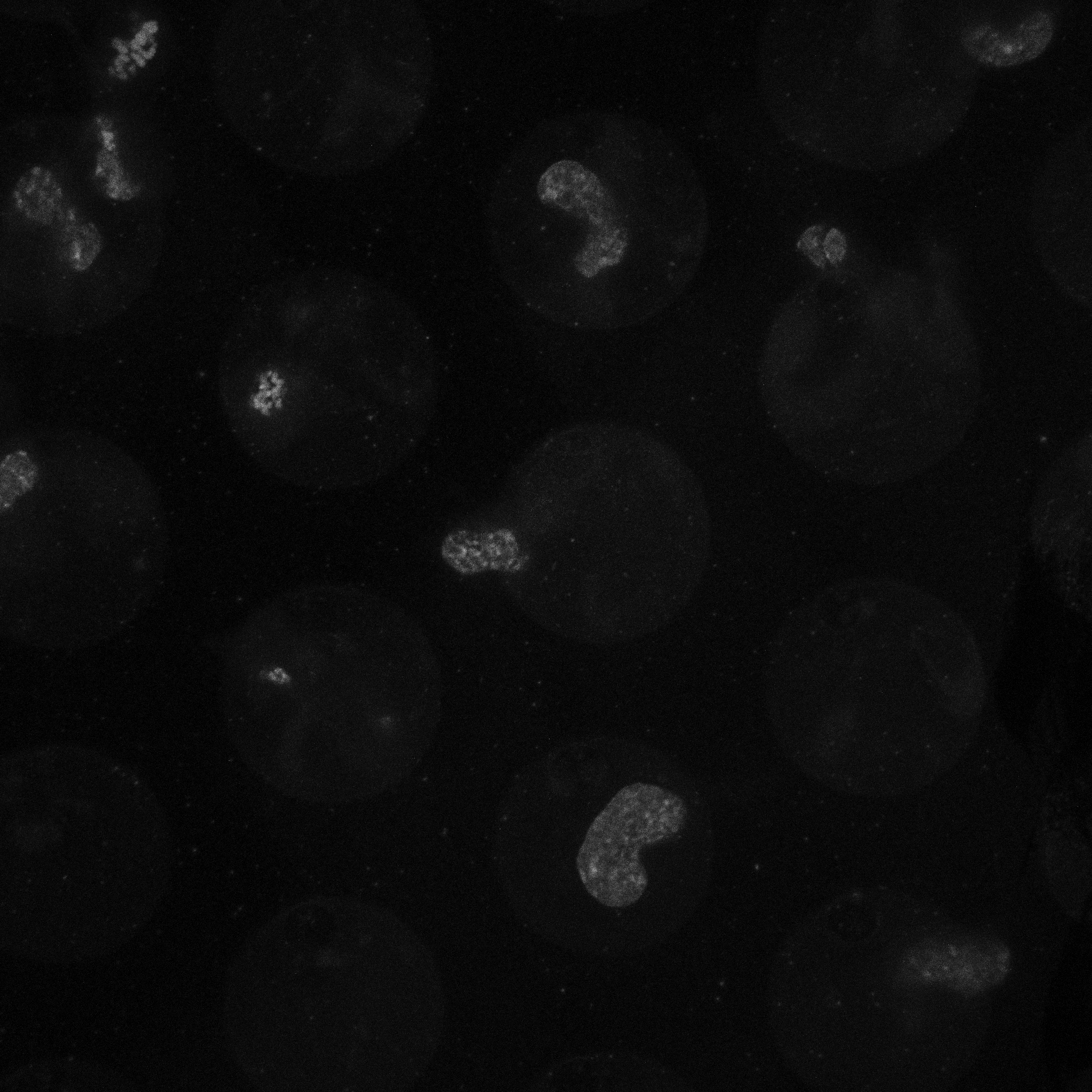

Supplement: Supplementary file 28 — Source Data for Figure 6 [file EMBJ-42-e113761-s024.zip › Figure 6/6B/Surface/405/blueMAXintensityprojectionLowerstacks_ScrambleControl-564phal-488wga-dapi+blue fibronectin-29-03-2023-03.tif]

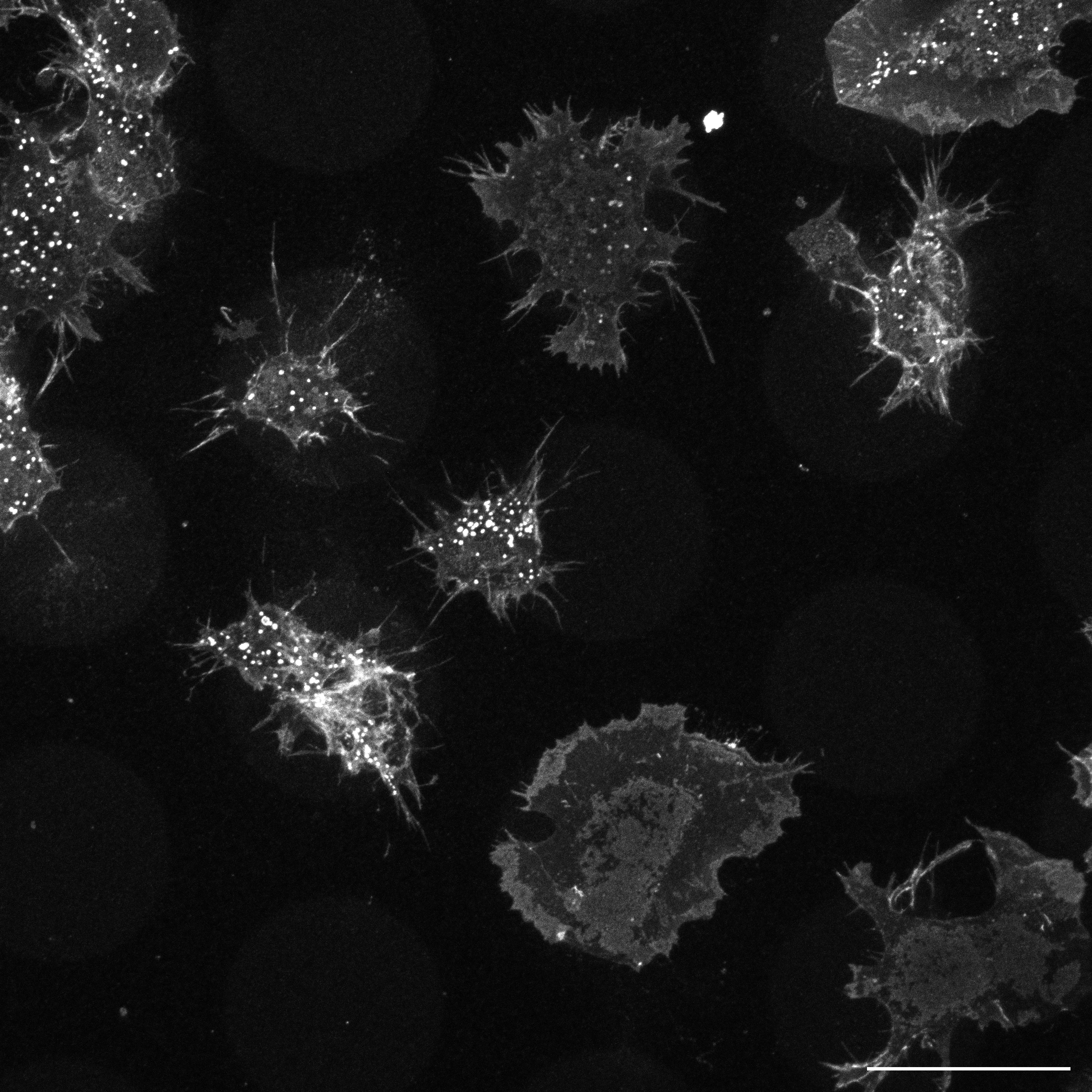

Supplement: Supplementary file 28 — Source Data for Figure 6 [file EMBJ-42-e113761-s024.zip › Figure 6/6B/Surface/488/greenMAXintensityprojectionLowerstacks_TNTcount-Scramblecontrol-564phalloidin-488wga-dapi-29-03-2023-03-1.png]

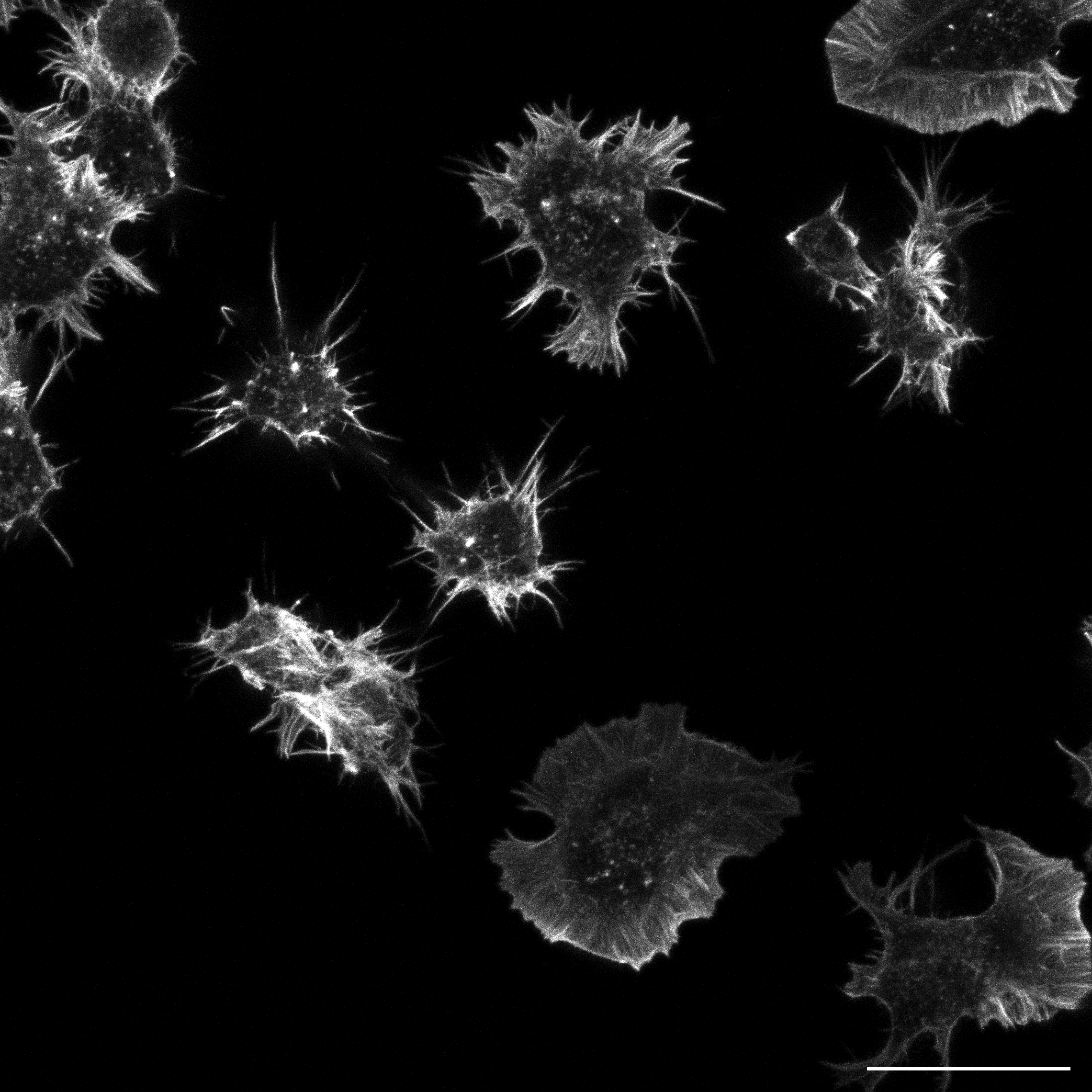

Supplement: Supplementary file 28 — Source Data for Figure 6 [file EMBJ-42-e113761-s024.zip › Figure 6/6B/Surface/564/redMAXintensityprojectionLowerstacks_TNTcount-Scramblecontrol-564phalloidin-488wga-dapi-29-03-2023-03-1.png (red).tif]

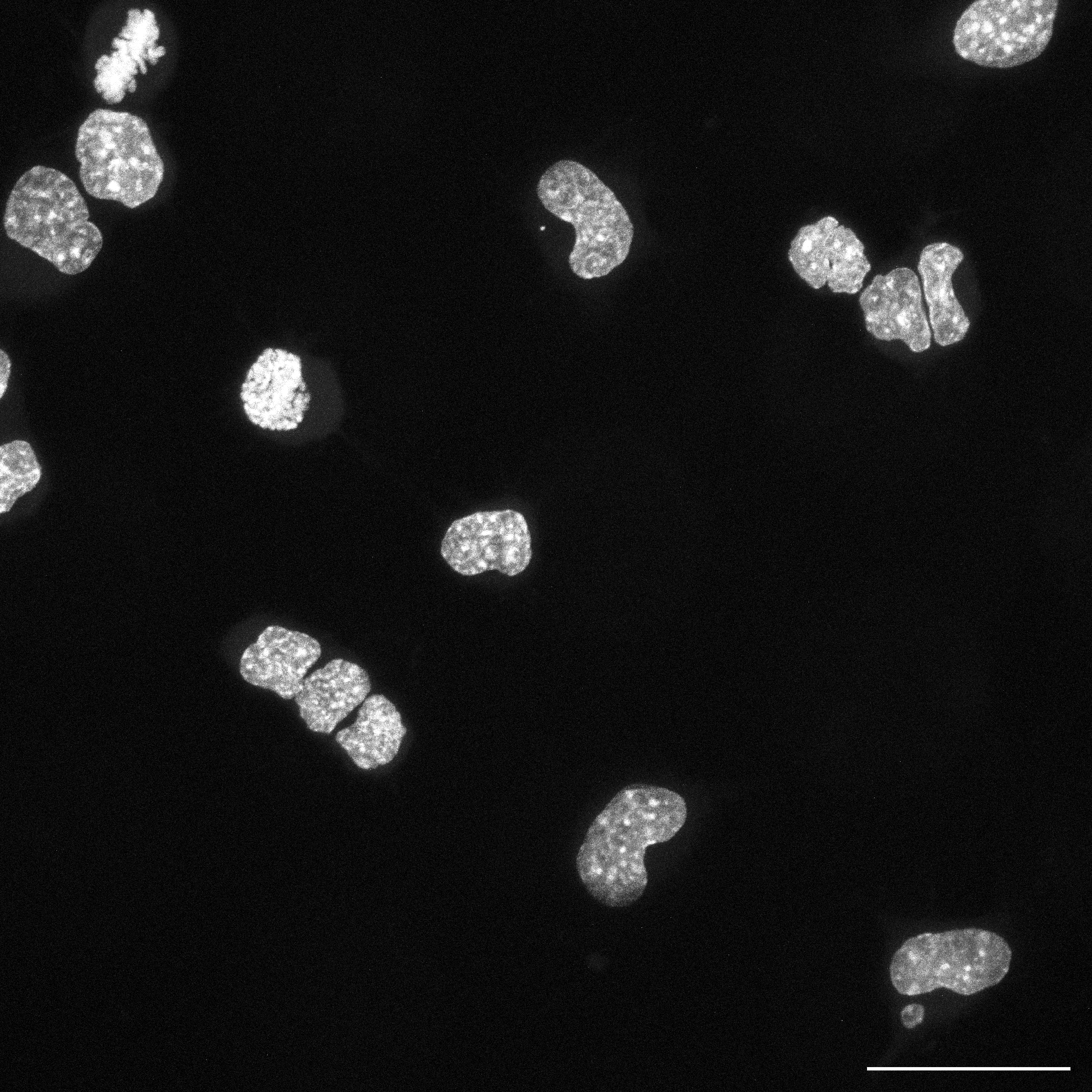

Supplement: Supplementary file 28 — Source Data for Figure 6 [file EMBJ-42-e113761-s024.zip › Figure 6/6B/Upper stacks/405/blueMaxintensityprojection-Upperstacks-ScrambleControl-redphal-488wga-dapi-29-03-2023.png (blue).tif]

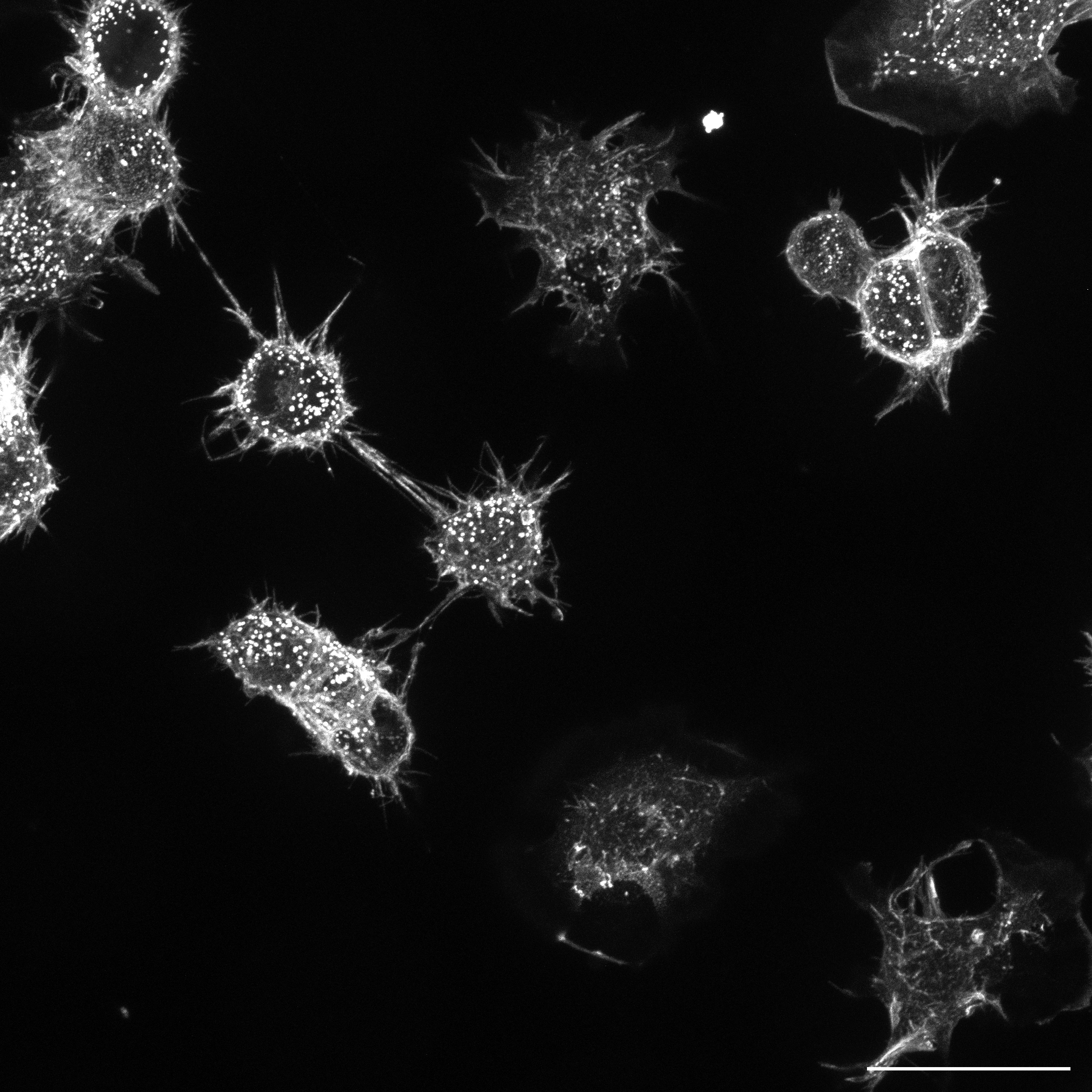

Supplement: Supplementary file 28 — Source Data for Figure 6 [file EMBJ-42-e113761-s024.zip › Figure 6/6B/Upper stacks/488/greenMaxintensityprojection-Upperstacks-ScrambleControl-redphal-488wga-dapi-29-03-2023.png (green).tif]

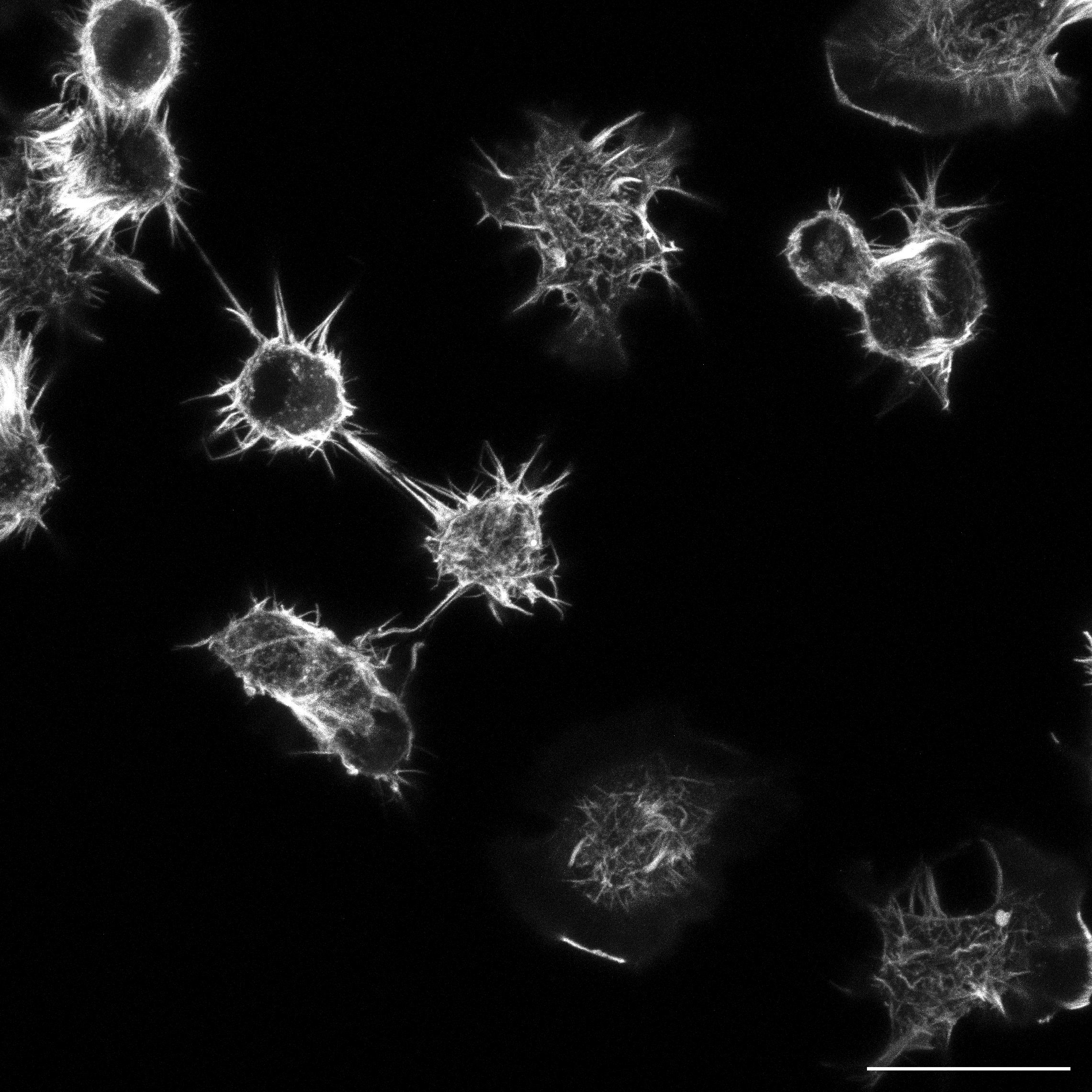

Supplement: Supplementary file 28 — Source Data for Figure 6 [file EMBJ-42-e113761-s024.zip › Figure 6/6B/Upper stacks/564/redMaxintensityprojection-Upperstacks-ScrambleControl-redphal-488wga-dapi-29-03-2023.png (red).tif]

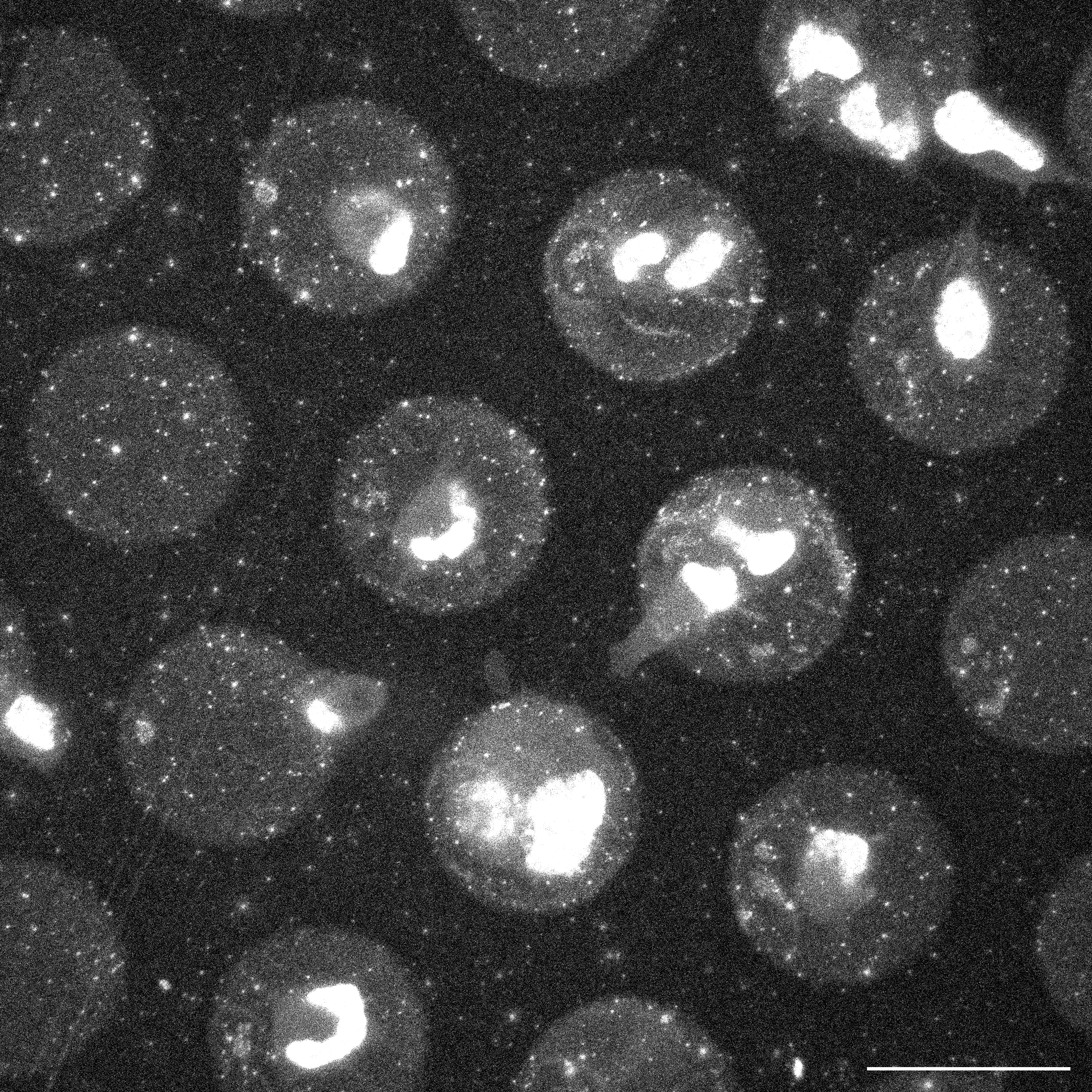

Supplement: Supplementary file 28 — Source Data for Figure 6 [file EMBJ-42-e113761-s024.zip › Figure 6/6C/Surface/405/Maxintensityprojections-Lowerstacks_IRSp53KD-564phalloidin-488wga-dapi+bluefibronectin-29-03-2023-02.png (blue).tif]

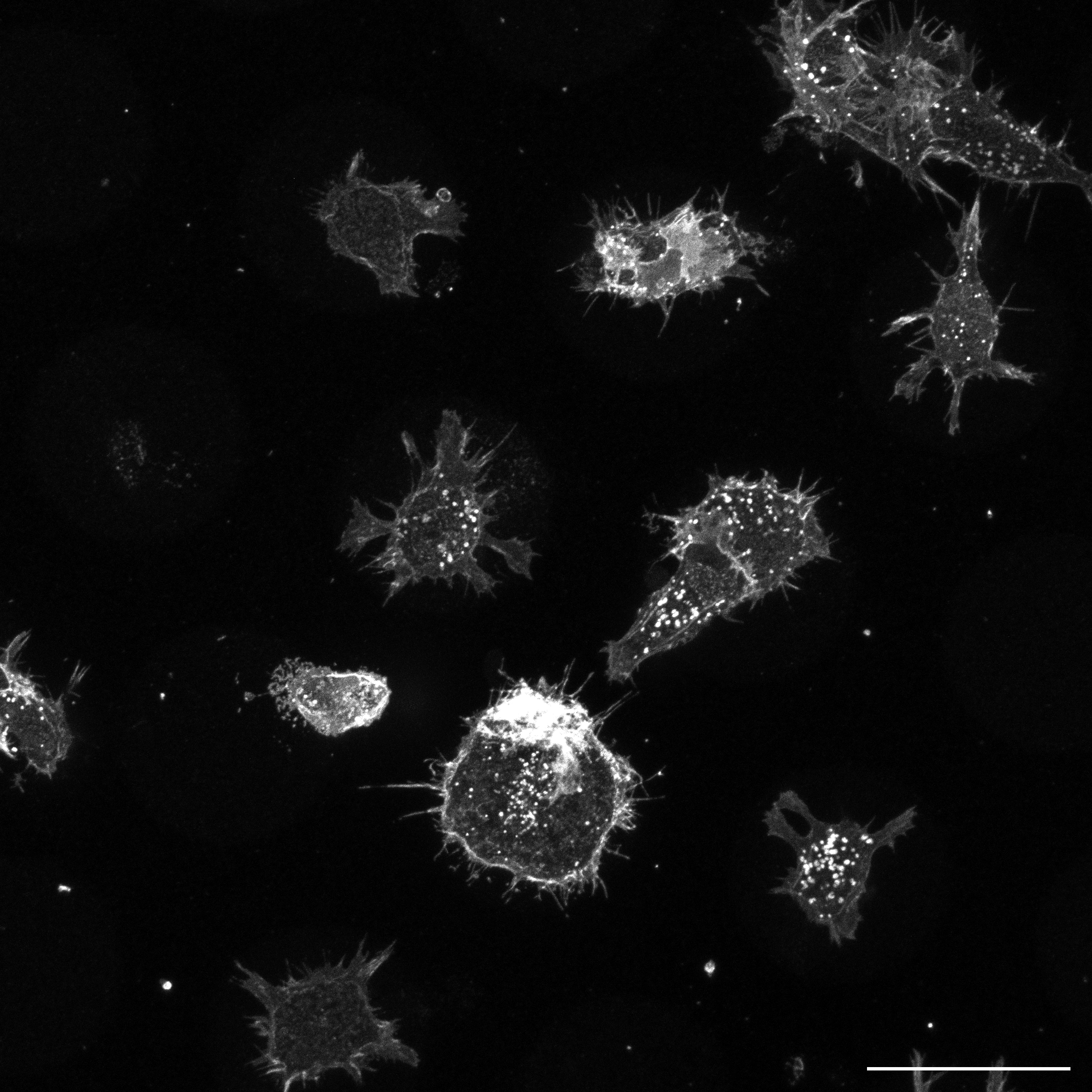

Supplement: Supplementary file 28 — Source Data for Figure 6 [file EMBJ-42-e113761-s024.zip › Figure 6/6C/Surface/488/Maxintensityprojections-Lowerstacks_IRSp53KD-564phalloidin-488wga-dapi+bluefibronectin-29-03-2023-02.png (green).tif]

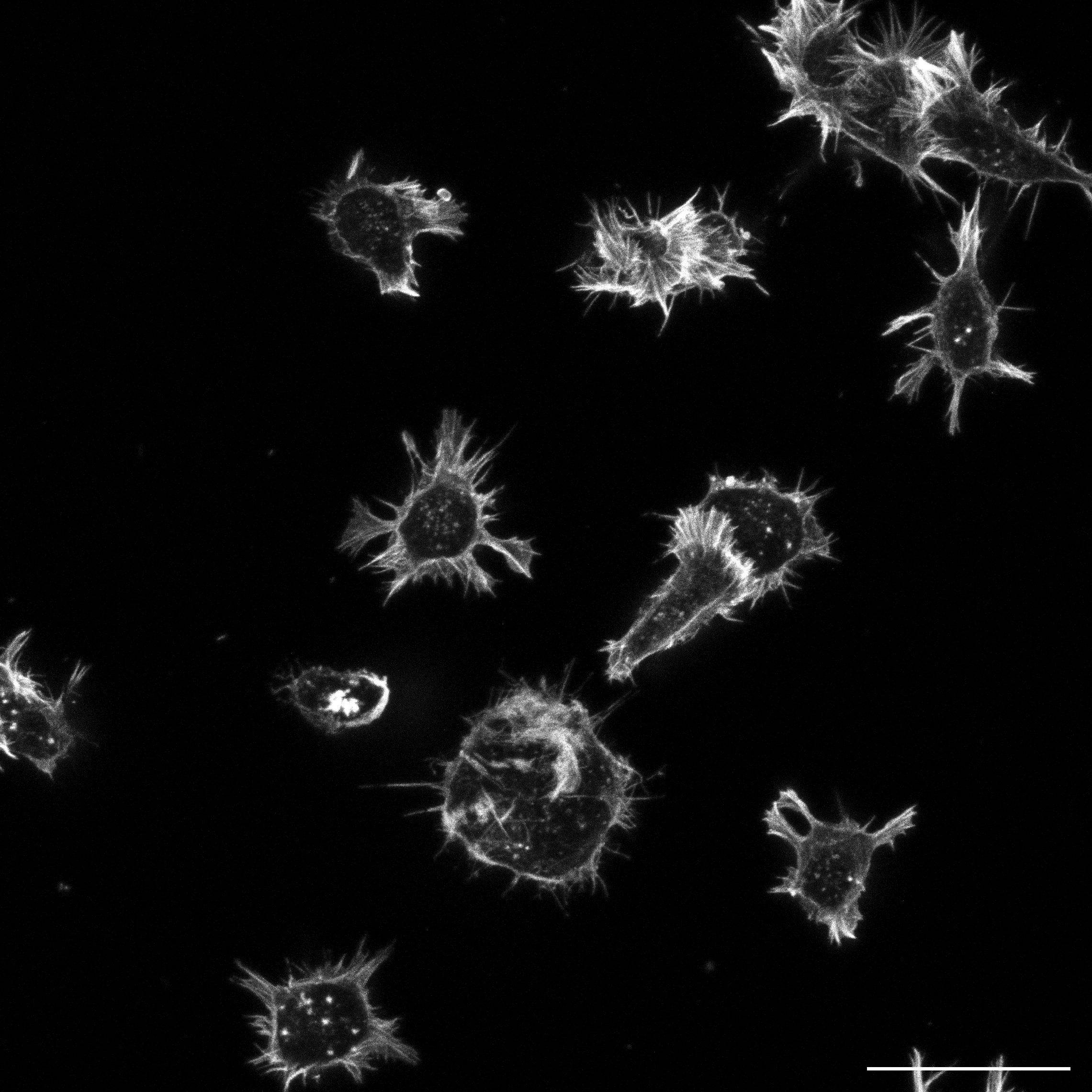

Supplement: Supplementary file 28 — Source Data for Figure 6 [file EMBJ-42-e113761-s024.zip › Figure 6/6C/Surface/564/Maxintensityprojections-Lowerstacks_IRSp53KD-564phalloidin-488wga-dapi+bluefibronectin-29-03-2023-02.png (red).tif]

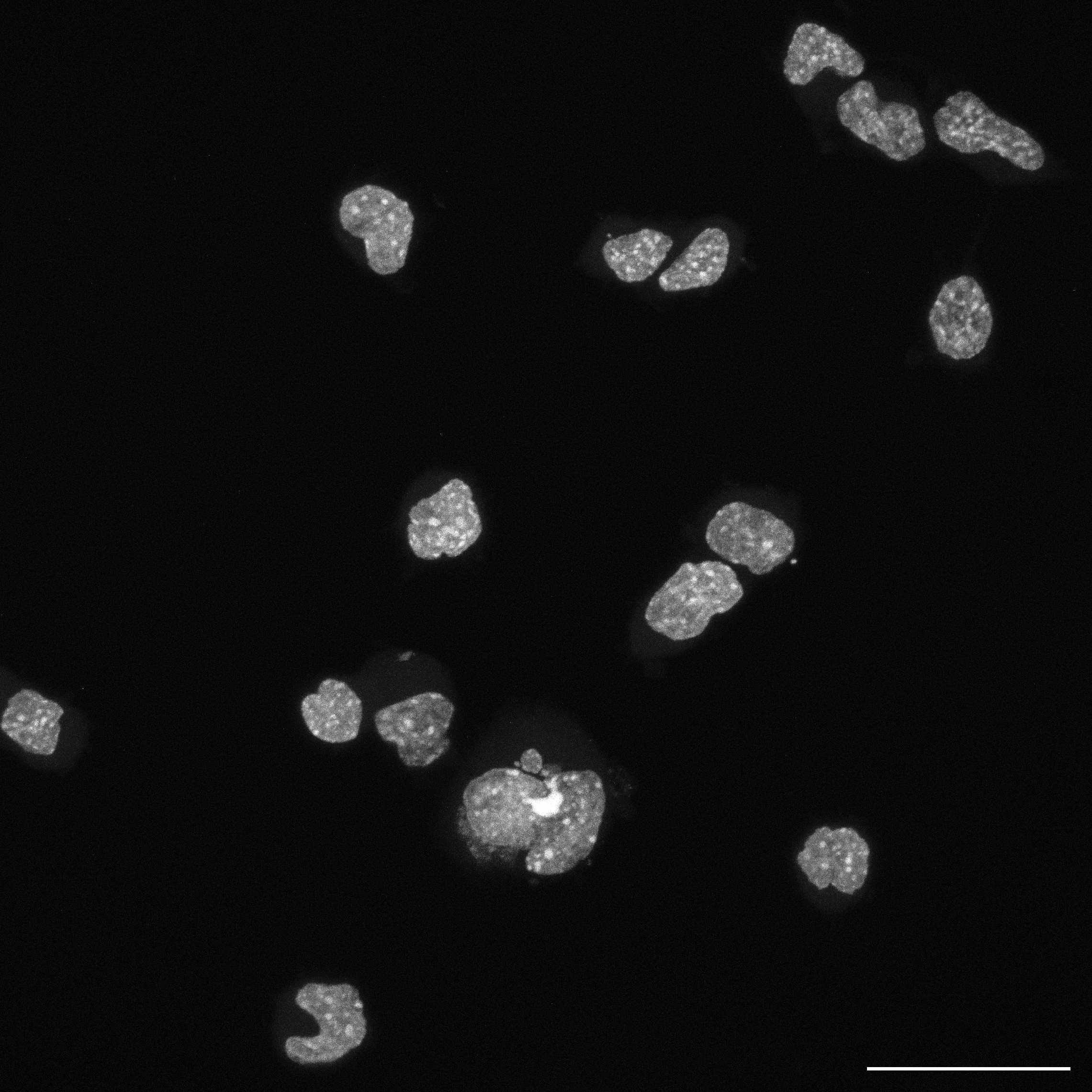

Supplement: Supplementary file 28 — Source Data for Figure 6 [file EMBJ-42-e113761-s024.zip › Figure 6/6C/Upper stacks/405/Maxintensityprojections-upperstacks_IRSp53KD-564phalloidin-488wga-dapi+bluefibronectin-29-03-2023-02.png (blue).tif]

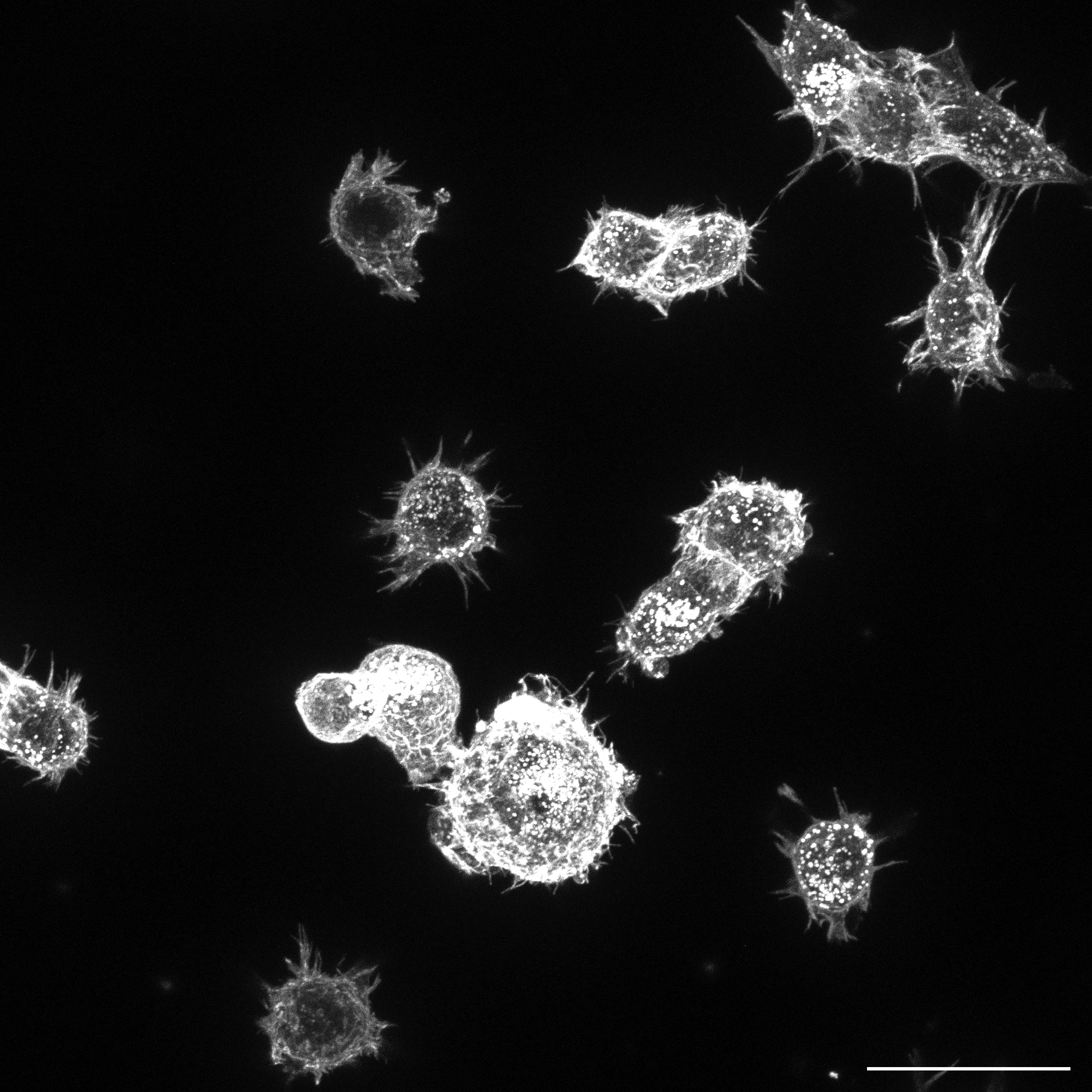

Supplement: Supplementary file 28 — Source Data for Figure 6 [file EMBJ-42-e113761-s024.zip › Figure 6/6C/Upper stacks/488/Maxintensityprojections-upperstacks_IRSp53KD-564phalloidin-488wga-dapi+bluefibronectin-29-03-2023-02.png (green).tif]

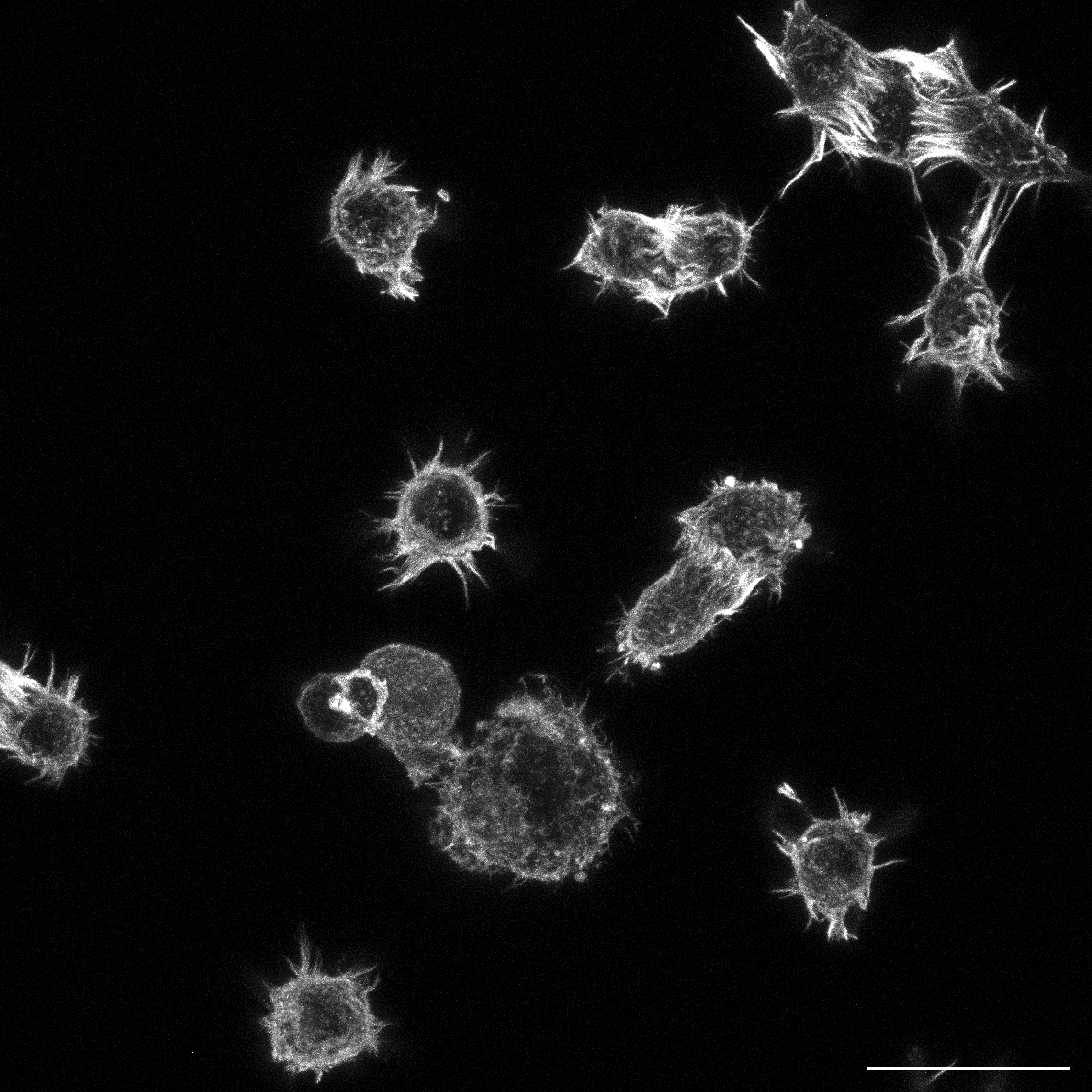

Supplement: Supplementary file 28 — Source Data for Figure 6 [file EMBJ-42-e113761-s024.zip › Figure 6/6C/Upper stacks/564/Maxintensityprojections-upperstacks_IRSp53KD-564phalloidin-488wga-dapi+bluefibronectin-29-03-2023-02.png (red).tif]

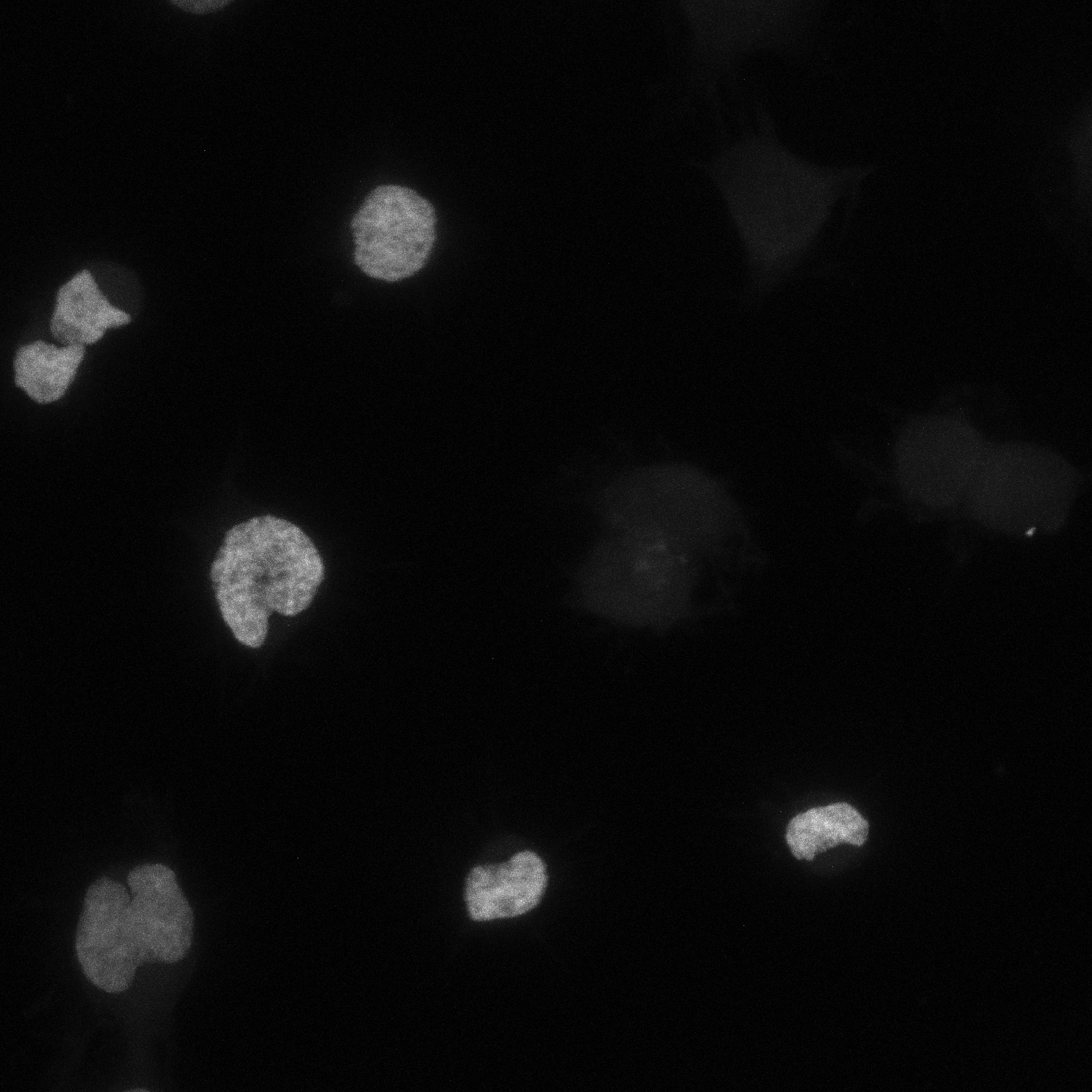

Supplement: Supplementary file 28 — Source Data for Figure 6 [file EMBJ-42-e113761-s024.zip › Figure 6/6E/Full field of view/405/C3-MAX_MAXprojectionscramblecontrol_488WGA-DiD-H2bBFPcoC-17032023.tif]

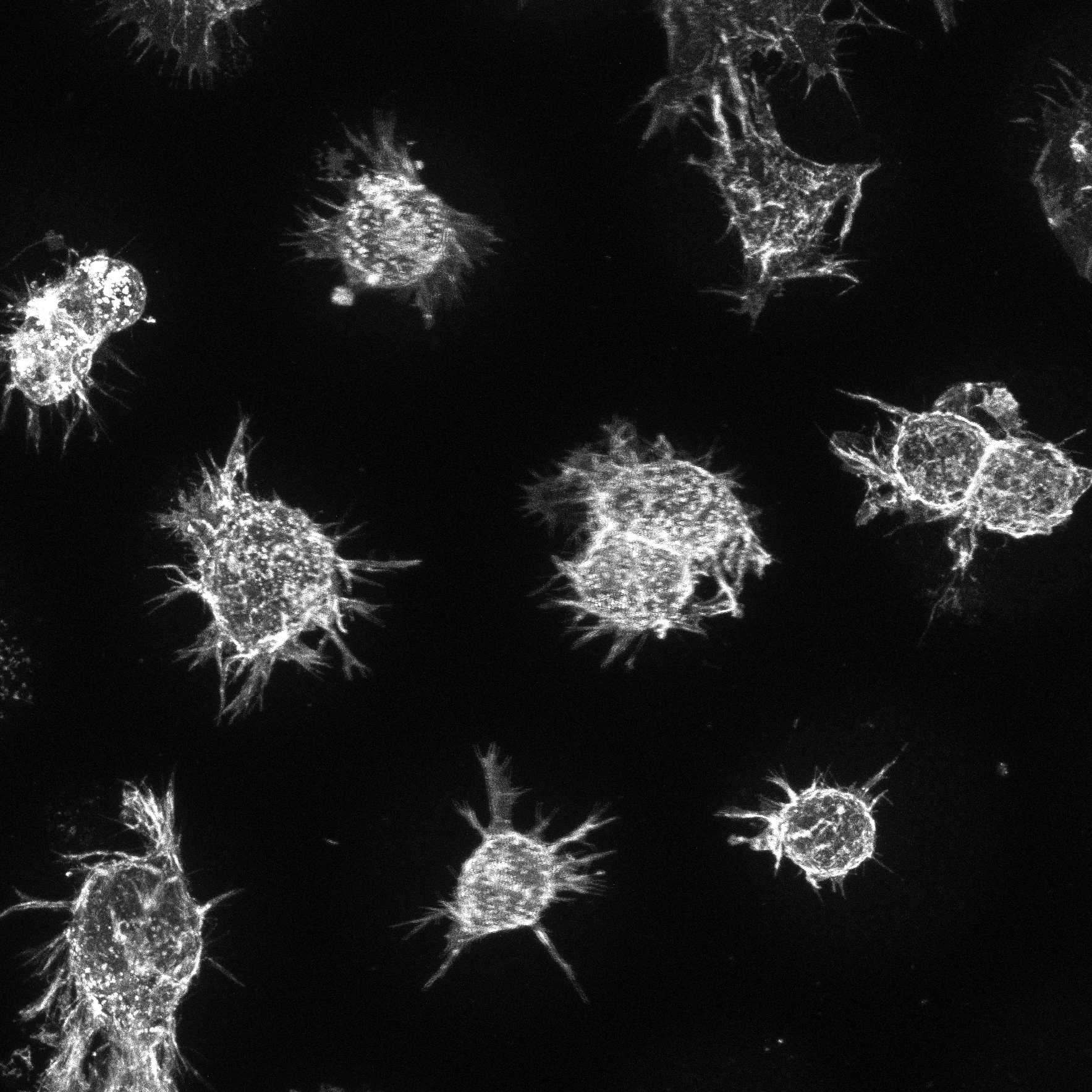

Supplement: Supplementary file 28 — Source Data for Figure 6 [file EMBJ-42-e113761-s024.zip › Figure 6/6E/Full field of view/488/C2-MAX_MAXprojectionscramblecontrol_488WGA-DiD-H2bBFPcoC-17032023.tif]

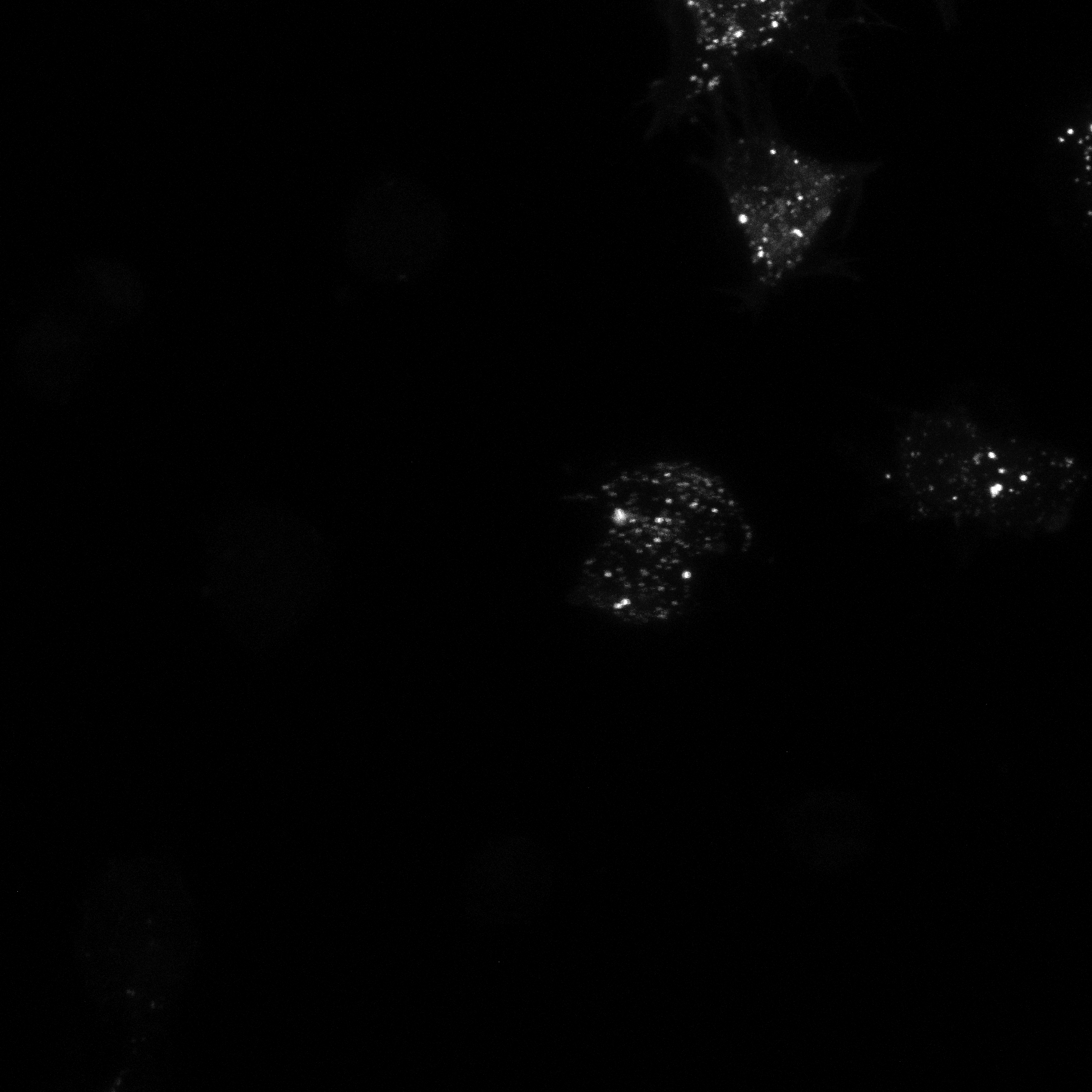

Supplement: Supplementary file 28 — Source Data for Figure 6 [file EMBJ-42-e113761-s024.zip › Figure 6/6E/Full field of view/647/C1-MAX_MAXprojectionscramblecontrol_488WGA-DiD-H2bBFPcoC-17032023.tif]

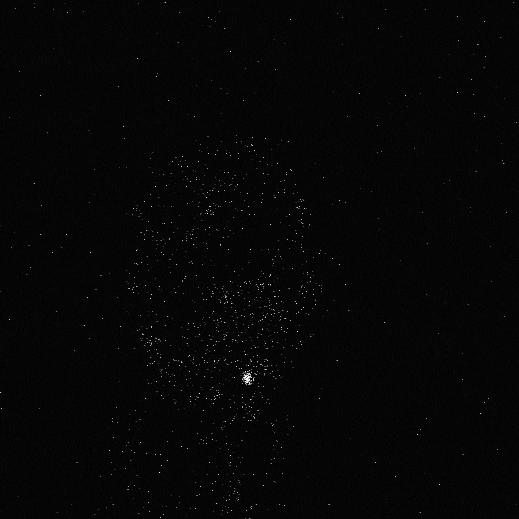

Supplement: Supplementary file 28 — Source Data for Figure 6 [file EMBJ-42-e113761-s024.zip › Figure 6/6E/i/XY.tif]

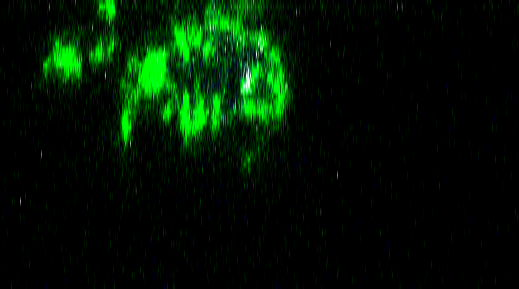

Supplement: Supplementary file 28 — Source Data for Figure 6 [file EMBJ-42-e113761-s024.zip › Figure 6/6E/i/XZ.tif]

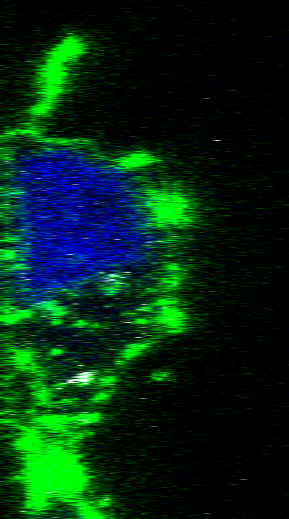

Supplement: Supplementary file 28 — Source Data for Figure 6 [file EMBJ-42-e113761-s024.zip › Figure 6/6E/i/YZ.tif]

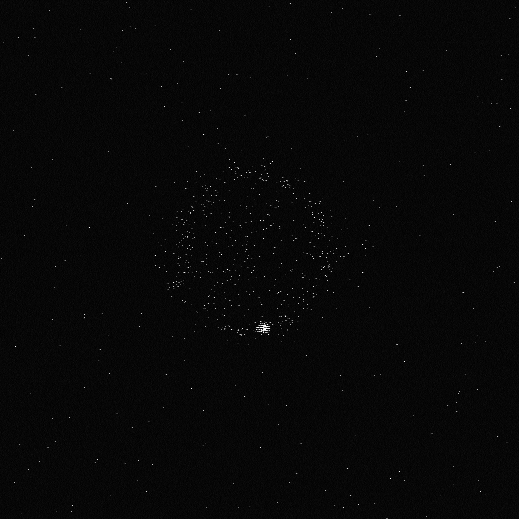

Supplement: Supplementary file 28 — Source Data for Figure 6 [file EMBJ-42-e113761-s024.zip › Figure 6/6E/ii/XY.tif]

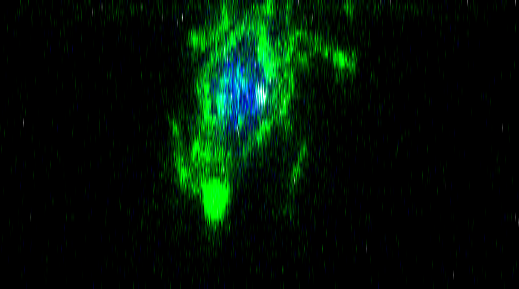

Supplement: Supplementary file 28 — Source Data for Figure 6 [file EMBJ-42-e113761-s024.zip › Figure 6/6E/ii/XZ.tif]

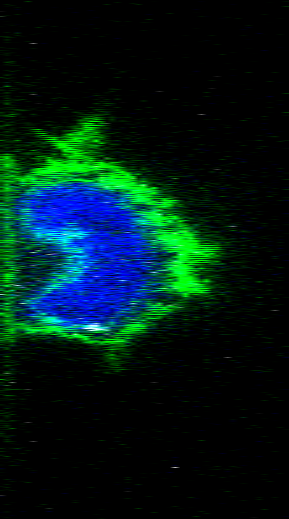

Supplement: Supplementary file 28 — Source Data for Figure 6 [file EMBJ-42-e113761-s024.zip › Figure 6/6E/ii/YZ.tif]

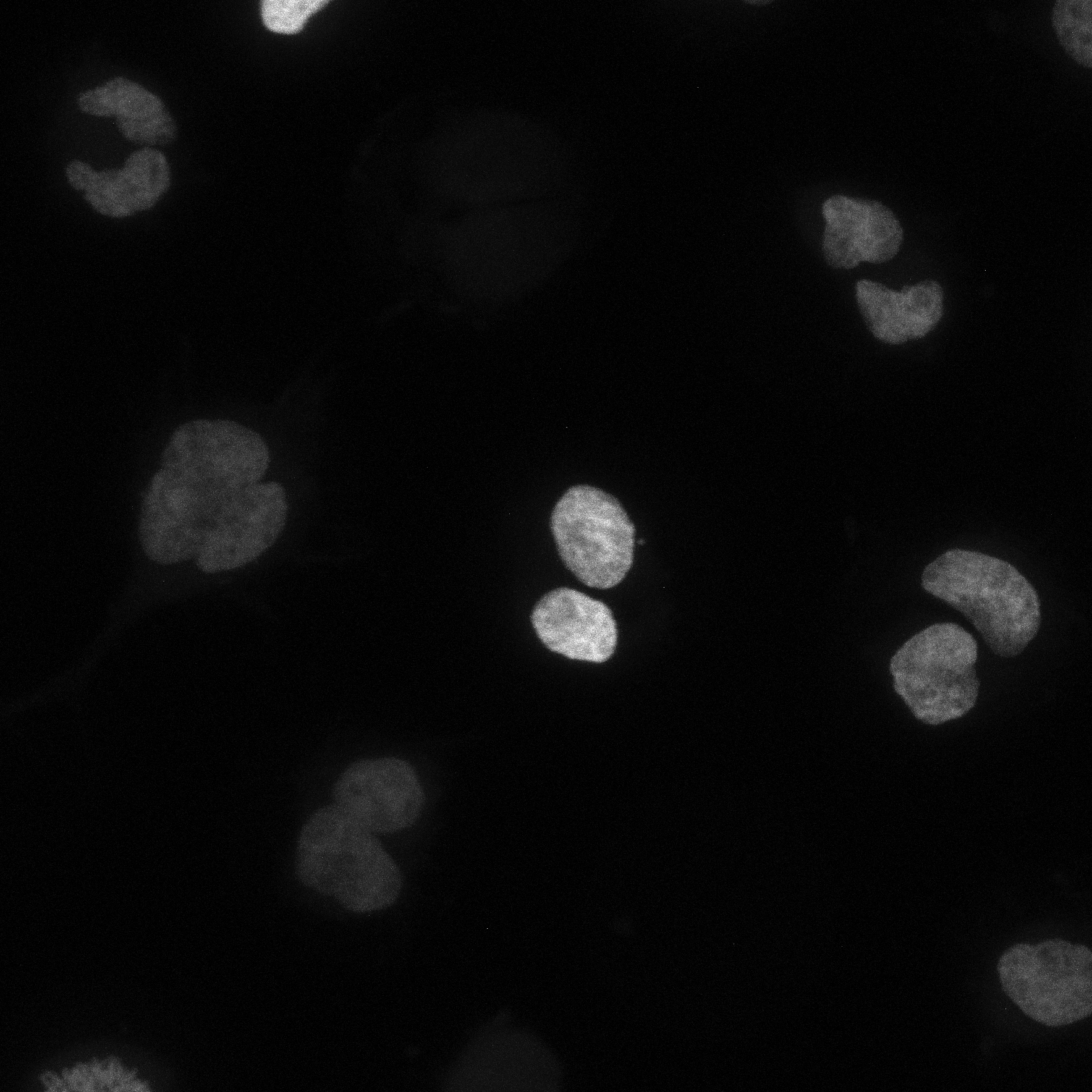

Supplement: Supplementary file 28 — Source Data for Figure 6 [file EMBJ-42-e113761-s024.zip › Figure 6/6F/Full field of view/405/C3-MAX_488WGA-DiD-H2bBFPcoC-IRSp53KD-14032023.tif]

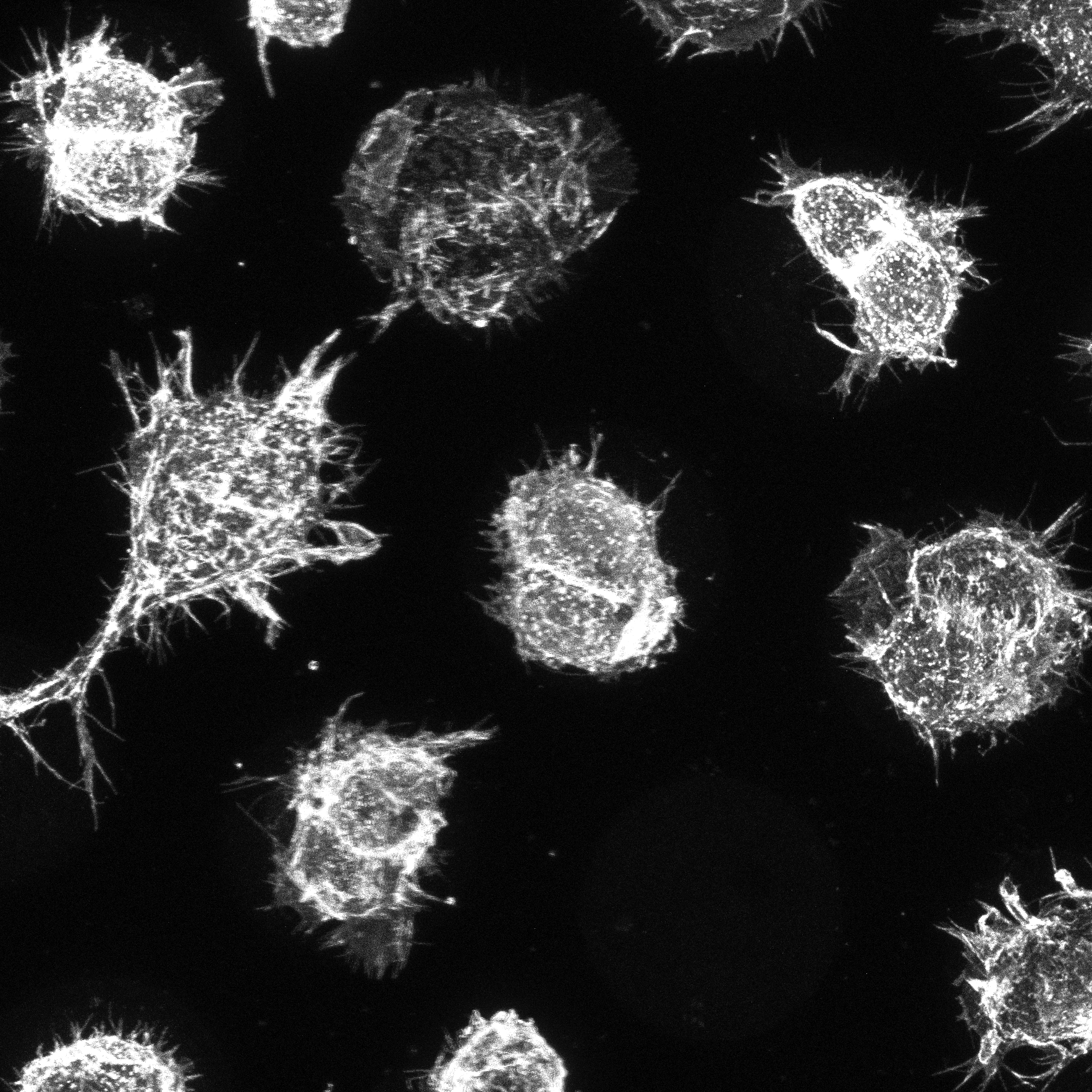

Supplement: Supplementary file 28 — Source Data for Figure 6 [file EMBJ-42-e113761-s024.zip › Figure 6/6F/Full field of view/488/C2-MAX_488WGA-DiD-H2bBFPcoC-IRSp53KD-14032023.tif]

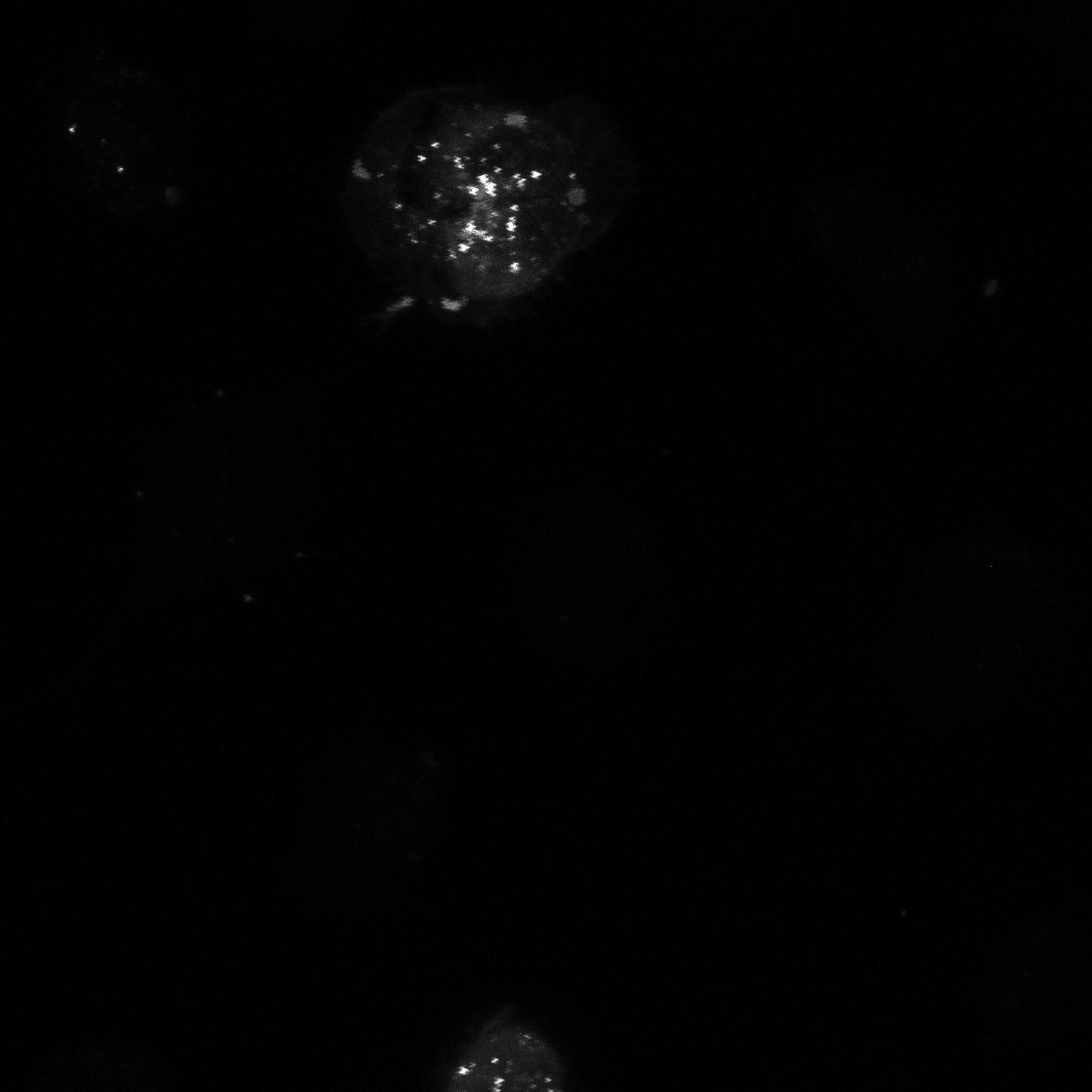

Supplement: Supplementary file 28 — Source Data for Figure 6 [file EMBJ-42-e113761-s024.zip › Figure 6/6F/Full field of view/647/C1-MAX_488WGA-DiD-H2bBFPcoC-IRSp53KD-14032023.tif]

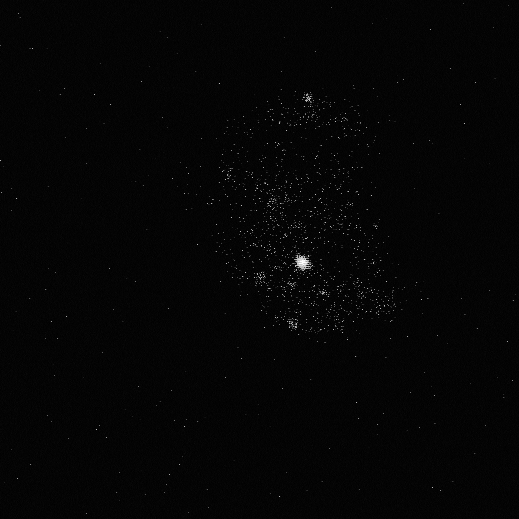

Supplement: Supplementary file 28 — Source Data for Figure 6 [file EMBJ-42-e113761-s024.zip › Figure 6/6F/i/XY.tif]

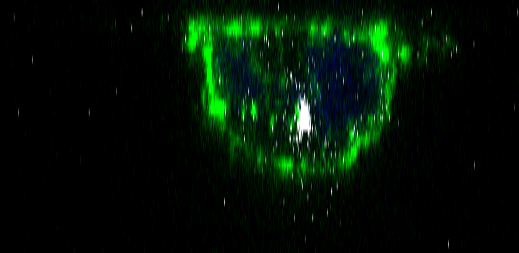

Supplement: Supplementary file 28 — Source Data for Figure 6 [file EMBJ-42-e113761-s024.zip › Figure 6/6F/i/XZ.tif]

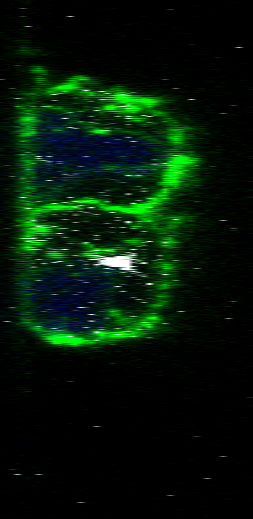

Supplement: Supplementary file 28 — Source Data for Figure 6 [file EMBJ-42-e113761-s024.zip › Figure 6/6F/i/YZ.tif]
