# Supplementary material for: Tunnelling nanotube formation is driven by Eps8/IRSp53‐dependent linear actin polymerization
Source: EMBO J. 2023 Nov 27;42(24):e113761. doi: 10.15252/embj.2023113761 (PMC10711657; doi:10.15252/embj.2023113761)
Supplement: Supplementary file 29 [file EMBJ-42-e113761-s003.pdf]

THE EMBO JOURNAL

Tunneling nanotube formation is driven by Eps8/IRSp53-dependent linear actin polymerization

J. Henderson, Nina Ljubojevic, Sevan Belian, Thibault Chaze, Daryl Castaneda, Aude Battistella, Quentin Giai Gianetto, Mariette Matondo, Stéphanie Descroix, Patricia Bassereau, and Chiara Zurzolo
DOI: 10.15252/embj.2023113761

Corresponding authors: Chiara Zurzolo (chiara.zurzolo@pasteur.fr) , Patricia Bassereau (patricia.bassereau@curie.fr), J. Henderson (m.henderson@bowdoin.edu)

Review Timeline:

Transfer from Review Commons:	13th Feb 23
Editorial Decision:	9th Mar 23
Revision Received:	31st Jul 23
Editorial Decision:	31st Aug 23
Revision Received:	27th Oct 23
Accepted:	2nd Nov 23

Editor: Ieva Gailite

Transaction Report:

Review COMMONS

This manuscript was transferred to The EMBO Journal following peer review at Review Commons.

(Note: With the exception of the correction of typographical or spelling errors that could be a source of ambiguity, letters and reports are not edited. Depending on transfer agreements, referee reports obtained elsewhere may or may not be included in this compilation. Referee reports are anonymous unless the Referee chooses to sign their reports.)

Review #1

1. Evidence, reproducibility and clarity:

Evidence, reproducibility and clarity (Required)

I applaud the authors for the creative experimentation and intriguing findings presented in this work, but the narrative lacks a clear biological question and, as written, comes across more like a collection of thematically related results, rather than a logical point A > B > C progression of studies that significantly advances our understanding of TNT formation beyond the current published literature. In addition, there are a number of inconsistencies and ambiguities in the approach - at least as described - that make it hard to follow the logic flow of the authors. Below I list some of the major inconsistencies and questions I had after reading through the manuscript.

1. From Figure 1b - the micro patterned substrates separated by different distances also appear to be printed as different diameters - is this true? I could find no mention of it in the text. My concern is that changing the contact area between the cell and substrate might impact the cell's ability to extend very long membrane protrusions towards its neighbors. Why not print patterns of exactly the same diameter separated by increasing distances?
2. By using micropatterned substrates the Authors hope to force the extension of TNTs using actin driven mechanisms, while eliminating the possibility that cells are form these connections by "dislodging" from the substrate. The Authors also state that the cells do not wander into the region in between the circular patterns. However, in many images featured in multiple figures, I can find examples where cells clearly extend into the space between the circular patterns (see Fig. 2.a.i for an example). The primary concern here is that the authors did not fully eliminate the possibility that cells are making direct contact and then pulling away from each other/dislodging to form TNTs. In the supplemental material, there were movies showing TNTs that had already formed, but I was unable to find examples of TNTs in the act of forming. If the authors do have timelapse data that clearly shows this, it should be front and center in this data set.
3. The authors make use of CK-666 to inhibit Arp2/3, which is thought to free up actin monomers that can be used to further elongate TNTs. Other orthogonal methods should be employed to corroborate the results from CK-666 treatment. There are other drugs that inhibit Arp2/3 (e.g. arpin, wiskostatin) and of course there is always genetic manipulation (shRNA KD).
4. The authors also employ a formin agonist, IMM-01, and the results from those experiments (Fig. S4) suggest that activating formin mDia could facilitate TNT elongation, but the authors do not follow up with direct molecular manipulation to further test this idea. Are formins (specifically mDia, the target of IMM-01) needed to elongate TNTs?
5. With the experiments shown in Figure 3, the authors apply optical tweezers to pull what they refer to as "nanotubes" from the cell surface under conditions where Arp2/3 is inhibited with CK-666. The panels appear to show that CK-666 allows actin to assemble out into a pulled nanotube more readily than control cells. However, the reporter for actin assembly in these experiments is F-Tractin, and it only partially fills the protrusion. I note here that F-Tractin appears more soluble under the CK-666 condition. Can the Authors rule out the possibility that there is just a larger soluble pool of F-Tractin probe under these conditions?
6. Related to the previous point, it is not clear to me why the Authors need to invoke the

application of external forces with an optical trap to study the enhanced elongation of TNTs under CK-666 conditions. Why don't the authors directly visualize actin accumulation and TNT elongation after treatment with this inhibitor?
7. In Figure 4, the Authors move away from TNTs and instead focus on "longer protrusions" (filopodia?) to examine the effects of EPS8 and IRSp53 on growth of these structures. The general findings here are consistent with the role of these factors in protrusion growth from previous studies.
8. The experiments in Figure 5 are performed with a truncated "bundling active" form of EPS8 allegedly lacking actin capping activity. The logic behind this choice is unclear. These experiments need to be repeated in parallel with full length WT EPS8 to allow for a full and clear interpretation of these results. Along these lines, could the Authors stain for endogenous Esp8/IRSp53 complex within TNT-connected cells?
9. Data in Figure 5 show that the Eps8dCAP/IRSp53 complex increases the vesicle transfer within TNT-connected cells from Eps8dCAP/IRSp53 donors to EBFP-H2B acceptors indicating the functionality of TNT. Interestingly, cells with overexpression of Eps8dCAP/IRSp53 and CK-666 treatment do not increase TNT-connections. Could the authors examine the stability of TNTs under this condition? The lack of increased vesicle transfer may be due to the instability of the TNT structure rather the saturation of the system.
10. Figure 5c. The data show that vesicle transfer from EpsdCAP/IRSp53 donors to EBFP-H2B acceptors increases under the overexpression of EpsdCAP/IRSp53. Are the EBFP-H2B acceptors able to form TNT? If so, could the authors show the vesicle transfer in those TNT-connected cells? The images indicate that there is no presence of TNT structures in these conditions.

2. Significance:

Significance (Required)

This study from Henderson et al. seeks to understand the mechanisms that drive tunneling nanotube (TNT) formation. TNTs are essentially giant filopodia that extend many microns from the cell surface to contact protrusions extending from neighboring cells, to establish channels that allow for exchange of biological material. The authors use an approach that involves micro patterned substrates, various inhibitors/agonists, construct overexpression experiments, and biophysical measurements with optical tweezers. The major insights from these studies are that actin monomer availability is limiting for the formation of long TNTs, and that proteins that are well known to regulate the formation of filopodia and related linear actin structures (namely EPS8 and IRSp53) promote TNT formation. These are interesting findings that are certainly consistent with the previously published literature on actin-based protrusions, and as such they should be of interest to cell biologists.

3. How much time do you estimate the authors will need to complete the suggested revisions:

Estimated time to Complete Revisions (Required)

(Decision Recommendation)

Between 3 and 6 months

4. *Review Commons* values the work of reviewers and encourages them to get credit for their work. Select 'Yes' below to register your reviewing activity at [Web of Science Reviewer Recognition Service] (formerly Publons); note that the content of your review will not be visible on Web of Science.

Web of Science Reviewer Recognition

No

Review #2

1. Evidence, reproducibility and clarity:

Evidence, reproducibility and clarity (Required)

This work is is distinguished by impressive technical feats and experimental breadth, and is another excellent contribution from the Zurzolo lab. My comments are advisory regarding more explicit descriptions and qualified conclusions.

Introduction: Please define how filopodia are distinguished from TNTs - is it length only or are there other characteristics? Do filopodia and individual TNTs have the same diameter? There is presumably a functional difference as well?

Please state the number of cells in each array spot?

Paragraph 2 of Results would benefit from a general description of procedure and rationale for assessing protrusions in the artificial setups used in this study of isolated cells. It would help to explicitly state when protrusions were assessed after fixation and when the observations were made with unfixed cells. What are the issues of concern with these methods and what aspects are relevant to general cell behavior? Isn't it important to point out that the conclusions regarding Arp2/3 inhibition and TNT formation are operational for the conditions used?

Ln 148: If filopodia are distinguished/defined by their shorter relative length, the observation that "filopodia lengths showed that a majority of filopodia were far shorter" is not informative.
Do cells with TNTs also have filopodia?

Does the negative effect of increasing array separation distance on frequency of TNTs suggest that the observations represent a steady state, and the possibility that the observed frequencies are measures of protrusion stability? If the experiments monitor the steady state, can the authors distinguish between stability and inherent ability to extend filopodia to longer

distances? Is the conclusion (ln 151) "there seems to be an upper limit to F-actin-based elongation" justified if stability or relative rates of extension and retraction are factors? Another possibility is that the observations reflect protrusion:protrusion interactions that promote stable TNTs. There is the precedent of cytoneme:cytoneme interactions associated with stable signaling contacts (Gonzalez-Mendez et al PMID: 28825565) as well as previous work from the Zurzolo lab (Sartori-Rupp et al PMID: 30664666). A kinetic analysis in real time might be very informative.

Ln 158: "cells displaying only lamellipodia accounted for 4.1% of [cells with?] TNTs examined"

2. Significance:

Significance (Required)

This work offers new insights into the cytoskeletal processes that generate long cell protrusions. The implications for understanding cell:cell interactions and signaling are fundamental and important.

3. How much time do you estimate the authors will need to complete the suggested revisions:

Estimated time to Complete Revisions (Required)

(Decision Recommendation)

Less than 1 month

4. *Review Commons* values the work of reviewers and encourages them to get credit for their work. Select 'Yes' below to register your reviewing activity at Web of Science Reviewer Recognition Service (formerly Publons); note that the content of your review will not be visible on Web of Science.

Web of Science Reviewer Recognition

No

Revision Plan

Manuscript number: RC-2022-01766
Corresponding author(s): J. Michael Henderson, Patricia Bassereau, Chiara Zurzolo

1. General Statements [optional]

This section is optional. Insert here any general statements you wish to make about the goal of the study or about the reviews.

Through our micropatterning approach we show that TNTs arising from neuronal cells are able to form at lengths longer than average filopodia (approaching 40 µm vs 5 µm) and that TNT formation at these unique length scales is enhanced when Arp2/3 activity is attenuated. This is the first quantitative evidence showing a difference between filopodia and TNTs based on the length over which actin can polymerize inside a protrusion. Furthermore, our optical tweezer-based assay permitted direct characterization of actin polymerization within nanotubes in a controlled manner and clearly demonstrated that attenuated Arp2/3 activity promotes F-actin growth to length scales that were in direct agreement with those of naturally forming TNTs. We also investigated the mechanism dictating the high-length scales TNTs are able to achieve through actin polymerization and identified Eps8 (Epidermal growth factor receptor kinase substrate 8) and the inverted Bin/Amphiphysin/Rvs (I-BAR) domain protein IRSp53 as major TNT promoters through their interaction. Proteomic analysis revealed that upon Arp2/3 inhibition, proteins enhancing actin filament turnover and depolymerization were reduced and instead heightened interactions between actin bundling factors and specifically Eps8-IRSp53 were observed. This supports our model where Arp2/3 inhibition promotes a shift to linear actin polymerization necessary for TNTs that is promoted by the Eps8-IRSp53 interaction. Overall, our work reinforces a general principle for actin network control for cellular protrusions where simple shifts in the balance between processes that inhibit actin growth versus those that promote outward growth dictate protrusion formation and the ultimate length scales protrusions like TNTs achieve.

2. Description of the planned revisions

Insert here a point-by-point reply that explains what revisions, additional experimentations and analyses are planned to address the points raised by the referees.

Reviewer 1, Point 2: By using micropatterned substrates the Authors hope to force the extension of TNTs using actin driven mechanisms, while eliminating the possibility that cells are form these connections by "dislodging" from the substrate. The Authors also state that the cells do not wander into the region in between the circular patterns. However, in many images featured in multiple figures, I can find examples where cells clearly extend into the space between the circular patterns (see Fig. 2.a.i for an example). The primary concern here is that the authors did not fully eliminate the possibility that cells are making direct contact and then pulling away from each other/dislodging to form TNTs. In the supplemental material, there were movies showing TNTs that had already formed, but I was unable to find example of TNTs in the act of forming. If the authors do have timelapse data that clearly shows this, it should be front and center in this data set.

Reply: Cells present in the inter-pattern areas arise as cell clumps in the prepared cell suspension sediment to the surface, leaving cells to straddle between patterns by remaining adhered with their neighbors that are spreading on the patterns. Though our established protocol (see Materials and Methods) uses divalent ion chelators to reduce intercellular cadherin-cadherin interactions, and 40 µm cell strainers to remove large cellular aggregates, cell clumping remains an unavoidable problem with CAD cells. Thus, as seen in Fig. 2.a.i as the reviewer noted, some random areas on the surface have large clumps of cells. However, in our TNT counting, we do not consider TNT-connected cells within these regions for our quantification given we cannot fully eliminate the possibility that they formed via dislodgment. Thus, all of our quantifications of TNT-connected cells were made on fields of view where CAD cells were well-patterned. As shown in the supplementary information where we tracked cell movement, areas where CAD cells were well-patterned showed they remained resident to their pattern and did not translocate to another (only 1 translocation event out of 56 total trajectories was observed). Additionally, as seen in **Fig. R1**, live imaging of patterned cells over the course of 30 minutes confirms that TNTs originate from actin-based processes and not from cell dislodgement in these areas.

In the transferred manuscript, we have added additional text within the **Materials and Methods, Line 690** to state that our TNT-quantifications indeed do not include these regions of cell clumping as TNT formation via cell dislodgement cannot be excluded. As part of our revision plan, we will be performing further imaging over longer timeframes than in **Fig. R1** to obtain more examples of TNT formation as recommended by the reviewer.

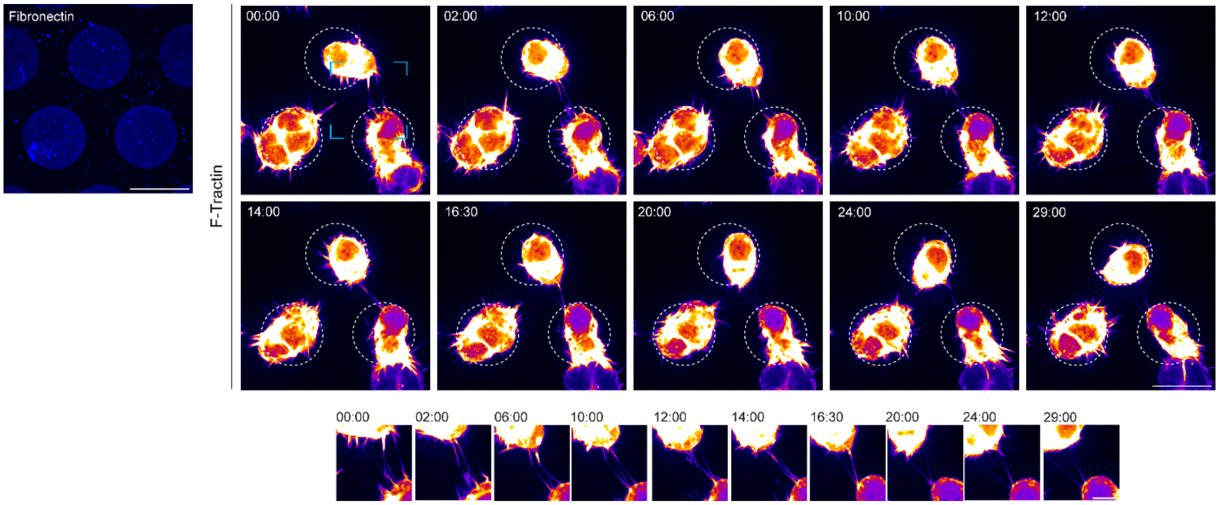

Fig. R1. TNT formation between micropatterned cells does not occur through cell dislodgment. *Selected time frames from a 30 min acquisition showing TNT-like protrusion formation between micropatterned cells expressing tdTomato F-Tractin. Images are max intensity projections of the upper stacks in the acquired Z range. White dotted circles annotate the fibronectin patterns shown on the left. Lower panels are zoom-ins of the indicated cyan box in the upper panels. Scale bars: Upper panels, 30 µm; Lower panels, 10 µm.*

Reviewer 1, Point 3: The authors make use of CK-666 to inhibit Arp2/3, which is thought to free up actin monomers that can be used to further elongate TNTs. Other orthogonal methods should be employed to corroborate the results from CK-666 treatment. There are other drugs that inhibit Arp2/3 (e.g. arpin, wiskostatin) and of course there s always genetic manipulation (shRNA KD).

Reply: We thank the reviewer for bringing to our attention the WASP inhibitor Wiskostatin, however, we have some reservations in using this drug, as instead of directly inhibiting Arp2/3 complex activity, it will act upstream to inactivate the Arp2/3 activator WASP and may therefore have unintended, off-target effects in cells. CK-666 is an accepted inhibitor (Nolen *et al*, 2009; Hetrick *et al*, 2013) and has had wide use in research such as in studying microvilli elongation (Faust *et al*, 2019), B cell receptor organization in the immunological synapse (Bolger-Munro *et al*, 2019), and in Arp2/3 activity in growth cone dynamics (Yang *et al*, 2012; Miguel-Ruiz & Letourneau, 2014). To further substantiate our CK-666 results as suggested by the reviewer, we have performed a trial siRNA knockdown of the Arp2/3 complex protein Actr3 (Arp3) in our CAD cells as it was not guaranteed to work and we have successfully achieved a strong reduction in protein expression as assessed by Western Blot (**Fig. R2**). **For revision, we will therefore corroborate our CK-666 results by assessing TNT-connected cells on *D*15 micropatterns following knockdown of Actr3.**

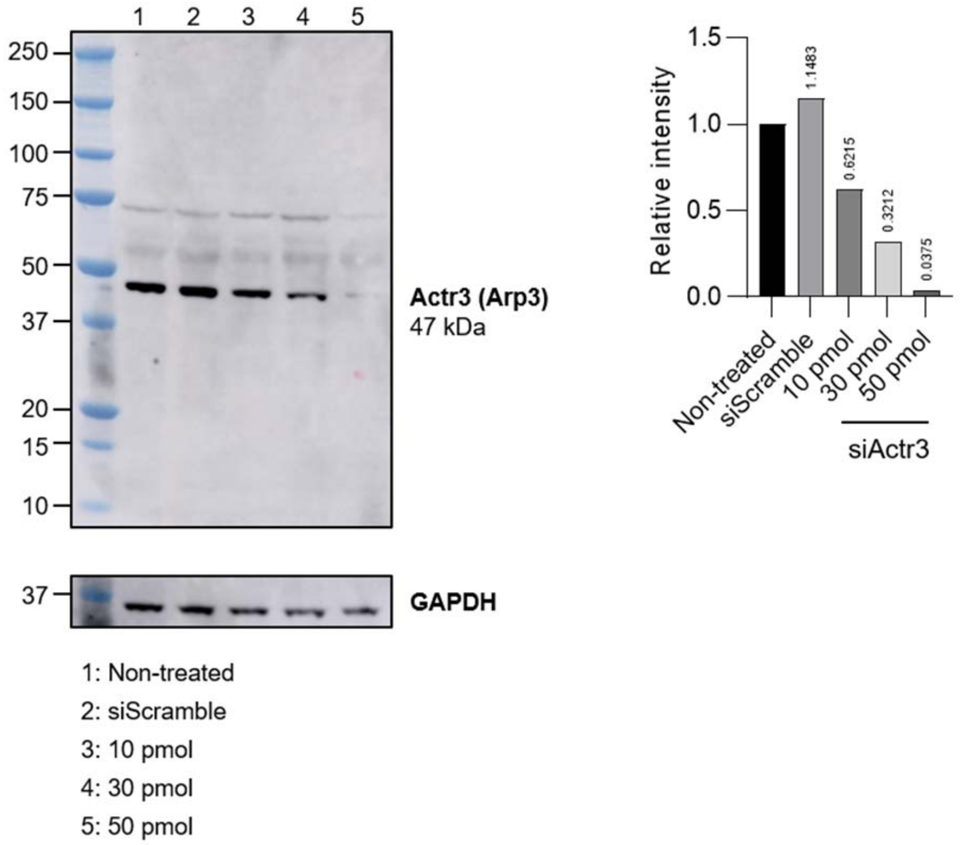

Fig. R2. siRNA knockdown of Actr3 (Arp3) in CAD cells. *Representative Western Blot (Left) and band quantification (Right) showing successful knockdown (KD) of Actr3 with increasing amounts of siRNA (Dharmacon, ON-TARGETplus Mouse Actr3 siRNA SMARTpool). A two-day KD was performed in which CAD cells on each day were transfected over 6 hrs with the indicated siRNA concentrations using Lipofectamine RNAiMAX. Primary antibodies utilized: Actr3 (1:2000, Rabbit anti-ARP3, Proteintech 13822-1-AP); GAPDH (1:5000, Rabbit anti-GAPDH, Sigma G9545).*

<u>Reviewer 1, Point 4</u>: The authors also employ a formin agonist, IMM-01, and the results from those experiments (Fig. S4) suggest that activating formin mDia could facilitate TNT elongation, but the authors do not follow up with direct molecular manipulation to further test this idea. Are formins (specifically mDia, the target of IMM-01) needed to elongate TNTs?

<u>Reply</u>: The reviewer raises an important point that we have been presently pursuing. We have screened several formins and assessed their effect on the number of TNT-connected cells through overexpression experiments using cells plated on non-micropatterned Ibidis. While mDia1 and FMNL2 appear to be the most promising candidates as there was a trend to increase the percentage of TNT-connected cells (**Fig. R3a, b**), overexpressing formins in general was troubled by poor transfection efficiency (likely given the large sizes of these proteins), and when successful in a few cells, the overexpression often leads to clear changes in the morphologies of our CAD cells that made interpretation of the data complicated.

For example, while mDia2-WT led to an increase in substrate-attached and dorsal filopodia formation with a clear localization of the protein to the tip of these protrusions (**Fig. R3c, top**), no long TNTs were observed in these conditions. This may be a result of mDia2 over-promoting protrusion numbers that are smaller in length, thus limiting TNT formation based on our results with Arp2/3 inhibition showing that G-actin availability is limiting within the cell. Moreover, the overexpression of active mDia2 lacking its DAD domain (mDia2-dDAD) showed a prominent, intracellular vesicular pattern that led to a general loss of protrusions (both filopodia and TNTs) while appearing to promote actin cable assembly (**Fig. R3c, bottom**). Similarly, overexpressing mDia3-WT showed the protein associated with vesicular compartments within the perinuclear region leading to a reduction in TNT numbers (**Fig. R3d**). This overexpression pattern of mDia3 in CAD cells resembles (and even more when mDia3 was co-transfected with active RhoD, data not shown) to the one observed in HeLa cells where RhoD, through hDia2C (or mDia3), was found to regulate endosome dynamic (Gasman *et al*, 2003). As for FMNL3, the WT form and FH1-FH2 domain-only form (i.e., active FMNL3) both led to a strong decrease in the percent of TNT-connected cells (**Fig. R3e**). Closer examination of the FH1-FH2 cells revealed cells exhibited a flatter, more adherent phenotype with prominent stress fibers that coincided with a lack of filopodia, implicating a role of FMNL3 in F-actin rearrangement in CAD cells that does not promote TNT formation.

Therefore, we could add the data with mDia1 and FMLN2 in our revised manuscript specifying that although there is a good trend, because of transfection problems there is no statistical significance.

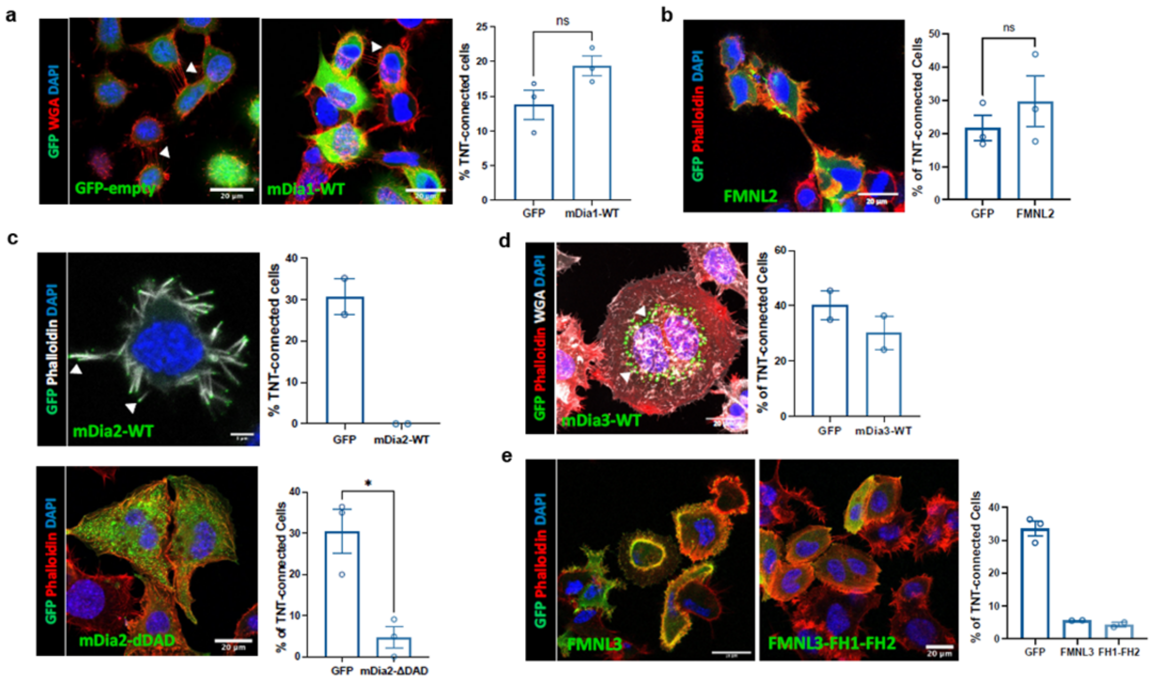

Fig. R3. Screen of formins in TNT formation. (a) *Left: Representative images of the upper stacks of control GFP-empty vector expressing cells and GFP-mDia1-WT expressing cells. White arrowheads point to TNTs. Right: Bar graph (n = 3 experiments) showing the quantification of TNT-connected cells in GFP control (13.8 ± 2.1%) and in GFP-mDia1-WT (19.4 ± 1.4%). Total cells analyzed: GFP ctrl, 367; GFP-mDia1-WT, 396. Data are represented as a mean ± SEM. Statistical analysis was performed using a t-test with Welch's correction, p = 0.1048.* ***(b)*** *Left: Representative image of the upper stacks of FMNL2-GFP expressing cells. Right: Bar graph (n = 3 experiments) showing the quantification of TNT-connected cells in GFP ctrl (21.6 ± 3.8%) and in FMNL2-GFP (29.6 ± 7.6%). Total cells analyzed: GFP ctrl, 335; FMNL2-GFP, 223. Data are represented as mean ± SEM. Statistical analysis was performed using a t-test with Welch's correction, p = 0.4223.* ***(c)*** *Top: Representative images of the upper stacks of GFP-mDia2-WT expressing cells, along with a bar graph (n = 2 experiments) showing the quantification of TNT-connected cells in GFP ctrl (30.6 ± 4.4%) and in GFP-mDia2-WT (0.0 ± 0.0%). Total cells analyzed: GFP ctrl, 59; GFP-mDia2-WT, 40. Data are represented as mean ± SEM. White arrowheads indicate the mDia2 formin at the tip of filopodia. Bottom: Representative images of the maximum stack projection of GFP-mDia2-dDAD (i.e., activated mDia2) expressing cells, along with a bar graph (n = 3 experiments) showing the quantification of TNT-connected cells in GFP ctrl (30.5 ± 5.2%) and in mDia2-dDAD (4.7 ± 2.6%). Total cells analyzed: GFP ctrl, 144; GFP-mDia2-dDAD, 140. Data are represented as a mean ± SEM. Statistical analysis was performed using a t-test with Welch's correction, p = 0.0227.* ***(d)*** *Left: Representative image of the maximum stack projection of GFP-mDia3-WT expressing cells. White arrowheads indicate the accumulation of mDia3 in vesicular compartments. Right: Bar graph (n = 2 experiments) showing the quantification of TNT-connected cells in GFP ctrl (40.2 ± 5.2%) and in GFP-mDia3-WT (30.2 ± 6.1%). Total cells analyzed: GFP ctrl, 95; GFP-mDia3-WT, 102. Data are represented as a mean ± SEM.* ***(e)*** *Left: Representative images of the maximum stack projection of FMNL3-GFP and GFP-FMNL3-FH1-FH2 (i.e., an activated FMNL3 construct containing only the FH1 and FH2 domains) expressing cells. Right: Bar graph from at least two experiments showing the quantification of TNT-connected cells in GFP ctrl (33.5 ± 2.2%), FMNL3-GFP (5.6 ± 0.05%) and in GFP-FMNL3-FH1-FH2 (4.3*

± 0.7%). Total cells analyzed: GFP ctrl, 146; FMNL3-GFP, 71; GFP-FMNL3-FH1-FH2, 95. Data are represented as a mean ± SEM.

In Addition, as our proteomic analysis identified only a single formin-family protein, Delphilin (Grid2ip), in the proteome of IRSp53 (Fig. 7), **our revision plan to address this reviewer's point is to assess the role of Delphilin in TNTs** given the unencouraging results observed with the other formins we screened (**Fig. R3**). However, as we have found it difficult to overexpress formins at appreciably high levels particularly for vesicle transfer assays, we will instead pursue a knockdown approach with Delphilin and assess if both TNT-connected cells and the number of vesicles transferred in a co-culture assay are reduced, a result expected if indeed Delphilin participates in TNT formation/function in our neuronal CAD cell model. **Fig. R4** shows the results of a trial Delphilin KD experiment and indicates a tendency for TNTs to reduce upon its diminished expression. Unfortunately, we were unable to quantify the extent of Delphilin KD by Western Blot as the primary antibody we purchased did not work (Santa Cruz, anti-Grid2ip (A-4), SC-390952). Given the cell phenotypes between control and KD samples look relatively similar, it is likely in this trial experiment we did not achieve a high enough KD of Delphilin to get a significant reduction in the percentage of TNT-connected cells. However, we are confident that our ongoing assessment will reveal a role for Delphilin in TNTs.

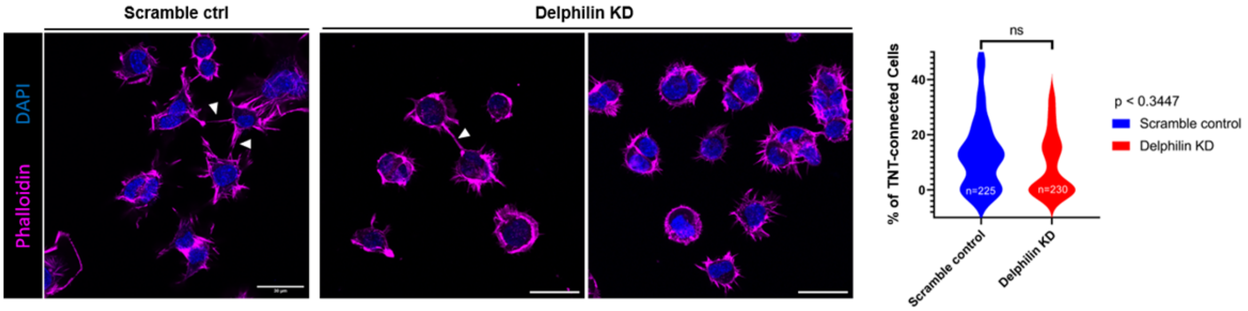

Fig. R4. Effect of Delphilin knockdown on TNTs.

Left: Representative images of the upper stacks of Scramble control and Delphilin KD cells (50 pmol, 1-day knockdown, Sigma-Aldrich, MISSION esiRNA, EMU010181) plated on D15 micropatterns (fibronectin was non-fluorescent). White arrowheads point to TNTs. Scale bars, 30 µm. Right: Violin plot (n = 1 experiment) showing the quantification of TNT-connected cells in Scramble ctrl (mean, 13.5%) and Delphilin KD (mean, 9.1%). Total cells analyzed: Scramble ctrl, 225; Delphilin KD, 230. Statistical analysis was performed using an unpaired t-test.

Reviewer 1, Point 7: In Figure 4, the Authors move away from TNTs and instead focus on "longer protrusions" (filopodia?) to examine the effects of EPS8 and IRSp53 on growth of these structures. The general findings here are consistent with the role of these factors in protrusion growth from previous studies.

Reply: We felt it was pertinent to do a comparison in our specific cell line, even if similar experiments have been done in other cells before, on the separate overexpression of IRSp53 and Eps8. Given our live imaging was done on isolated cells to observe the possible extent to which such IRSp53- and Eps8-driven protrusions could reach at any given moment, we could not call them TNTs as no “acceptor” cell was nearby for the protrusion to connect with. However, our results presented in Fig. 4 do provide new information in regards to how both IRSp53 and Eps8 must be released from their involvement in branched actin networks in order to drive actin elongation that is able to reach TNT length scales, a result

that is consistent with our proteomics analysis. Additionally, as part of our revision plan we will be conducting further experiments using our optical tweezer assay to monitor the co-recruitment of both Eps8 and IRSp53 in pulled nanotubes and how this coincides with the amount of F-actin growth extending within the nanotube. Preliminary experiments in **Fig. R5** already confirm that both Eps8 and IRSp53 are present in these pulled nanotubes. Future experiments will be using a stable cell line we are currently generating using lentiviral transduction to express EGPF-F-Tractin, IRSp53-mCherry, and iRFP-Eps8-ΔCAP (cloned in-house from the EGFP-Eps8 constructs we had been using).

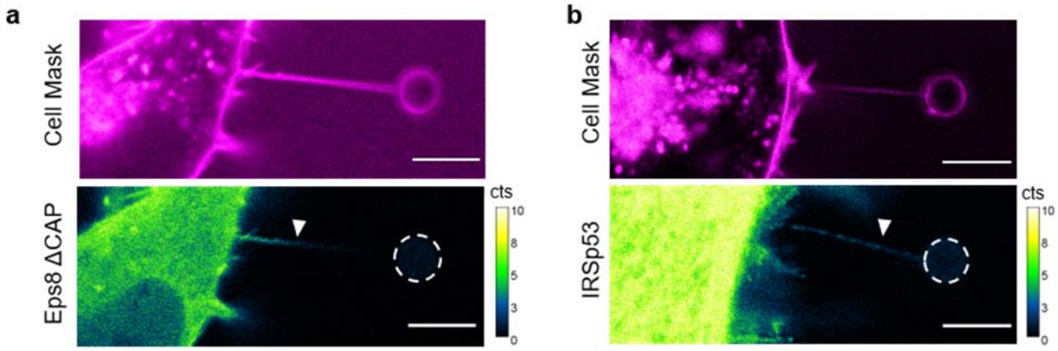

Fig. R5. Eps8 and IRSp53 are recruited into optically pulled nanotubes.
Representative confocal images of nanotubes pulled form Eps8ΔCAP ***(a)*** *and IRSp53* ***(b)*** *expressing CAD cells. White arrowheads point to the protein-positive nanotubes. Displayed intensities for the protein channel were set between 0 and 10 photon counts (cts) for better visualization.Scale bars, 5 μm.*

Reviewer 1, Point 8: The experiments in Figure 5 are performed with a truncated "bundling active" form of EPS8 allegedly lacking actin capping activity. The logic behind this choice is unclear. These experiments need to be repeated in parallel with full length WT EPS8 to allow for a full and clear interpretation of these results. **Along these lines, could the Authors stain for endogenous Esp8/IRSp53 complex within TNT-connected cells?**

Reply: Please note that on Page 16 below, we reply to the beginning half of this reviewer's point.

In regards to staining for endogenous Eps8/IRSp53, we have performed a preliminary immunofluorescence, and as shown in **Fig. R6**, TNTs found in the upper stacks connecting cells were found to be positive for both Eps8 and IRSp53. As part of our revision plan, we will continue our immunofluorescence on cells instead plated on *D*15 micropatterns and will use super resolution microscopy (Live-SR or SIM) to obtain higher resolution images of the Eps8 and IRSp53 signals within the TNTs.

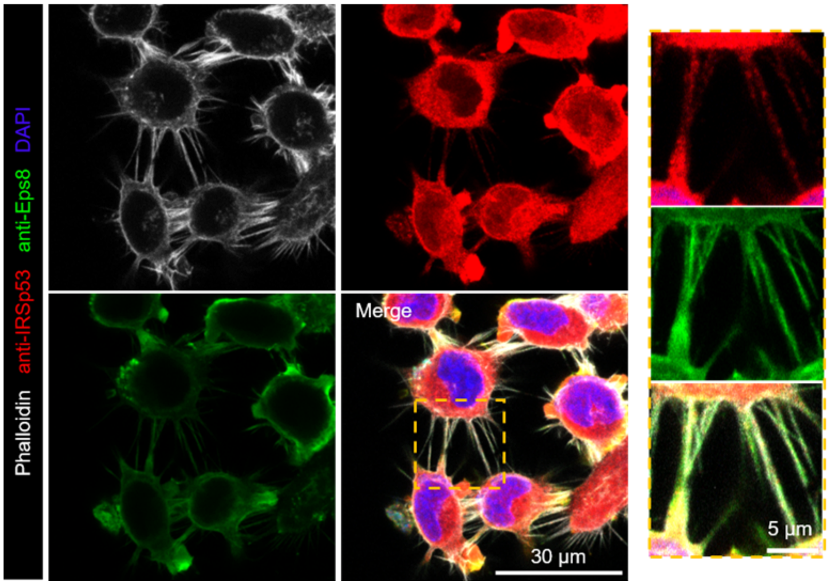

Fig. R6. Immunofluorescence revels Eps8 and IRSp53 are endogenously present in TNTs. *Representative confocal images of the upper stacks of CAD cells connected by TNTs positive for endogenous Eps8 and IRSp53. CAD cells were plated on non-micropatterned Ibidi dishes the day before sample processing. Cells were fixed in 4% PFA, permeabilized with 0.05% Saponin, and blocked in 2% BSA. Primaries: 1:100 anti-Eps8 (Mouse IgG_1, BD Biosciences, 610143); 1:500 anti-IRSp53 (Rabbit polyclonal, Atlas, HPA023310). Dashed yellow box correspond to zoom-in subpanels on the right.*

Reviewer 1, Point 9: Data in Figure 5 show that the Eps8dCAP/IRSp53 complex increases the vesicle transfer within TNT-connected cells from Eps8dCAP/IRSp53 donors to EBFP-H2B acceptors indicating that functionality of TNT. Interestingly, cells with overexpression of Eps8dCAP/IRSp53 and CK-666 treatment do not increase TNT-connections. Could the authors examine the stability of TNTs under this condition? The lack of increase vesicle transfer may be due to the instability of the TNT structure rather the saturation of the system.

Reply: It is not clear why CK-666 addition to cells expressing Eps8/IRSp53 would lead to greater TNT instability as the reviewer suggests given the co-expression of both proteins promotes more TNTs. For example, our preliminary siRNA silencing experiments further confirm a function role of IRSp53 in TNT formation and their functionality to facilitate vesicle transfer (**Fig. R7**). Thus, it is difficult to imagine how their expression leads to protrusion instability. What we hypothesize may be happening is that the over-expression of Eps8 and IRSp53 already promotes TNTs on these *D*15 substrates to reach their maximal lengths in the absence of CK-666. Thus, the addition of the drug doesn't further enhance the number of TNT-connected cells, as the connections have already reached a length needed to connect cells separated by the 15-micron spacing on the surface, and hence no additional increase in vesicle transfer is observed. Our revision plan is to quantify the length distributions of protrusions and see whether Eps8 and IRSp53 combined shift the distribution to length scales achieved when wild-type cells are alone treated with CK-666. Additionally, our proposed optical tweezer experiments addressing **Reviewer 1, Point 7** (Page 6) will also be able to confirm whether Eps8 and IRSp53 together promote greater F-actin extension in the absence of CK-666.

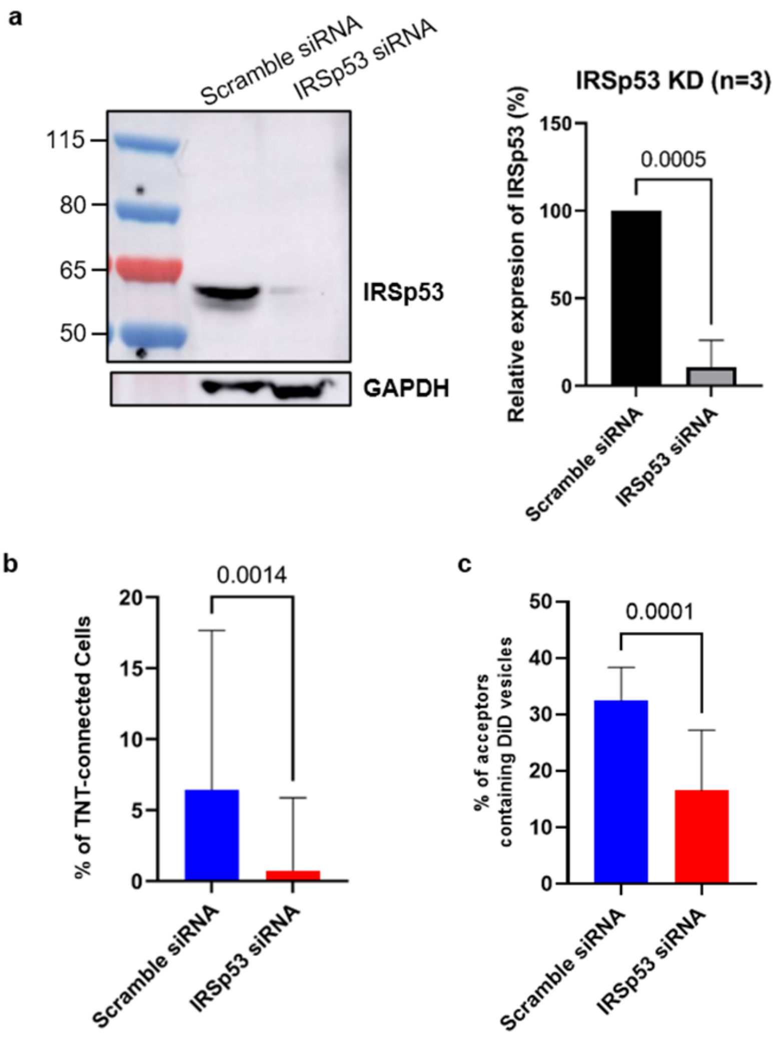

Fig. R7. Effect of IRSp53 knockdown on TNT-connected cells and vesicle transfer. (a) *Representative Western Blot (Left) and band quantification (Right) showing successful knockdown (KD) of IRSp53 (50 pmol, 1-day knockdown, Dharmacon, ON-TARGETplus Mouse IRSp53 siRNA SMARTpool).* ***(b)*** *Bar graph (n = 3 experiments) showing the quantification of TNT-connected cells in Scramble ctrl (mean, 6.4%) and IRSp53 KD (mean, 0.7%). Total cells analyzed: Scramble ctrl, 490; IRSp53 KD, 437.* ***(c)*** *Bar graph (n = 1 experiment) showing the quantification of acceptor cells having received DID-labelled vesicles from Scramble ctrl (mean, 32.5%) or IRSp53 (mena, 16.6%) donor cells.*

<u>Reviewer 1, Point 10</u>: Figure 5c. The data show that vesicle transfer from EpsdCAP/IRSp53 donors to EBFP-H2B acceptors increases under the overexpression of EpsdCAP/IRSp53. Are the EBFP-H2B acceptors able to form TNT? If so, could the authors show the vesicle transfer in those TNT-connected cells? The images indicate that there is no presence of TNT structures in these conditions.

Reply: We agree and as part of our revision plan, we will re-prepare these Eps8dCAP/IRSp53 and H2B co-cultures on *D*15 micropatterns and provide better images that showcase this event.

Reviewer 2, Point 1: Introduction: Please define how filopodia are distinguished from TNTs - is it length only or are there other characteristics? Do filopodia and individual TNTs have the same diameter? There is presumably a functional difference as well?

Reply: In our revision plan, we will update the introduction to have an expanded description on the similarities and differences between filopodia and TNTs, and the implications these have on their differing functionality. Similar to filopodia, TNTs are thin structures having diameters frequently in the range of 100 to 300 nm (Sartori-Rupp et al, 2019). The widths of both of these protrusions are determined by the number of actin filaments residing in the core of the protrusion which average between 10−30 filaments in filopodia, for example. In contrast to filopodia— which harbor adhesion molecules such as integrins that permit their interaction with the extracellular matrix and enable cell adhesion and migration (Galbraith *et al*, 2007; Partridge & Marcantonio, 2006)—TNTs are non-surface adherent structures that cytoplasmically connect distant cells for the transfer of cargo of various sizes, encompassing not only small ions, but also larger organelles. Our recent cryo-EM work (Sartori-Rupp et al, 2019) supports that vesicular cargo is transported in TNTs while such cargo is absent in CAD cell filopodia, mirroring other past reports that show no vesicles nor other organelles within filopodia arising from Dictyostelium (Medalia et al, 2007), HeLa (Mageswaran et al, 2021), HUVEC (Almagro et al, 2010) and NG108-15 cells (Aramaki et al, 2016). Thus, a key distinguishing characteristic of TNTs is their functional ability to transfer cargo that is afforded by their open-endedness.

Reviewer 2, Point 2: Please state the number of cells in each array spot?

Reply: We are a bit unsure what the reviewer was specifically asking here, but if the reviewer was referring to the approximate number of cell-occupied micropatterns associated with each datapoint in Whisker plots for TNT quantification (e.g., Figs. 1f and 5b), this information is provided within the respective figure captions. If, however, the reviewer was referring to the average number of CAD cells per fibronectin patch, then this data is presented in Fig. S1b. **Nevertheless, as part of our revision plan, we will provide a supplementary Excel file that will list all the pertinent information associated with each Whisker plots, bar graphs, etc. presented in the manuscript figures.**

Reviewer 2, Point 3: Paragraph 2 of Results would benefit from a general description of procedure and rationale for assessing protrusions in the artificial setups used in this study of isolated cells. It would help to explicitly state when protrusions were assessed after fixation and when the observations were made with unfixed cells. What are the issues of concern with these methods and what aspects are relevant to general cell behavior? Isn't it important to point out that the conclusions regarding Arp2/3 inhibition and TNT formation are operational for the conditions used?

Reply: While all the pertinent information can be presently found in the Materials and Methods, and the Supplementary Information, as suggested by the reviewer, we will add additional descriptions in the results of the micropatterning procedure that includes specifics on how long cells were cultured on the patterns prior to fixation for TNT quantification.

Reviewer 2, Point 4: Ln 148: If filopodia are distinguished/defined by their shorter relative length, the observation that "filopodia lengths showed that a majority of filopodia were far shorter" is not informative. Do cells with TNTs also have filopodia?

Reply: The reviewer is not wrong that this phrase on Line 148 is tautological. However, as our work is the first to demonstrate unequivocally that TNTs originating through actin polymerization clearly reach lengths greater than filopodia, and provide quantitative statistically valid data, we felt it was necessary to demonstrate that filopodia display lengths that are far shorter (Fig. 1g). As shown in Fig 2, for example, TNTs and filopodia can co-exist on the same cell.

Reviewer 2, Point 5: Does the negative effect of increasing array separation distance on frequency of TNTs suggest that the observations represent a steady state, and the possibility that the observed frequencies are measures of protrusion stability? If the experiments monitor the steady state, can the authors distinguish between stability and inherent ability to extend filopodia to longer distances? Is the conclusion (ln 151) "there seems to be an upper limit to F-actin-based elongation" justified if stability or relative rates of extension and retraction are factors? Another possibility is that the observations reflect protrusion:protrusion interactions that promote stable TNTs. There is the precedent of cytoneme:cytoneme interactions associated with stable signaling contacts (Gonzalez-Mendez et al PMID: 28825565) as well as previous work from the Zurzolo lab (Sartori-Rupp et al PMID: 30664666). A kinetic analysis in real time might be very informative.

Reply: Our interpretation is that the negative effect of increasing separation distance reflects a decrease in the probability that a given TNT can reach such extreme lengths. This decrease in probability is supported by our optical tweezer assay which shows that F-actin growth is limited to <5 μm within the pulled nanotube in the absence of Arp2/3 inhibition, but reaches 15 μm when Arp2/3 activity is inhibited. **As part of our revision**, we will be continuing to explore this hypothesis by performing additional optical tweezer experiments to pull instead longer nanotubes reaching 40 μm in length. These longer nanotubes will permit us to assess the probable lengths that F-actin is able to achieve in control vs. CK-666 treated conditions and more easily record any instances in which F-actin grows to extreme lengths (>20 μm).

To address the reviewer's hypothesis that the negative effect of increasing separation distance reflects a change in TNT stability requires assessing the total lifetime of a TNT from its initiation to its eventual connection to a recipient cell, and finally to the point at which it detaches/breaks from the recipient cell. Such experiments would not only involve long imaging sessions, but be practically difficult to perform as we cannot predict where in a given sample a TNT forms at any given time. Thus, such experiments would be statistically poor to record changing lifetimes as a function of different TNT lengths.

However, the live imaging experiments proposed to address **Reviewer 1, Point 2** will provide information on the growth of TNTs and their dynamic history, such if they tend to not retract as frequently which may in turn lead to more events of elongation, that could assess some of the points raised by this reviewer.

3. Description of the revisions that have already been incorporated in the transferred manuscript

Please insert a point-by-point reply describing the revisions that were already carried out and included in the transferred manuscript. If no revisions have been carried out yet, please leave this section empty.

Reviewer 1, Point 1: From Figure 1b - the micro patterned substrates separated by different distances also appear to be printed as different diameters - is this true? I could find no mention of it in the text. My concern is that changing the contact area between the cell and substrate might impact the cell's ability to extend very long membrane protrusions towards its neighbors. Why not print patterns of exactly the same diameter separated by increasing distances?

Reply: The representative micropatterns in Fig 1b are not of different sizes; the image of the *D*40 substrate is slightly zoomed out as compared to the *D*15 substrate in order to show several patterns within the field of view given the greater 40 μm separation distance. In our study, we kept the micropattern diameter constant at 31 μm. This information was originally stated in the figure captions for Fig.1 and Fig. S1. To avoid future reader confusion, we have added small clarifications in the **Results, Line 132 and Line 134**.

Reviewer 1, Point 5: With the experiments shown in Figure 3, the authors apply optical tweezers to pull what they refer to as "nanotubes" from the cell surface under conditions where Arp2/3 is inhibited with CK-666. The panels appear to show that CK-666 allows actin to assemble out into a pulled nanotube more readily than control cells. However, the reporter for actin assembly in these experiments is F-Tractin, and it only partially fills the protrusion. I note here that F-Tractin appears more soluble under the CK-666 condition. Can the Authors rule out the possibility that there is just a larger soluble pool of F-Tractin probe under these conditions?

Reply: F-Tractin is a well-established reporter for F-actin in living cells and we choose to use it given it more accurately reproduces the visualization of phalloidin-stained F-actin structures in cells unlike Lifeact or Utrophin reporters (Belin *et al*, 2014). The reviewer raises the concern that CK-666 treatment appears to solubilize F-Tractin, creating a ctyoplasmic pool of F-Tractin that then determines the fluorescent profile measured within the pulled nanotube, however, we can rule out this diffusion-dependent effect and confirm F-Tractin is accurately reflecting F-actin development within the nanotubes. First, as seen in **Fig. R8**, cells with different F-Tractin levels within their cell body exhibited identical profiles within the pulled nanotubes that reached an endpoint at 10 μm within the nanotube (see Example 1 and Example 2). If the profiles within these tubes were completely dependent on a cytosolic pool of unbound F-Tractin diffusing into the nanotube, then the length scale of the profile would therefore be set by the bulk concentration of soluble F-Tractin via Fick's Diffusion Law. Thus, cells with more soluble F-Tractin would exhibit longer profiles and vice versa for cells with lower amounts. However, we clearly observe identical endpoints of the F-Tractin fluorescence within the nanotube for cells of differing cellular F-Tractin levels with only a difference in the absolute intensities between the profiles, thus ruling out such a diffusion-mediated effect and. Furthermore, if a diffusion-mediated effect predominated, then cells with comparable F-Tractin levels should always exhibit identical endpoints in their profiles. However, as seen when comparing Example 1 with Example 3, different endpoints where observed supporting the conclusion that F-actin grew to different lengths in these cases and that F-Tractin is reporting on the extent of F-actin growth within the

nanotube. Secondly, **new Kymograph analysis provided in Fig. S5** of the transferred manuscript shows F-Tractin dynamics that clearly arise from the presence of F-actin within the nanotube (e.g., stationary, non-growth phases that transition to a phase of growth within the nanotube) that would otherwise be absent in a diffusion-mediated case where F-Tractin would quickly establish a stable profile that does not vary through time.

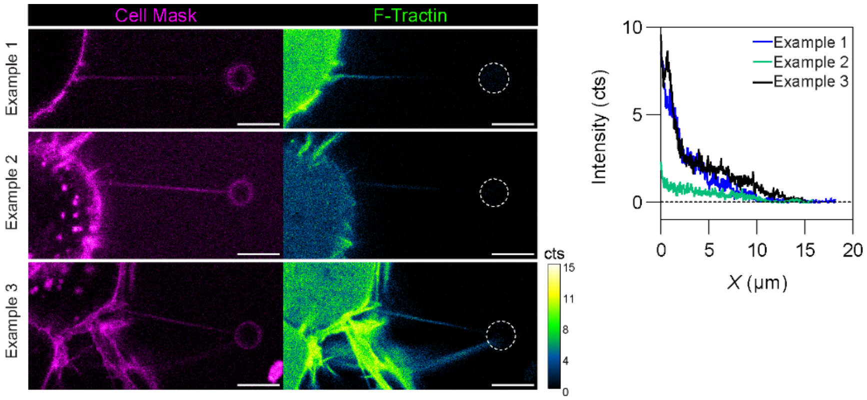

Fig. R8. Cellular F-Tractin level does not establish the resulting fluorescent profile measured in pulled nanotubes. *(Left) Representative confocal images of nanotubes pulled form CK-666-treated CAD cells having different cellular F-Tractin intensity levels. Images in the F-Tractin channel were identically scaled between 0 and 15 photon counts (cts) for visual comparison. (Right) Plot of the intensity profiles within the respective pulled nanotubes measured along the length of the nanotube (X) from its base to its end attached to the trapped bead. Scale bars, 5 µm.*

In the transferred manuscript, we have included our new kymograph analysis as a figure in the supplementary information (**Fig. S5**) and made reference to it in the **Results, Line 187** and **Line 189**.

New Fig. S5

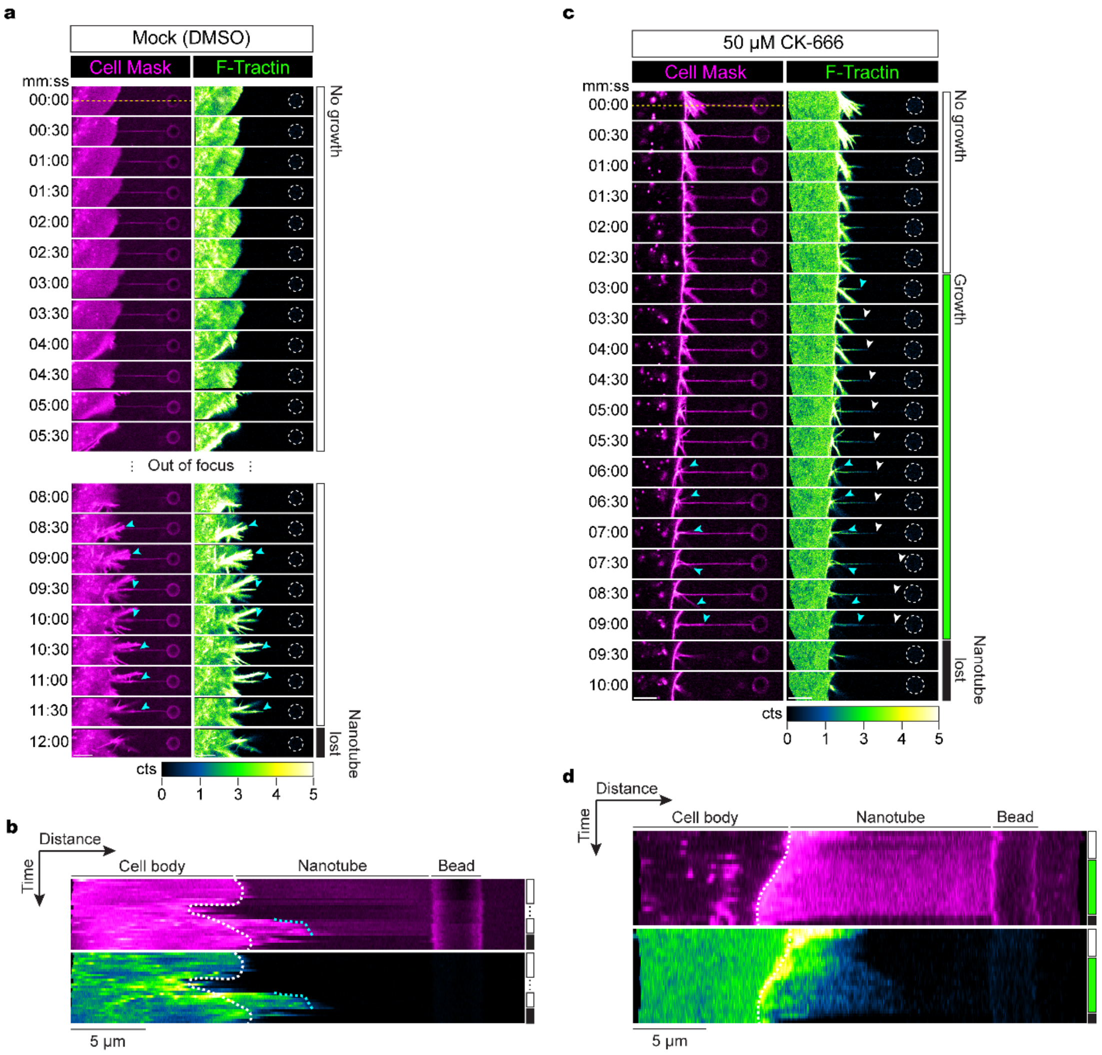

Fig. S5: Spatiotemporal dynamics of F-actin development in pulled nanotubes.
(a, b) Time-lapse montage of a nanotube pulled from a DMSO-treated cell (Movie S5) along with a corresponding kymograph showing no actin growth within the nanotube. **(c, d)** Time-lapse montage of a nanotube pulled from a CK-666-treated cell (Movie S6) along with a corresponding kymograph showing actin growth within the nanotube. Displayed intensities for the F-Tractin channel were set between 0 and 5 photon counts (cts) for better visualization. Kymographs were taken along the nanotube axis (yellow dotted line in **a** and **c**). White arrowheads annotate the progression of actin development within the nanotube, while cyan arrowheads annotate obstructing protrusions that grew along the pulled nanotube. Dotted white and cyan lines in the kymographs mark the cell body edge and obstructing protrusions, respectively. Scale bars, 5 µm.

<u>Reviewer 1, Point 6</u>: Related to the previous point, it is not clear to me why the Authors need to invoke the application of external forces with an optical trap to study the enhanced elongation of TNTs under CK-666 conditions. Why don't the authors directly visualize actin accumulation and TNT elongation after treatment with this inhibitor?

<u>Reply</u>: We use our tube pulling experiment to create a structure that resembles a protrusion to independently measure actin growth in a protrusion-like structure. In "real" protrusions, the length of the protrusion is a direct read-out of the actin filament length, but their growth can be perturbed by other parameters, for instance, membrane tension that can restrain actin growth. Thus, this nanotube pulling assay permits a controlled and high throughput means for directly measuring the true extent Arp2/3 inhibition shifts the cellular system towards favoring longer filament formation. With this assay we were able to clearly characterize a difference in the most probable F-actin length scales achievable in control vs. CK-666-treated conditions. In control conditions, F-actin length on average did not extend more than 3–5 μm within the nanotubes which nicely corresponded to the filopodia lengths we measured for CAD cells (Fig. 1g). Contrastingly, Arp2/3 inhibition showed the mean F-actin length was instead shifted to far longer distances.

In the transferred manuscript, we have updated the text beginning on **Line 181 in the Results** to better emphasize our rationale for using this optical tweezer approach, expanded the explanation of the results presented in Fig. 3, and added a new kymograph analysis presented in **Fig. S5**.

<u>Reviewer 2, Point 6</u>: Ln 158: "cells displaying only lamellipodia accounted for 4.1% of [cells with?] TNTs examined"

<u>Reply</u>: Based on the reviewer's suggestion, we have corrected this to now read: (Line 157) "Phenotypic characterization of TNT-connected cells on *D*15 micropatterns revealed that cells displaying only lamellipodia accounted for 4.1% of the examined cells with TNTs.

<u>Minor Revisions:</u>
- While Eps8 was properly identified previously in the volcano plot in Fig. 7a (upper panel), we found that Eps8 was erroneously missing as a protein node in the STRING-generated network for IRSp53 in Fig. 7b. We have corrected this issue and have updated the STRING-generated network in Fig. 7b to include a protein node for Eps8.

4. Description of analyses that authors prefer not to carry out

Please include a point-by-point response explaining why some of the requested data or additional analyses might not be necessary or cannot be provided within the scope of a revision. This can be due to time or resource limitations or in case of disagreement about the necessity of such additional data given the scope of the study. Please leave empty if not applicable.

Reviewer 1, Point 8: The experiments in Figure 5 are performed with a truncated "bundling active" form of EPS8 allegedly lacking actin capping activity. The logic behind this choice is unclear. These experiments need to be repeated in parallel with full length WT EPS8 to allow for a full and clear interpretation of these results. Along these lines, could the Authors stain for endogenous Esp8/IRSp53 complex within TNT-connected cells?

Reply: We apologize to the reviewer for a minor misunderstanding, but the Eps8-ΔCAP (bundling active) mutant is not a truncated version. In actuality, the mutant was made through single-point mutations within Eps8's C-terminal region (V689D, L693D) to impair its actin capping ability. While we did point readers to the original reference from the G. Scita lab for further details into its construction (45. Hertzog *et al*, 2010), to avoid further confusion on this matter, we have added an additional explanatory phrase in the transferred manuscript within the **Materials and Methods, Line 429**.

Our rationale for using Eps8-ΔCAP is that protrusions in general require bundled actin filaments that can collectively generate enough localized force to overcome the resistance imposed by the tension of the plasma membrane to grow outwards effectively (Mogilner & Rubinstein, 2005). Otherwise, poor or inefficient bundling of the core filaments within a nascent protrusion would buckle under the strain of the membrane tension and no outward growth would happen. Moreover, our past study showed Eps8-ΔCAP had a pronounced effect in promoting not only the percentage of TNT-connected cell, but also the number of vesicles transferred in a donor-acceptor co-culture assay (Delage *et al*, 2016). To better emphasize our logic for the use of Eps8-ΔCAP, we have added new text within the **Results on Line 211 and Line 227**.

We respectively disagree with the reviewer that re-preforming all experiments in Fig. 5 with Eps8-WT would provide a clearer interpretation of the results. First, we know that using Eps8 in its bundling active version does not impair any interaction with IRSp53 (**Fig. R9**). Beyond the fact that this would be a big endeavor, secondly, such experiments would not add to the interpretation but complicate them as the expression of Eps8 WT would create a scenario in which it could act predominately in its capping function, reducing F-actin elongation and hence limit overall protrusion formation. Thus, we do not wish to perform these suggested experiments.

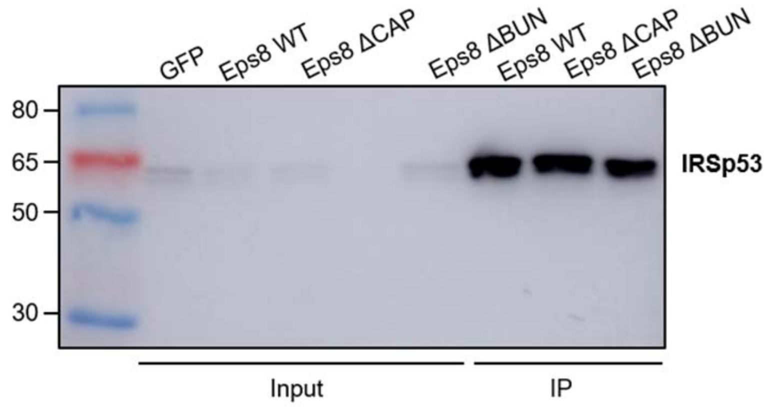

Fig. R9. Eps8 interacts with IRSp53 irrespective if its bundling or capping active form. *Representative Western Blot showing GFP-trap immunoprecipitation bands of the pray protein IRSp53 in the Eps8 WT, Eps8 ΔCAP and Eps8 ΔBUN bait precipitates (right) and input lysates (left).*

References:

Almagro S, Durmort C, Chervin-Pétinot A, Heyraud S, Dubois M, Lambert O, Maillefaud C, Hewat E, Schaal JP, Huber P, *et al* (2010) The Motor Protein Myosin-X Transports VE-Cadherin along Filopodia To Allow the Formation of Early Endothelial Cell-Cell Contacts. *Mol Cell Biol* 30: 1703–1717

Aramaki S, Mayanagi K, Jin M, Aoyama K & Yasunaga T (2016) Filopodia formation by crosslinking of F-actin with fascin in two different binding manners. *Cytoskeleton* 73: 365–374

Belin BJ, Goins LM & Mullins RD (2014) Comparative analysis of tools for live cell imaging of actin network architecture. *BioArchitecture* 4: 189–202

Bolger-Munro M, Choi K, Scurll JM, Abraham L, Chappell RS, Sheen D, Dang-Lawson M, Wu X, Priatel JJ, Coombs D, *et al* (2019) Arp2/3 complex-driven spatial patterning of the BCR enhances immune synapse formation, BCR signaling and B cell activation. *eLife* 8: e44574

Delage E, Cervantes DC, Pénard E, Schmitt C, Syan S, Disanza A, Scita G & Zurzolo C (2016) Differential identity of Filopodia and Tunneling Nanotubes revealed by the opposite functions of actin regulatory complexes. *Sci Rep* 6: 39632

Faust JJ, Millis BA & Tyska MJ (2019) Profilin-Mediated Actin Allocation Regulates the Growth of Epithelial Microvilli. *Curr Biol* 29: 3457-3465.e3

Galbraith CG, Yamada KM & Galbraith JA (2007) Polymerizing actin fibers position integrins primed to probe for adhesion sites. *Science* 315: 992–995

Gasman S, Kalaidzidis Y & Zerial M (2003) RhoD regulates endosome dynamics through Diaphanous-related Formin and Src tyrosine kinase. *Nat Cell Biol* 5: 195–204

Hetrick B, Han MS, Helgeson LA & Nolen BJ (2013) Small Molecules CK-666 and CK-869 Inhibit Actin-Related Protein 2/3 Complex by Blocking an Activating Conformational Change. *Chem Biol* 20: 701–712

Mageswaran SK, Yang WY, Chakrabarty Y, Oikonomou CM & Jensen GJ (2021) A cryo–electron tomography workflow reveals protrusion-mediated shedding on injured plasma membrane. *Sci Adv* 7: eabc6345

Medalia O, Beck M, Ecke M, Weber I, Neujahr R, Baumeister W & Gerisch G (2007) Organization of Actin Networks in Intact Filopodia. *Curr Biol* 17: 79–84

Miguel-Ruiz JES & Letourneau PC (2014) The Role of Arp2/3 in Growth Cone Actin Dynamics and Guidance Is Substrate Dependent. *J Neurosci* 34: 5895–5908

Mogilner A & Rubinstein B (2005) The Physics of Filopodial Protrusion. *Biophys J* 89: 782–795

Nolen B, Tomasevic N, Russell A, Pierce D, Jia Z, CD M, Hartman J, Sakowicz R & Pollard T (2009) Characterization of two classes of small molecule inhibitors of Arp2/3 complex. *Nature* 460: 1031–1034

Partridge MA & Marcantonio EE (2006) Initiation of attachment and generation of mature focal adhesions by integrin-containing filopodia in cell spreading. *Mol Biol Cell* 17: 4237–4248

Sartori-Rupp A, Cervantes DC, Pepe A, Gousset K, Delage E, Corroyer-Dulmont S, Schmitt C, Krijnse-Locker J & Zurzolo C (2019) Correlative cryo-electron microscopy reveals the structure of TNTs in neuronal cells. *Nat Commun* 10: 342

Yang Q, Zhang X-F, Pollard T & Forscher P (2012) Arp2/3 complex–dependent actin networks constrain myosin II function in driving retrograde actin flow. *J Cell Biol* 197: 939–956

Dear Chiara,

Thank you again for submitting your manuscript for consideration by the EMBO Journal. I have now read your manuscript, the reviewer comments and your response to them, as well as discussed your revision plan with my colleagues and an external scientific advisor. Based on these discussions, I would like to invite you to submit a revised version of the manuscript along the lines indicated in your revision plan. Please note that since referee #1 was not responsive, I will likely have to involve an arbitrating referee in the assessment of the revised manuscript to judge your responses to the points raised by this reviewer.

We generally allow three months as standard revision time. As a matter of policy, competing manuscripts published during this period will not negatively impact on our assessment of the conceptual advance presented by your study. However, please contact me as soon as possible upon publication of any related work to discuss the appropriate course of action. Should you foresee a problem in meeting this three-month deadline, please let us know in advance in order to arrange an extension.

When preparing your letter of response to the referees' comments, please bear in mind that this will form part of the Review Process File and will therefore be available online to the community. For more details on our Transparent Editorial Process, please visit our website: https://www.embopress.org/page/journal/14602075/authorguide#transparentprocess. Please also see the attached instructions for further guidelines on preparation of the revised manuscript.

Please feel free to contact me if you have any further questions regarding the revision. Thank you for the opportunity to consider your work for publication. I look forward to receiving your revised manuscript.

With best regards,

Ieva

Ieva Gailite, PhD
Senior Scientific Editor
The EMBO Journal
Meyerhofstrasse 1
D-69117 Heidelberg
Tel: +4962218891309
i.gailite@embojournal.org

Instructions for preparing your revised manuscript:

Please make sure you upload a letter of response to the referees' comments together with the revised manuscript.

Please also check that the title and abstract of the manuscript are brief, yet explicit, even to non-specialists.

When assembling figures, please refer to our figure preparation guideline in order to ensure proper formatting and readability in print as well as on screen:
https://bit.ly/EMBOPressFigurePreparationGuideline
See also figure legend guidelines: https://www.embopress.org/page/journal/14602075/authorguide#figureformat

At EMBO Press we ask authors to provide source data for the main manuscript figures. Our source data coordinator will contact you to discuss which figure panels we would need source data for and will also provide you with helpful tips on how to upload and organize the files.

IMPORTANT: When you send the revision we will require
- a point-by-point response to the referees' comments, with a detailed description of the changes made (as a word file).
- a word file of the manuscript text.
- individual production quality figure files (one file per figure)
- a complete author checklist, which you can download from our author guidelines (https://www.embopress.org/page/journal/14602075/authorguide).
- Expanded View files (replacing Supplementary Information)
Please see out instructions to authors
https://www.embopress.org/page/journal/14602075/authorguide#expandedview

Please remember: Digital image enhancement is acceptable practice, as long as it accurately represents the original data and conforms to community standards. If a figure has been subjected to significant electronic manipulation, this must be noted in the figure legend or in the 'Materials and Methods' section. The editors reserve the right to request original versions of figures and the original images that were used to assemble the figure.

Further information is available in our Guide For Authors: https://www.embopress.org/page/journal/14602075/authorguide

We realize that it is difficult to revise to a specific deadline. In the interest of protecting the conceptual advance provided by the work, we recommend a revision within 3 months (7th Jun 2023). Please discuss the revision progress ahead of this time with the editor if you require more time to complete the revisions. Use the link below to submit your revision:

Link Not Available

***

Rev_Com_number: RC-2022-01766
New_manu_number: EMBOJ-2023-113761
Corr_author: Zurzolo
Title: A balance between actin and Eps8/IRSp53 utilization in branched versus linear actin networks determines tunneling nanotube formation

Response to reviewers EMBOJ-2023-13761

We would like to thank both reviewers for their general appreciation of our work, for their constructive and relevant comments and for their suggestions that helped us greatly improve our manuscript. We have followed all their suggestions and performed relevant additional experiments wherever feasible, providing new figures and additional data that we believe have clarified the questions raised and further supported our conclusions. Our point-by-point reply for each comment is presented below.

Reviewer 1, Point 1: From Figure 1b - the micro patterned substrates separated by different distances also appear to be printed as different diameters - is this true? I could find no mention of it in the text. My concern is that changing the contact area between the cell and substrate might impact the cell's ability to extend very long membrane protrusions towards its neighbors. Why not print patterns of exactly the same diameter separated by increasing distances?

Reply: We apologize for the confusion. The representative micropatterns in Figure 1B are not of different sizes; the image of the *D*40 substrate is slightly zoomed out as compared to the *D*15 substrate in order to show several patterns within the field of view given the greater 40 µm separation distance. In our study, we kept the micropattern diameter constant at 31 µm and increased the separation distance. This information was originally found in the figure captions for Figure 1 and Figure S1 (now Appendix Figure S1).

To avoid future confusion for readers, we have added small clarifications in the Results section as follows.
Line 156: Thus, to assess TNT formation as a function of intercellular distances, we fabricated hexagonal arrays of circular fibronectin (FN) micropatterns of constant diameter (31 µm) in which the separation distance *D* (Figure 1A and B, Appendix Figure S1A) was tuned to be beyond the reach of filopodia which typically ranges 1–2 µm and rarely exceeds 10 µm (Mattila & Lappalainen, 2008; Mogilner & Rubinstein, 2005).

Reviewer 1, Point 2: By using micropatterned substrates the Authors hope to force the extension of TNTs using actin driven mechanisms, while eliminating the possibility that cells are form these connections by "dislodging" from the substrate. The Authors also state that the cells do not wander into the region in between the circular patterns. However, in many images featured in multiple figures, I can find examples where cells clearly extend into the space between the circular patterns (see Fig. 2.a.i for an example). The primary concern here is that the authors did not fully eliminate the possibility that cells are making direct contact and then pulling away from each other/dislodging to form TNTs. In the supplemental material, there were movies showing TNTs that had already formed, but I was unable to find example of TNTs in the act of forming. If the authors do have timelapse data that clearly shows this, it should be front and center in this data set.

Reply: Cells present in the inter-pattern areas arise as cell clumps in the prepared cell suspension sediment to the surface during plating, leaving cells to straddle between patterns by remaining adhered with their neighbours that are spreading on the patterns. Though our established protocol

(see Materials and Methods) uses divalent ion chelators to reduce intercellular cadherin-cadherin interactions, and 40 μm nylon cell strainers to remove large cellular aggregates prior to cell plating, cell clumping remains an unavoidable problem with CAD cells. Thus, as seen in Figure 2A, Subpanel i as the reviewer noted, some random areas on the surface have large clumps of cells. However, in our TNT counting, we do not consider TNT-connected cells within these regions for our quantification given we cannot fully eliminate the possibility that they formed via dislodgment. In our quantifications, we thus assess the number of TNT connections linking well-patterned cells within a field of view and normalize by the number of nearest-neighbour cell-occupied micropatterns to arrive at the percentage of TNT-connected micropatterns. As shown in Figure EV1A and B (previously Figure S1A and B) where we tracked cell movement, areas where CAD cells were well-patterned showed they remained resident to their pattern and did not translocate to another (only 1 translocation event out of 56 total trajectories was observed). In the revised manuscript, we have added additional text within the Materials and Methods to state that our TNT-quantifications indeed do not include these regions of cell clumping as TNT formation via cell dislodgement cannot be excluded. Additionally, as requested by the reviewer, we have performed new live-cell imaging using F-Tractin expressing cells that clearly show that between well-patterned cells TNT formation indeed occurs through the generation of actin protrusions and not through cell dislodgement; these data can be found in Figure EV1C with accompanying Movies EV5 and EV6.

New additions found within the Results and the Materials and Methods are as follows.

Line 166: Cells rarely exhibited events of inter-micropattern translocation where only 1 event out of 56 was observed (Figure EV1A and B, Example 3 and Movie EV4). Furthermore, live-cell imaging of patterned cells expressing the F-actin reporter F-Tractin showed that TNTs originate from dorsal generated actin-based protrusions that reach a neighbouring cell to form a stable cell-to-cell connection (Figure EV1C and Movies EV5 and EV6).

Line 854: The percent of TNT-connected cells on micropatterns was obtained by counting the TNT-connected cells in areas of robust patterning (areas of cell clumping were avoided as TNT formation by cell dislodgment could not be fully excluded) and dividing by the number of nearest-neighbour cell-occupied micropatterns (i.e., isolated cell-occupied micropatterns were excluded since nearest-neighbour micropatterns are unoccupied and thus incapable of having a TNT spanning between micropatterns).

<u>New Figure EV1C</u>

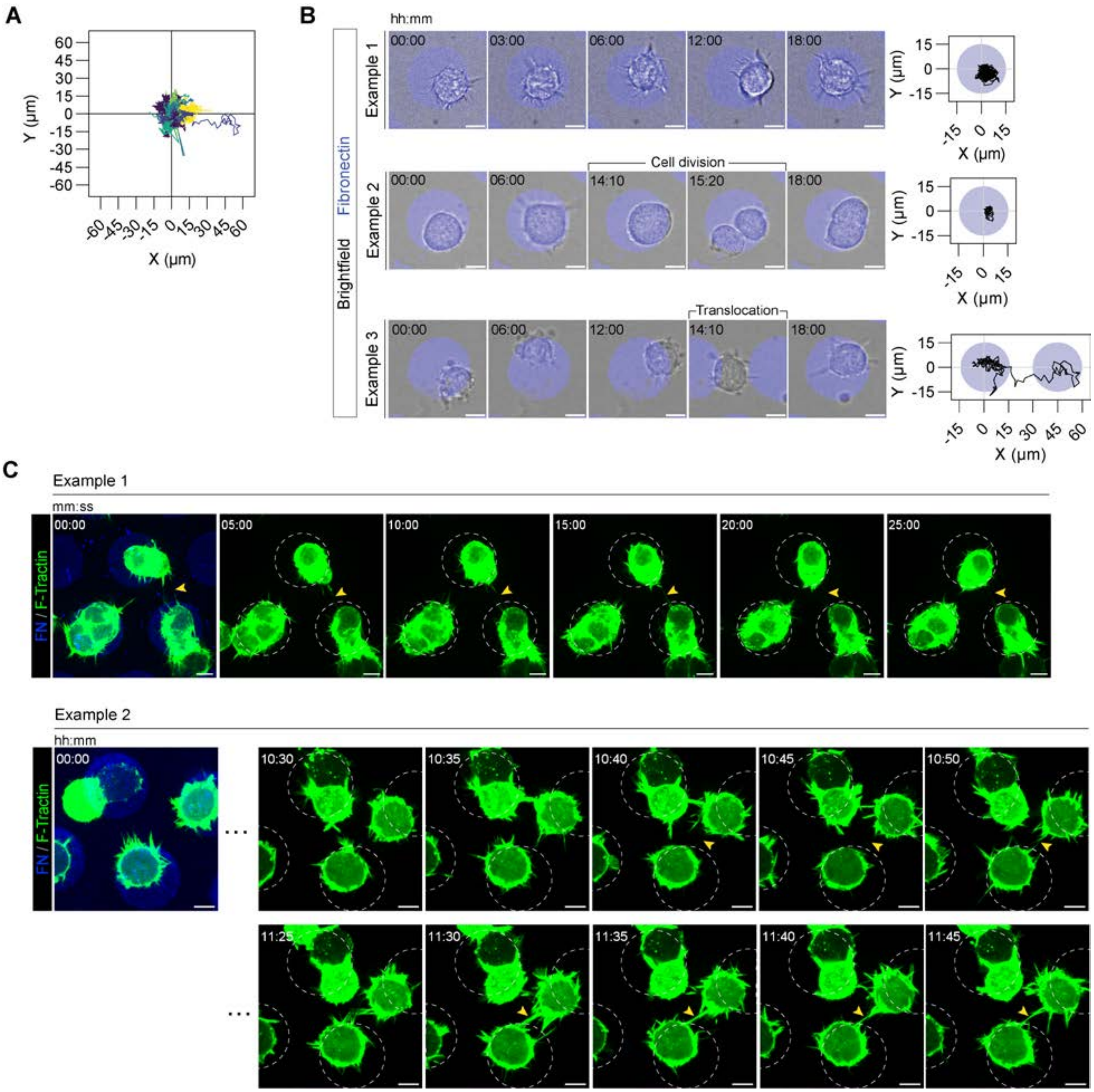

Figure EV1: TNT formation between micropatterned cells does not occur through cell dislodgment.

(**A, B**) Assessment of CAD cell confinement over an extended period of time (18 hr) on *D*15 fibronectin micropatterns. (**A**) Plot of individual cell trajectories normalized with respect to the center of their micropattern (n = 56 trajectories). (**B**) Representative time-lapse images of CAD cells and their corresponding trajectories. Fibronectin micropatterns are false-colored in blue (Rhodamine fibronectin). Scale bars, 10 µm.

(**C**) Selected time frames from a 30 min (Example 1, Movie EV5) and an over-night (Example 2, Movie EV6) acquisition showing TNT-like protrusion formation between *D*15 micropatterned cells (expressing F-Tractin, green) occurs through actin-based protrusions. Images of the F-Tractin channel are max intensity projections of the upper stacks in the acquired Z range, and were

overlayed with the fibronectin channel to highlight cell residency to the micropatterns. White dotted circles annotate the AX-405-labelled fibronectin patterns (blue) shown on the left, and yellow arrowheads point to representative TNTs. Scale bars, 10 µm.

Reviewer 1, Point 3: The authors make use of CK-666 to inhibit Arp2/3, which is thought to free up actin monomers that can be used to further elongate TNTs. Other orthogonal methods should be employed to corroborate the results from CK-666 treatment. There are other drugs that inhibit Arp2/3 (e.g. arpin, wiskostatin) and of course there s always genetic manipulation (shRNA KD).

Reply: We thank the reviewer for bringing to our attention the WASP inhibitor Wiskostatin, however, we had reservations in using this drug, as instead of directly inhibiting Arp2/3 complex activity, it will act upstream to inactivate the Arp2/3 activator WASP and may therefore have unintended, off-target effects in our cells. CK-666 is a well-accepted inhibitor (Nolen *et al*, 2009; Hetrick *et al*, 2013) and has had wide use in research such as in studying microvilli elongation (Faust *et al*, 2019), B cell receptor organization in the immunological synapse (Bolger-Munro *et al*, 2019), and in Arp2/3 activity in growth cone dynamics (Yang *et al*, 2012; Miguel-Ruiz & Letourneau, 2014).

Thus, to further substantiate our CK-666 results, as suggested by the reviewer, we have performed new knockdown (KD) experiments against the Arp2/3 complex protein Actr3 (Arp3) using siRNA. Following KD, we first assessed the impact on TNT-connected cells adhered on micropatterns of increasing separation distances (*D*15 and *D*30) and found that Actr3 KD had an identical effect as our previous CK-666 results in promoting the number of connections. Secondly, we performed co-culture experiments and found that Actr3 KD in donor cells leads to an increase in the percentage of acceptor cells containing donor-derived vesicles, indicating reduced cellular Arp2/3 activity leads to increased functional TNT formation capable of contact-mediated cargo transfer.

Overall, these data corroborate our results in an orthogonal, independent manner as suggested by the reviewer. In the revised manuscript, we have included the new Actr3 KD results and a brief description of the knockdown method as listed below. Additionally, in regards to Figure 2, we have added new supplementary figures in the Appendix that provide an extended gallery of representative images for the CK-666 (Appendix Figure S4) and Actr3 KD (Appendix Figure S5) studies.

Results

Line 210: To further substantiate the negative role that Arp2/3 activity has on TNT formation, we then implemented a knockdown (KD) approach using siRNA to interfere with the expression of the Arp2/3 complex protein Actr3, i.e., Arp3, (an average decrease of 98% compared to Scramble control) in CAD cells (Figure 2D). Quantification of the percent of TNT-connected cells on both *D*15 and *D*30 micropatterns showed a significant increase in TNT numbers for Actr3 KD cells as compared to control cells treated with non-targeting “Scramble” siRNA duplexes (Figure 2E−G, and Appendix Figure S5) and was in agreement with our previous CK-666 results. To examine whether the increase in TNTs upon Actr3 KD is correlated with an increase in functional TNTs capable of cargo transfer, we then performed a vesicle transfer assay (Appendix Figure S2B) in which Scramble- and siActr3-treated donor cells containing DiD-labelled vesicles were co-cultured with H2B-EBFP expressing acceptor cells (Figure 2H and I). In parallel, samples in which

conditioned media from Scramble and siActr3 donor cells were applied to acceptor cells were prepared in order to rule out the contribution of vesicle transfer by secretion and determine the amount of vesicles transferred via cell-cell contact. After 16 to 18 hr of co-culture, Actr3 KD co-cultures showed an increased contact-mediated transfer of vesicles (Figure 2J) that was approximately 25% higher when compared to Scramble controls (Figure 2K), suggesting that a reduction in active Arp2/3 complexes enhances the transfer of vesicles via an increased number of TNT connections between patterned cells.

Materials and Methods

Line 612: ON-TARGETplus siRNA pools against mouse Actr3 (L-046642-01) and IRSp53 (L-046696-01) were purchased from Horizon Discovery, while a non-targeting, universal Scramble siRNA duplex (SR30004) was purchased from Origene. siRNAs were transiently transfected into cells using serum-free Opti-MEM media and Lipofectamine RNAiMAX (Thermo Fisher Scientific) following the manufacture's recommendation. Briefly, CAD cells were plated in 6-well plates to achieve 70% confluency at the time of transfection. A two-day knockdown procedure was performed in which cells on each day were incubated with 30 pmol of siRNA over 6 hr. Cells were used for further experiments at 24 hr following a knockdown procedure and knockdown efficiency was assessed by SDS-PAGE and Western blot at the time of sample fixation (i.e., 48 hr post knockdown completion).

New Figure 2D–K

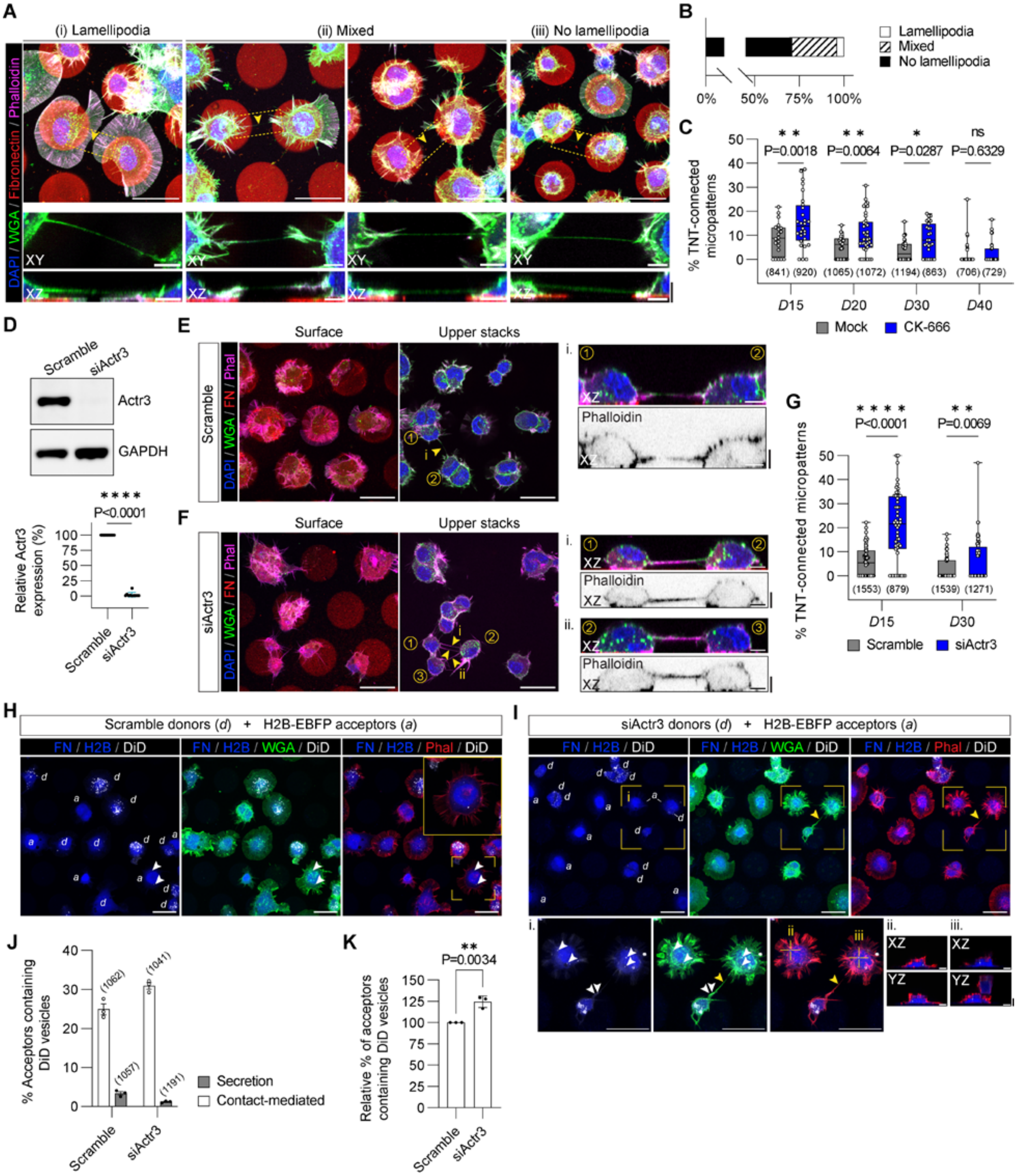

Figure 2: TNT prevalence negatively correlates with Arp2/3 activity.

(**A**) TNT-connected cells characteristically lack lamellipodial features. Representative maximum intensity projection images of cells with different morphologies that are connected by TNTs; Scale bars, 30 µm. TNTs can be formed between: (i) two cells forming only lamellipodia, (ii) two cells showing a mix of phenotypes (e.g., one of the connected cells displays only (or partial)

lamellipodia while the other connected cell shows no lamellipodia), or (iii) two non-lamellipodia cells displaying a higher density of peripheral protrusions. Dashed yellow boxes correspond to subpanels below each image that show expanded views and XZ projections of TNTs that were made through the long axis of the connection; Scale bars, 5 µm. Yellow arrowheads directly point to the TNT.

(**B**) Bar graph showing the characterization of TNTs between cells of different morphologies plated on *D*15 micropatterns (n = 97 TNTs). Lamellipodia, 4.1%; Mixed, 24.7%; and No Lamellipodia, 71.1%.

(**C**) Whisker box plot showing the percentage of TNT-connected micropatterns for *D*15, *D*20, *D*30 and *D*40 micropatterns in cells treated with 50 µM CK-666 as compared to the mock condition (DMSO vehicle control). Mock vs. CK-666 average values were 8.8 ± 1.5% vs. 15.5 ± 2.0% for *D*15 micropatterns, 5.0 ± 0.8% vs. 10.4 ± 1.3% for *D*20 micropatterns, 3.6 ± 0.7% vs. 8.5 ± 1.3% for *D*30 micropatterns, and 2.0 ± 0.9% vs. 2.3 ± 0.8% for *D*40 micropatterns (mean ± SEM). Each data point corresponds to a quantified image in which on average approximately 30 cell-occupied micropatterns were within the acquired field of view. Representative images of the different conditions can be found in Appendix Figure S4. The total number of individual micropatterns quantified in each condition is indicated below each box. Data was pooled from 3 experiments, and was analysed using an unpaired Mann-Whitney test.

(**D**) siRNA knockdown of Actr3 (Arp3) in CAD cells. Top: Representative Western blot of Scramble control cells and siActr3 cells revealed with α-Actr3 and α-GAPDH (loading control) antibodies. Bottom: Graph showing the relative expression of Actr3 in Scramble control cells (set to 100%) and siActr3 cells (1.9 ± 1.2%, mean ± SEM). Statistical analysis was performed using an unpaired Student's t-test (n = 10).

(**E**) Representative surface and upper stack maximum intensity projections of Scramble control CAD cells plated on *D*15 micropattern; Scale bars, 30 µm. Subpanels in i show XZ projections made through the long axis of the indicated TNT (yellow arrowhead); Scale bars, 5 µm. Cells were fixed and stained with DAPI (blue), AX-488 WGA (green) and AX-647 Phalloidin (magenta); micropatterns were visualized using Rhodamine FN (red).

(**F**) Representative surface and upper stack maximum intensity projections of siActr3 treated CAD cells plated on *D*15 micropatterns; Scale bars, 30 µm. Subpanels (i, ii) show XZ projections made through the long axis of the indicated TNTs (yellow arrowheads); Scale bars, 5 µm. Cells were fixed and stained with DAPI (blue), AX-488 WGA (green) and AX-647 Phalloidin (magenta); micropatterns were visualized using Rhodamine-FN (red).

(**G**) Whisker box plot showing the percentage of TNT-connected micropatterns for Scramble control and siActr3 CAD cells plated on *D*15 and *D*30 micropatterns. Scramble vs. siActr3 average values were 5.9 ± 0.7% vs. 20.5 ± 1.9% for *D*15 micropatterns, and 2.6 ± 0.6% vs. 6.3 ± 1.1% for *D*30 micropatterns (mean ± SEM). Each data point corresponds to a quantified image in which on average approximately 20 cell-occupied micropatterns were within the acquired field of view. Representative images of the different conditions can be found in Appendix Figure S5. The total number of individual micropatterns quantified in each condition is indicated below each box. Data for *D*15 and *D*30 was pooled from 4 and 3 experiments, respectively, and was analysed using an unpaired Mann-Whitney test.

(**H**) Representative images of Scramble-treated donor cells (*d*) co-cultured with H2B-EBFP expressing acceptor cells (*a*) on *D*15 micropatterns; Scale bars, 30 µm. White arrowheads annotate donor-originating DiD-labeled vesicles (white) in an acceptor cell. The yellow box indicates the enlarged area shown in the upper right inset. Cells were fixed and stained with AX-488 WGA (green) and Rhodamine Phalloidin (red); micropatterns were visualized using AX-405 FN (blue).

(**I**) Representative images of siActr3-treated donor cells (*d*) co-cultured with H2B-EBFP expressing acceptor cells (*a*) on *D*15 micropatterns; Scale bars, 30 µm. Subpanels in i show an enlargement of the enclosed area noted by the yellow box in the main micrographs; Scale bars, 30 µm. White arrowheads annotate donor-originating DiD-labelled vesicles (white) in an acceptor cell; yellow arrowheads annotate TNTs connecting cells on neighbouring micropatterns. Indicated crosshairs (ii, iii) correspond to XZ and YZ orthogonal projections of internalized vesicles in the subpanels; Scale bars, 5 µm. Cells were fixed and stained with AX-488 WGA (green) and Rhodamine Phalloidin (red); micropatterns were visualized using AX-405 FN (blue).

(**J**) Bar graph showing the percentage of H2B-EBFP acceptors containing DiD-labelled vesicles received through cell-cell contact (i.e., corrected for secretion-based transfer) from Scramble- (24.9 ± 1.4%) and siActr3-treated (30.9 ± 0.9%) donors. Corresponding secretion-based transfer levels used for determining contact-mediated transfer levels are shown for comparison, and were 3.3 ± 0.4% and 1.1 ± 0.1% for Scramble and siActr3, respectively. The total number of quantified acceptor cells is indicated for each condition. Data are from 3 individual experiments and are represented as a mean ± SEM.

(**K**) Bar graph showing the relative percentage of H2B-EBFP acceptors containing DiD-labelled vesicles from siActr3-treated donors (124.5 ± 3.9%, mean ± SEM) as compared to Scramble control donors (set to 100%). Data are from 3 individual experiments and were analysed using an unpaired Student's t-test.

<u>**New Appendix Figure S4**</u>

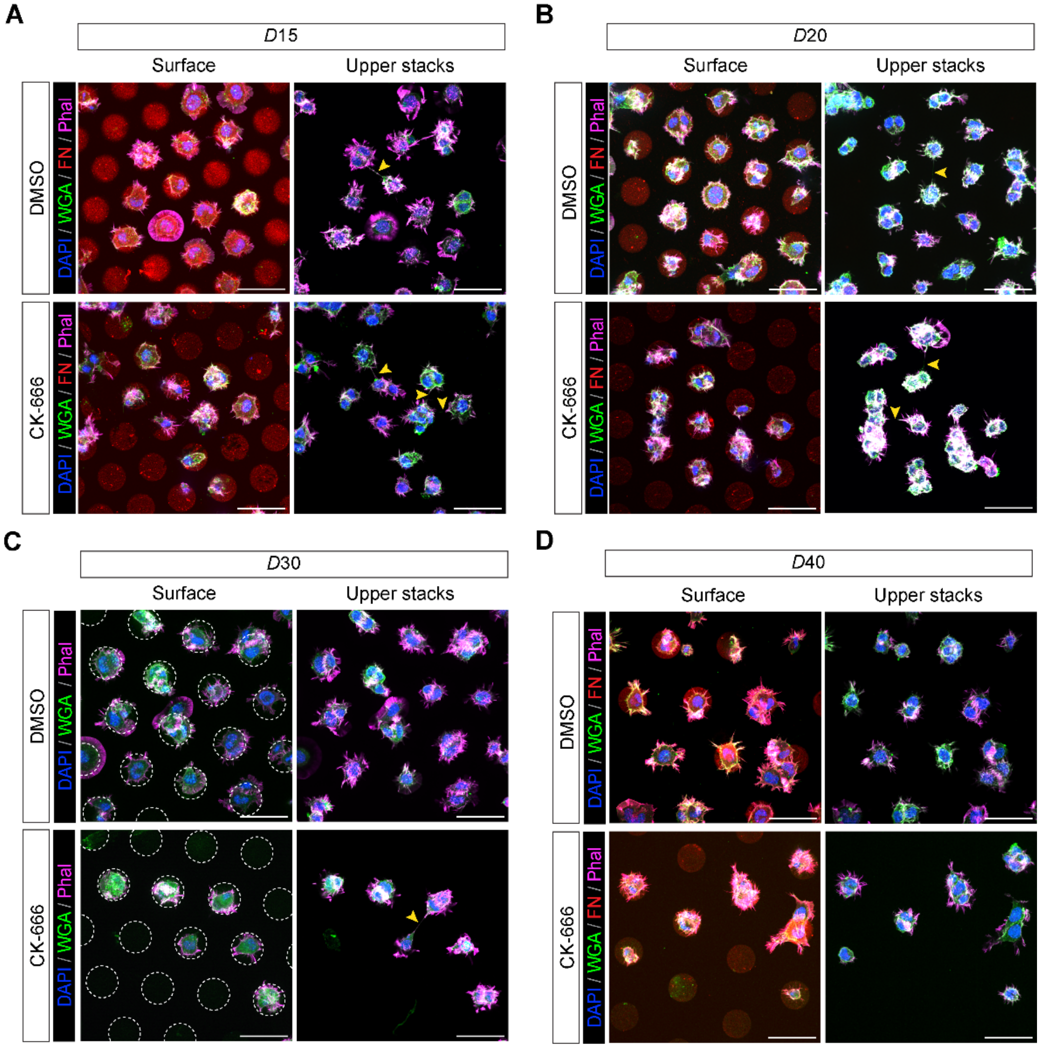

Appendix Figure S4: Gallery of representative images for DMSO- and CK-666-treated CAD cells adhered on micropatterns of increasing separation distances.

(**A**–**D**) Surface and upper stack maximum intensity projections of CAD cells plated on *D*15 (**A**), *D*20 (**B**), *D*30 (**C**) and *D*40 (**D**) fibronectin (FN) micropatterns. Upper panels show DMSO-treated cells while lower panels show CK-666-treated (50 µM) cells. Scale bars, 50 µm. TNTs connecting cells on two different micropatterns are annotated with yellow arrowheads. Cells were fixed and stained with DAPI (blue), AX-488 WGA (green) and AX-647 Phalloidin (magenta); micropatterns were generally visualized using Rhodamine FN (red). When non-fluorescent fibronectin was used, micropatterns were visible upon over-saturation in the green channel and outlined with dotted white circles for clarity.

__**New Appendix Figure S5**__

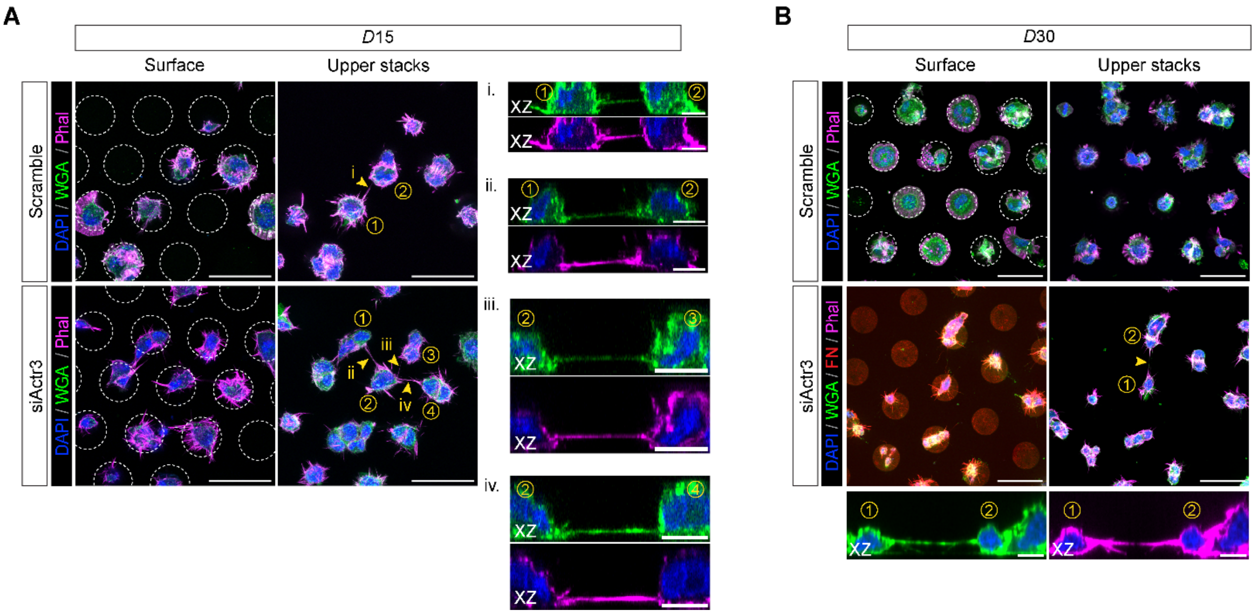

Appendix Figure S5: Gallery of representative images of Scramble control and Actr3 knockdown CAD cells adhered on micropatterns of increasing separation distances.

(**A**, **B**) Surface and upper stack maximum intensity projections of Scramble control and siActr3 CAD cells plated on *D*15 (**A**) and *D*30 (**B**) fibronectin (FN) micropattern. Scale bars, 50 µm. Subpanels (i–iv) show XZ projections made through the long axis of the indicated TNTs (yellow arrowheads); Scale bars, 10 µm. Cells were fixed and stained with DAPI (blue), AX-488 WGA (green) and AX-647 Phalloidin (magenta); micropatterns were visualized using Rhodamine FN (red). When non-fluorescent fibronectin was used, micropatterns were visible upon over-saturation in the green channel and outlined with dotted white circles for clarity.

__Reviewer 1, Point 4__: The authors also employ a formin agonist, IMM-01, and the results from those experiments (Fig. S4) suggest that activating formin mDia could facilitate TNT elongation, but the authors do not follow up with direct molecular manipulation to further test this idea. Are formins (specifically mDia, the target of IMM-01) needed to elongate TNTs?

__Reply__: The reviewer raises an important point. As a first approach, we have screened several formins and assessed their effect on the number of TNT-connected cells through overexpression experiments using cells plated on non-micropatterned Ibidis. While mDia1 and FMNL2 appear to be the most promising candidates as there was a non-significant trend to increase the percentage of TNT-connected cells (**Fig. R1a, b**), we found that overexpressing formins in general was difficult due to poor transfection efficiency (likely given the large sizes of these proteins), and when successful in a few cells, the overexpression often leads to clear changes in the morphologies of our CAD cells or differentiation into neurons that made interpretation of the data complicated.

For example, while mDia2-WT led to an increase in substrate-attached and dorsal filopodia formation with a clear localization of the protein to the tip of these protrusions (**Fig. R1c, top**), no long TNTs were observed in these conditions. This may be a result of mDia2 over-promoting protrusion numbers that are smaller in length, thus limiting TNT formation based on our results with Arp2/3 inhibition showing that G-actin availability is limiting within the cell. Moreover, the overexpression of active mDia2 lacking its DAD domain (mDia2-dDAD) showed a prominent, intracellular vesicular pattern that led to a general loss of protrusions (both filopodia and TNTs) while appearing to promote actin cable assembly (**Fig. R1c, bottom**). Similarly, overexpressing mDia3-WT showed the protein associated with vesicular compartments within the perinuclear region leading to a reduction in TNT numbers (**Fig. R1d**). This overexpression pattern of mDia3 in CAD cells resembles (and even more when mDia3 was co-transfected with active RhoD, data not shown) to the one observed in HeLa cells where RhoD, through hDia2C (or mDia3), was found to regulate endosome dynamic (Gasman *et al*, 2003). As for FMNL3, the WT form and FH1-FH2 domain-only form (i.e., active FMNL3) both led to a strong decrease in the percent of TNT-connected cells (**Fig. R1e**). Closer examination of the FH1-FH2 cells revealed cells exhibited a flatter, more adherent phenotype with prominent stress fibers that coincided with a lack of filopodia, implicating a role of FMNL3 in F-actin rearrangement in CAD cells that does not promote TNT formation.

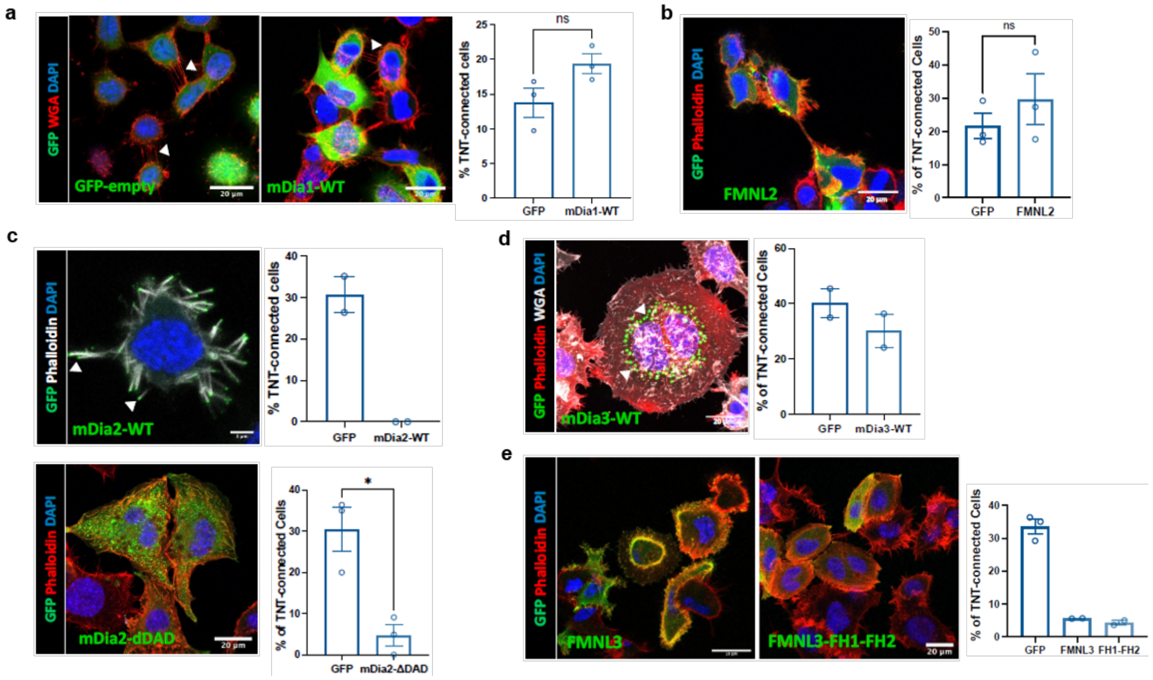

Figure R1: Screen of formins in TNT formation. (a) *Left: Representative images of the upper stacks of control GFP-empty vector expressing cells and GFP-mDia1-WT expressing cells. White arrowheads point to TNTs. Right: Bar graph (n = 3 experiments) showing the quantification of TNT-connected cells in GFP control (13.8 ± 2.1%) and in GFP-mDia1-WT (19.4 ± 1.4%). Total cells analyzed: GFP ctrl, 367; GFP-mDia1-WT, 396. Data are represented as a mean ± SEM. Statistical analysis was performed using a t-test with Welch's correction, p = 0.1048.* ***(b)*** *Left: Representative image of the upper stacks of FMNL2-GFP expressing cells. Right: Bar graph (n = 3 experiments) showing the quantification of TNT-connected cells in GFP ctrl (21.6 ± 3.8%) and*

in FMNL2-GFP (29.6 ± 7.6%). Total cells analyzed: GFP ctrl, 335; FMNL2-GFP, 223. Data are represented as mean ± SEM. Statistical analysis was performed using a t-test with Welch's correction, p = 0.4223. ***(c)*** *Top: Representative images of the upper stacks of GFP-mDia2-WT expressing cells, along with a bar graph (n = 2 experiments) showing the quantification of TNT-connected cells in GFP ctrl (30.6 ± 4.4%) and in GFP-mDia2-WT (0.0 ± 0.0%). Total cells analyzed: GFP ctrl, 59; GFP-mDia2-WT, 40. Data are represented as mean ± SEM. White arrowheads indicate the mDia2 formin at the tip of filopodia. Bottom: Representative images of the maximum stack projection of GFP-mDia2-dDAD (i.e., activated mDia2) expressing cells, along with a bar graph (n = 3 experiments) showing the quantification of TNT-connected cells in GFP ctrl (30.5 ± 5.2%) and in mDia2-dDAD (4.7 ± 2.6%). Total cells analyzed: GFP ctrl, 144; GFP-mDia2-dDAD, 140. Data are represented as a mean ± SEM. Statistical analysis was performed using a t-test with Welch's correction, p = 0.0227.* ***(d)*** *Left: Representative image of the maximum stack projection of GFP-mDia3-WT expressing cells. White arrowheads indicate the accumulation of mDia3 in vesicular compartments. Right: Bar graph (n = 2 experiments) showing the quantification of TNT-connected cells in GFP ctrl (40.2 ± 5.2%) and in GFP-mDia3-WT (30.2 ± 6.1%). Total cells analyzed: GFP ctrl, 95; GFP-mDia3-WT, 102. Data are represented as a mean ± SEM.* ***(e)*** *Left: Representative images of the maximum stack projection of FMNL3-GFP and GFP-FMNL3-FH1-FH2 (i.e., an activated FMNL3 construct containing only the FH1 and FH2 domains) expressing cells. Right: Bar graph from at least two experiments showing the quantification of TNT-connected cells in GFP ctrl (33.5 ± 2.2%), FMNL3-GFP (5.6 ± 0.05%) and in GFP-FMNL3-FH1-FH2 (4.3 ± 0.7%). Total cells analyzed: GFP ctrl, 146; FMNL3-GFP, 71; GFP-FMNL3-FH1-FH2, 95. Data are represented as a mean ± SEM.*

At this juncture, we unfortunately cannot definitively conclude for the reviewer that formins are or are not needed for elongating TNTs given the expression difficulties we experienced with these proteins. The IMM-01-induced TNT increase we observed could be resultant from mDia1 and FMNL2 activity as, even with the poor expression, the transfection of these two proteins resulted in a trend to increase TNT-connected cells. We have added the data for the reviewer but we believe that the data will be too confusing and preliminary to add them in the manuscript. However, we have added the following sentences in the Discussion beginning at **Line 520**:

"**Our results using a formin agonist support our conclusions that the promotion of linear actin networks over branched directly favours functional TNT formation (Appendix Figure S6). It also suggests that activating diaphanous-related formins (i.e., those such as mDia and FMNL proteins that harbour auto-inhibitory interactions between their C-terminal diaphanous autoregulatory domain (DAD) with an N-terminal diaphanous inhibitory domain (DID)) could facilitate TNT elongation. However, in our preliminary experiments, the effect of formin expression on TNTs in neuronal CAD cells was confounded by a strong effect on neuronal morphology which impaired the recognition of TNTs in the absence of a specific marker (data not shown). Thus, the direct involvement of formins in TNT elongation needs to be further explored.**"

Reviewer 1, Point 5: With the experiments shown in Figure 3, the authors apply optical tweezers to pull what they refer to as "nanotubes" from the cell surface under conditions where Arp2/3 is inhibited with CK-666. The panels appear to show that CK-666 allows actin to assemble out into a pulled nanotube more readily than control cells. However, the reporter for actin assembly in these experiments is F-Tractin, and it only partially fills the protrusion. I note here that F-Tractin appears more soluble under the CK-666 condition. Can the Authors rule out the possibility that there is just a larger soluble pool of F-Tractin probe under these conditions?

Reply: F-Tractin is a well-established reporter for F-actin in living cells and we choose to use it given it more accurately reproduces the visualization of phalloidin-stained F-actin structures in cells unlike Lifeact or Utrophin reporters (Belin *et al*, 2014). The reviewer raises the concern that CK-666 treatment appears to solubilize F-Tractin, creating a ctyoplasmic pool of F-Tractin that then determines the fluorescent profile measured within the pulled nanotube, however, we can rule out this diffusion-dependent effect and confirm F-Tractin is accurately reflecting F-actin development within the nanotubes. First, as seen in **Fig. R2**, cells with different F-Tractin levels within their cell body exhibited identical profiles within the pulled nanotubes that reached an endpoint at 10 μm within the nanotube (see Example 1 and Example 2). If the profiles within these tubes were completely dependent on a cytosolic pool of unbound F-Tractin diffusing into the nanotube, then the length scale of the profile would therefore be set by the bulk concentration of soluble F-Tractin via Fick's Diffusion Law. Thus, cells with more soluble F-Tractin would exhibit longer profiles and vice versa for cells with lower amounts. However, we clearly observe identical endpoints of the F-Tractin fluorescence within the nanotube for cells of differing cellular F-Tractin levels with only a difference in the absolute intensities between the profiles, thus ruling out such a diffusion-mediated effect. Furthermore, if a diffusion-mediated effect predominated, then cells with comparable F-Tractin levels should always exhibit identical endpoints in their profiles. However, as seen when comparing Example 1 with Example 3, different endpoints were observed supporting the conclusion that F-actin grew to different lengths in these cases and that F-Tractin is a reporter of the extent of F-actin growth within the nanotube, but does not influence it. Secondly, we have provided **new Kymograph analysis** in Figure 3B–E of the revised manuscript showing F-Tractin dynamics that clearly arise from the presence of F-actin within the nanotube (e.g., stationary, non-growth phases that transition to a phase of growth within the nanotube) that would otherwise be absent in a diffusion-mediated case where F-Tractin would quickly establish a stable profile that does not vary through time.

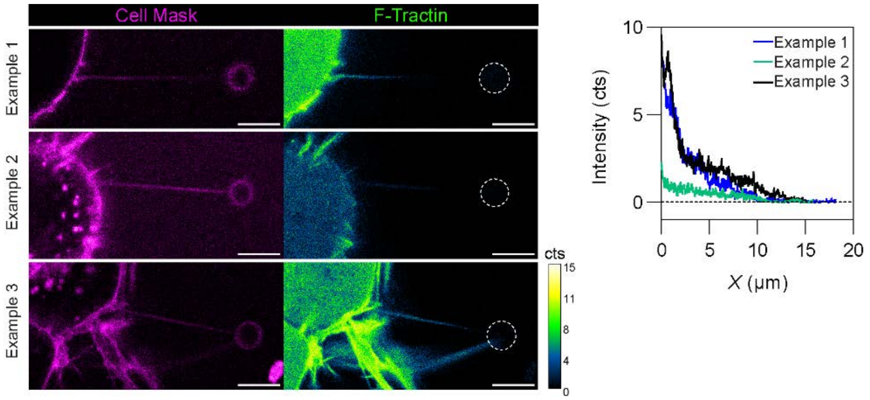

Figure R2: Cellular F-Tractin level does not establish the resulting fluorescent profile measured in pulled nanotubes. *(Left) Representative confocal images of nanotubes pulled form CK-666-treated CAD cells having different cellular F-Tractin intensity levels. Images in the F-Tractin channel were identically scaled between 0 and 15 photon counts (cts) for visual comparison. (Right) Plot of the intensity profiles within the respective pulled nanotubes measured along the length of the nanotube (X) from its base to its end attached to the trapped bead. Scale bars, 5 µm.*

In the revised manuscript, we have included our new kymograph analysis as follows.

Results

Line 252: In mock, DMSO-treated conditions, cells displayed little to no F-actin growth within the nanotubes (Figure 3B and Movie EV7) while CK-666-treated cells showed instead a robust development of F-actin (Figure 3C and Movie EV8) that emanated from the base of the nanotube and grew towards the nanotube end (see comparative kymograph analysis in Figure 3D and E).

New Figure 3B–E

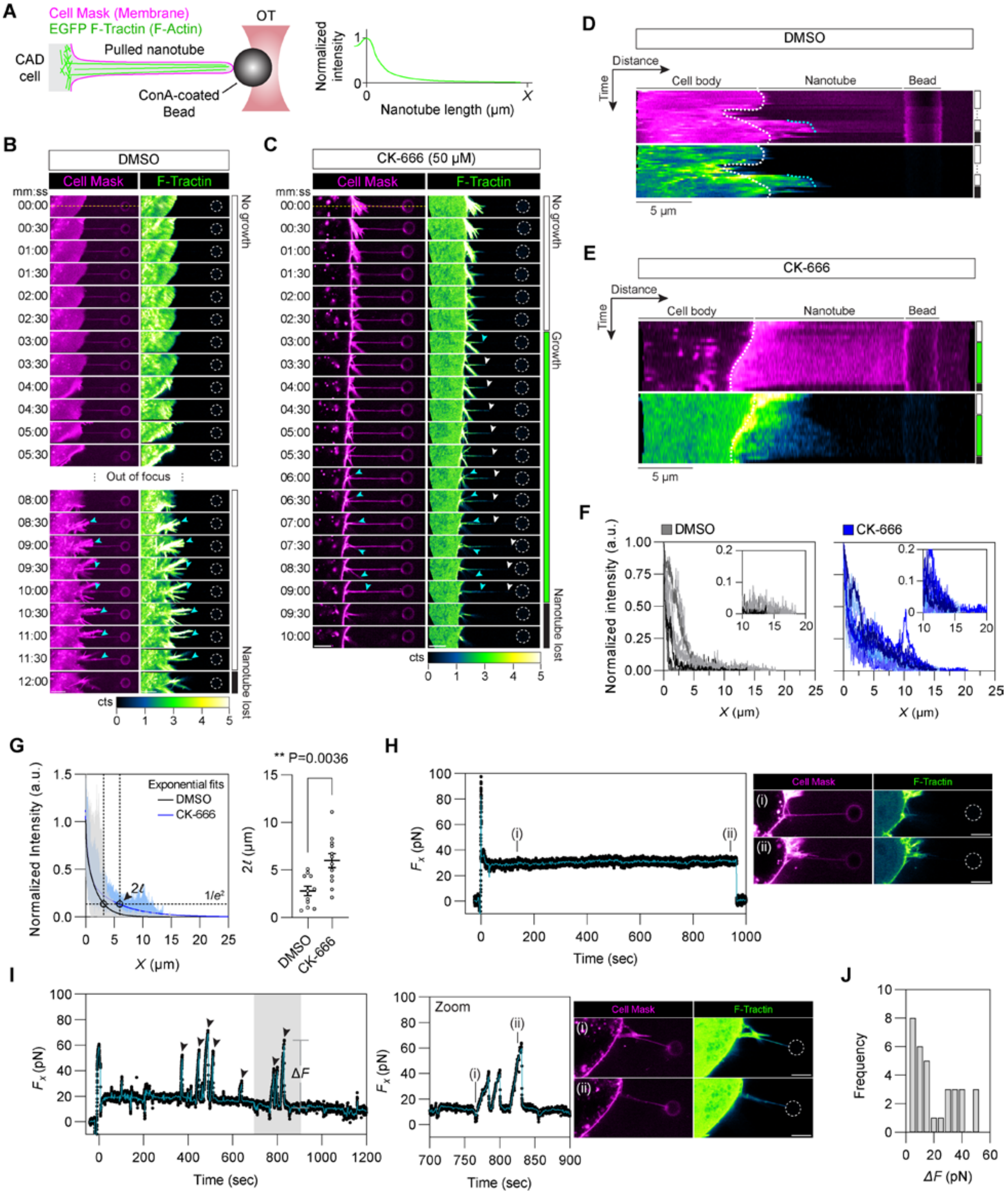

Figure 3: Inhibition of branched actin formation results in greater F-actin development in pulled nanotubes.

(**A**) Experimental setup for pulling membrane nanotubes using a concanavalin A (Con-A)-coated bead (~3 μm in diameter) trapped in an optical tweezer (OT). CAD cells stably expressing the

small F-actin-binding peptide F-Tractin fused to EGFP (green) were exogenously labelled with the lipophilic Cell Mask^{TM} Deep Red plasma membrane stain (magenta). Intensity profiles of the F-actin fluorescence were measured along the nanotube axis and plotted against the nanotube length. Pulled nanotube lengths averaged 14.2 ± 0.6 µm (mean ± SEM, n = 23 tubes).

(**B, C**) Time-lapse montages of nanotubes pulled from a DMSO-treated cell (Movie EV7) and a CK-666-treated cell (Movie EV8). Displayed intensities for the F-Tractin channel were set between 0 and 5 photon counts (cts) for better visualization. White arrowheads annotate the progression of actin development within the nanotube, while cyan arrowheads annotate obstructing protrusions that grew along the pulled nanotube. The trapped bead is annotated with a dotted white circle when not clearly visible. Scale bars, 5 µm.

(**D, E**) Corresponding kymographs for the time-lapse montages in B and C, generated along the nanotube axis (yellow dotted lines in B and C). Dotted white and cyan lines in the kymographs mark the cell body edge and obstructing protrusions, respectively. Scale bars, 5 µm.

(**F**) Plot of actin profiles within pulled nanotubes for mock- and CK-666-treated cells. Insets show a magnified view at the tube extremity to better highlight the greater presence of F-actin in the CK-666 condition as compared to the mock condition. Mock condition, 11 tubes; CK-666 condition, 12 tubes.

(**G**) Left: Exponential fits to the actin intensity profiles were performed to determine a characteristic decay length (2l) at which the initial intensity at $X = 0$ decays to a value of $1/e^2$. For visualization purposes of the analysis, exponential fits are shown for the mean actin profiles computed from the individual plots presented in **F**. Upper and lower limits of the intensity range are shaded. Right: Dot plot of the characteristic decay lengths (2l) for mock- and CK-666-treated cells. Data is represented as the mean ± SEM. Mock (11 tubes), 2.78 ± 0.50; CK-666 (12 tubes), 5.80 ± 0.73. Statistical analysis was performed using an unpaired Mann-Whitney test.

(**H**) Left: Force plot of a pulled nanotube from a mock-treated cell showing no F-actin development. Solid teal line, 10-point moving average curve. Right: Associated images of the indicated time points (i, ii). The trapped bead is annotated with a dotted white circle when not clearly visible. Scale bars, 5 µm.

(**I**) Left: Force plot of a pulled nanotube from a CK-666-treated cell showing F-actin development spanning the entire nanotube length. Peaks in the force plot (black arrowheads), with magnitudes of ΔF, arise when retrograde flows outcompete actin polymerization (at the nanotube tip) causing bead displacement towards the cell body (recorded as a positive rise in the force in the lab frame). Solid teal line, 10-point moving average curve. Shaded grey region corresponds to a magnified view in the centre. Right: Associated images of the indicated time points (i, ii). The trapped bead is annotated with a dotted white circle when not clearly visible. Scale bars, 5 µm.

(**J**) Histogram of the force peak magnitudes (ΔF). Sample size, 33 peaks.

Reviewer 1, Point 6: Related to the previous point, it is not clear to me why the Authors need to invoke the application of external forces with an optical trap to study the enhanced elongation of TNTs under CK-666 conditions. Why don't the authors directly visualize actin accumulation and TNT elongation after treatment with this inhibitor?

Reply: We use our tube pulling experiment to create a structure that resembles a protrusion to independently measure actin growth in a protrusion-like structure. In "real" protrusions, the length of the protrusion is a direct read-out of the actin filament length, but their growth can be perturbed by other parameters, notably, membrane tension that can restrain actin growth. Thus, this nanotube pulling assay permits a controlled and high-throughput means for directly measuring the true extent Arp2/3 inhibition can shift the cellular system towards favouring longer filament formation. With this assay we were able to clearly characterize a difference in the most probable F-actin length scales achievable in control vs. CK-666-treated conditions (Figure 3). In control conditions, F-actin length on average did not extend more than 3–5 µm within the nanotubes which nicely corresponded to the filopodia lengths we measured for CAD cells (Figure 1G). Contrastingly, Arp2/3 inhibition showed the mean F-actin length was instead shifted to far longer distances. As part of our revision, we have added new data from nanotubes pulled to extreme lengths (Figure EV2); these data support our earlier conclusions and clearly demonstrates that Arp2/3 inhibition increases the frequency with which actin filaments are able to polymerize to lengths beyond those obtainable by typical filopodia. Moreover, we believe OT-pulled nanotubes are a good model system to study TNTs as we were able to recapitulate the recruitment of both Eps8 and IRSp53 in actin-positive nanotubes (see new Appendix Figure S9) that mirrored our new IF data presented in Appendix Figure S7 showing the endogenous presence of Eps8 and IRSp53 in TNTs (in response to Point 8 below).

In the revised manuscript, we have updated the text within the Results to better emphasize our rationale for using this optical tweezer approach, expanded the explanation of the results presented in Figure 3 based on our new kymograph analysis, and add new text in regards to Figure EV2.

Results

Line 243: Given these pulled nanotubes are initially devoid of any actin, filament growth within the nanotube can be monitored by confocal microscopy (Figure 3A) (Tsai *et al*, 2022; Datar *et al*, 2015; Bornschlögl *et al*, 2013; Pontes *et al*, 2011). While the measured length of any cellular protrusion is a direct readout of the overall actin filament length within, our assay instead advantageously permits us to ascertain the most probable length scales actin reaches in a given condition as filament growth can occur in a situation unencumbered by restraining factors, such as the tension of the membrane, that "real" protrusions must work against to outwardly grow. Thus, our assay permits a controlled and high-throughput means for assessing the true extent to which Arp2/3 inhibition may favour longer filament formation necessary for achieving the lengths exhibited by TNTs.

Line 252: In mock, DMSO-treated conditions, cells displayed little to no F-actin growth within the nanotubes (Figure 3B and Movie EV7) while CK-666-treated cells showed instead a robust development of F-actin (Figure 3C and Movie EV8) that emanated from the base of the nanotube and grew towards the nanotube end (see comparative kymograph analysis in Figure 3D and E). Measurement of the F-actin fluorescence profiles showed a rapid decay of the signal in mock-treated cells (Figure 3F) where intensity values were indistinguishable from background levels

beyond 5 µm, a length scale consistent with the average length measured previously for CAD cell filopodia (Figure 1G).

Line 269: These data indicate that an inhibition of Arp2/3 activity directly correlates with a greater occurrence of actin filaments able to polymerize and reach longer distances. To further substantiate these results, we pulled nanotubes at more extreme lengths averaging 33.6 ± 1.2 µm (mean ± SEM, 42 tubes) (Figure EV2) to assess the most probable lengths that F-actin is able to achieve upon Arp2/3 inhibition. As before, we found that cells in the mock, control condition exhibited little infiltration of F-actin growth within the nanotube (Figure EV2A and B); F-actin profiles typically decayed to intensity values indistinguishable from background levels within 5 µm inside the nanotube and did not extend beyond 10 µm (Figure EV2E). Contrastingly, Arp2/3 inhibition led to far more F-actin growth (Figure EV2C and D) with a noticeable increase in the percentage of pulled tubes exhibiting F-actin infiltration beyond 10 µm with even events of F-actin growth reaching distances upwards of 20 µm (Figure EV2E). These data indicate that an inhibition of Arp2/3 activity directly correlates with the polymerization of longer actin filaments, permitting TNTs to more frequently reach extreme distances (i.e., their distance dependency is shifted to longer length scales).

<u>**New Figure EV2**</u>

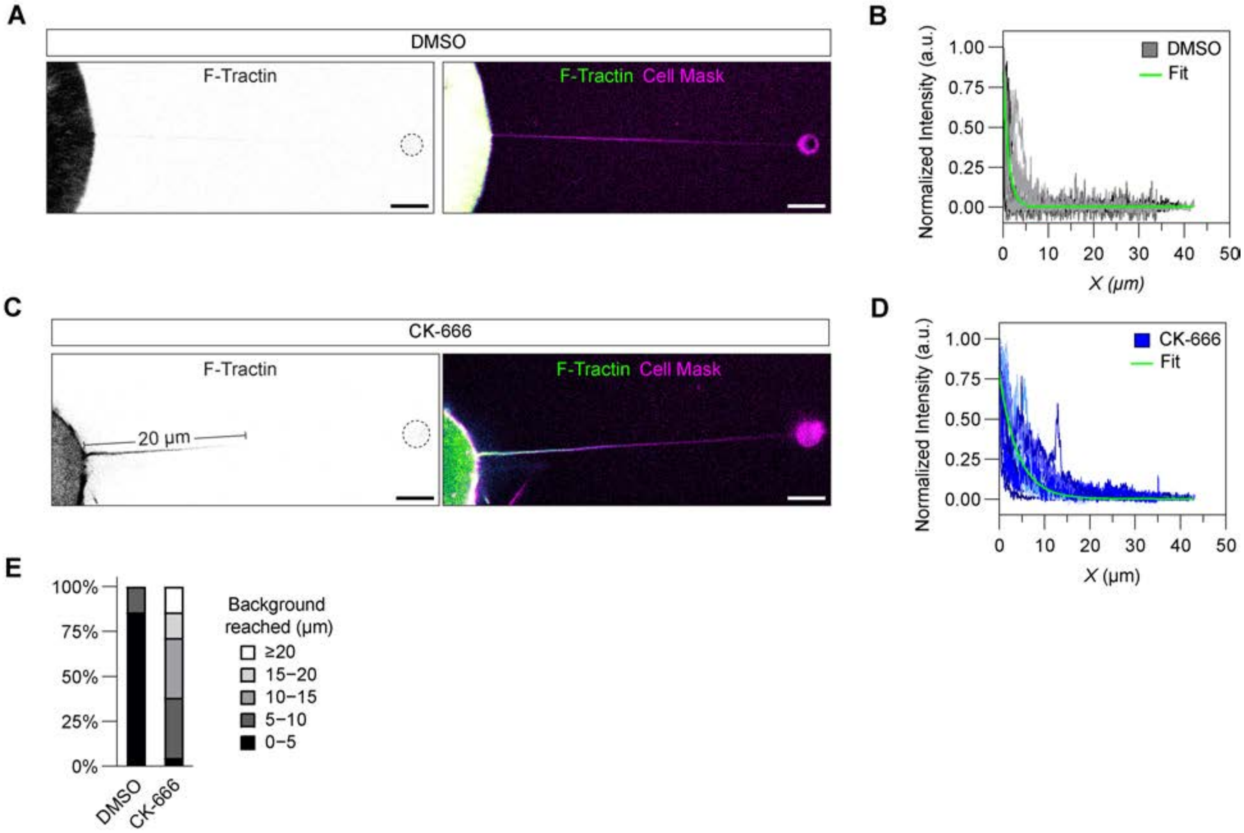

Figure EV2: Inhibition of Arp2/3 activity increases the frequency of actin polymerization occurring at extreme distances.

(**A**) Representative nanotube pulled to an extreme length from a DMSO-treated CAD cell showing little to no actin polymerization within. Displayed F-Tractin intensities were set between 0 and 10 photon counts for better visualization. The trapped bead is annotated with a dotted white circle when not clearly visible. Scale bars, 5 µm.

(**B**) Plot of actin profiles within pulled nanotubes of extreme lengths for DMSO-treated CAD cells (n = 21 tubes). The solid green line is an exponential fit to the combined data.

(**C**) Representative nanotube pulled to an extreme length from a CK-666-treated (50 µM) CAD cell showing actin growth 20 µm within the nanotube. Displayed F-Tractin intensities were set between 0 and 10 photon counts for better visualization. The trapped bead is annotated with a dotted white circle when not clearly visible. Scale bars, 5 µm.

(**D**) Plot of actin profiles within pulled nanotubes of extreme lengths for CK-666-treated CAD cells (n = 21 tubes). The solid green line is an exponential fit to the combined data.

(**E**) Categorization of the percentage of pulled nanotubes with F-actin profiles becoming indistinguishable from background intensity levels within a given micron range for DMSO- and CK-666-treated CAD cells.

Reviewer 1, Point 7: In Figure 4, the Authors move away from TNTs and instead focus on "longer protrusions" (filopodia?) to examine the effects of EPS8 and IRSp53 on growth of these structures. The general findings here are consistent with the role of these factors in protrusion growth from previous studies.

Reply: We agree with the reviewer that the general findings here are consistent with the role of these factors in protrusion growth from previous studies, as we also discuss in our discussion. Because of this, we felt it was pertinent to do a comparison in our specific cell line, even if similar experiments have been done in other cells before, on the separate overexpression of IRSp53 and Eps8. Given our live imaging was done on isolated cells to observe the possible extent to which such IRSp53- and Eps8-driven protrusions could reach at any given moment, we could not call them TNTs as no "acceptor" cell was nearby for the protrusion to connect with. However, our results presented in Figure 4 do provide new information in regards to how both IRSp53 and Eps8 must be released from their involvement in branched actin networks in order to drive actin elongation that is able to reach TNT length scales, a result that is consistent with our proteomics analysis. Additionally, the endogenous presence of IRSp53 and Eps8 in TNTs determined by immunofluorescence (Appendix Figure S7) as well as our new data showing the recruitment of both IRSp53 and Eps8 in pulled nanotubes (Appendix Figures S9) further supports the role of these actors in TNT formation.

Reviewer 1, Point 8: The experiments in Figure 5 are performed with a truncated "bundling active" form of EPS8 allegedly lacking actin capping activity. The logic behind this choice is unclear. These experiments need to be repeated in parallel with full length WT EPS8 to allow for a full and clear interpretation of these results. Along these lines, could the Authors stain for endogenous Esp8/IRSp53 complex within TNT-connected cells?

Reply: We apologize to the reviewer for a minor misunderstanding, but the Eps8-ΔCAP (bundling active) mutant is not a truncated version. In actuality, the mutant was made through single-point

mutations within Eps8's C-terminal region (V689D, L693D) to impair its actin capping ability. While we did point readers to the original reference from the G. Scita lab for further details into its construction (Hertzog *et al*, 2010), to avoid further confusion on this matter, we have added an additional explanatory phrase in the Materials and Methods of the revised manuscript as follows.

Line 571: GFP-Eps8-WT and the capping-deficient mutant GFP-Eps8-ΔCAP (V689D and L693D point mutations within the C-terminal region) were a kind gift from Giorgio Scita (Istituto FIRC di Oncologia Molecolare, Italy) and have been described elsewhere (Hertzog *et al*, 2010).

Our rationale for using Eps8-ΔCAP is that protrusions in general require bundled actin filaments that can collectively generate enough localized force to overcome the resistance imposed by the tension of the plasma membrane to grow outwards effectively (Mogilner & Rubinstein, 2005). Otherwise, poor or inefficient bundling of the core filaments within a nascent protrusion would buckle under the strain of the membrane tension and no outward growth would happen. Moreover, our past study showed Eps8-ΔCAP had a pronounced effect in promoting not only the percentage of TNT-connected cells, but also the number of vesicles transferred in a donor-acceptor co-culture assay (Delage *et al*, 2016). Thus, we respectively disagree with the reviewer that re-preforming all experiments in Figure 5 with Eps8-WT would provide a clearer interpretation of the results. First, we know that using Eps8 in its bundling active version does not impair any interaction with IRSp53 (**Fig. R3**). Secondly, such experiments would not add to the interpretation of Eps8's role in TNT formation but complicate it as the expression of Eps8 WT would create a scenario in which the protein could act predominately in its capping function, reducing F-actin elongation and hence limit overall protrusion formation.

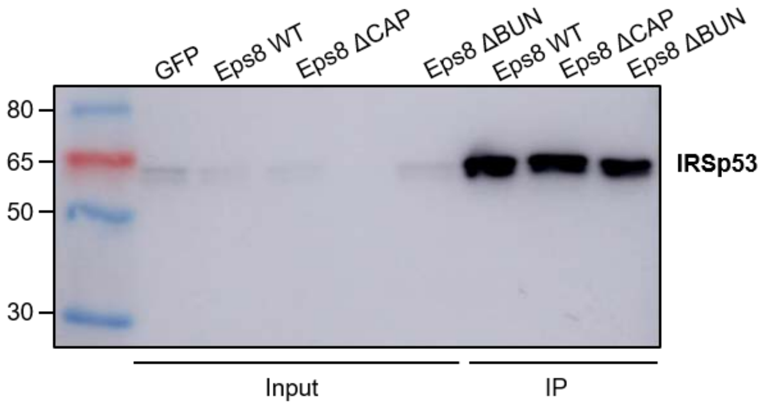

Figure R3: Eps8 interacts with IRSp53 irrespective if its bundling or capping active form. *Representative Western Blot showing GFP-trap immunoprecipitation bands of the pray protein IRSp53 in the Eps8 WT, Eps8 ΔCAP and Eps8 ΔBUN bait precipitates (right) and input lysates (left).*

To better emphasize our logic for the use of Eps8-ΔCAP, we have added new text within the Results as follows.

<u>Results</u>

Line 286: …we investigated whether a specific Eps8-I-BAR interaction favours long TNT formation. Such an interaction would couple Eps8's ability to bundle F-actin necessary to overcome the tension imposed by the membrane, and an I-BAR's ability to induce membrane deformation through the protein's intrinsic curvature.

Line 306: In these experiments we utilized Eps8 in its bundling active form (capping defunct mutant; Eps8-ΔCAP) to thus observe the impact of this function on protrusion formation without secondary effects caused by its capping function.

Additionally, as suggested by the reviewer, we have provided new immunofluorescence data in the Results showing the endogenous presence of both Eps8 and IRSp53 in TNTs.

Line 297: Furthermore, immunofluorescence showed both endogenous Eps8 and IRSp53 were detected in TNT-like structures (Appendix Figure S7).

New Appendix Figure S7

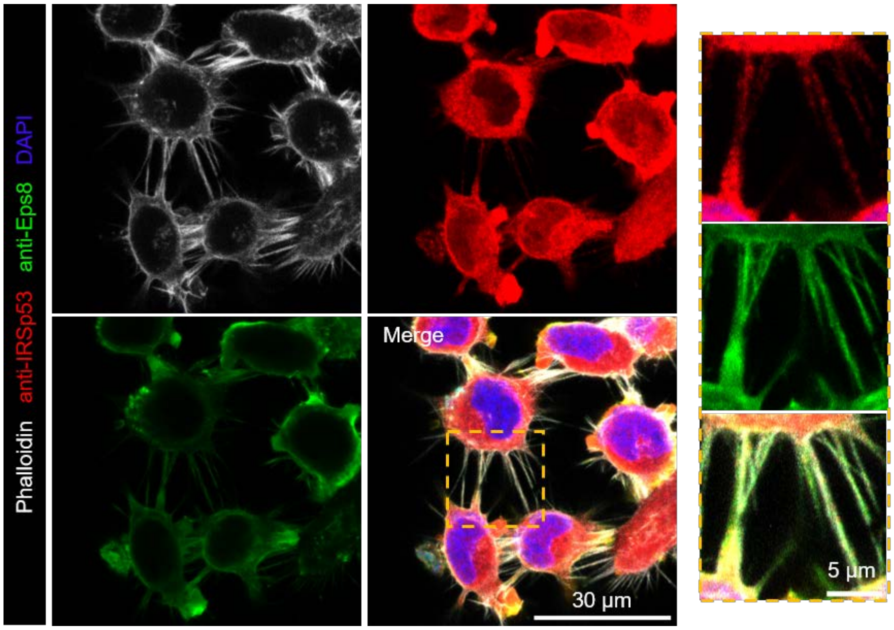

Appendix Figure S7: Immunofluorescence reveals Eps8 and IRSp53 are endogenously present in TNTs.
Representative confocal images of the upper stacks of CAD cells connected by TNTs positive for endogenous Eps8 and IRSp53. CAD cells were plated on non-micropatterned Ibidi dishes the day before sample processing. Cells were fixed in 4% PFA, permeabilized with 0.05% Saponin, and blocked in 2% BSA. Primaries: 1:100 anti-Eps8 (Mouse IgG_1, BD Biosciences, 610143); 1:500 anti-IRSp53 (Rabbit polyclonal, Atlas, HPA023310). Dashed yellow box correspond to zoom-in subpanels on the right.

Reviewer 1, Point 9: Data in Figure 5 show that the Eps8dCAP/IRSp53 complex increases the vesicle transfer within TNT-connected cells from Eps8dCAP/IRSp53 donors to EBFP-

H2B acceptors indicating that functionality of TNT. Interestingly, cells with overexpression of Eps8dCAP/IRSp53 and CK-666 treatment do not increase TNT-connections. Could the authors examine the stability of TNTs under this condition? The lack of increase vesicle transfer may be due to the instability of the TNT structure rather the saturation of the system.

Reply: It is not clear why CK-666 addition to cells expressing Eps8:IRSp53 would lead to greater TNT instability as the reviewer suggests given the co-expression of both proteins promotes more TNTs (Figure 5). Furthermore, we have performed new siRNA knockdown experiments against IRSp53 in the revised manuscript and have shown through both TNT counting and vesicle transfer assays that IRSp53 is a necessary component in forming functional TNTs (Figure 6). Nevertheless, we agree with this reviewer that assessing the TNT stability (i.e., duration) in our experimental condition is feasible and could lead to further understanding of the mechanism leading to TNT formation. Thus, to explore the reviewer's hypothesis, we have performed over 6000 min of new live imaging to measure the duration of TNTs in these different conditions of co-expression and drug treatment and have included these results in the revised manuscript as a new figure (Figure EV4). Here we first cloned in-house a lentiviral construct having an iRFP-tagged version of Eps8-ΔCAP so that Eps8 could be stably expressed simultaneously with IRSp53-mCherry and the F-actin reporter F-Tractin (to visualize TNTs). Using these newly constructed cell lines, we used spinning disc microscopy to image (every 15 sec for up to 300 min) pre-existing TNTs linking Eps8-ΔCAP:IRSp53 cells and determine the time it takes the TNT to break (i.e., a proxy for its lifetime) in conditions of either DMSO or CK-666 treatment, as compared to TNT-connected cells solely expressing F-Tractin (i.e., control cells). The data shows that CK-666 treatment had no significant effect on increasing TNT duration as compared to DMSO-treated control cells (Ctrl + DMSO vs. Ctrl + CK-666), as the median TNT duration was 33.50 min and 36.13 min respectively for DMSO- and CK-666 treated control cells. This result signifies that the dominant effect of CK-666 treatment is its promotion of TNT numbers through their increased ability to reach the distant-patterned cells, and is in agreement with our early data in Figures 2 and 3. However, of interest, upon expressing Eps8-ΔCAP:IRSp53, the median TNT duration was significantly increased from 33.50 min (Ctrl + DMSO) to 66.50 min (Eps8-ΔCAP:IRSp53 + DMSO), and was not further increased upon CK-666 treatment (Eps8-ΔCAP:IRSp53 + CK-666) as the median TNT duration was merely 64.75 min. Together, these data show that the saturation effect observed in Figure 5B and D for TNT counting and vesicle transfer upon Eps8:IRSp53 co-expression arises from an increased stability of TNTs that is directly dependent on the presence of Eps8 and IRSp53. We therefore thank the reviewer for pushing us to perform these arduous but informative experiments.

In the revised manuscript, we have included our new IRSp53 siRNA and TNT duration data as listed below. Additionally, a brief description of the cloning strategy used in the generation of the novel pCDH1-iRFP670-Eps8-ΔCAP construct along with a new section on the Assessment of TNT Duration can be found in the Materials and Methods.

Line 332: To perturb the interaction, we performed siRNA silencing against IRSp53 achieving an 89% decrease in protein expression levels (Figure 6A). As compared to Scramble control cells, IRSp53 silenced cells plated on *D*15 micropatterns showed a near abrogation in the percent of TNT-connected cells (Figure 6B–D). We then examined whether such a sharp decrease in TNTs upon IRSp53 KD is correlated with a similar reduction in TNT functionality. Scramble- and IRSp53 siRNA-treated donor cells containing DiD-labelled vesicles were co-cultured with H2B-EBFP expressing acceptor cells (Figure 6E and F) and assayed in parallel against secretion-based

controls to determine the portion of transfer mediated by cell-cell contact. Following overnight co-culture, IRSp53 KD co-cultures showed a marked decrease in contact-mediated transfer of vesicles (Figure 6G), reflecting a relative decrease of 58% (Figure 6H).

Line 356: To test this, we performed live imaging of pre-existing TNTs linking micropatterned Eps8-ΔCAP:IRSp53 cells expressing F-Tractin and measured the duration of the TNT to the point of its breakage (i.e., a proxy for its lifetime) in conditions of either DMSO or CK-666 treatment as compared to TNT-connected cells solely expressing F-Tractin (control cells) (Figure EV4A–D). CK-666 treatment had no significant effect on increasing TNT duration as compared to DMSO-treated control cells (Figure EV4E, Ctrl + DMSO vs. Ctrl + CK-666), signifying the dominant effect of CK-666 treatment is its promotion of TNT numbers not their stability. However, upon expressing Eps8-ΔCAP:IRSp53, the median TNT duration was significantly increased from 33.50 min (Figure EV4E, Ctrl + DMSO) to 66.50 min (Figure EV4E, Eps8-ΔCAP:IRSp53 + DMSO), and was not further increased with CK-666 treatment (Figure EV4E, Eps8-ΔCAP:IRSp53 + CK-666). These data support that the saturation effect observed in Figure 5B and D arises from an increased TNT stability afforded by Eps8 and IRSp53.

Line 595: An iRFP670 tagged version of Eps8-ΔCAP was generated using the aforementioned pCDH1-GFP-Eps8-ΔCAP construct as a backbone template and a vector containing the iRFP670 sequence of interest (generous gift from Mathieu Coppey, Institut Curie). Briefly, the pCDH1-GFP-Eps8-ΔCAP construct was cut at EcoRI and XhoI sites to excise the GFP sequence. Following PCR amplification and agarose gel extraction of the iRFP670 amplicon, the amplicon was ligated into the cut backbone using the In-Fusion HD Cloning Plus kit (Takara Bio) according to the manufacturer's recommendations. Primers used in the cloning strategy were: iRFP forward (5'-CTAGAGCTAGCGAATTCGCCACCATGGTAGCAGGTCATGCCTCTGGCAG-3′); iRFP reverse (5'-GAGCGGTTAGACATATGACCATTCATCTCGAGCCCACTCCCGCTTCCGCTC TCAAGCGCGGTGATCCGCGTC-3′

Line 933: Assessment of TNT Duration. CAD cells expressing EGFP F-Tractin alone, or co-expressing EGFP F-Tractin, IRSp53-mCherry and iRFP670-Eps8-ΔCAP were plated and cultured on *D*15 micropatterns (generally labelled with AX-405 fibronectin) minimally for 4 hr and up to 16–18 hr overnight before acquisition. One hour prior to the start of acquisition, samples were mounted on a Chemlide CMB 35 mm magnetic chamber (Quorum Technologies) and treated with 50 µM CK-666 or an equivalent amount of DMSO solubilized in phenol-free complete Opti-MEM medium containing ProLong^{TM} Live Antifade Reagent (Invitrogen) at a 1:75 dilution. Time-lapse imaging of an existing TNT connecting well-patterned CAD cells was performed on an Eclipse Ti2 inverted microscope (Nikon) equipped with a CSU-W1 spinning disk confocal scanning unit (Yokogawa), a 60X 1.4 NA oil immersion objective (Nikon Plan Apo VC), a fibre-coupled Coherent OBIS laser illumination system (405/488/561/640 nm), and a Prime 95B sCMOS camera (Teledyne Photometrics). Acquisition was controlled by Nikon's NIS-Elements software, and a humidified 37°C and a 5% CO_2 environment was maintained in an OkoLab enclosure. Z-stack slices (0.5 µm thickness) encompassing the TNT and covering the cell volume were acquired at a 15-sec interval until the TNT broke or until the maximum allotted observational time was reached (300 min) if the TNT remained attached and unbroken. After the initial time point in which reference images for the micropatterns, Eps8 and IRSp53 were acquired, the remaining time points continued only with F-Tractin acquisitions to prevent photo-induced cellular toxicity.

New Figure 6

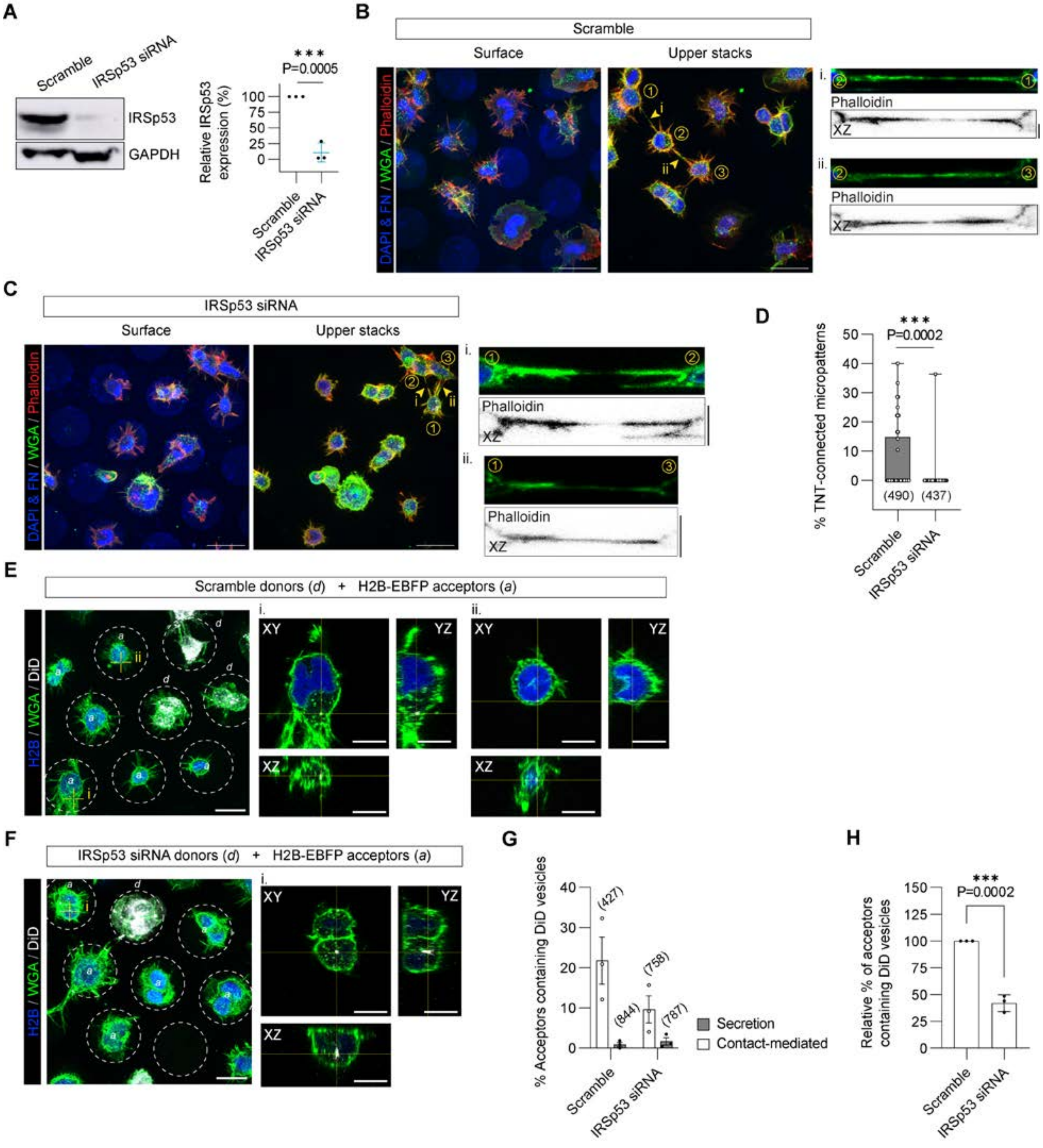

Figure 6: IRSp53 knockdown leads to a decrease in functional TNTs.

(**A**) siRNA knockdown of IRsp53 in CAD cells. Left: Representative Western blot of Scramble control cells and IRSp53 siRNA cells revealed with α-IRSp53 and α-GAPDH (loading control) antibodies. Right: Graph showing the relative expression of IRSp53 in Scramble control cells (set

to 100%) and IRSp53 siRNA cells (10.8 ± 8.7%, mean ± SEM). Statistical analysis was performed using an unpaired Student's t-test (n = 3).

(**B**) Representative surface and upper stack images of control CAD cells plated on *D*15 micropatterns treated with Scramble siRNA; Scale bars, 30 µm. Subpanels (i, ii) show XZ projections made through the long axis of the indicated TNTs (yellow arrowheads); Scale bars, 5 µm. Cells were fixed and stained with DAPI (blue), AX-488 WGA (green) and Rhodamine Phalloidin (red); micropatterns were visualized using AX-405 FN (blue).

(**C**) Representative surface and upper stack images of IRSp53 knock-down CAD cells plated on *D*15 micropatterns treated with a pool of IRSp53 siRNA; Scale bars, 30 µm. Subpanels (i, ii) show XZ projections made through the long axis of the indicated TNTs (yellow arrowheads); Scale bars, 10 µm. Cells were fixed and stained with DAPI (blue), AX-488 WGA (green) and Rhodamine Phalloidin (red); micropatterns were visualized using AX-405 FN (blue).

(**D**) Whisker box plot showing the percentage of TNT-connected micropatterns for Scramble control and IRSp53 siRNA CAD cells plated on *D*15 micropatterns. Scramble vs. IRSp53 siRNA average values were 6.8 ± 0.8% vs. 0.7 ± 0.7% (mean ± SEM). Each data point corresponds to a quantified image in which on average approximately 10 cell-occupied micropatterns were within the acquired field of view. The total number of individual micropatterns quantified in each condition is indicated below each box. Data was pooled from 3 experiments and was analyzed using an unpaired Mann-Whitney test.

(**E**) Representative images of Scramble-treated donor cells (*d*) co-cultured with H2B-EBFP (blue) expressing acceptor cells (*a*) on *D*15 micropatterns; Scale bars, 20 µm. Indicated crosshairs (i, ii) correspond to XZ and YZ orthogonal projections of DiD-labeled vesicles (white) internalized within acceptor cells (see subpanels); Scale bars, 10 µm. Cells were fixed and stained with AX-488 WGA (green); micropatterns were unstained but visible upon over-saturation in the green channel and outlined with dotted white circles for clarity.

(**F**) Representative images of IRSp53 siRNA-treated donor cells (*d*) co-cultured with H2B-EBFP (blue) expressing acceptor cells (*a*) on *D*15 micropatterns; Scale bars, 20 µm. The indicated crosshair (i) corresponds to XZ and YZ orthogonal projections of an example DiD-labeled vesicle (white) internalized within an acceptor cell (see subpanels); Scale bars, 10 µm. Cells were fixed and stained with AX-488 WGA (green); micropatterns were unstained but visible upon over-saturation in the green channel and outlined with dotted white circles for clarity.

(**G**) Bar graph showing the percentage of H2B-EBFP acceptors containing DiD-labelled vesicles received through cell-cell contact (i.e., corrected for secretion-based transfer) from Scramble- (21.8± 5.8%) and IRSp53 siRNA-treated (9.7 ± 3.4%) donors. Corresponding secretion-based transfer levels used for determining contact-mediated transfer levels are shown for comparison, and were 0.9 ± 0.5% and 1.7 ± 0.9% for Scramble and siActr3, respectively. The total number of quantified acceptor cells is indicated for each condition. Data are from 3 individual experiments and are represented as a mean ± SEM.

(**H**) Bar graph showing the relative percentage of H2B-EBFP acceptors containing DiD-labelled vesicles from IRSp53 siRNA-treated donors (42.0 ± 4.5%, mean ± SEM) as compared to Scramble control donors (set to 100%). Data are from 3 individual experiments and were analyzed using an unpaired Student's t-test.

New Figure EV4

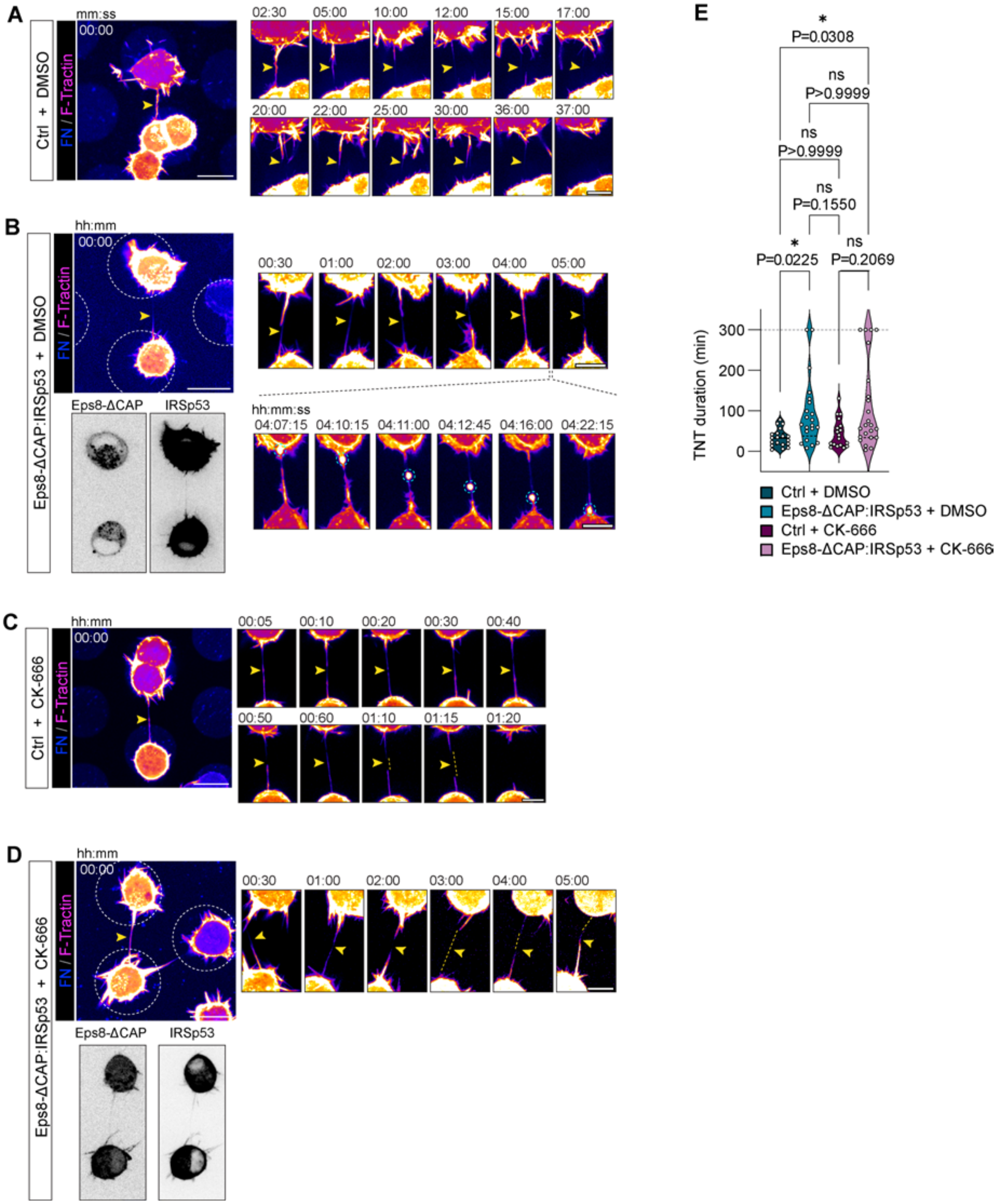

Figure EV4: The co-expression of Eps8 and IRSp53 alone increases the stability of TNTs.

(**A**) Selected time frames from Movie EV15 showing the duration of a TNT connecting EGFP F-Tractin expressing control cells treated with DMSO. Displayed images of the F-Tractin channel

(false coloured in the “Fire” lookup table) are max intensity projections of the upper stacks in the acquired Z range. At the initial time point (left large panel), the *D*15 micropatterns (AX-405 FN, blue) visualized at the sample surface are overlayed for reference. Yellow arrowheads point to the TNT throughout its lifetime. Scale bars, 20 µm (large panel) and 10 µm (subpanels).

(**B**) Selected time frames from Movie EV16 and Movie EV17 showing the duration of a TNT connecting i670-Eps8-ΔCAP, IRSp53-mCherry, and EGFP F-Tractin expressing CAD cells treated with DMSO. Displayed images of the F-Tractin channel (false coloured in the “Fire” lookup table) are max intensity projections of the upper stacks in the acquired Z range. At the initial time point, the *D*15 micropatterns (AX-405 FN, blue) visualized at the sample surface are overlayed with the F-Tractin channel and outlined with white dotted circles for reference (top large panel); additionally, images confirming Eps8 and IRSp53 expression are presented (bottom panels with inverted grayscales). Yellow arrowheads point to the TNT throughout its lifetime. Cyan dotted circles annotate an observed transfer event (see Movie EV17). Scale bars, 20 µm (large panel) and 10 µm (subpanels).

(**C**) Selected time frames from Movie EV18 showing the duration of a TNT connecting EGFP F-Tractin expressing control cells treated with 50 µM CK-666. Displayed images of the F-Tractin channel (false coloured in the “Fire” lookup table) are max intensity projections of the upper stacks in the acquired Z range. At the initial time point (left large panel), the *D*15 micropatterns (AX-405 FN, blue) visualized at the sample surface are overlayed for reference. Yellow arrowheads point to the TNT throughout its lifetime. Yellow dotted lines annotate sections of the TNT with weak F-Tractin fluorescence. Scale bars, 20 µm (large panel) and 10 µm (subpanels).

(**D**) Selected time frames from Movie EV19 showing the duration of a TNT connecting i670-Eps8-ΔCAP, IRSp53-mCherry, and EGFP F-Tractin expressing CAD cells treated with 50 µM CK-666. Displayed images of the F-Tractin channel (false coloured in the “Fire” lookup table) are max intensity projections of the upper stacks in the acquired Z range. At the initial time point, the *D*15 micropatterns (AX-405 FN, blue) visualized at the sample surface are overlayed with the F-Tractin channel and outlined with white dotted circles for reference (top large panel); additionally, images confirming Eps8 and IRSp53 expression are presented (bottom panels with inverted grayscales). Yellow arrowheads point to the TNT throughout its lifetime. Yellow dotted lines annotate sections of the TNT with weak F-Tractin fluorescence. Scale bars, 20 µm (large panel) and 10 µm (subpanels).

(**E**) Violin plot of TNT durations for control cells (those only expressing EGFP F-Tractin) and cells additionally co-expressing i670-Eps8-ΔCAP and IRSp53-mCherry mock treated with DMSO or treated with 50 µM CK-666. Median TNT durations were: Ctrl + DMSO, 33.50 min (n = 19); Eps8-ΔCAP:IRSp53 + DMSO, 66.50 min (n = 21); Ctrl + CK-666, 36.13 min (n = 18); and Eps8-ΔCAP:IRSp53 + CK-666, 64.75 min (n = 23). The black dotted line marks TNTs remaining up until the maximum allotted observational time. Statistical analysis was performed using a Kruskal Wallis test with Dunn’s multiple comparison test. Adjusted P values for each comparison are provided on the plot.

Reviewer 1, Point 10: Figure 5c. The data show that vesicle transfer from EpsdCAP/IRSp53 donors to EBFP-H2B acceptors increases under the overexpression of EpsdCAP/IRSp53. Are the EBFP-H2B acceptors able to form TNT? If so, could the authors show the vesicle transfer in those TNT-connected cells? The images indicate that there is no presence of TNT structures in these conditions.

Reply: Thanks, we realized we were missing this figure. In Figure 5C (CK-666 condition) of the revised manuscript, we have provided a new image to better highlight an Eps8-ΔCAP:IRSp53 donor cell that is linked to an acceptor having received DiD-labelled vesicles.

Reviewer 2, Point 1: Introduction: Please define how filopodia are distinguished from TNTs - is it length only or are there other characteristics? Do filopodia and individual TNTs have the same diameter? There is presumably a functional difference as well?

Reply: We thank the reviewer for the suggestion. We have updated the introduction to have an expanded description on the similarities and differences between filopodia and TNTs, and the implications these have on their differing functionality. Similar to filopodia, TNTs are thin structures having diameters frequently in the range of 100 to 300 nm (Sartori-Rupp et al, 2019). The widths of both of these protrusions are determined by the number of actin filaments residing in the core of the protrusion which average between 10−30 filaments in filopodia, for example. In contrast to filopodia— which harbor adhesion molecules such as integrins that permit their interaction with the extracellular matrix and enable cell adhesion and migration (Galbraith *et al*, 2007; Partridge & Marcantonio, 2006)—TNTs are non-surface adherent structures that cytoplasmically connect distant cells for the transfer of cargo of various sizes, encompassing not only small ions, but also larger organelles. Our recent cryo-EM work (Sartori-Rupp et al, 2019) supports that vesicular cargo is transported in TNTs while such cargo is absent in CAD cell filopodia, mirroring other past reports that show no vesicles nor other organelles within filopodia arising from Dictyostelium (Medalia et al, 2007), HeLa (Mageswaran et al, 2021), HUVEC (Almagro et al, 2010) and NG108-15 cells (Aramaki *et al*, 2016). Thus, a key distinguishing characteristic of TNTs is their functional ability to transfer cargo that is afforded by their open-endedness.

In the revised manuscript, we have updated the text within the Introduction and provided new references as follows:

Line 68: One mechanism relies on the generation of filamentous actin (F-actin)-based protrusions in which the plasma membrane (PM) envelopes a core of 10–30 bundled, parallel-oriented filaments that emanate outwards from the cell periphery (Mattila & Lappalainen, 2008; Blake & Gallop, 2023). Typically projecting only a few microns (<5 μm) from the cell, filopodia are thin (diameters of the order of several hundred nanometres), finger-like structures that dynamically probe the environment through repeated cycles of growth and retraction, establishing focal adhesions through activated integrins at their tip (Blake & Gallop, 2023) or cell-cell connections important for collective migration (Bischoff *et al*, 2021), tissue closure (Jacinto *et al*, 2000), and neurogenesis (Mancinelli *et al*, 2021). While exhibiting comparable widths to filopodia, tunneling nanotubes (TNTs) contrastingly are non-surface adherent extensions and are instead able to reach significantly longer distances (tens of microns), critical in establishing more stable and longer-lived means for direct communication between distant cells (Ljubojevic *et al*, 2021). Though both protrusions are supported by F-actin, recent cryogenic electron microscopy (cryo-EM) work shows TNTs are structurally different from filopodia, revealing TNTs have more continuous and linearly organized filaments as compared to filopodia (Sartori-Rupp *et al*, 2019).

Line 101: Thus, a key distinguishing characteristic of TNTs is their unique functional ability to transfer cargo, a property absent for canonical filopodia as previous ultrastructural studies have found no vesicles nor other organelles within them (Medalia *et al*, 2007; Almagro *et al*, 2010; Aramki *et al*, 2016; Mageswaran *et al*, 2021).

New References

Almagro S, Durmort C, Chervin-Pétinot A, Heyraud S, Dubois M, Lambert O, Maillefaud C, Hewat E, Schaal JP, Huber P, *et al* (2010) The Motor Protein Myosin-X Transports VE-

Cadherin along Filopodia To Allow the Formation of Early Endothelial Cell-Cell Contacts. *Mol Cell Biol* 30: 1703–1717

Aramaki S, Mayanagi K, Jin M, Aoyama K & Yasunaga T (2016) Filopodia formation by crosslinking of F-actin with fascin in two different binding manners. *Cytoskeleton* 73: 365–374

Blake TCA & Gallop JL (2023) Filopodia In Vitro and In Vivo. *Annu Rev Cell Dev Biol* 39: 7.1-7.23

Mageswaran SK, Yang WY, Chakrabarty Y, Oikonomou CM & Jensen GJ (2021) A cryo–electron tomography workflow reveals protrusion-mediated shedding on injured plasma membrane. *Sci Adv* 7: eabc6345

Medalia O, Beck M, Ecke M, Weber I, Neujahr R, Baumeister W & Gerisch G (2007) Organization of Actin Networks in Intact Filopodia. *Curr Biol* 17: 79–84

Reviewer 2, Point 2: Please state the number of cells in each array spot?

Reply: We are a bit unsure what the reviewer was specifically asking here, but if the reviewer was referring to the approximate number of cell-occupied micropatterns associated with each datapoint in a given TNT quantification plot (e.g., Figures 1F and 5B), this information is provided within the respective figure captions and stated as an average number of cell-occupied micropatterns within the acquired field of view. If, however, the reviewer was referring to the average number of CAD cells per fibronectin patch, then this data can be found in Appendix Fig. S1B. As part of our revision, all source data for the main figures has now been provided. For example, the reviewer can find for an individual data point in a given TNT quantification, the number of cell-occupied micropatterns, the number of TNT connections between cell-occupied micropatterns, etc. We believe that the information and quantitation provided are satisfactory and fulfil the high standards of EMBO Journal.

Reviewer 2, Point 3: Paragraph 2 of Results would benefit from a general description of procedure and rationale for assessing protrusions in the artificial setups used in this study of isolated cells. It would help to explicitly state when protrusions were assessed after fixation and when the observations were made with unfixed cells. What are the issues of concern with these methods and what aspects are relevant to general cell behavior? Isn't it important to point out that the conclusions regarding Arp2/3 inhibition and TNT formation are operational for the conditions used?

Reply: As suggested by the reviewer, we have added an expanded description on the rationale for assessing TNTs through our micropatterning approach. Additionally, while all pertinent information in regards to TNT quantification can be found in the Materials and Methods, we have added new text within the Results that specifies how long cells were cultured on the patterns prior to fixation for TNT quantification.

In the revised manuscript, we have updated the text within the Results as follows:

Line 152: How TNTs form and connect cells has remained elusive largely due to standard cell culture methods, as they lack the ability to control cell plating and intercellular distances.

Moreover, as TNTs are morphologically similar to filopodia, standard 2D culture methods hamper the proper identification of TNTs if cells are grown in an unconstrained manner where cell-to-cell separations approach the typical length scales that filopodia are able to achieve

Line 173: Thus, after establishing a micropatterning approach that faithfully ensures actin-driven TNT formation, we evaluated the propensity for TNTs to form as a function of increasing intercellular distances; cells were fixed following a 16−18 hr overnight culture on the patterns to give sufficient time for a basal level of TNTs to form (see Materials and Methods).

Reviewer 2, Point 4: Ln 148: If filopodia are distinguished/defined by their shorter relative length, the observation that "filopodia lengths showed that a majority of filopodia were far shorter" is not informative. Do cells with TNTs also have filopodia?

Reply: The reviewer is not wrong that this phrase is tautological (now found on **Line 185** of the revised manuscript). However, as our work is the first to demonstrate unequivocally that TNTs originating through actin polymerization clearly reach lengths greater than filopodia, and provide quantitative statistically valid data, we felt it was necessary to demonstrate that filopodia in a given cell display lengths that are far shorter (Figure 1G). As shown in Figure 2, for example, TNTs and filopodia can co-exist on the same cell.

Reviewer 2, Point 5: Does the negative effect of increasing array separation distance on frequency of TNTs suggest that the observations represent a steady state, and the possibility that the observed frequencies are measures of protrusion stability? If the experiments monitor the steady state, can the authors distinguish between stability and inherent ability to extend filopodia to longer distances? Is the conclusion (ln 151) "there seems to be an upper limit to F-actin-based elongation" justified if stability or relative rates of extension and retraction are factors? Another possibility is that the observations reflect protrusion:protrusion interactions that promote stable TNTs. There is the precedent of cytoneme:cytoneme interactions associated with stable signaling contacts (Gonzalez-Mendez et al PMID: 28825565) as well as previous work from the Zurzolo lab (Sartori-Rupp et al PMID: 30664666). A kinetic analysis in real time might be very informative.

Reply: Our interpretation is that the negative effect of increasing separation distance reflects a decrease in the probability that a given TNT can reach such extreme lengths. This decrease in probability is supported by our optical tweezer assay which shows that F-actin growth is limited to <5 μm within the pulled nanotube in the absence of Arp2/3 inhibition, but reaches far longer distances when Arp2/3 activity is inhibited. As addressed in **Reviewer 1, Point 6**, we performed additional optical tweezer experiments in which longer nanotubes reaching 40 μm in length have been pulled. These longer nanotubes permitted us to assess the probable lengths that F-actin is able to achieve in control vs. CK-666 treated conditions and more easily record instances in which F-actin infiltrated and grew to extreme lengths (>20 μm) within the pulled nanotube. These new results comprise Figure EV2.
On the other hand, the possibility of increased TNT duration (i.e., stability) is also valuable and not mutually exclusive with our above interpretation on F-actin growth and TNT distance dependency. Indeed, in our **response to Point 9 raised by Referee 1** (see full response above),

we have performed TNT duration experiments to explore this possibility and concluded that the presence of Eps8 and IRSp53 alone leads to an increased stability of TNTs in the absence of CK-666 treatment (see new Figure EV4). The lack of either an increase nor a decrease in TNT stability upon CK-666 treatment in our control experiments (Figure EV4) supports our earlier interpretation that the increased number of TNTs that we observed at larger separation distances upon Arp2/3 inhibition is afforded through the dominant effect of increased F-actin growth. However, the increased TNT lifetimes afforded by the co-expression of Eps8 and IRSp53, which was invariant to CK-666 treatment, instead reflect a stabilizing role for Eps8 and IRSp53 that promote longer-lived cell-to-cell connections. Thus, in the Discussion we have added at **Line 464** the following phrase: **"In support of this TNT-promoting Eps8-IRSp53 pathway, we found that TNT lifetimes were increased in the presence of the two proteins (Figure EV4)…"**

Reviewer 2, Point 6: Ln 158: "cells displaying only lamellipodia accounted for 4.1% of [cells with?] TNTs examined"

Reply: Based on the reviewer's suggestion, we have corrected this to now read:

Line 192: Phenotypic characterization of TNT-connected cells on *D*15 micropatterns revealed that cells displaying lamellipodia accounted for 4.1% of the examined cells with TNTs (Figure 2A and B).

Minor Revisions/Additions:

- (Figure 2C) In the original manuscript, the plot linked to the figure was erroneously an earlier version lacking some analyzed images not missing from the plots. This has been updated with a corrected Whisker box plot, and the corresponding mean percentages and total number of cell-occupied micropatterns assessed have been updated accordingly.

- While Eps8 was properly identified previously in the volcano plot in Fig. 7a (upper panel) of the original draft, we found that Eps8 was erroneously missing as a protein node in the STRING-generated network for IRSp53 in Fig. 7b. We have corrected this issue and have updated the STRING-generated network in Figure 8B to include a protein node for Eps8.

Dear Chiara,

Thank you for submitting your revised manuscript to The EMBO Journal. I sincerely apologise for the protracted assessment process due to delays in referee report submission. Your manuscript has now been seen by both original reviewers, who find that their main concerns have been addressed and now broadly recommend publication of the manuscript. I will therefore be happy to accept the manuscript for publication in The EMBO Journal after incorporation of textual adjustments as requested by reviewer #1. Please also address the following editorial points:

1. Our publisher has done their pre-publication check on your manuscript. I have attached the file here. Please take a look at the included edits in the attached manuscript file and incorporate them also in the final manuscript version.
2. Please check that the authors' names are identical and correct in the manuscript and in our online submission system. In particular, please check the correct spelling of these names: J. Michael Henderson and Thibault Chaze.
3. Please submit up to five keywords.
4. Please make sure that the order of the sections in the manuscript is as follows: abstract, introduction, results, discussion, materials & methods, data availability section, acknowledgments, disclosure statement and competing interests, references, main figure legends, tables, expanded figure legends
5. Please include the funding information in the "Acknowledgments" section of the manuscript.
6. Each movie and its legend need to be zipped together and uploaded as one folder; the legend can be provided as a README.txt file. Please update the nomenclature to Movie EV1, etc.
7. In the Appendix, please add page numbers to the table of contents. The text should be plain (currently textual changes are marked in red). Please also remove movie legends from the file as indicated above.
8. There is a reference to "data not shown" on page 12. According to our policy, which does not permit references to "data not shown", please include this information in the Appendix. Please see also https://www.embopress.org/page/journal/14602075/authorguide#unpublisheddata.
9. In our standard image check we have noticed several splice sites in figure panels 4A and 6A. In figure 6A, we find that the splice is not necessary, as in the source data both lanes are next to each other. In Fig 4A, I would recommend showing all four lanes for the GFP blot at kDa 30 and kDa 130. Additionally, in Fig. 4A, the IRSp53 blot for the precipitate, the GFP lanes contain no background signal - the panel should include also the control lanes with the background signal.
10. Please rename "Conflict of interest" section into "Disclosure and competing interests statement" (further info: https://www.embopress.org/page/journal/14602075/authorguide#conflictsofinterest).
11. CRediT has replaced the traditional author contributions section because it offers a systematic, machine-readable author contributions format that allows for more effective research assessment. Please remove the Authors Contributions from the manuscript and use the free text boxes beneath each contributing author's name in our online submission system to add specific details on the author's contribution. More information is available in our guide to authors.
12. Papers published in The EMBO Journal are accompanied online by a 'Synopsis' to enhance discoverability of the manuscript. Please submit synopsis text that consists of A) a short (1-2 sentences) summary of the findings and their significance, B) 3-4 bullet points highlighting key results and C) a synopsis image that is 550x300-600 pixels large (width x height, jpeg or png format). You can either show a model or key data in the synopsis image. Please note that the image size is rather small and that text needs to be readable at the final size.

Please feel free to contact me if you have any questions regarding this final editorial revision. Please use the link below to upload the revised files.

Thank you for the opportunity to consider your work for publication, and I look forward to receiving your revised manuscript.

With best wishes,

Ieva

Ieva Gailite, PhD
Senior Scientific Editor
The EMBO Journal
Meyerhofstrasse 1
D-69117 Heidelberg
Tel: +4962218891309
i.gailite@embojournal.org

We realize that it is difficult to revise to a specific deadline. In the interest of protecting the conceptual advance provided by the work, we recommend a revision within 3 months (29th Nov 2023). Please discuss the revision progress ahead of this time with the editor if you require more time to complete the revisions.:

Referee #1:

I commend the authors on their efforts to improve this paper and deepen our understanding of the mechanisms underpinning TNT formation. In this response, the authors provide extensive additional clarification and, in several cases, whole new lines of experimentation with new data and insights. Key new pieces of data include: new live imaging studies with F-Tractin-expressing cells showing that TNTs form via protrusion growth and collision; new Arp3 siRNA experiments that phenocopy the CK-666 results; formin overexpression screening for effects on TNT formation (in the rebuttal at least); careful analysis of F-Tractin signal in optical trap-formed TNTs; and improved rationale for several experiments. My questions and concerns were addressed in detail, and I am now supportive of publication.

Referee #2:

This work adds new and important information about the molecular processes that are the basis for nanotube formation. The revision has addressed suggestions from the previous review, but one concern that remains is the distinction the authors make between filopodia, which they define as short, and nanotubes, which they show are longer ("tens of microns") and more stable and longer lived. I think this is inaccurate as filopodia have been described (cytonemes) that also reach tens of microns and whose lifetime relative to nanotubes is not known. It is confusing and not helpful to ignore a known entity, and unwise to make distinctions that are not meaningful.

Reviewer #1:
I commend the authors on their efforts to improve this paper and deepen our understanding of the mechanisms underpinning TNT formation. In this response, the authors provide extensive additional clarification and, in several cases, whole new lines of experimentation with new data and insights. Key new pieces of data include: new live imaging studies with F-Tractin-expressing cells showing that TNTs form via protrusion growth and collision; new Arp3 siRNA experiments that phenocopy the CK-666 results; formin overexpression screening for effects on TNT formation (in the rebuttal at least); careful analysis of F-Tractin signal in optical trap-formed TNTs; and improved rationale for several experiments. My questions and concerns were addressed in detail, and I am now supportive of publication.

We thank the reviewer for the very positive feedback on the new data and explanations we included in our revised manuscript.

Reviewer #2:
This work adds new and important information about the molecular processes that are the basis for nanotube formation. The revision has addressed suggestions from the previous review, but one concern that remains is the distinction the authors make between filopodia, which they define as short, and nanotubes, which they show are longer ("tens of microns") and more stable and longer lived. I think this is inaccurate as filopodia have been described (cytonemes) that also reach tens of microns and whose lifetime relative to nanotubes is not known. It is confusing and not helpful to ignore a known entity, and unwise to make distinctions that are not meaningful.

We apologize as it was not our intent to ignore cytonemes. We have updated our manuscript to reflect the ability of cytonemes to also reach long distances, and further clarified that the protrusions we refer to as canonical (conventional) filopodia are those that are short and dynamic in nature. Furthermore, we have added new material within the Discussion section to highlight similarities in our IRSp53 K/D results with results showing that the expression of a dominant-negative IRSp53 mutant led to abnormal cytoneme formation.

Line 77: To establish cell-to-cell communication at longer distances spanning several cell diameters, cells remarkably construct actin-based protrusions such as cytonemes (often termed as specialized signalling filopodia) (Yamashita *et al*, 2018) and tunneling nanotubes (TNTs) (Ljubojevic *et al*, 2021) that, while exhibit comparable widths to conventional filopodia, are able to reach significantly longer distances (tens of microns). For clarity, we will refer to the short, dynamic protrusions cells make simply as filopodia or qualify them as conventional (canonical) filopodia.

Line 85: While both cytonemes and TNTs are similar in terms of their capacity to reach extreme intercellular distances, the two differ in their cellular functionality. Cytonemes, originally identified in the developing wing tissue of *Drosophila* (Felipe-Andrés & Kornberg, 1999), direct morphogen signalling such as the binding of Wnt ligands with Frizzled receptors on another cell (Stanganello & Scholpp, 2016) through closed-ended structures. Contrastingly, TNTs instead were functionally found to cytoplasmically connect distant cells (Rustom *et al*, 2004),

Line 477: This result bares similarity with recent work on cytonemes showing that the overexpression of a dominant-negative IRSp53 mutant perturbed in its membrane binding capacity led to shortened cytonemes (Brunt *et al*, 2021).

New References pertaining to cytonemes:

Brunt L, Greicius G, Rogers S, Evans BD, Virshup DM, Wedgwood KCA & Scholpp S (2021) Vangl2 promotes the formation of long cytonemes to enable distant Wnt/β-catenin signaling. *Nat Commun* 12: 2058

Felipe-Andrés R-W & Kornberg T (1999) Cytonemes: Cellular Processes that Project to the Principal Signaling Center in Drosophila Imaginal Discs. *Cell* 97: 599–607

Stanganello E & Scholpp S (2016) Role of cytonemes in Wnt transport. *J Cell Sci* 129: 665–672

Yamashita YM, Inaba M & Buszczak M (2018) Specialized Intercellular Communications via Cytonemes and Nanotubes. *Annu Rev Cell Dev Biol* 34: 59–84

2nd Revision - Editorial Decision 2nd Nov 2023

Dear Chiara,

Thank you for addressing most of the final editorial issues. I am now pleased to inform you that your manuscript has been accepted for publication.

Before we forward your manuscript to our publishers, there remain a couple of points that need addressing. I would need your input on this until tomorrow to ensure timely publication of your article. I am sorry for this urgency, but I hope that the issues are minor enough to be rapidly solved.
1. I appreciate your reasoning for including GFP bands from two molecular weights in a single panel for Fig 4A. However, please insert vertical dark separating lines in the figure to indicate that these sections come from different areas of the gel. You can send me the correct file via email.
2. I would like to propose a couple of minor changes in the article title, abstract and synopsis to increase their accessibility to our more general readership. I have also written a short blurb that will accompany the title of your manuscript in our online table of contents. Please take a look at the text below and in the attached manuscript text file and let me know if any corrections are necessary.

Title:
Tunneling nanotube formation is driven by Eps8/IRSp53-dependent linear actin polymerization

Blurb:
Inhibition of branched actin networks reveals the role of linear actin filament polymerization in the formation of functional tunneling nanotubes.

Synopsis
Tunneling nanotubes (TNTs) are specialized actin-based protrusions that bridge distant cells to facilitate direct intercellular communication and cargo transfer in both physiological and pathological contexts. Here, linear actin filament elongation induced by Eps8 and IRSp53 is shown to promote functional and stable TNT formation.
• A micropatterning approach shows that TNTs originate via actin polymerization rather than cell dislodgement and exceed the length of conventional filopodia.
• Inhibition of Arp2/3 activity promotes functional TNT formation due to enhanced actin filament polymerization and elongation, suggesting a shift from branched to linear actin.
• An interaction between Eps8 and the I-BAR domain protein IRSp53 promotes formation of functional TNTs with an increased lifetime.
• Arp2/3 inhibition leads to reduction of Eps8 interactions with proteins facilitating actin filament depolymerization and turnover.

Please note that it is EMBO Journal policy for the transcript of the editorial process (containing referee reports and your response letter) to be published as an online supplement to each paper. If you do NOT want this, you will need to inform the Editorial Office via email immediately. More information is available here:
https://emboj.embopress.org/about#Transparent_Process

Your manuscript will be processed for publication in the journal by EMBO Press. Manuscripts in the PDF and electronic editions of The EMBO Journal will be copy edited, and you will be provided with page proofs prior to publication. Please note that supplementary information is not included in the proofs.

Please note that you will be contacted by Wiley Author Services to complete licensing and payment information. The 'Page Charges Authorization Form' is available here: https://www.embopress.org/pb-assets/embo-site/tej_apc.pdf

EMBO Press participates in many Publish and Read agreements that allow authors to publish Open Access with reduced/no publication charges. Check your eligibility: https://authorservices.wiley.com/author-resources/Journal-Authors/open-access/affiliation-policies-payments/index.html

Should you be planning a Press Release on your article, please get in contact with embojournal@wiley.com as early as possible, in order to coordinate publication and release dates.

If you have any questions, please do not hesitate to call or email the Editorial Office. Thank you again for this contribution to The EMBO Journal and congratulations on a successful publication!

Best regards,

Ieva

Ieva Gailite, PhD
Senior Scientific Editor
The EMBO Journal
Meyerhofstrasse 1
D-69117 Heidelberg
Tel: +4962218891309
i.gailite@embojournal.org

***

Rev_Com_number: RC-2022-01766
New_manu_number: EMBOJ-2023-113761R1
Corr_author: Zurzolo
Title: A balance of actin and Eps8/IRSp53 usage in branched versus linear networks determines TNT formation

EMBO Press Author Checklist

Corresponding Author Name: Chiara Zurzolo	
Journal Submitted to: The EMBO Journal	
Manuscript Number: EMBOJ-2023-13761	

USEFUL LINKS FOR COMPLETING THIS FORM
The EMBO Journal - Author Guidelines
EMBO Reports - Author Guidelines
Molecular Systems Biology - Author Guidelines
EMBO Molecular Medicine - Author Guidelines

Reporting Checklist for Life Science Articles (updated January
This checklist is adapted from Materials Design Analysis Reporting (MDAR) Checklist for Authors. MDAR establishes a minimum set of requirements in transparent reporting in the life sciences (see Statement of Task: 10.31222/osf.io/9sm4x). Please follow the journal's guidelines in preparing your
Please note that a copy of this checklist will be published alongside your article.

Abridged guidelines for figures
1. Data
The data shown in figures should satisfy the following conditions:
- the data were obtained and processed according to the field's best practice and are presented to reflect the results of the experiments in an accurate and unbiased manner.
- ideally, figure panels should include only measurements that are directly comparable to each other and obtained with the same assay.
- plots include clearly labeled error bars for independent experiments and sample sizes. Unless justified, error bars should not be shown for technical
- if n<5, the individual data points from each experiment should be plotted. Any statistical test employed should be justified.
- Source Data should be included to report the data underlying figures according to the guidelines set out in the authorship guidelines on Data

2. Captions
Each figure caption should contain the following information, for each panel where they are relevant:
- a specification of the experimental system investigated (eg cell line, species name).
- the assay(s) and method(s) used to carry out the reported observations and measurements.
- an explicit mention of the biological and chemical entity(ies) that are being measured.
- an explicit mention of the biological and chemical entity(ies) that are altered/varied/perturbed in a controlled manner.
- the exact sample size (n) for each experimental group/condition, given as a number, not a range;
- a description of the sample collection allowing the reader to understand whether the samples represent technical or biological replicates (including how many animals, litters, cultures, etc.).
- a statement of how many times the experiment shown was independently replicated in the laboratory.
- definitions of statistical methods and measures:
  - common tests, such as t-test (please specify whether paired vs. unpaired), simple χ2 tests, Wilcoxon and Mann-Whitney tests, can be unambiguously identified by name only, but more complex techniques should be described in the methods section;
  - are tests one-sided or two-sided?
  - are there adjustments for multiple comparisons?
  - exact statistical test results, e.g., P values = x but not P values < x;
  - definition of 'center values' as median or average;
  - definition of error bars as s.d. or s.e.m.

Please complete ALL of the questions below.
Select "Not Applicable" only when the requested information is not relevant for your study.

Materials

Newly Created Materials	Information included in the manuscript?	In which section is the information available? (Reagents and Tools Table, Materials and Methods, Figures, Data Availability Section)
New materials and reagents need to be available; do any restrictions apply?	Not Applicable	

Antibodies	Information included in the manuscript?	In which section is the information available? (Reagents and Tools Table, Materials and Methods, Figures, Data Availability Section)
For **antibodies** provide the following information:<br>- Commercial antibodies: RRID (if possible) or supplier name, catalogue number and or/clone number<br>- Non-commercial: RRID or citation	Yes	Materials and Methods

DNA and RNA sequences	Information included in the manuscript?	In which section is the information available? (Reagents and Tools Table, Materials and Methods, Figures, Data Availability Section)
**Short novel DNA or RNA including primers, probes**: provide the sequences.	Yes	Materials and Methods

Cell materials	Information included in the manuscript?	In which section is the information available? (Reagents and Tools Table, Materials and Methods, Figures, Data Availability Section)
**Cell lines:** Provide species information, strain. Provide accession number in repository **OR** supplier name, catalog number, clone number, and/**OR** RRID.	Yes	Materials and Methods
**Primary cultures:** Provide species, strain, sex of origin, genetic modification status.	Not Applicable	
Report if the cell lines were recently **authenticated** (e.g., by STR profiling) and tested for mycoplasma contamination.	Not Applicable	

Experimental animals	Information included in the manuscript?	In which section is the information available? (Reagents and Tools Table, Materials and Methods, Figures, Data Availability Section)
**Laboratory animals or Model organisms:** Provide species, strain, sex, age, genetic modification status. Provide accession number in repository **OR** supplier name, catalog number, clone number, **OR** RRID.	Not Applicable	
**Animal observed in or captured from the field:** Provide species, sex, and age where possible.	Not Applicable	
Please detail **housing and husbandry conditions**.	Not Applicable	

Plants and microbes	Information included in the manuscript?	In which section is the information available? (Reagents and Tools Table, Materials and Methods, Figures, Data Availability Section)
**Plants:** provide species and strain, ecotype and cultivar where relevant, unique accession number if available, and source (including location for collected wild specimens).	Not Applicable	
**Microbes:** provide species and strain, unique accession number if available, and source.	Not Applicable	

Human research participants	Information included in the manuscript?	In which section is the information available? (Reagents and Tools Table, Materials and Methods, Figures, Data Availability Section)
If collected and within the bounds of privacy constraints report on age, sex and gender or ethnicity for all study participants.	Not Applicable	

Core facilities	Information included in the manuscript?	In which section is the information available? (Reagents and Tools Table, Materials and Methods, Figures, Data Availability Section)
If your work benefited from core facilities, was their service mentioned in the acknowledgments section?	Yes	Acknowledgements

Design

Study protocol	Information included in the manuscript?	In which section is the information available? (Reagents and Tools Table, Materials and Methods, Figures, Data Availability Section)
If study protocol has been **pre-registered, provide DOI in the manuscript**. For clinical trials, provide the trial registration number **OR** cite DOI.	Not Applicable	
Report the **clinical trial registration number** (at ClinicalTrials.gov or equivalent), where applicable.	Not Applicable	

Laboratory protocol	Information included in the manuscript?	In which section is the information available? (Reagents and Tools Table, Materials and Methods, Figures, Data Availability Section)
Provide DOI OR other citation details if **external detailed step-by-step protocols** are available.	Not Applicable	

Experimental study design and statistics	Information included in the manuscript?	In which section is the information available? (Reagents and Tools Table, Materials and Methods, Figures, Data Availability Section)
Include a statement about **sample size** estimate even if no statistical methods were used.	Not Applicable	
Were any steps taken to minimize the effects of subjective bias when allocating animals/samples to treatment (e.g. **randomization procedure**)? If yes, have they been described?	Yes	Materials and Methods
Include a statement about **blinding** even if no blinding was done.	Not Applicable	
Describe **inclusion/exclusion criteria** if samples or animals were excluded from the analysis. Were the criteria pre-established?<br><br>If sample or data points were omitted from analysis, report if this was due to attrition or intentional exclusion and provide justification.	Not Applicable	
For every figure, are **statistical tests** justified as appropriate? Do the data meet the assumptions of the tests (e.g., normal distribution)? Describe any methods used to assess it. Is there an estimate of variation within each group of data? Is the variance similar between the groups that are being statistically compared?	Yes	Figures

Sample definition and in-laboratory replication	Information included in the manuscript?	In which section is the information available? (Reagents and Tools Table, Materials and Methods, Figures, Data Availability Section)
In the figure legends: state number of times the experiment was **replicated** in laboratory.	Yes	Figures
In the figure legends: define whether data describe **technical or biological replicates**.	Yes	Figures

Ethics

Ethics	Information included in the manuscript?	In which section is the information available? (Reagents and Tools Table, Materials and Methods, Figures, Data Availability Section)
Studies involving **human participants**: State details of **authority granting ethics approval** (IRB or equivalent committee(s), provide reference number for approval.	Not Applicable	
Studies involving **human participants**: Include a statement confirming that **informed consent** was obtained from all subjects and that the experiments conformed to the principles set out in the WMA Declaration of Helsinki and the Department of Health and Human Services Belmont Report.	Not Applicable	
Studies involving **human participants: For** publication of **patient photos**, include a statement confirming that consent to publish was obtained.	Not Applicable	
Studies involving experimental **animals**: State details of **authority granting ethics approval** (IRB or equivalent committee(s), provide reference number for approval. Include a statement of compliance with ethical regulations.	Not Applicable	
Studies involving **specimen and field samples:** State if relevant **permits** obtained, provide details of authority approving study; if none were required, explain why.	Not Applicable	

Dual Use Research of Concern (DURC)	Information included in the manuscript?	In which section is the information available? (Reagents and Tools Table, Materials and Methods, Figures, Data Availability Section)
Could your study fall under dual use research restrictions? Please check biosecurity documents and list of **select agents and toxins** (CDC): https://www.selectagents.gov/sat/list.htm	Not Applicable	
If you used a select agent, is the security level of the lab appropriate and reported in the manuscript?	Not Applicable	
If a study is subject to dual use research of concern regulations, is the name of the **authority granting approval and reference number** for the regulatory approval provided in the manuscript?	Not Applicable	

Reporting

The MDAR framework recommends adoption of discipline-specific guidelines, established and endorsed through community initiatives. Journals have their own policy about requiring specific guidelines and recommendations to complement MDAR.

Adherence to community standards	Information included in the manuscript?	In which section is the information available? (Reagents and Tools Table, Materials and Methods, Figures, Data Availability Section)
State if relevant guidelines or checklists (e.g., **ICMJE, MIBBI, ARRIVE, PRISMA**) have been followed or provided.	Not Applicable	
For **tumor marker prognostic studies,** we recommend that you follow the **REMARK** reporting guidelines (see link list at top right). See author guidelines, under 'Reporting Guidelines'. Please confirm you have followed these guidelines.	Not Applicable	
For **phase II and III randomized controlled trials**, please refer to the **CONSORT** flow diagram (see link list at top right) and submit the CONSORT checklist (see link list at top right) with your submission. See author guidelines, under 'Reporting Guidelines'. Please confirm you have submitted this list.	Not Applicable	

Data Availability

Data availability	Information included in the manuscript?	In which section is the information available? (Reagents and Tools Table, Materials and Methods, Figures, Data Availability Section)
Have **primary datasets** been deposited according to the journal's guidelines (see 'Data Deposition' section) and the respective accession numbers provided in the Data Availability Section?	Yes	Data availability
Were **human clinical and genomic datasets** deposited in a public access-controlled repository in accordance to ethical obligations to the patients and to the applicable consent agreement?	Not Applicable	
Are **computational models** that are central and integral to a study available without restrictions in a machine-readable form? Were the relevant accession numbers or links provided?	Not Applicable	
If publicly available data were reused, provide the respective **data citations in the reference list**.	Not Applicable	
